# Single electron/energy transfer photocatalysis: α-/β-switchable synthesis of 3-deoxy-d-manno-oct-2-ulosonic acid *O*-glycosides†

**DOI:** 10.1039/d5sc02980e

**Published:** 2025-06-13

**Authors:** Jing-dong Zhang, Jia-long Jie, Shu-yi Yan, Hui Zhang, Jia-meng Chen, Jiang-cheng Wu, Lu-yang Qin, Guang-jian Liu, Hong-mei Su, Guo-wen Xing

**Affiliations:** a College of Chemistry, Beijing Normal University Beijing 100875 P. R. China gwxing@bnu.edu.cn jialong@bnu.edu.cn

## Abstract

Stereoselective glycosidation of 3-deoxy-d-manno-oct-2-ulosonic acid (Kdo) has emerged as a focal point in glycoscience, attributed to the burgeoning identification of naturally occurring α- or β-Kdo glycosides within the glycoconjugate structures of various organisms. Nonetheless, advancements in α-/β-switchable stereoselective Kdo *O*-glycosidation remain scarce due to the complicated synthesis of Kdo donors and the complex chemical environment at the anomeric carbon of Kdo. Herein, inspired by the property that the conditions of the photocatalytic reaction can be facilely controlled and mediated, we report an efficient photocatalytic Ir^III^/Cu^II^-catalysed Kdo *O*-glycosidation for the stereoselective synthesis of both α- and β-Kdo *O*-glycosides with the dual mediation of MeCN and (*p*-Tol)_2_SO. Within a facile photoreactor, the glycosidation reactions were carried out at −78 °C to generate β-Kdo *O*-glycosides in excellent yields (up to 99%) *via* the glycosyl nitrilium ion, and at −30 °C to generate α-Kdo *O*-glycosides in good yields (57–99%) *via* the oxosulfonium ion. Two crystals of α-Kdo *O*-glycosides were cultivated to assess the stereochemical configurations. Subsequently, laser flash photolysis, steady-state measurement and ESR spectral measurement were conducted to first reveal a single electron transfer (SET) together with the Dexter energy transfer (EnT) process of the photocatalytic activation by monitoring the trifluoromethyl radical, the cation radical of dibenzothiophene and the cation radical of 4,5,7,8-tetra-*O*-acetyl-Kdo *p*-toluenethioglycoside. (TD)-DFT calculations further supported this process and illustrated a S_N_2-like mechanism for the attack of hydroxyl acceptors.

## Abstract 2

An acetonitrile/(*p*-Tol)_2_SO dual mediated photocatalytic Ir^III^/Cu^II^-catalysed Kdo *O*-glycosidation was developed to synthesize α-/β-Kdo *O*-glycosides in good to excellent yields *via* a dual pathway (SET & EnT) photoactivation and a S_N_2-like mechanism.

## Graphical abstract

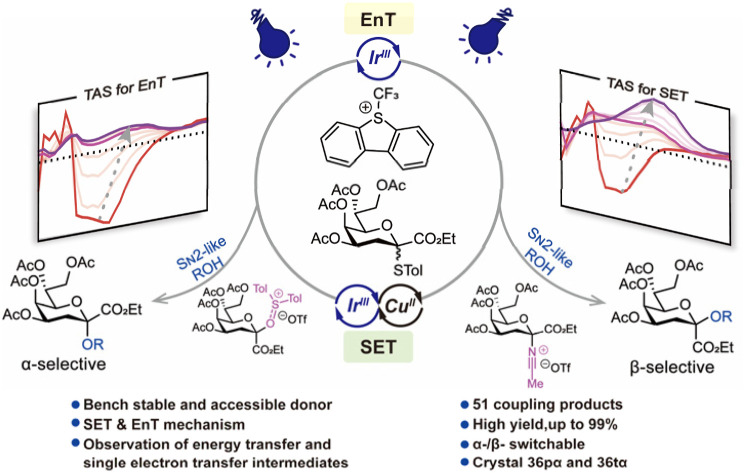

## Introduction

**Fig. 1:**
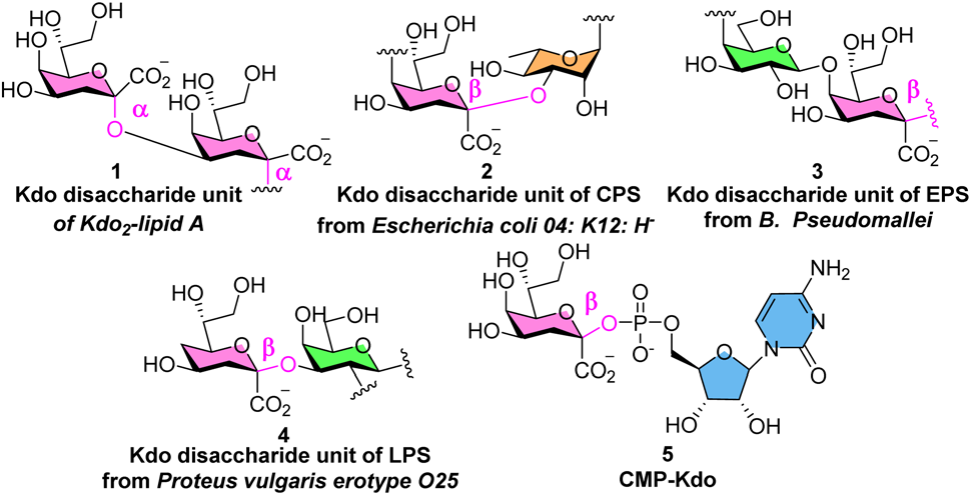
Naturally occurring compounds containing the Kdo structure.

3-Deoxy-d-manno-oct-2-ulosonoic acid (Kdo) is a nonmammalian eight-carbon monosaccharide.^1,2^ Kdo mostly exists as α-Kdo glycosides in nature, which are the smallest unit of Kdo_2_-lipid A in the lipopolysaccharide (LPS) of Gram-negative bacteria.^3^ In contrast, the β-Kdo glycosides are mainly located in bacterial capsular polysaccharides (CPSs)^4^ as well as the extracellular exopolysaccharides (EPS)^5^ (Fig. 1, 1–3). β-Kdo disaccharide unit 4 was also found in the core region of LPS from *Proteus vulgaris* serotype O25. Moreover, in the biosynthetic pathway of natural Kdo glycosides, cytidine-5′-monophospho-Kdo 5 (CMP-Kdo), features a β-linkage of the Kdo moiety to facilitate the assembly of bacterial glycans as the principal substrate for glycosyltransferases.^6^

Since Kdo glycosides can be recognized by the human native and adaptive immune systems, the stereoselective synthesis of Kdo glycosides has attracted increasing attention in recent years.^7^ However, stereoselective and efficient synthesis of Kdo glycosides remains a great challenge, due to the presence of the electron-withdrawing carboxylic group at the C1 position, which not only reduces the reaction activity but also allows the elimination reaction to occur more easily to generate the 2,3-glycal byproduct. In addition, the lack of a hydroxyl group at the C3 position makes it difficult to completely control the stereochemistry of the glycosidation reaction by using the neighboring group participation.^7,8^

**Scheme 1:**
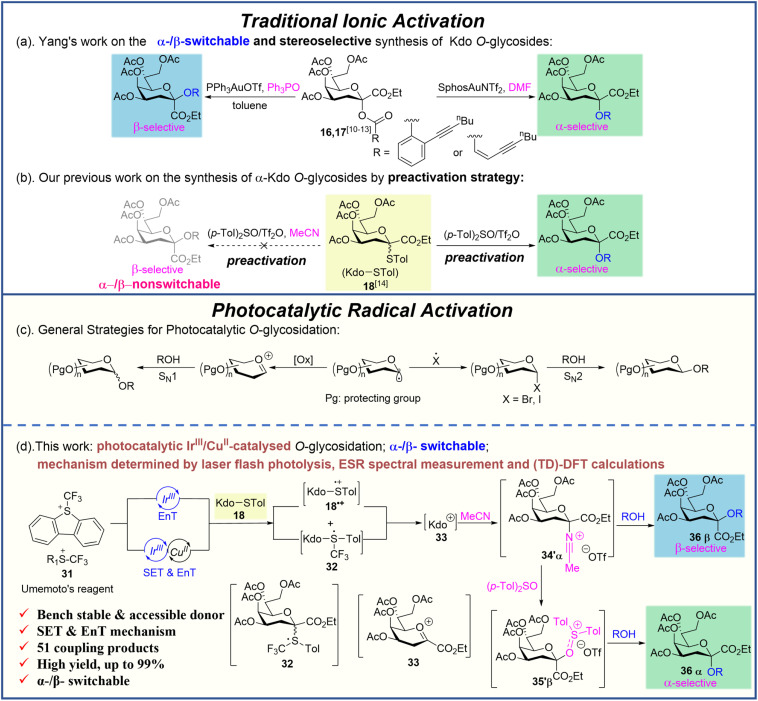
Brief introduction of α-/β-stereoselective Kdo *O*-glycosidation, photocatalytic *O*-glycosidation and our photocatalytic Ir^III^/Cu^II^-catalysed Kdo *O*-glycosidation.

In recent years, based on the ionic activation mechanism, various strategies^7,9^ have been developed for the efficient synthesis of α-Kdo glycosides (Fig. S1,†6–25) or β-Kdo glycosides (Fig. S1,†26–30). Nevertheless, only the Au(i)-catalyzed Kdo *O*-glycosidation developed by Yang's group^10–13^ achieved the α-/β-switchable stereoselective Kdo *O*-glycosidation by adding DMF or not (Scheme 1a). Recently, using the 4,5,7,8-tetra-*O*-acetyl-Kdo *p*-toluenethioglycoside 18 as the donor, by the (*p*-Tol)_2_SO/Tf_2_O preactivation strategy, our group successfully obtained α-Kdo *O*-glycosides (Scheme 1b) with the mediation of excess (*p*-Tol)_2_SO, but failed to synthesize the β-Kdo *O*-glycosides with the mediation of acetonitrile (MeCN).^14^ Therefore, a more general and efficient method for stereoselectively synthesizing both α- and β-Kdo glycosides with high yields and accessible donors still needs to be developed.

Photocatalytic reactions, due to their controllable reaction conditions and widespread applications in organic synthesis, have attracted a lot of attention from more and more scientific researchers.^15–17^ However, photocatalysis is addressed relatively less in carbohydrate chemistry, especially in *O*-glycosidation.^18^ To obtain *O*-glycosides during glycosidation, usually the nucleophile needs to attack the widely accepted intermediate oxacarbenium ion, while in photocatalytic glycosidation the special intermediate glycosyl radical needs to attack the double bond or combine with the transition metal catalyst to achieve *C*-glycosides or *N*-glycosides, which means that such a radical process tolerates hydroxyl groups.^18^ Thus, two different methods for generating *O*-glycosides during photocatalytic glycosidation were developed in recent years (Scheme 1c). One is to oxidize the glycosyl radicals into oxacarbenium ions or directly generate them with light induced catalytic activation.^19–24^ The other is to transfer the glycosyl radicals into less active glycosides such as bromo glycosides^25^ and iodine glycosides^26^ to obtain the target products through the S_N_2 process.

Umemoto's reagent, as the precursor of the highly electrophilic trifluoromethyl radical (CF_3_˙), was disclosed to activate thioglycoside donors^20,21^ or selenoglycoside donors^24^ for the synthesis of *O*-glycosides. However, the detailed mechanism of photocatalytic *O*-glycosidation with thioglycoside as the donor and Umemoto's reagent as the promoter remained obscure. Furthermore, to date, no photocatalytic Kdo glycosidations have been reported.

Herein, considering the increasing importance of both α- and β-Kdo glycosides^3–7,27^ and the versatility of the accessible thioglycoside donors,^14,28,29^ we reported a photocatalytic Ir^III^/Cu^II^-catalysed and MeCN/(*p*-Tol)_2_SO dual mediated Kdo *O*-glycosidation using 4,5,7,8-tetra-*O*-acetyl-Kdo *p*-toluenethioglycoside 18 as the donor, Ir[dF(CF_3_)ppy]_2_(dtbbpy)PF_6_ (Ir^III^) as the photocatalyst and Umemoto's reagent as the light-driven activator for the stereoselective synthesis of both α- and β- Kdo *O*-glycosides (Scheme 1d). Moreover, detailed mechanism studies were conducted to clarify the reaction process of photocatalytic glycosidation.

## Results and discussion

### Glycosidation study

To determine the stereochemistry of Kdo glycoside, the selective proton decoupled ^13^C NMR spectra were used to obtain the coupling constant between the H_ax_ at C3 and the carbon atom at the C1-position (^3^*J*_C1/H3axial_).^30^ Generally, the ^3^*J*_C1/H3axial_ value of α-anomer is ≤1.0 Hz, while the β-anomer's value is 5.0–6.0 Hz.^31^

**Fig. 2:**
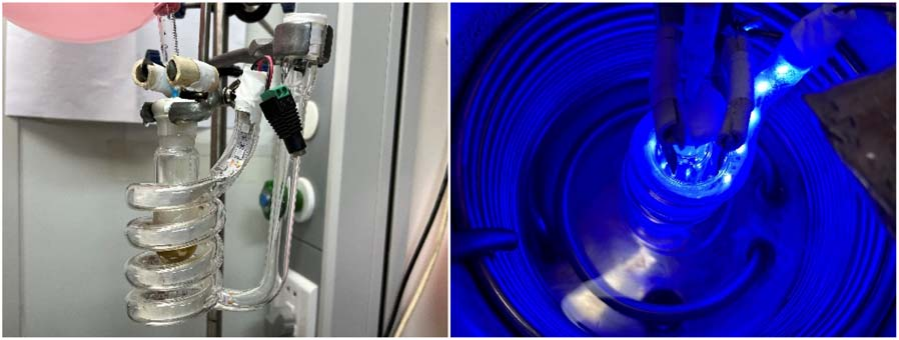
The photocatalytic glycosidation apparatus designed, constructed and applied in this work.

According to the previous reports on Kdo glycosidation,^7^ it is necessary to conduct the glycosidation reactions at low temperatures to achieve better stereoselectivities and higher yields. Consequently, we designed and constructed a facile, accessible, and cost-effective photocatalytic glycosidation apparatus for this study (Fig. 2). The apparatus comprises an inexpensive LED light strip integrated within a glass helical tube, a reaction flask, and a low-temperature thermostatic reaction bath. The glass helical tube enables the direct immersion of the LED light strip in ethanol, thereby facilitating a photocatalytic reaction within the bath at low temperatures. The external lighting apparatus allows for superior sealing of the reaction environment. Based on the low power consumption of the LED light source, the reaction can be safely maintained for prolonged periods.

The model photocatalytic glycosidation between Kdo thioglycoside donor 18 and 1-adamantanol S15 (Fig. S2†) was conducted under specific conditions (Table S1,† entry 1), resulting in moderate yield (70%) but poor stereoselectivity (*α* : *β* = 1.4 : 1). Since the glycosidation reactions are complex and affected by many factors,^32^ the optimization of the photocatalytic glycosidation reaction was carried out based on multiple factors respectively (Tables 1 and S1–S5†).

**Table 1:** Comparison of reaction components

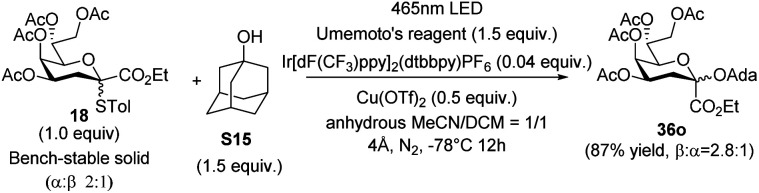			
Entry	Variation from labelled conditions	Yield^a^	*β* : *α*^b^
1	None	87%	2.8 : 1
2	No Umemoto's reagent	6%^94%^	1.5 : 1
3	No light	NR	ND
4	1 : 1 instead of 2 : 1 (the *α*/*β* ratio of donors)	88%	2.8 : 1
5	3.0 equiv. of PBN as the additive	NR	ND
6^c^	3.4 equiv. of (*p*-Tol)_2_SO as the additive	99%	1 : 13

a Isolated yields and superscripts indicating the amount of donor regained.

b Determined by ^1^H NMR.

c 1.5 equiv. of Cu(OTf)_2_ and reacting at −30 °C with MeCN/DCM = 2/1 as the solvent. NR: not reaction. ND: not determined.

To verify the oxidative effect of Cu(OTf)_2_ (Cu^II^) in photocatalytic Kdo *O*-glycosidation, a series of experiments were performed as shown in Table S1.† The results showed that Cu^II^ could accelerate the process of the reaction as the catalyst, which improved the activation of donors (Table S1,† entry 3 and 5). Besides, when changing the amount of Cu(OTf)_2_, the stereoselectivity of glycosidation was constant (Table S1,† entry 1–5), indicating that Cu^II^ could not participate in the process of nucleophilic attack of acceptors.

Since temperature is a key factor for the reaction, especially in Kdo glycosidation reactions,^14,32^ the influence of temperature was investigated carefully (Table S2†). Apparently, lower temperature could benefit the stabilization of the reactive intermediate and lead to higher yield (−78 °C, 87%, *β* : *α* = 2.8 : 1). It was worth noting that there was a transition of the stereoselectivity from *α* to *β* with the decrease in temperature (Table S2,† entry 2–5). Considering the effect of acetonitrile in the glycosidation reaction,^28^ the mixed solvent system was changed to clarify the relationship between solvent and Kdo glycosidation (Table S3†). On reducing the amount of acetonitrile, both the yield and the β-stereoselectivity decreased, indicating that acetonitrile could control the stereoselectivity by reacting with the reactive intermediate oxacarbenium ion 33 to form the more stable intermediate glycosyl nitrilium ions 34′α (Scheme 1c). Further density functional theory (DFT) calculation and analysis^33^ supported such a conclusion (Scheme 5).

Based on our previous work on the (*p*-Tol)_2_SO/Tf_2_O preactivation strategy of Kdo *O*-glycosidation,^14^ (*p*-Tol)_2_SO is an important additive that could efficiently modulate reaction yields and lead to α-stereoselectivities by forming the reported and verified oxosulfonium salts 35 (ref. 14, 34 and 35) (Scheme 1c). Thus, the effect of the amount of (*p*-Tol)_2_SO on the photocatalytic Kdo *O*-glycosidation was investigated carefully, as shown in Table S4.† To our delight, (*p*-Tol)_2_SO as an additive could obviously improve both the yield and the α-stereoselectivity of the photocatalytic Kdo *O*-glycosidation (Table 1, entry 6). However, adding excess (*p*-Tol)_2_SO to 4.0 equivalent led to a decrease in yield (Table S4,† entry 2–3), suggesting that the excess (*p*-Tol)_2_SO could suppress the activation reaction of the donor.

Photocatalysts (PC) were also evaluated (Table S5†). It was found that PC had a weak correlation with the stereoselectivity of glycosidation but strong correlation with the yield of glycosidation, indicating that the PC could not participate in the nucleophilic attack of acceptors towards the reactive intermediate but only influenced the activation of the donor. It was important to note that the yield was highly correlated with the *E*_1/2 PC^+^/PC_, indicating that the PC^+^ species could be a vital part of the photocatalytic cycle.

Next, the preliminary studies on the mechanism of photocatalytic Kdo *O*-glycosidation were conducted as shown in Table 1. The photocatalytic Kdo *O*-glycosidation was not affected by the *α*/*β* ratio of Kdo donors (Table 1, entry 1 and 4), showing that the target products were produced through the intermediates instead of the glycosyl donors. Furthermore, the addition of *N-tert*-butyl-α-phenylnitrone (PBN) as the radical scavenger significantly suppresses the reaction, thereby suggesting that the reaction was initiated through the generation of radical species (Table 1, entry 2, 3 and 5).

**Scheme 2:**
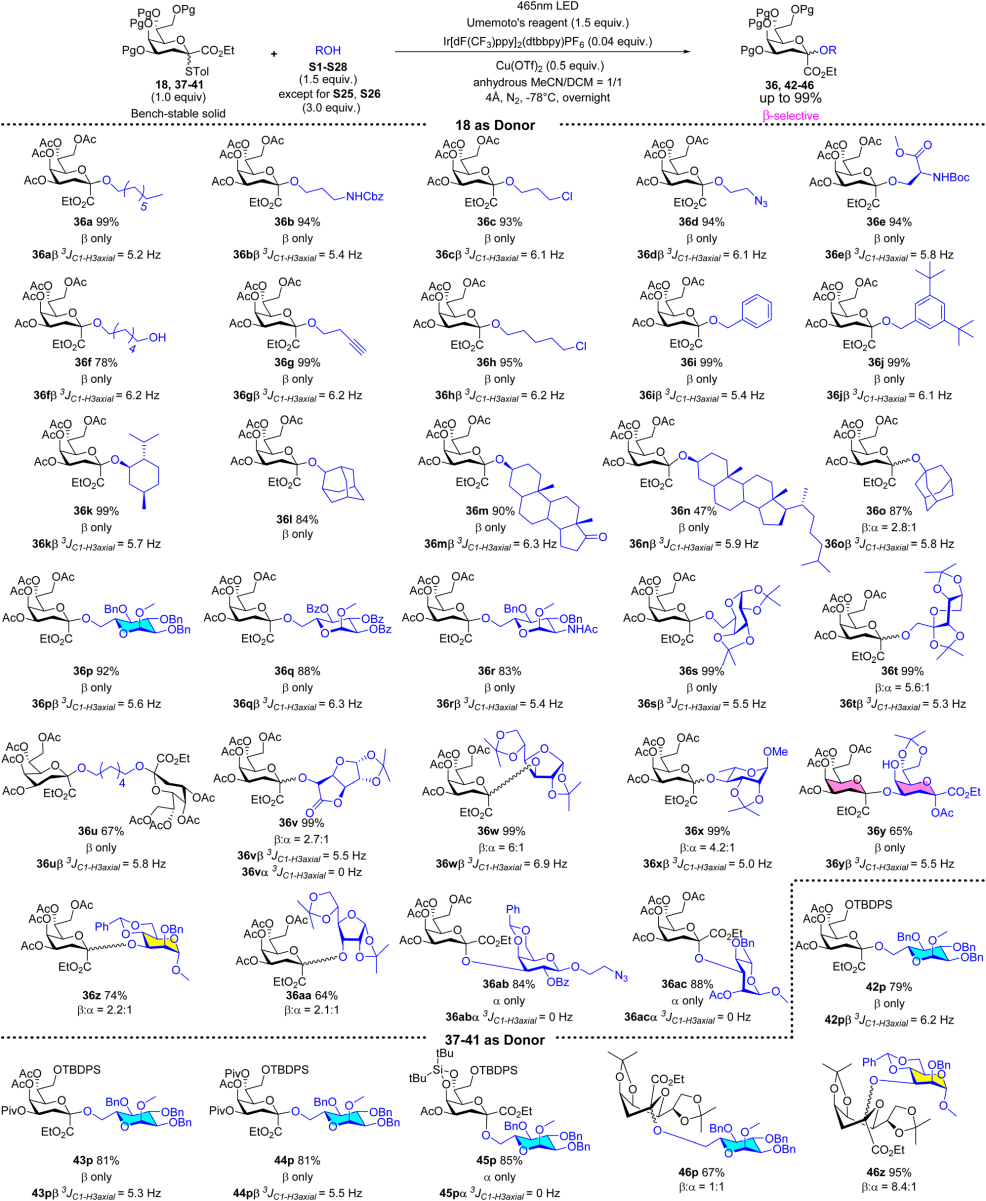
β-Selective photocatalytic Kdo *O*-glycosidation of 18 with a series of acceptors and 37–41 with S16 as the acceptor.

Under the optimized reaction conditions (1.0 equiv. of 18, 1.5 equiv. of acceptor, 1.5 equiv. of Umemoto's reagent, 0.04 equiv. of Ir[dF(CF_3_)ppy]_2_(dtbbpy)PF_6_, and 0.5 equiv. of Cu(OTf)_2_, −78 °C 12 h, in acetonitrile/dichloromethane = 1/1), a series of glycosyl acceptors shown in Fig. S2† were examined for the Kdo glycosidation reactions. As shown in Scheme 2, when primary or secondary non-carbohydrate alcohols (S1–S14) were used as acceptors to be coupled with 18, the desired β-Kdo *O*-glycosides (36aβ–36nβ) were successfully obtained in favorable yields (78–99%) and excellent stereoselectivities. Since dihydrocholesterol S14 had poor solubility in acetonitrile and its mixed solvent, the yield was moderate (47%) compared with epiandrosterone S13 (90%). For tertiary alcohol 1-ada, due to its larger steric hindrance and weaker nucleophilic properties, glycosidation might proceed *via* an S_N_2-like mechanism, resulting in moderate β-stereoselectivity (36o, *α*/*β* = 1 : 2.7). On employing sugar alcohols as acceptors, the glycosidation reactions were more complicated. For primary sugar alcohols S16–S20 as acceptors, Kdo glycosidation with 18 also furnished excellent β-selective *O*-glycosides (36pβ–36tβ) in high yields (83–99%). But for secondary sugar alcohols or their derivatives as acceptors, the results showed that the stereoselectivities of glycosidation depended on the steric hindrance of acceptors (36v–36ac). Greater steric hindrance and less nucleophilic ability might cause the mechanism to be S_N_1-like, leading to poor β-stereoselectivity (36z and 36aa) or α-stereoselectivity (glycosyl *cis*-diol S27 as the acceptor to generate 36abα and glycosyl *cis*-diol S28 as the acceptor to generate 36acα, respectively). Notably, using 0.5 equivalent of 1,6-hexanediol S6 in glycosidation, the desired pseudo β-Kdo disaccharide 36uβ was obtained in a good yield (67%). Moreover, the β-Kdo-(2 → 4)-α-Kdo-OAc disaccharide 36yβ was successfully obtained in good yield (65%) using this strategy, demonstrating the method's excellent tolerance for glycosyl acetates, which contrasts with traditional ionic glycosidation.

On changing the protecting group from the acetyl group to other electron-withdrawing protecting groups (Piv, 37–39, Scheme S1†) the reactivity and stereoselectivity could be maintained to generate β-Kdo *O*-glycosides (42pβ–44pβ) in high yields. For Si-protected donor 40 (Scheme S1†), it could be effectively activated, although the regulatory role of acetonitrile is not significant in this case. The product was α-Kdo *O*-glycoside 45pα, indicating that the bulky protecting group effectively restricted the reaction space on the donor's β-face.^9*e*^ Additionally, when using armed Kdo glycosyl donor 41 (Scheme S1†) to furnish *O*-glycosides (46p and 46z (ref. 28)), the role of acetonitrile was consistent with that reported in the traditional ionic activation glycosidation.

**Scheme 3:**
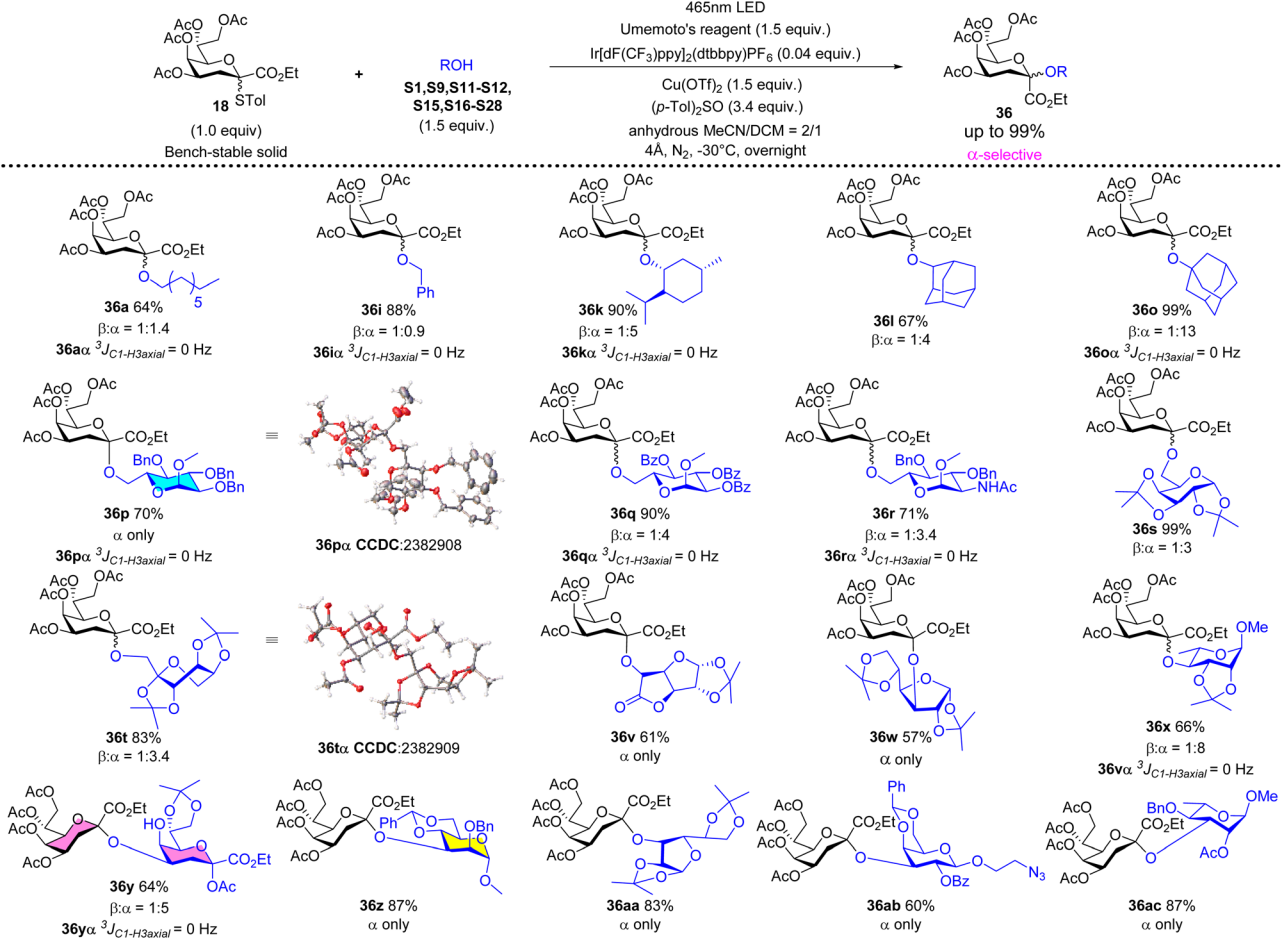
α-Selective photocatalytic Kdo *O*-glycosidation of 18 with a series of acceptors.

Adding (*p*-Tol)_2_SO as the additive in photocatalytic Kdo *O*-glycosidation could change the stereoselectivity from β-to α-anomer. Under the optimized reaction conditions (1.0 equiv. of 18, 1.5 equiv. of acceptor, 1.5 equiv. of Umemoto's reagent, 0.04 equiv. of Ir[dF(CF_3_)ppy]_2_(dtbbpy)PF_6_, 1.5 equiv. of Cu(OTf)_2_, and 3.4 equiv. of (*p*-Tol)_2_SO, −30 °C 12 h, in MeCN/CH_2_Cl_2_ = 2/1), the switchable phenomenon of stereoselectivity was explored using different types of acceptors. As shown in Scheme 3, using primary non-carbohydrate alcohols (1-octanol S1 or benzyl alcohol S9) as acceptors could not achieve such goals (36a and 36i, *α*/*β* ≈ 1 : 1), because of their strong nucleophilic properties and less steric hindrance which lead to the competition between the attack of primary alcohols and the attack of (*p*-Tol)_2_SO towards the glycosyl nitrilium ions. Surprisingly, for other non-primary alcohols (S11–S12, S15, and S21–28) and primary sugar alcohols (S16–S20), based on their weaker nucleophilic properties and larger steric hindrance, the switchable phenomenon of stereoselectivity was successfully achieved (36k, 36l, 36o, 36p–36t, 36v–36ac, *α*/*β* > 3 : 1) in good yields (57–99%). The single crystal structures of 36pα and 36tα were obtained to further determine the absolute stereochemical configurations. When using Kdo glycosyl acetate S24 as the acceptor, the desired α-Kdo-(2 → 4)-α-Kdo-OAc disaccharide 36yα, which is the Kdo disaccharide unit of Kdo_2_-lipid A, could be obtained in a good yield (64%) with high α-stereoselectivity.

Besides, scale-up reactions were conducted to evaluate the pragmatic value of the present method and designed apparatus (Table 2). It was shown that such a reaction could efficiently proceed at the milligram scale level. However, at the gram scale level, limited by the size of the photoreactor, the activation of donors was 60% resulting in moderate yields (59%) while the configuration remained β-selective.

**Table 2:** Scale-up reaction

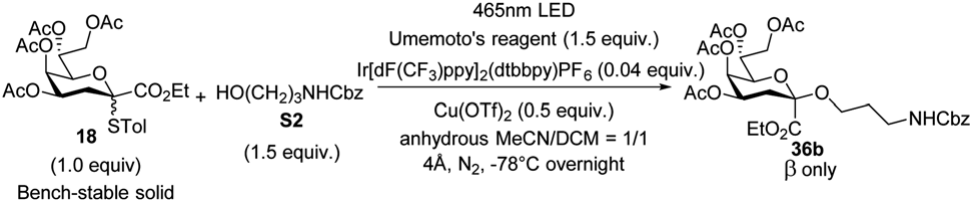			
Entry	Amount of donor	Concentration	Yield^a^
1	32.4 mg	0.033 M	94%
2	324.3 mg	0.17 M	88%
3	1.04 g	0.19 M	59%^40%^

a Isolated yields and the superscripts indicating the amount of donor regained.

### Mechanism study

#### Activation of the donor

Based on the above experimental results, the mechanism of the photocatalytic Kdo *O*-glycosidation reaction could be divided into two parts: the activation of the donor to form the active intermediate and the reaction between the intermediate and acceptor to give the final product.

**Scheme 4:**
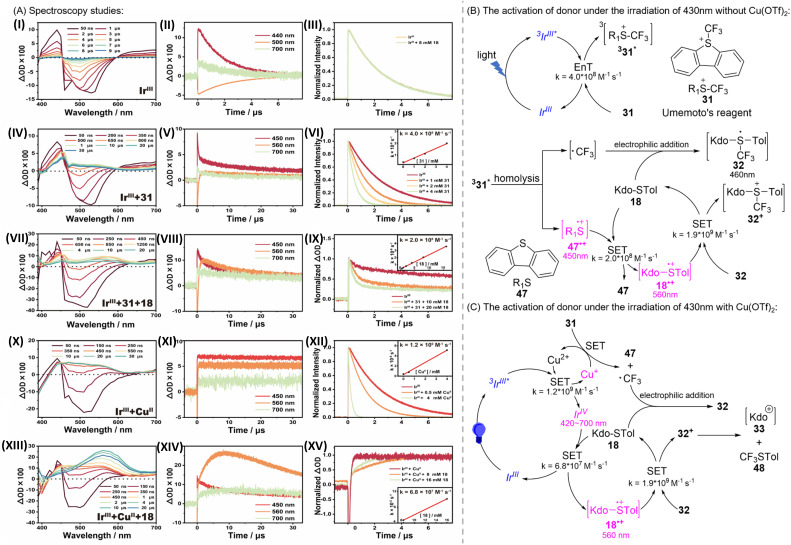
(A) Spectroscopy studies: (I) transient absorption spectra of ^3^Ir^III*^; (II) kinetics curves for transient absorption at 440, 500, and 700 nm of ^3^Ir^III*^; (III) normalized transient emission kinetics of ^3^Ir^III*^ at 500 nm in the absence and presence of 18; (IV) transient absorption spectra of ^3^Ir^III*^ + 31; (V) kinetics curves for transient absorption at 450, 500, and 700 nm of ^3^Ir^III*^ + 31; (VI) normalized transient emission kinetics of ^3^Ir^III*^ at 500 nm with different concentrations of 18; inset: Stern–Volmer plot obtained from the decay of ^3^Ir^III*^ with different concentrations of 18; (VII) transient absorption spectra of ^3^Ir^III*^ + 31 + 18; (VIII) kinetics curves for transient absorption at 450, 560, and 700 nm of ^3^Ir^III*^ + 31 + 18; (IX) normalized kinetics curves for transient absorption at 450, 560, and 700 nm of ^3^Ir^III*^ + 31 + 18; inset: Stern–Volmer plot obtained from the decay of ^3^Ir^III*^ + 31 at 450 nm with different concentrations of 18. (X) Transient absorption spectra of ^3^Ir^III*^ + Cu^II^; (XI) kinetics curves for transient absorption at 450, 560, and 700 nm of ^3^Ir^III*^ + Cu^II^; (XII) normalized transient emission kinetics of ^3^Ir^III*^ at 500 nm with different concentrations of Cu^II^; inset: Stern–Volmer plot obtained from the decay of ^3^Ir^III*^ with different concentrations of Cu^II^; (XIII) transient absorption spectra of ^3^Ir^III*^ + Cu^II^ + 18; (XIV) kinetics curves for transient absorption at 450, 560, and 700 nm of ^3^Ir^III*^ + Cu^II^ + 18; (XV) normalized kinetics curves for transient absorption for ^3^Ir^III*^ + Cu^II^ at 560 nm with different concentrations of 18; inset: Stern–Volmer plot obtained from the decay of ^3^Ir^III*^ + Cu^II^ with different concentrations of 18. Experimental conditions (unless otherwise stated): under deoxygenated conditions in MeCN solution at 430 nm. The concentrations of Ir^III^, Cu^II^, 31, and 18 used are 400 μM, 8 mM, 8 mM, and 8 mM, respectively. (B) Proposed mechanism of the activation of glycosidation under 430 nm irradiation without Cu(OTf)_2_. (C) Proposed mechanism of the activation of glycosidation under 430 nm irradiation with Cu(OTf)_2_.

Considering that the activation of the donor is not affected by temperature (Table S2†), which only influences the subsequent attack on 34α and 35β (Scheme 1c) by the acceptor, to elucidate the underlying mechanism by which the photocatalyst Ir^III^ activates substrate 31/18, we recorded the nanosecond transient absorption (ns-TA) spectra at room temperature for Ir^III^ alone and in a mixture with 31/18 under 430 nm laser excitation. As depicted in Scheme 4Ai–ii, upon laser excitation of Ir^III^ under N_2_-saturated conditions, both the negative signals (negative bands at around 500 nm) and positive signals (a positive peak at around 440 and a featureless, flat absorption from 600 to 700 nm) are observed initially. The decay kinetics at 440, 500, and 700 nm all exhibit mono-exponential kinetics ([OD] = [OD]_0_ e^−*k*/*t*^) with a consistent lifetime of 2.5 μs, suggesting the presence of a single transient species. This species is attributed to the triplet state (^3^Ir^III*^) based on the strong spin–orbit coupling effect (*ξ*_Ir_ = 3909 M^−1^ cm^−1^), which facilitates rapid intersystem crossing from singlet to triplet states (<100 fs), and the sensitivity of the transient signal to oxygen (Fig. S3a†).^36–38^ Therefore, the obtained lifetime of 2.5 μs corresponds to the photophysical decay process of ^3^Ir^III*^ to its ground state.

In the presence of 18, the decay of ^3^Ir^III*^ is not affected (Scheme 4Aiii and S3b†). In contrast, for Ir^III^ in the presence of excess 31, as shown in Scheme 4Aiv–vi, the decay of ^3^Ir^III*^ is significantly accelerated. This is consistent with the steady-state luminescence experiments: as more 31 is added, the luminescence intensity of ^3^Ir^III*^ decreases continuously (Fig. S3c†). Linear fitting of the measured pseudo-first-order reaction rate constants *versus*31 concentration allows for the direct determination of the quenching efficiency of ^3^Ir^III*^ by using 31 (4.0 × 10^8^ M^−1^ s^−1^) (Scheme 4Avi). These findings suggest that the entire photocatalytic cycle is initiated by the quenching of ^3^Ir^III*^ by 31.

The efficient reaction of ^3^Ir^III*^ + 31 is accompanied by the emergence of a new spectral shape within 1.5 μs, characterized by an absorption peak at around 450 nm and a broad band at around 540 nm (Scheme 4Aiv). According to Wu *et al.*'s work,^39^ this quenching reaction proceeds through a Dexter energy transfer catalytic mechanism: ^3^Ir^III*^ transfers energy to 31, resulting in Ir^III^ and 31 in the triplet state (^3^31^*^). The generated triplet of 31 undergoes C–S σ-bond homo-cleavage, yielding ˙CF_3_ and R_1_S˙^+^. In the illuminated Ir^III^ + 31 system, the direct observation of both ˙CF_3_ and R_1_S˙^+^ through ESR spectroscopy supports the above mechanism (Fig. S4†). Interestingly, such a new spectral shape is also observed when 31 is directly excited (Fig. S3d†). Upon direct light absorption, the substrate is expected to follow this pathway: excitation of 31 to the singlet excited state, followed by intersystem crossing (ISC) to ^3^31^*^, which induces homolytic cleavage. This control experiment provides additional evidence supporting the Dexter energy transfer mechanism mainly responsible for this quenching reaction.

The possible assignment of this new transient spectral shape as ^3^31^*^ or its subsequent homo-cleavage species (˙CF_3_ and/or R_1_S˙^+^), was further assessed. First, the decay of these new transient signals is independent of the oxygen concentration, which excludes the ascription as ^3^31^*^ (Fig. S3e†). Indeed, C–S σ-bond homo-cleavage of ^3^31^*^ should be very fast, resulting in the immediate conversion of ^3^31^*^ into homolytically cleaved radical species (˙CF_3_ and R_1_S˙^+^), preventing the detectable accumulation of ^3^31^*^. Second, the ˙CF_3_ radical is expected to absorb primarily in the UV region, as supported by our TD-DFT calculations (Fig. S5a†). In contrast, the calculated spectrum of R_1_S˙^+^ is in good agreement with this new spectral shape (Fig. S5b†). These results suggest that the observed spectrum should be assigned to the R_1_S˙^+^, rather than ^3^31^*^ or ˙CF_3_. The observation of R_1_S˙^+^ supports that Dexter energy transfer is the dominant pathway, rather than the electron transfer from ^3^Ir^III*^ to 31. The latter would result in 47 (ref. 40) (Scheme 4B) and ˙CF_3_ (Fig. S5a†), neither of which have a characteristic signal above 400 nm. Nevertheless, we cannot completely rule out the contribution of the electron transfer mechanism from ^3^Ir^III*^ to 31, based on the following points: (1) the oxidation potential of ^3^Ir^III*^ (−0.65 V (ref. 41)) and the reduction potential of 31 (−0.06 V (ref. 42)). The potential difference indicates that electron transfer is thermodynamically feasible (Table S6†). (2) The new spectral shape obtained from the efficient reaction of ^3^Ir^III*^ + 31 differs slightly from the spectrum after photolysis of 31 alone (Fig. S3f†). This difference may result from the contribution of Ir^IV^ produced by the electron transfer from ^3^Ir^III*^ to 31.

After elucidating that the photocatalytic cycle begins with Dexter energy transfer from ^3^Ir^III*^ to 25, leading to ^3^31^*^ and its conversion into radicals (˙CF_3_ and R_1_S˙^+^), and subsequently identifying R_1_S˙^+^ as the observed spectral species, we measured the transient absorption spectra and kinetics for ^3^Ir^III*^ + 31 + 18. As shown in Scheme 4Avii–ix, after introducing excess 18, the decay of the transient absorption at around 450 nm for R_1_S˙^+^ was significantly accelerated, with a concomitant build-up of a new broad band at around 560 nm. Linear fitting of the measured pseudo-first-order reaction rate constants *versus*18 concentration yields the second-order rate constant for the reaction R_1_S˙^+^ + 18 (2.0 × 10^8^ M^−1^ s^−1^) (Scheme 4Aix). These results demonstrate that 18 can efficiently quench R_1_S˙^+^. Our DFT calculations on the standard free energy change indicate that the electron transfer from 18 to R_1_S˙^+^ is thermodynamically favorable (Table S7†). Moreover, TD-DFT calculations show that the calculated spectrum of 18^·+^ matches well with the spectrum shape at around 560 nm, suggesting the assignment of this new spectral feature as 18^·+^ (Fig. S5c†). The appearance of 18^·+^ from the reaction R_1_S˙^+^ + 18 provides direct evidence for the electron transfer from 18 to R_1_S˙^+^.

This transient signal at around 560 nm for 18^·+^ decays on a much longer timescale. By analyzing the decay kinetics at 560 nm, we found that instead of first-order kinetics (mono exponential decay behavior), the decay of 18^·+^ can be approximated by using second-order reaction behavior (Fig. S3g†). This suggests that the decay of 18^·+^ likely results from reactions with species of comparable concentration in the system. These species include ˙CF_3_ or its subsequent transformations. Considering that ˙CF_3_ is electrophilic, it can effectively undergo electrophilic addition reactions with thioglycosides (18) containing lone pairs on sulfur atoms, leading to rapid quenching and the formation of 32. Therefore, we propose that 32, rather than the ˙CF_3_ radical, is more likely responsible for quenching 18^·+^. The quenching process of 18^·+^ by 32 is expected to most likely be through the electron transfer pathway, leading to the re-generation of 18 and formation of 32^+^. The structure of 32^+^ is quite unstable and decomposes into species 33 and 48*via* C–S band cleavage. This heterolytic dissociation process is supported by our DFT calculations, which predict a barrier-free reaction potential for the conversion of 32^+^ into 33 and 48 (Fig. S6†). Species 33 can further transform into the target product. Experimental observations of species 48 (ref. 20) (Scheme 4C) and the target product also support the above reaction pathway. Based on the calculated extinction coefficients for 32 and 18^·+^ at 560 nm (31 and 7176 L M^−1^ cm^−1^, respectively), we determined the second-order reaction rate constant for 32 + 18^·+^ through kinetic fitting of the decay at 560 nm (1.9 × 10^9^ M^−1^ s^−1^) (Fig. S3g, S5c and d†).

The results of the steady-state experiments indicate that the introduction of Cu^II^ enhances the efficiency and yield of the reaction. To elucidate its role, we further examined the impact of Cu^II^ on the underlying reaction mechanism and kinetics of the photocatalytic cycle. As shown in Scheme 4Ax–xii, it was found that Cu^II^ could efficiently quench ^3^Ir^III*^, with the quenching being linearly dependent on the concentration of Cu^II^ (1.2 × 10^9^ M^−1^ s^−1^). After ^3^Ir^III*^ is quenched by Cu^II^, a new transient spectrum is observed (Scheme 4Ax). This includes a negative signal at around 400 nm and positive signals from 420 to 700 nm, with a broad band at around 490 nm, together with a shoulder at around 560 nm. The decay of these new spectral signals is not affected by oxygen (Fig. S3h†). Therefore, rather than being triplet species generated from the potential Dexter energy transfer from ^3^Ir^III*^ to Cu^II^, these new spectral signals are expected to result from electron transfer between ^3^Ir^III*^ and Cu^II^. Given that the new spectral signals do not display the spectral features of Ir^II^,^43–45^ characterized by two resolved absorption bands at around 490 and 525 nm, we propose that, rather than a reductive quenching, the reaction between ^3^Ir^III*^ and Cu^II^ is likely an oxidative quenching, resulting in Ir^IV^ and Cu^I^. Besides, the calculated negative Δ*G* also suggests that electron transfer from ^3^Ir^III*^ to Cu^II^ is thermodynamically favorable, based on the oxidation potential of ^3^Ir^III*^ (−0.65 V (ref. 41)) and the reduction potential of Cu^II^ (+1.21 V (ref. 46)). The generated species, Ir^IV^ and Cu^I^, can further complete the catalytic cycle through electron acceptance and donation, respectively. Subsequently, we explored the possible quenching species for Ir^IV^ and Cu^I^, separately. In the ^3^Ir^III*^ + Cu^II^ system, the addition of 18 also resulted in the efficient generation of 18^·+^, characterized by the emergence of a band at around 560 nm (Scheme 4Axii–xiv). The formation of 18^·+^ increased with the increasing concentration of 18, with a rate constant of 6.8 × 10^7^ M^−1^ s^−1^ (Scheme 4Axv). This observation demonstrates that the substrate 18 can be oxidized by the sole oxidizing species in solution, Ir^IV^, through a bimolecular electron transfer pathway, thus completing the Ir^IV^/Ir^III^ cycle. In contrast, previous reports suggested that the electron-donating species, Cu^I^, can transfer an electron to the substrate 31, which upon receiving the electron, generates dibenzothiophene (47, Scheme 4B), ˙CF_3_, and Cu^II^, thus achieving Cu^I^/Cu^II^ cycling.^47,48^

**Fig. 3:**
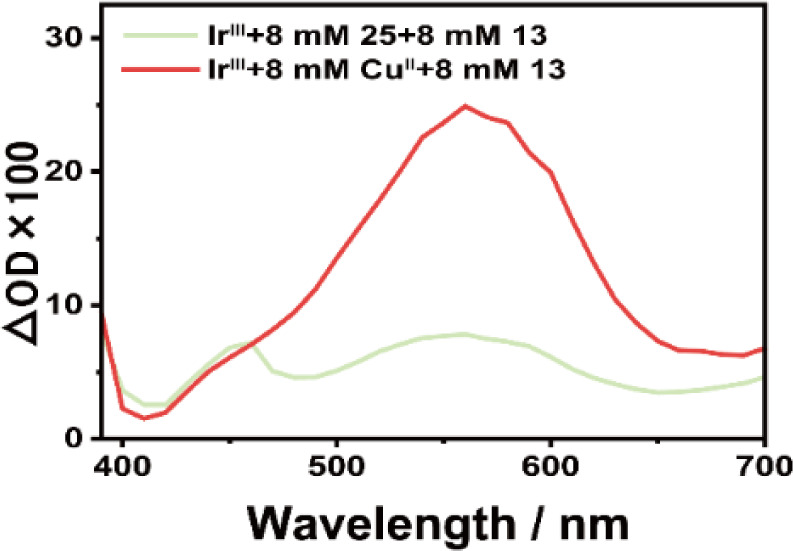
Transient absorption spectrum obtained at 10 μs for ^3^Ir^III*^ + 31 + 18, in comparison with that for ^3^Ir^III*^ + Cu^II^ + 18. Experimental conditions (unless otherwise stated): under deoxygenated conditions in MeCN solution at 430 nm. The concentrations of Ir^III^, 31, 18, and Cu^II^ used are 400 μM, 8 mM, 8 mM, and 8 mM, respectively. Note: the absorption peak at around 460 nm could be assigned to 32 supported by the calculated spectrum of 32 shown in Fig. S5d.†

Interestingly, we observed that the transient signals of 18^·+^ generated in the ^3^Ir^III*^ + Cu^II^ + 18 reaction are notably stronger than in the ^3^Ir^III*^ + 31 + 18 system (Fig. 3). In both systems, ^3^Ir^III*^ is effectively quenched by Cu^II^ or 31. This indicates that the generation of 18^·+^ through Ir^IV^/Ir^III^ and Cu^I^/Cu^II^ cycles is more effective compared to the Dexter energy transfer from ^3^Ir^III*^ to 31 followed by oxidation of 18 by R_1_S˙^+^. The increased generation of 18^·+^ is expected to result in more products, explaining why the addition of Cu^II^ enhances the efficiency of the entire photocatalytic reaction.

Besides, the much higher second-order rate constant for Cu^II^-quenched ^3^Ir^III*^ (1.2 × 10^9^ M^−1^ s^−1^), compared with 31-quenched ^3^Ir^III*^ (4.0 × 10^8^ M^−1^ s^−1^), demonstrates the superior photon utilization efficiency of the electron transfer quenching mechanism to that of its energy transfer counterpart. Incorporating Cu^II^ preserves the energy transfer pathway of ^3^Ir^III*^ quenching while introducing an additional electron transfer pathway. This dual-pathway mechanism enhances overall ^3^Ir^III*^ quenching efficiency, thereby markedly improving total photon utilization. This enhanced photon utilization, facilitated by the Cu^II^-mediated electron transfer mechanism, also leads to increased generation of 18^·+^ radicals, which would result in more products.

### Stereoselectivity of glycosidation

**Scheme 5:**
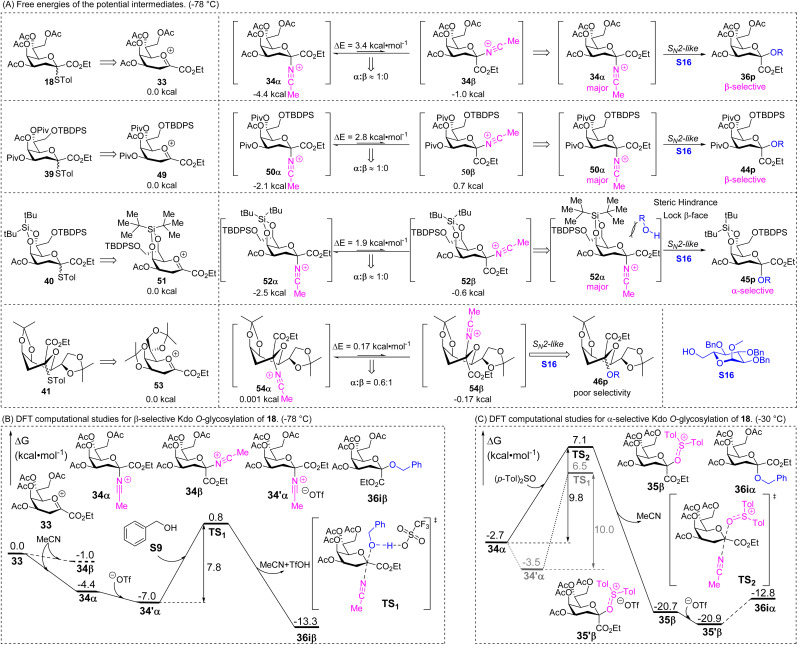
DFT calculation studies determined at B3LYP-D3(BJ)/6-311+G(d, p) in the MeCN/DCM = 1 : 1 mixed solvent system (SMD).^32^ (A) Free energies of potential intermediates at −78 °C. (B) Free energy profile of the formation of 36iβ with benzyl alcohol S9 as the acceptor at −78 °C. (C) Free energy profile of the formation of 36iα with (*p*-Tol)_2_SO as the additive at −30 °C.

Based on the experimental results shown in Tables 1, S3–S4† and Scheme 2 & 3 the selectivity of photocatalytic Kdo *O*-glycosidation was highly controlled by acetonitrile and (*p*-Tol)_2_SO. After forming the intermediate oxacarbenium ion 33, it would be stabilized by MeCN to generate the less active intermediate glycosyl nitrilium ions 34 immediately at −78 °C (Scheme 5A). Based on the DFT method, benzyl alcohol S9 was selected to further explore the mechanism from intermediates 33 (Scheme 5B). Due to the evident free energy difference (3.4 kcal mol^−1^) between 34α and 34β, the reaction would proceed through 34α (99.98%), according to the Boltzmann distribution. The intermediate is further combined with the trifluoromethanesulfonic anion to generate more stable species 34′α. Then nucleophile S9 subsequently approaches the anomeric carbon from the β-face *via* a S_N_2-like transition state TS1 to form MeCN, TfOH and product 36iβ, accompanied by a hydrogen transfer from the hydroxyl group of benzyl alcohol to the TfO^−^ ion. The free energy barrier of this step is 7.8 kcal mol^−1^.

For other Kdo glycosyl donors with a chair conformation (^4^C_1_) and electron-withdrawing groups, such as 39 (Scheme S1†), the free energy differences of the glycosyl nitrilium ions (50α/50β) are similar to that of 34α/34β, which results in β-stereoselectivity (Scheme 5A). The bulky protecting group in donor 40 acts as a more significant factor, restricting the reaction space on the donor's β-face and resulting in α-stereoselectivity. When using 41 as the donor, the twist-boat conformation of 41 results in the different energy differences of 54α/54β, thereby reducing the reaction selectivity (Scheme 5A).

When adding additive (*p*-Tol)_2_SO in the glycosidation at −30 °C, the glycosyl nitrilium ion 34α with a free energy of −2.7 kcal mol^−1^ is first attacked by (*p*-Tol)_2_SO from the β-face to generate the more stable intermediate 35β with a free energy of −20.7 kcal mol^−1^ through the transition state TS2 (Scheme 5C). Then 35β is stabilized by the TfO^−^ ion to generate 35′β with a free energy of −20.9 kcal mol^−1^ and further reacts with the nucleophile S9*via* the S_N_2-like transition state TS3 to produce the product 36iα, which is reported in our previous work.^14^ Since the acceptor S9 is a primary alcohol with strong nucleophilic properties and low steric hindrance, for the rate-limiting step 34α → TS2 (9.8 kcal mol^−1^), compared with the free energy barrier of the direct attack of 34′α by S9 to obtain product 36iβ (10.0 kcal mol^−1^), the tiny difference between them makes the stereoselectivity poor (*α*/*β* = 0.9 : 1). But for other acceptors with less nucleophilic properties and larger steric hindrance, the energy difference might be obvious and the direct attack of 34α by acceptors might be harder, leading to higher α-selectivity.

On the basis of the mechanism study above, two plausible and substantiated catalytic cycles were drawn as shown in Scheme 4B and C. Ir^III^ is first excited to reach the triplet state ^3^Ir^III*^. Through the single electron transfer (SET) process, Cu^II^ is reduced into Cu^I^ by ^3^Ir^III*^, followed by the oxidation of Cu^I^ with Umemoto's reagent to generate 47 and ˙CF_3_. The electrophile ˙CF_3_ immediately binds with the excess thioglycoside donor 18 to produce intermediate 32 (Scheme 4C). Meanwhile, the donor 18 is oxidized into 18^·+^ by Ir^IV^. Through the SET reaction between 32 and 18^·+^, 32^+^ is generated and immediately decomposes into species 48 and 33. Notably, it is necessary to consider the EnT process at the activation stage to form 33 (Scheme 4B). The intermediate 33 is further stabilized by MeCN or (*p*-Tol)_2_SO to form 34′α or 35′β. Finally, the nucleophile ROH attacks the intermediate 34′α or 35′β*via* a S_N_2-like mechanism to produce β- or α-Kdo *O*-glycosides (Scheme 5B and C).

## Conclusions

In summary, a photocatalytic stereoselective Kdo *O*-glycosidation was developed using 4,5,7,8-tetra-*O*-acetyl-protected Kdo *p*-toluenethioglycoside 18 as the donor. Based on such a strategy, the α- and β-Kdo *O*-glycosides could be switchably synthesized by adding (*p*-Tol)_2_SO or not. With the special photoreactor improved by us, from the primary alcohol acceptors and several secondary alcohols, β-Kdo *O*-glycosides could be obtained efficiently for the complex saccharide synthesis. The detailed mechanism studies first showed that both EnT and SET processed could be preserved at the initiation stage and copper(ii) was the key catalyst in the SET process. Further DFT calculation indicated that the photocatalytic Kdo *O*-glycosidation proceeds *via* the S_N_2-like mechanism (Scheme 1d). This work pioneered a photocatalytic glycosidation method for Kdo *O*-glycosidation and provided a practical MeCN/(*p*-Tol)_2_SO dual mediated strategy for α-/β-switchable stereoselective glycosidation. The detailed mechanism revealed by laser flash photolysis and steady-state spectral measurement offered a novel framework for understanding the activation processes in photocatalytic glycosidation reactions.

## Author contributions

Conceptualization, J.-d. Zhang and J.-l. Jie; methodology, J.-d. Zhang and J.-l. Jie; validation, J.-d. Zhang and S.-y. Yan; formal analysis, J.-d. Zhang, J.-l. Jie and S.-y. Yan; investigation, J.-d. Zhang, J.-l. Jie, S.-y. Yan, H. Zhang, J.-m. Chen, J.-c. Wu, L.-y. Qin and G.-j. Liu; visualization, J.-d. Zhang and S.-y. Yan; writing – original draft, J.-d. Zhang and J.-l. Jie; writing – review & editing, J.-d. Zhang, J.-l. Jie, H.-m. Su and G.-w. Xing; funding acquisition, J.-l. Jie, H.-m. Su and G.-w. Xing; resources, J.-l. Jie, H.-m. Su and G.-w. Xing; supervision, J.-l. Jie, H.-m. Su and G.-w. Xing.

## Conflicts of interest

There are no conflicts to declare.

## Supplementary Material

SC-OLF-D5SC02980E-s001

SC-OLF-D5SC02980E-s002

## Data Availability

The data supporting this article have been included as part of the ESI.†

## References

[cit1] Unger F. M. (1981). Adv. Carbohydr. Chem. Biochem..

[cit2] Cipolla L., Gabrielli L., Bini D., Russo L., Shaikh N. (2010). Nat. Prod. Rep..

[cit3] Wilkinson S. G. (1996). Prog. Lipid Res..

[cit4] Schmidt M. A., Jann K. (1983). Eur. J. Biochem..

[cit5] Laroussarie A., Barycza B., Andriamboavonjy H., Tamigney Kenfack M., Blériot Y., Gauthier C. (2015). J. Org. Chem..

[cit6] Ovchinnikova O. G., Mallette E., Koizumi A., Lowary T. L., Kimber M. S., Whitfield C. (2016). Proc. Natl. Acad. Sci. U. S. A..

[cit7] Hansson J., Oscarson S. (2000). Curr. Org. Chem..

[cit8] Oscarson S. (2012). Carbohydr. Chem..

[cit9] Imoto M., Kusunose N., Matsuura Y., Kusumoto S., Shiba T. (1987). Tetrahedron Lett..

[cit10] Lou Q., Hua Q., Zhang L., Yang Y. (2020). Org. Lett..

[cit11] Miao H., Lu S., Chen H., Shang J., Zheng J., Yang Y. (2024). Org. Biomol. Chem..

[cit12] Mi X., Lou Q., Fan W., Zhuang L., Yang Y. (2017). Carbohydr. Res..

[cit13] Miao H., Yu R., Zheng J., Shang J., Zhang L., Ma M., Yang Y. (2024). Org. Lett..

[cit14] Zhang J., Gao X., Liu S., Geng Z., Chang L., Liu Y., Ma Q., Xing G., Liu G., Fang D. (2023). Org. Lett..

[cit15] Hoffmann N. (2008). Chem. Rev..

[cit16] Narayanam J. M. R., Stephenson C. R. J. (2011). Chem. Soc. Rev..

[cit17] Kumar G. S., Lin Q. (2021). Chem. Rev..

[cit18] Wang H., Wu P., Zhao X., Zeng J., Wan Q. (2019). Acta Chim. Sin..

[cit19] Zhang J., Luo Z., Wu X., Gao C., Wang P., Chai J., Liu M., Ye X., Xiong D. (2023). Nat. Commun..

[cit20] Mao R., Xiong D., Guo F., Li Q., Duan J., Ye X. (2016). Org. Chem. Front..

[cit21] Yu Y., Xiong D., Mao R., Ye X. (2016). J. Org. Chem..

[cit22] Spell M. L., Deveaux K., Bresnahan C. G., Bernard B. L., Sheffield W., Kumar R., Ragains J. R. (2016). Angew. Chem., Int. Ed..

[cit23] Krumb M., Lucas T., Opatz T. (2019). Eur. J. Org Chem..

[cit24] Valenzuela E. A., Duong T., Pradeep V., He X., Dobson J. M., Lee S., Lopata K., Ragains J. R. (2025). Angew. Chem., Int. Ed..

[cit25] Dang Q., Deng Y., Sun T., Zhang Y., Li J., Zhang X., Wu Y., Niu D. (2024). Nature.

[cit26] Zhang C., Zuo H., Lee G. Y., Zou Y., Dang Q., Houk K. N., Niu D. (2022). Nat. Chem..

[cit27] Li L., Yin X., Jiang Y., Xia Y., Wang X., Li J., Li H., Qin Y., Yang J. (2024). Org. Lett..

[cit28] Ngoje P., Crich D. (2020). J. Am. Chem. Soc..

[cit29] Zhang J., Liu G., Xing G. (2025). Chem. Commun..

[cit30] Hori H., Nakajima T., Nishida Y., Ohrui H., Meguro H. (1988). Tetrahedron Lett..

[cit31] Unger F. M., Stix D., Schulz G. (1980). Carbohydr. Res..

[cit32] Andreana P. R., Crich D. (2021). ACS Cent. Sci..

[cit33] Lu T., Chen Q. (2021). Comput. Theor. Chem..

[cit34] Crich D., Li W. (2006). Org. Lett..

[cit35] Liu G., Li C., Zhang X., Du W., Gu Z., Xing G. (2018). Chin. Chem. Lett..

[cit36] Flamigni L. , Barbieri A., Sabatini C., Ventura B. and Barigelletti F., Photochemistry and photophysics of coordination compounds: iridium, Springer, 2007

[cit37] Hedley G. J., Ruseckas A., Samuel I. D. W. (2010). J. Phys. Chem. A.

[cit38] Tschierlei S., Neubauer A., Rockstroh N., Karnahl M., Schwarzbach P., Junge H., Beller M., Lochbrunner S. (2016). Phys. Chem. Chem. Phys..

[cit39] Liu T., Li T., Tea Z. Y., Wang C., Shen T., Lei Z., Chen X., Zhang W., Wu J. (2024). Nat. Chem..

[cit40] Jepsen T. H., Larsen M., Jørgensen M., Solanko K. A., Bond A. D., Kadziola A., Nielsen M. B. (2011). Eur. J. Org Chem..

[cit41] Koike T., Akita M. (2014). Inorg. Chem. Front..

[cit42] Yasu Y., Koike T., Akita M. (2012). Angew. Chem., Int. Ed..

[cit43] Lin Z., Zhou Q., Liu Y., Chen C., Jie J., Su H. (2024). Chem. Sci..

[cit44] Qin Y., Sun R., Gianoulis N. P., Nocera D. G. (2021). J. Am. Chem. Soc..

[cit45] Sun R., Qin Y., Ruccolo S., Schnedermann C., Costentin C., Nocera D. G. (2019). J. Am. Chem. Soc..

[cit46] Johnsson M., Persson I. (1987). Inorg. Chim. Acta.

[cit47] Barata-Vallejo S., Lantaño B., Postigo A. (2014). Chem.–Eur. J..

[cit48] Zhang C. (2014). Org. Biomol. Chem..

