# Supplementary material for: Single electron/energy transfer photocatalysis: α-/β-switchable synthesis of 3-deoxy-d-manno-oct-2-ulosonic acid O-glycosides
Source: Chem Sci. 2025 Jun 13;16(28):13070–81. doi: 10.1039/d5sc02980e (PMC12179690; doi:10.1039/d5sc02980e)
Supplement: SC-016-D5SC02980E-s001 [file SC-016-D5SC02980E-s001.pdf]

Supplementary Information (SI) for Chemical Science.
This journal is © The Royal Society of Chemistry 2025

Supporting Information

Single Electron/Energy Transfer Photocatalysis: α-/β- Switchable Synthesis of 3-Deoxy-D-manno-oct-2-ulosonic Acid *O*-Glycosides

Jing-dong Zhang,[‡a] Jia-long Jie,[‡*a] Shu-yi Yan,[a] Hui Zhang,[a] Jia-meng Chen,[a] Jiang-cheng Wu,[a] Lu-yang Qin,[a] Guang-jian Liu,[a] Hong-mei Su[a] and Guo-wen Xing*[a]

[a] College of Chemistry, Beijing Normal University, Beijing 100875, P. R. China.

E-mail: gwxing@bnu.edu.cn, jialong@bnu.edu.cn

‡ J.-d. Zhang and J.-l. Jie contributed equally to this work.

Contents of Supporting Information

1 Supplementary tables and figures. 1

2 Materials and instruments 9

3 Experimental procedures 10

3.1 Preparation of donors 10

3.2 Preparation of acceptors 17

3.3 General procedure (GP) for glycosidation reactions 22

4 X-ray Crystallography 53

4.1 Crystallographic Information of Ethyl (4,5,7,8-tetra-*O*-acetyl-deoxy-α-D-manno-oct-2-ulopyranosyl)onate-(2→6)-methyl 2,3,4-tri-*O*-benzyl-α-D-glucopyranoside (CCDC:2382908) : 54

4.2 Crystallographic Information of Ethyl (4,5,7,8-tetra-*O*-acetyl-3-deoxy-α-D-manno-oct-2-ulopyranosyl)onate-(2→1)-Diacetone-β-D-fructopyranose (CCDC:2382909) : 55

5 Computational methods and data 56

6 NMR spectra of compounds 109

7 Reference 166

1 Supplementary tables and figures.

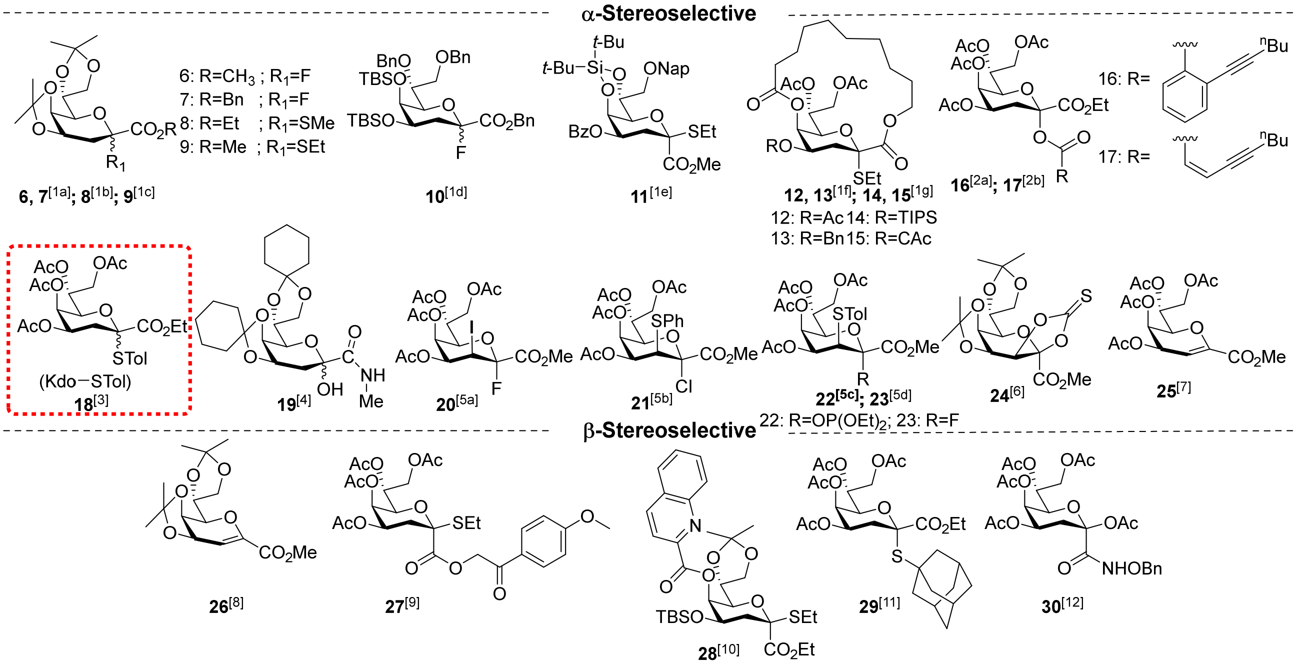

Figure S1. Previous work on Kdo glycosidation by the ionic activation.

As shown in Figure S1, various strategies (**6-25**) have been developed for the efficient synthesis of α-Kdo glycosides, including using bulky protecting group to limit the β-face attack of receptors with strong steric hin-drance,[1] adding exogenous nucleophile such as DMF[2] or (p-Tol)$_2$SO[3] to stabilize oxacarbenium ion, and utilizing solvent and ion effect.[4] Moreover, some indirect methods have been published to better achieve complete α-stereocontrol and less byproduct, such as introducing an auxiliary group (-I,[5a] -SPh[5b] and -STol[5c, 5d]) at C3 position and the conversion from Ko[6] or 2,3-ene.[7] These indirect ways need more additional steps to construct the special donors and the target Kdo glycosides.

Nevertheless, the researches on the stereoselective synthesis of β-Kdo glycosides (**26-30**) are relatively limited. Kdo 2,3-ene, which was initially applied to synthesizing α-Kdo glycosides, was otherwise used to construct the β-Kdo glycosidic linkages with AIBN as activator by Mong's group in 2014.[8] In 2016, Gauthier and co-workers[9] introduced the 4'-methoxyphenacyl group at C1 position as an auxiliary group to generate β-Kdo O-glycosides through an α-spiroPhen intermediate. Yang's group[10] used a Kdo thioglycoside donor with the quinolinecarbonyl group at the C5 position as an auxiliary group to generate β-Kdo glycoside for 1° acceptors through the hydrogen-bond mediation. In 2020, with the effect of side chain configuration and conformation, Crich's group[11] disclosed a novel methodology for the synthesis of β-Kdo O-glycosides. Recently, Saha et al.[12] used α-

acyloxyhydroxamate as the glycosyl donor to generate β-Kdo glycosides in moderate yields by forming an α-lactam-like species which could block the α-face of the anomeric center.

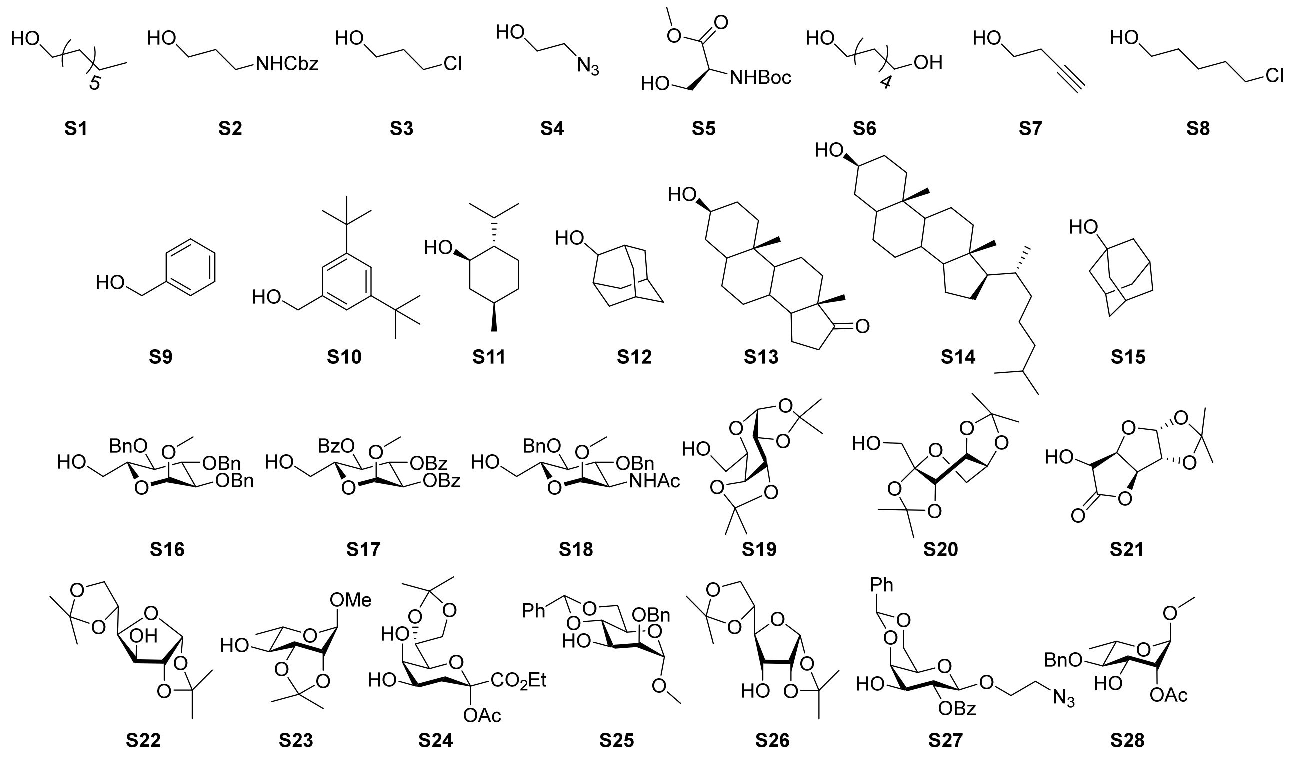

Figure S2. The chemical structures of various glycosyl acceptors **S1-S28**. **S1-S10** are non-carbohydrate alcohols with primary hydroxyl groups, while **S11-S14** are non-carbohydrate alcohols with secondary hydroxyl groups. **S15** is tertiary alcohol. **S16-S20** are sugar acceptors with primary hydroxyl groups, and **S21-S28** are sugar acceptors with secondary hydroxyl groups.

Table S1. Effect of the amount of $Cu(OTf)_2$ on the Photocatalytic Kdo *O*-glycosidation reaction of **18**.

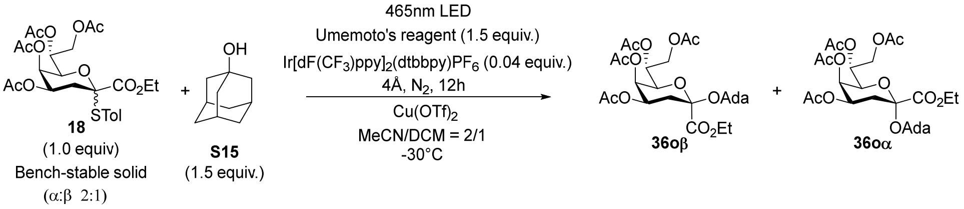

Entry	$Cu(OTf)_2$	Yield[a]	β:α[b]
**1**	1.5 equiv.	70%	1:1.4
**2**	1.3 equiv.	77%	1:1.3
**3**	0.5 equiv.	81%	1:1.3
**4**	0.2 equiv.	$73\%^{8\%}$	1:1.3
**5**	--	$30\%^{63\%}$	1:1.4

[a] Isolated yields and the superscripts indicating the amount of donor regained. [b] Determined by ^1H NMR.

Table S2. Effect of temperature on the Photocatalytic Kdo *O*-glycosidation reaction of **18**.

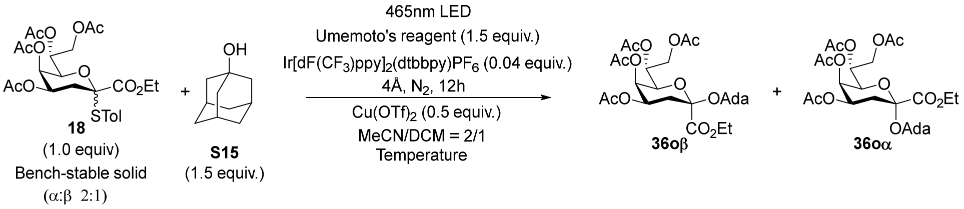

Entry	Temperature	Yield[a]	β:α[b]
**1**	R. T.	46%	1:5.1
**2**	-30 °C	81%	1:1.3
**3**	-50 °C	75%	1.2:1
**4**	-60 °C	81%	1.7:1
**5**[c]	-78 °C	87%	2.8:1

[a] Isolated yields and the superscripts indicating the amount of donor regained. [b] Determined by ^1H NMR. [c] Due to the different freezing point between acetonitrile (-45 °C) and dichloromethane (-95 °C), the reaction needed occur in the DCM/MeCN = 1:1 mixed solvent instead of the DCM/MeCN = 1:2 mixed solvent at -78 °C.

Table S3. Effect of solvent on the Photocatalytic Kdo *O*-glycosidation reaction of **18**.

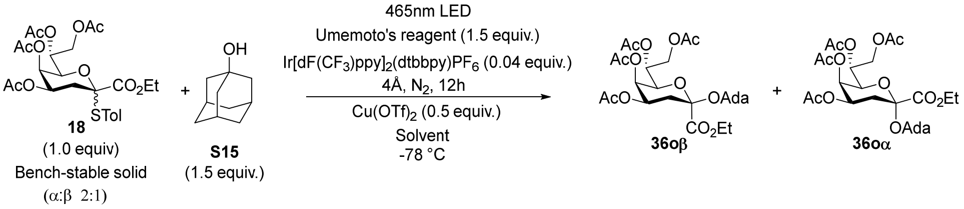

Entry	Solvent<br>MeCN/DCM	Yield[a]	β:α[b]
**1**	1/1	87%	2.8:1
**2**	1/2	68%	2.6:1
**3**	DCM	$49\%^{10\%}$	1.3:1
**4**[c]	2/1	99%	1:13
**5**[c]	MeCN	72%	1:9
**6**[c]	DCM	$15\%^{80\%}$	α only

[a] Isolated yields and the superscripts indicating the amount of donor regained. [b] Determined by ^1H NMR. [c] 1.5 equiv. of $Cu(OTf)_2$ and 3.4 equiv. of $(p\text{-Tol})_2SO$.

Table S4. Effect of the amount of $(p\text{-Tol})_2SO$ on Photocatalytic Kdo *O*-glycosidation reaction of **18**.

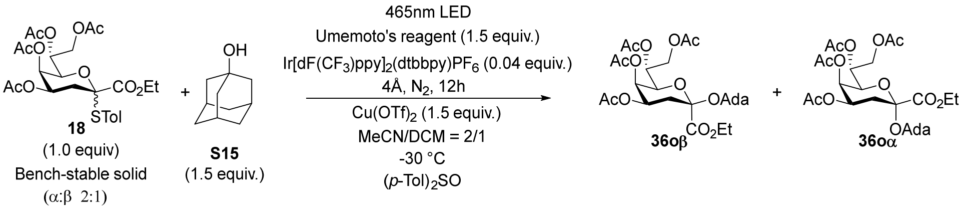

Entry	$(p\text{-Tol})_2SO$	Yield[a]	β:α[b]
**1**[c]	3.4 equiv.	85%	1:20
**2**	4.0 equiv.	56%	1:10
**3**	3.4 equiv.	99%	1:13
**4**	3.0 equiv.	88%	1:9.7
**5**	2.0 equiv.	81%	1:7.1

[a] Isolated yields and the superscripts indicating the amount of donor regained. [b] Determined by ^1H NMR. [c] 0.5 equiv. of $Cu(OTf)_2$.

Table S5. Evaluation of various photocatalysts. Triplet energies (E_T) and potentials ($E_{1/2}$) of photocatalysts are based on literature values. [a, 13-17]

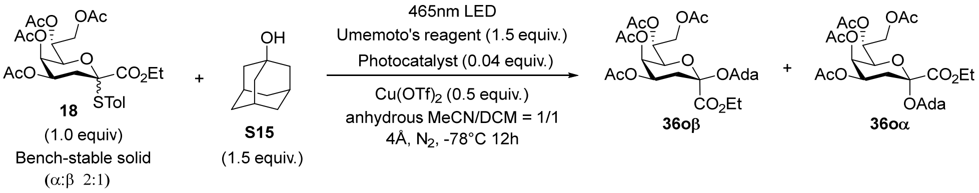

Entry	Photocatalyst	E_T (kcal*mol^{-1})	$E_{1/2}$ PC$^+$/PC* (V)	$E_{1/2}$ PC$^+$/PC (V)	Yield[b] (%)	β:α[c]
**1**	Ir[dF(CF$_3$)ppy]$_2$(dtbbpy)PF$_6$	60.1	-0.89	+1.69	87%	2.8:1
**2**	4CzlPN	58.3	-1.18	+1.49	82%	2.7:1
**3**	Ru(bpy)$_3$(PF$_6$)$_2$	46.5	-0.81	+1.29	79%	2.8:1
**4**	Ir(dtbbpy)(ppy)$_2$PF$_6$	49.2	-0.96	+1.21	31%[67%]	2.7:1
**5**	Eosin Y	45.4	-1.11	+0.78	NR	ND

[a] Potentials versus saturated calomel electrode (SCE). [b] Isolated yields and the superscripts indicating the amount of donor regained. [c] Determined by ^{1}H NMR. NR: not reaction. ND: not determined.

Table S6. Electromotive force [E = $\varphi_+ - \varphi_-$] for the single electron transfer processes.

Entry	Reactions	E (V)
1	$^3\mathbf{Ir^{III*}} + \mathbf{31} \rightarrow \mathbf{Ir^{IV}} + \mathbf{31}^{\cdot -}$	+0.59
2	$^3\mathbf{Ir^{III*}} + \mathbf{Cu^{II}} \rightarrow \mathbf{Ir^{IV}} + \mathbf{Cu^{I}}$	+1.86

[a] The electromotive force was calculated using the Nernst equation [$\Delta G\ (\mathrm{kJ/mol}) = -nFE(V)$].

Table S7. B3LYP-D3(BJ)/6-311+g(d,p) calculated Gibbs Free Energy difference[ΔG = ΣG(product) - ΣG(reactant)] in DCM/MeCN = 1:1 solvent system.

Entry	Reactions	ΔG (kcal/mol)
1	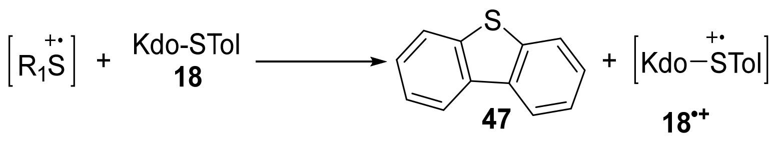	**-1.7** 298.15K
		**-1.7** 243.15K
		**-1.7** 195.15K

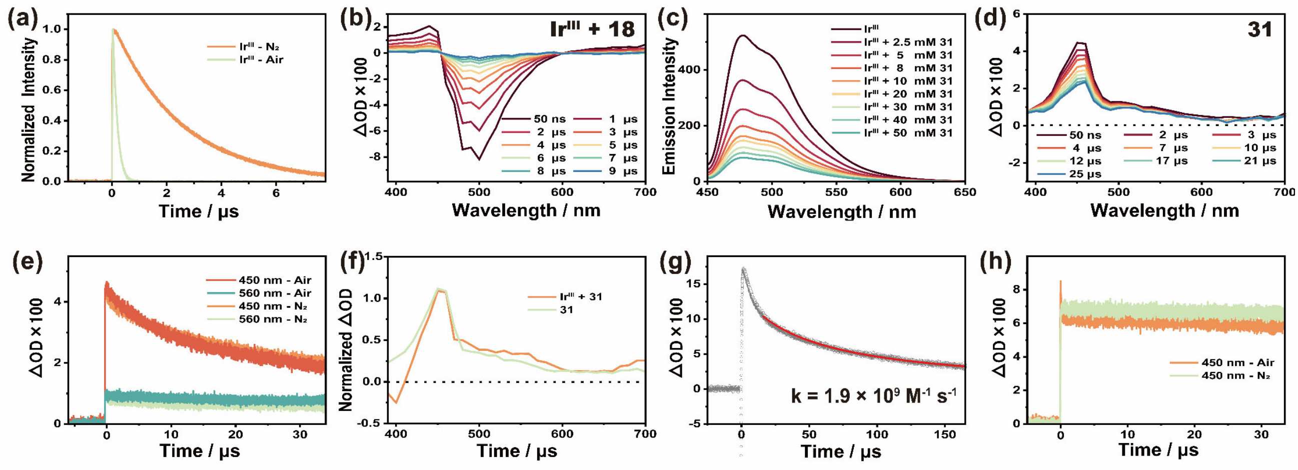

Figure S3. (a) Normalized transient emission kinetics of ^{3}**Ir^{III*}** at 500 nm in deoxygenated conditions and in air conditions; (b) Transient absorption spectra of ^{3}**Ir^{III*}** + **18**; (c) Steady-state emission spectra of ^{3}**Ir^{III*}** at different concentrations of **31**; (d) Transient absorption spectra of **31** (1 mM) under deoxygenated conditions in MeCN solution following 355 nm excitation; (e) Kinetics curves for transient absorption at 450 and 560 nm of **31** (1 mM) under both deoxygenated and air conditions in MeCN solution following 355 nm excitation; (f) Normalized transient absorption spectrum obtained at 10 μs for ^{3}**Ir^{III*}** + **31**, in comparison with that for **31** (1 mM) under deoxygenated conditions in MeCN solution following 355 nm excitation; (g) Kinetics curve for transient absorption at 560 nm of ^{3}**Ir^{III*}** + **31** + **18**. The red line is the fit; (h) Kinetics curves for transient absorption at 450 of ^{3}**Ir^{III*}** + **Cu^{II}** under both deoxygenated and air conditions. Experimental conditions (unless otherwise stated): under deoxygenated conditions in MeCN solution following 430 nm. The concentrations of **Ir^{III}**, **31**, **18**, and **Cu^{II}** used are 400 uM, 8 mM, 8 mM, and 8 mM, respectively.

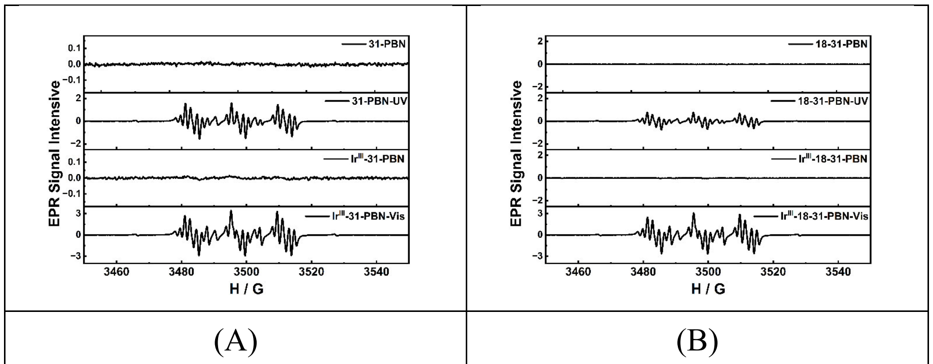

(A) (B)

Figure S4. (a) ESR experiments for trapping radicals using PBN as radical scavenger in MeCN (80μL in total) from the homolysis of **31** under the irradiation of UV and the homolysis of the Ir[dF(CF$_3$)ppy]$_2$(dtbbpy)PF$_6$ & **31** system under the irradiation of visible light; (b) Comparative ESR experiments for trapping radicals using PBN as radical scavenger in MeCN (80μL in total). (PBN: 200mM, 10μL; **31**: 200mM, 20μL; KdoSTol **18**: 200mM, 20μL; Ir[dF(CF$_3$)ppy]$_2$(dtbbpy)PF$_6$: 20mM, 10μL). The spectroscopic data of ·CF$_3$ and R$_1$S$^{·+}$ coincided with the previous report. [19, 20]

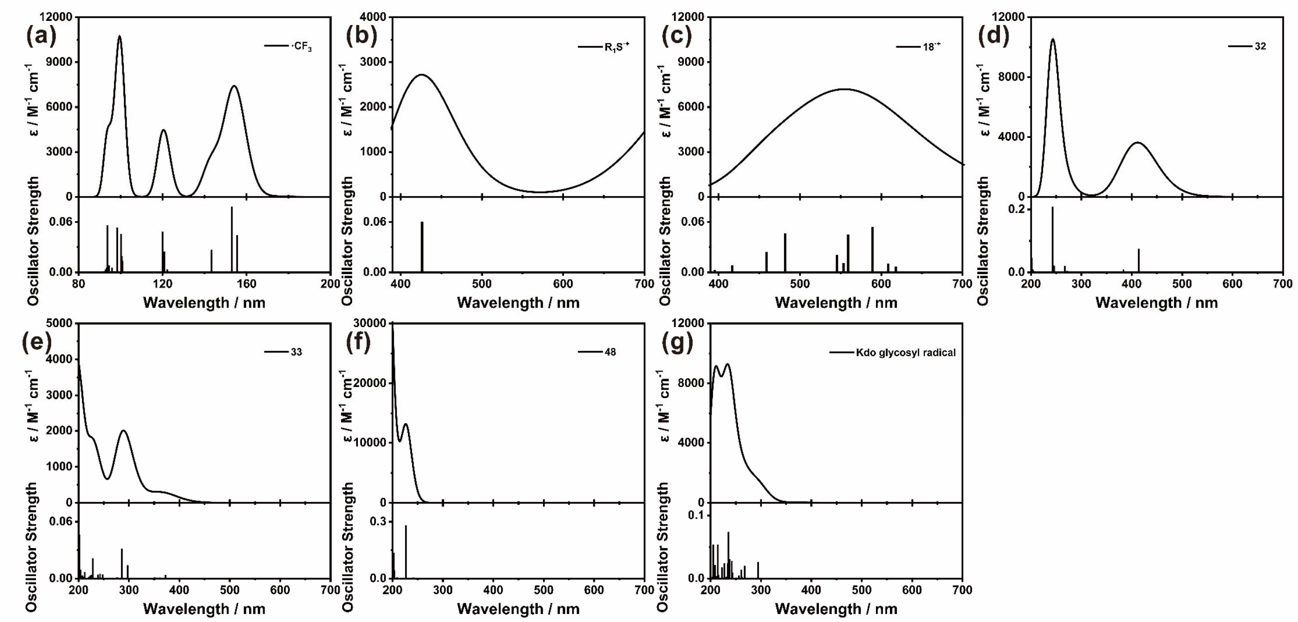

Figure S5. Calculated absorption spectra at the TD-B3LYP-D3(BJ)/6-311+G(d, p)/SMD/ (MeCN/DCM = 1:1) level of theory. UV-Vis peak half-width at half height was set at 0.3 eV. (a) for $\cdot CF_3$; (b) for $R_1S^{\cdot+}$; (c) for $\mathbf{18}^{\cdot+}$; (d) for **32**; (e) for **33**; (f) for **48**; (g) for Kdo glycosyl radical.

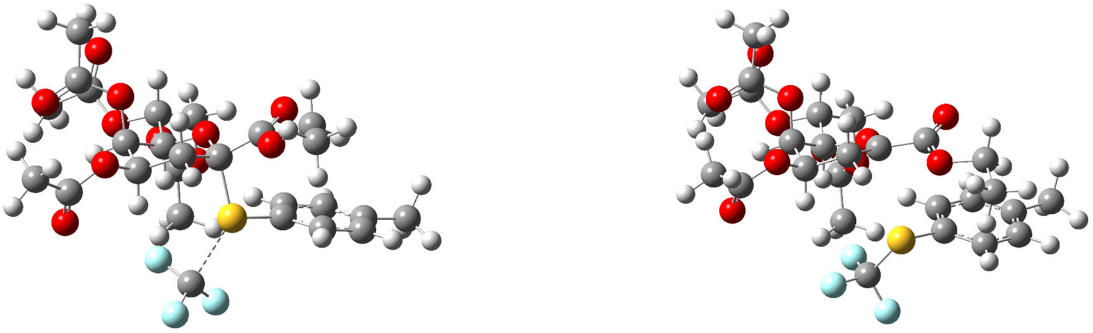

(a) (b)

Figure S6. (a) The input structure of $\mathbf{32}^{+}$; (b) The optimized structure of $\mathbf{32}^{+}$ obtained at the level of B3LYP-D3(BJ)/6-311+G(d, p)/SMD/(MeCN/DCM = 1:1).

In comparison with the input structure of $\mathbf{32}^{+}$ cation, the optimized structure reveals that CF_3STol (**48**) has been already detached, suggesting the expulsion of **48** from the $\mathbf{32}^{+}$ cation to yield the oxacarbenium ion **33** is a barrier-free process. Consequently, once this cation is formed, a rapid detachment of **48** can be anticipated.

Note:

In Figure S3f, the transient absorption spectra resulting from the homolytic cleavage of **31** upon individual excitation and that arising from the quenching of **^{3}Ir^{III*}** by **31** both exhibit a characteristic absorption band around 450 nm. If the quenching of **^{3}Ir^{III*}** by **31** were solely governed by the Dexter energy transfer pathway, its the spectral signature would necessarily coincide precisely with the transient absorption spectrum observed from the homolytic cleavage of **31** excited independently. However, upon meticulous comparison of the transient absorption spectra under these two conditions, subtle differences emerge near 400 nm and within the 500-600 nm region: the spectrum resulting from the reaction of **^{3}Ir^{III*}** + **31** displays negative signals near 400 nm and slightly stronger absorption signals in the 500-600 nm range, compared to the system where **31** is excited alone. This observation suggests the presence of additional transient species beyond those generated solely by homolytic cleavage in the **^{3}Ir^{III*}** + **31** system. This extra transient species indicates that the **^{3}Ir^{III*}** + **31** reaction encompasses quenching pathways other than energy transfer. Upon observing novel spectral features, including negative signals near 400 nm and positive absorption within the 500-600 nm range, which mirror signals documented in literature for oxidized Ir photocatalyst (**Ir^{IV}**)[18], and considering the thermodynamic favorability of **31** quenching **^{3}Ir^{III*}** through electron transfer, as supported by redox potential calculations (Table S6, Entry 1), we hypothesize that, in addition to the primary Dexter energy transfer pathway, an electron transfer quenching mechanism may also contribute to the quenching reaction between **^{3}Ir^{III*}** and **31**.

For Figure S3g, given that the generation of species **18**$^{\cdot+}$ and its decay occur concurrently at the initial time, to eliminate the interference from the process of species **18**$^{\cdot+}$ generation and ensure a more accurate description of its decay kinetics, we chose to analyze its decay behavior starting from 15 μs. We found that its decay can be well described by a second-order reaction behavior, in contrast to the mono-exponential decay.

2 Materials and instruments

General experiments and characterizations. All reactions were carried out under nitrogen atmosphere unless otherwise stated. Solvents used were analytical grade. Chemical reactions were monitored by analytical thin-layer chromatography (TLC) on silica gel F254 glass plates and revealed by UV light (254 nm) or heating after dipping in EtOH-H_2SO_4 (7%). Flash column chromatography was performed on silica gel (200-300 mesh). High-resolution mass spectra (HRMS) were obtained in the ESI mode (Bruker micrOTOF-QII mass spectrometer (ESI)). Optical rotation was determined in chloroform or methanol by an automatic polarimeter. NMR spectra were recorded using $CDCl_3$ as solvents. Chemical shifts (δ) were reported in units per million (ppm) and coupling constants (J) in Hz. ^{1}H NMR spectra, ^{13}C NMR spectra and the selective proton decoupled NMR spectra were recorded on JNM-ECZR spectrometer (400 and 600 MHz) or Bruker Avance III spectrometer (500 MHz). EPR spectra were collected on a Bruker E500 spectrometer.

Laser flash photolysis. Nanosecond time-resolved transient absorption spectra were measured using a flash photolysis setup Edinburgh LP980 spectrometer (Edinburgh Instruments Ltd.). In this work, the sample was excited by a 430 nm or 355 nm laser pulse (1 Hz, 20 mJ/pulse/cm^2, fwhm ≈ 7 ns). A Surelite II-10 Q-Switched Nd:YAG laser (Continuum) provides third harmonic laser pulses at 355 nm with a repetition rate of 10 Hz and maximum output of 1.5 W. The laser output was directed to a Horizon I Mid-band Optical Parametric Oscillator (OPO) (Continuum) to produce 430 nm pump pulse. The pump pulse of 355 nm is generated by a commercial Nd:YAG laser (Lab 170, Spectral Physics Inc.) Each measurement was performed in a quartz cuvette with 1 cm path length at room temperature. The analyzing light was from a 150 W pulsed xenon lamp. A monochromator equipped with a photomultiplier for collecting the spectral range from 300 to 700 nm was used to analyze transient absorption spectra. The signals from the photomultiplier were displayed and recorded as a function of time on a 100 MHz (1.25 Gs/s sampling rate) oscilloscope (Tektronix, TDS 3012C), and the data were transferred to a personal computer. The fitting quality was judged by weighted residuals and reduced $\chi 2$ value.

Steady-state Spectral Measurement. The UV-vis absorption spectra were measured using a UV-vis spectrometer (U-3010, Hitachi). Each measurement was collected from 300 to 700 at a scan speed of 600 nm/min. The spectrum of a blank sample containing the pure solvent was used as the background, which was subtracted from the averaged spectra. Emission spectra were measured on a fluorescence spectrometer (F4600, Hitachi). Quartz cuvettes of 1 × 1 cm were used for all absorption and emission spectral measurements.

3 Experimental procedures

3.1 Preparation of donors

Ethyl (*p*-tolyl 4,5,7,8-tetra-*O*-acetyl-3-deoxy-2-thio-α,β-D-manno-oct-2-ulopyranosid)onate (18)[3]

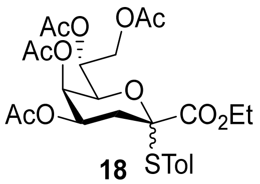

18

Compound **18** was prepared according to the literature method.[3]

18α: ^{1}H NMR (600 MHz, $CDCl_3$): *δ* = 7.31 (d, *J* = 8.1 Hz, 2H), 7.11 (d, *J* = 8.0 Hz, 2H), 5.48 – 5.40 (m, 2H), 5.24 (ddd, *J* = 9.6, 4.3, 2.5 Hz, 1H), 4.72 (dd, *J* = 9.6, 1.0 Hz, 1H), 4.47 (dd, *J* = 12.3, 2.5 Hz, 1H), 4.06 – 4.02 (m, 2H), 4.00 (dd, *J* = 12.2, 4.4 Hz, 1H), 2.43 – 2.38 (m, 1H), 2.34 (s, 3H), 2.30 (t, *J* = 6.6 Hz, 1H), 2.08 (s, 3H), 2.07 (s, 3H), 2.01 (s, 3H), 2.00 (s, 3H), 1.12 (t, *J* = 7.1 Hz, 3H).

18β: ^{1}H NMR (600 MHz, $CDCl_3$): *δ* = 7.43 (d, *J* = 7.6 Hz, 2H), 7.14 (d, *J* = 7.7 Hz, 2H), 5.27 (s, 1H), 5.22 (dd, *J* = 8.4, 6.1 Hz, 1H), 4.93 – 4.82 (m, 1H), 4.57 (d, *J* = 12.1 Hz, 1H), 4.10 – 3.99 (m, 2H), 3.98 – 3.92 (m, 1H), 3.89 (d, *J* = 9.5 Hz, 1H), 2.57 (dd, *J* = 12.6, 4.6 Hz, 1H), 2.35 (s, 3H), 2.19 (t, *J* = 12.6 Hz, 1H), 2.08 (s, 3H), 2.04 (s, 3H), 1.98 (s, 6H), 1.10 (t, *J* = 7.1 Hz, 3H).

The spectroscopic data coincided with the previous report.[3]

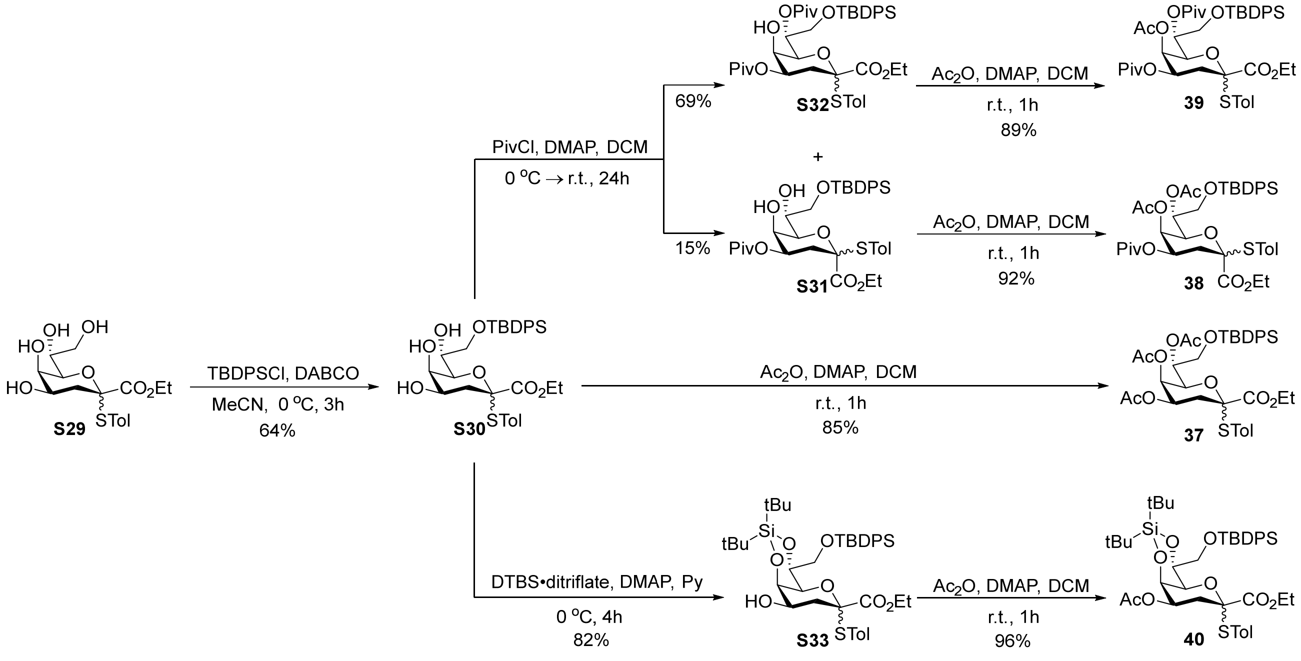

Scheme S1. The synthesis of Kdo donors **37-40**.

Ethyl (*p*-tolyl 8-*O*-*tert*-butyldiphenylsilyl-3-deoxy-2-thio-α,β-D-*manno*-oct-2-ulopyranosid)onate (S30)

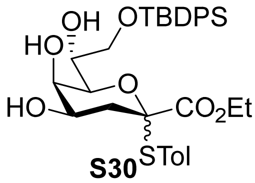

S30

To a solution of **S29**[3] (1.12 g, 3.0 mmol) and DABCO (1.35 g, 12.0 mmol) in dry MeCN (15 mL) were added TBDPSCl (3.0 mL, 12 mmol) at 0 °C under the N_2 atmosphere. The reaction mixture was stirred 3h at 0 °C and then it was quenched by MeOH. The resulting mixture was concentrated in vacuo. The obtained residue was purified by silica gel column chromatography (50:1, CH_2Cl_2-MeOH, v/v) to afford **S30** (1.16 g, 64%) as a white amorphous solid.

S30β: ^{1}H NMR (400 MHz, $CDCl_3$): δ = 7.70 (d, *J* = 7.3 Hz, 2H), 7.66 (d, *J* = 6.8 Hz, 2 H), 7.50 – 7.34 (m, 6H), 7.23 (d, *J* = 7.9 Hz, 2H), 6.74 (d, *J* = 7.9 Hz, 2H), 4.13 (td, *J* = 7.7, 3.8 Hz, 1H), 4.03 (s, 1H), 4.01 (dd, *J* = 10.7, 4.2 Hz, 1H), 3.86 (dq, *J* = 11.3, 7.2 Hz, 1H), 3.71 (dq, *J* = 11.0, 7.2 Hz, 1H), 3.61 (ddd, *J* = 11.5, 4.4, 3.5 Hz, 1H), 3.54 (dd, *J* = 10.3, 8.2 Hz, 1H), 3.29 (d, *J* = 7.7 Hz, 1H), 2.65 (dd, *J* = 12.8, 4.9 Hz, 1H), 2.24 (s, 3H), 1.97 (t, *J* = 12.2 Hz, 1H), 1.06 (s, 9H), 0.80 (t, *J* = 7.1 Hz, 3H).

^{13}C NMR (101 MHz, $CDCl_3$): δ = 168.5 (C1, $^3J_{C\text{-}1/H\text{-}3ax}$ = 7.0 Hz, 101 MHz), 139.8, 136.4, 135.6, 135.6, 133.1, 133.0, 130.2, 130.0, 129.3, 128.1, 128.0, 125.8, 87.3, 76.6, 70.4, 67.3, 6 6.2, 65.8, 61.7, 35.2, 26.9, 21.3, 19.3, 13.7.

HRMS (ESI) m/z Calcd for $C_{33}H_{42}O_7SiSNa$ $[M + Na]^+$ 633.2312, found: 633.2307.

Ethyl (*p*-tolyl 4,5,7-tri-*O*-acetyl-8-*O*-*tert*-butyldiphenylsilyl-3-deoxy-2-thio-α,β-D-*manno*-oct-2-ulopyranosid)onate (37)

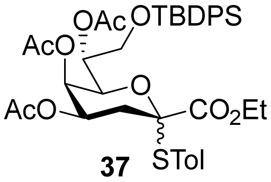

37

To a solution of **S30** (263 mg, 0.43 mmol) and DMAP (211 mg, 1.72 mmol) in dry CH_2Cl_2 (5 mL) were added Ac_2O (488 μL, 5.17 mmol) at room temperature under the N_2 atmosphere. The reaction mixture was stirred 1h and then it was quenched by saturated aqueous $NaHCO_3$, diluted with CH_2Cl_2, washed with water, dried by anhydrous $MgSO_4$ and concentrated in vacuo. The obtained residue was purified by silica gel column chromatography (5:1, Petroleum ether/ethyl acetate, v/v) to afford **37** (269 mg, 85%) as a white amorphous solid.

37α/β: ^{1}H NMR (600 MHz, $CDCl_3$): δ = 7.71 (d, *J* = 6.5 Hz, 2H), 7.64 (dt, *J* = 15.3, 7.1 Hz, 5.4H), 7.49 – 7.39 (m, 7.4H), 7.36 (q, *J* = 7.4 Hz, 3.2H), 7.26 (d, *J* = 7.9 Hz, 2H), 7.

22 (d, J = 8.0 Hz, 1.6H), 6.79 (d, J = 7.9 Hz, 2H), 6.75 (d, J = 7.9 Hz, 1.6H), 5.45 – 5.41 (m, 0.8H), 5.40 (s, 0.8H), 5.38 – 5.34 (m, 1H), 5.30 – 5.26 (m, 0.8H), 5.25 (s, 1H), 4.88 – 4.81 (m, 1H), 4.70 (d, J = 9.6 Hz, 0.8H), 4.06 – 3.98 (m, 2.6H), 3.87 (ddd, J = 11.9, 8.3, 5.4 Hz, 1.8H), 3.81 (dd, J = 11.5, 6.1 Hz, 0.8H), 3.77 – 3.72 (m, 1H), 3.69 (d, J = 9.5 Hz, 1H), 3.66 (dd, J = 11.2, 8.1 Hz, 1H), 2.55 (dd, J = 12.5, 4.7 Hz, 1H), 2.37 (dd, J = 13.6, 4.8 Hz, 0.8H), 2.29 (t, J = 12.7 Hz, 1H), 2.25 (s, 3H), 2.16 (t, J = 12.6 Hz, 0.8H), 2.14 (s, 2.4H), 2.12 (s, 3H), 2.05 (s, 2.4H), 1.99 (s, 2.4H), 1.97 (s, 3H), 1.96 (s, 3H), 1.93 (s, 2.4H), 1.08 (t, J = 7.1 Hz, 2.4H), 1.03 (s, 7.2H), 1.02 (s, 9H), 0.82 (t, J = 7.2 Hz, 3H).

^{13}C NMR (151 MHz, $CDCl_3$): δ = 170.8, 170.7, 170.0, 170.0, 169.9, 169.9, 168.0, 167.9, 140.0, 139.7, 136.5, 135.9, 135.8, 135.8, 135.7, 135.6, 133.8, 133.6, 133.6, 133.5, 130.0, 129.9, 129.8, 129.8, 129.6, 129.4, 127.9, 127.9, 127.8, 127.8, 126.2, 125.6, 89.0, 88.4, 87.7, 73.4, 70.4, 70.3, 69.7, 67.5, 67.1, 64.8, 64.0, 64.0, 63.1, 62.0, 61.8, 32.4, 32.1, 26.9, 26.8, 21.4, 21.2, 21.0, 20.9, 20.9, 19.4, 19.3, 13.9, 13.7.

HRMS (ESI) m/z Calcd for $C_{39}H_{48}O_{10}SiSNa$ $[M + Na]^+$ 759.2629, found: 759.2625.

Ethyl (*p*-tolyl 4-*O*-trimethylacetyl-5,7-di-*O*-acetyl-8-*O*-*tert*-butyldiphenylsilyl-3-deoxy-2-thio-α,β-D-*manno*-oct-2-ulopyranosid)onate (38)

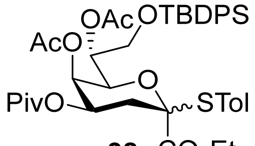

38

To a solution of **S30** (534 mg, 0.87 mmol) and DMAP (428 mg, 3.5 mmol) in dry DCM (15 mL) were added PivCl (1.3 mL, 10.5 mmol) at 0 °C under the N_2 atmosphere. The reaction mixture was stirred 24h at room temperature and then it was quenched by saturated aqueous $NaHCO_3$, diluted with CH_2Cl_2, washed with water, dried by anhydrous Na_2SO_4 and concentrated in vacuo. The obtained residue was purified by silica gel column chromatography (3:1, Petroleum ether/ethyl acetate, v/v) to afford **S31** (91 mg, 15%) as a colorless syrup.

S31β: ^{1}H NMR (600 MHz, $CDCl_3$): δ = 7.70 (d, J = 7.1 Hz, 2H), 7.67 (d, J = 7.3 Hz, 2H), 7.44 (dt, J = 18.5, 9.3 Hz, 4H), 7.36 (t, J = 7.1 Hz, 2H), 7.23 (d, J = 7.3 Hz, 2H), 6.73 (d, J = 7.5 Hz, 2H), 4.77 – 4.63 (m, 1H), 4.20 – 4.12 (m, 2H), 4.01 (d, J = 10.2 Hz, 1H), 3.92 – 3.82 (m, 1H), 3.80 – 3.69 (m, 1H), 3.55 (t, J = 9.1 Hz, 1H), 3.35 (d, J = 7.9 Hz, 1H), 2.60 (dd, J = 12.3, 4.4 Hz, 1H), 2.23 (s, 3H), 2.21 (t, J = 12.3 Hz, 1H), 1.22 (s, 9H), 1.06 (s, 9H), 0.81 (t, J = 7.1 Hz, 3H).

^{13}C NMR (151 MHz, $CDCl_3$): δ = 177.4, 168.2 (C1, $^3J_{C\text{-}1/H\text{-}3ax}$ = 6.4 Hz, 151 MHz), 139.9,

136.4, 135.7, 135.6, 133.2, 133.2, 130.1, 130.0, 129.3, 128.1, 128.0, 125.8, 87.3, 76.6, 70.0, 69.6, 65.8, 64.4, 61.8, 39.0, 31.6, 27.3, 27.2, 27.0, 21.4, 19.3, 13.8.
HRMS (ESI) m/z Calcd for $C_{38}H_{51}O_8SiS$ $[M + H]^+$ 695.3068, found: 695.3062.

To a solution of **S31β** (209 mg, 0.3 mmol) and DMAP (110 mg, 0.9 mmol) in dry CH_2Cl_2 (5 mL) were added Ac_2O (227 μL, 2.4 mmol) at room temperature under the N_2 atmosphere. The reaction mixture was stirred 1h and then it was quenched by saturated aqueous $NaHCO_3$, diluted with CH_2Cl_2, washed with water, dried by anhydrous $MgSO_4$ and concentrated in vacuo. The obtained residue was purified by silica gel column chromatography (8:1, Petroleum ether/ethyl acetate, v/v) to afford **38β** (215 mg, 92%) as a white amorphous solid.

38β: ^{1}H NMR (400 MHz, $CDCl_3$): δ = 7.73 – 7.68 (m, 2H, OTBDPS), 7.63 (d, *J* = 7.0 Hz, 2H, OTBDPS), 7.42 (dt, *J* = 7.0, 5.8 Hz, 4H, OTBDPS), 7.35 (t, *J* = 7.3 Hz, 2H, OTBDPS), 7.26 (d, *J* = 7.6 Hz, 2H, STol), 6.78 (d, *J* = 7.9 Hz, 2H, STol), 5.40 – 5.28 (m, 2H, H-5 & H-7), 4.86 – 4.72 (m, 1H, H-4), 4.02 (dd, *J* = 11.2, 2.3 Hz, 1H, H-8), 3.87 (dq, *J* = 10.7, 7.1 Hz, 1H, CH_2 of Et group), 3.74 (dt, *J* = 12.5, 8.2 Hz, 2H, H-6 & CH_2 of Et group), 3.65 (dd, *J* = 11.2, 8.0 Hz, 1H, H-8), 2.58 (dd, *J* = 12.4, 4.8 Hz, 1H, H-3), 2.25 (s, 3H, CH_3 of STol), 2.15 (t, *J* = 12.4 Hz, 1H, H-3), 2.11 (s, 3H, OAc), 1.98 (s, 3H, OAc), 1.13 (s, 9H, OPiv), 1.01 (s, 9H, OTBDPS), 0.81 (t, *J* = 7.1 Hz, 3H, CH_3 of Et group).
^{13}C NMR (101 MHz, $CDCl_3$): δ = 177.1, 170.6, 170.0, 167.9 (C1, $^3J_{C\text{-}1/H\text{-}3ax}$ = 6.9 Hz, 101 MHz), 140.0, 136.4, 135.8, 135.7, 133.6, 133.6, 130.0, 129.8, 129.4, 127.9, 127.8, 125.6, 87. 6, 73.5, 70.2, 67.4, 64.0, 63.9, 62.0, 38.8, 32.2, 27.1, 26.8, 21.4, 21.0, 20.9, 19.3, 13.8.
HRMS (ESI) m/z Calcd for $C_{42}H_{55}O_{10}SiS$ $[M + H]^+$ 779.3279, found: 779.3288.

Ethyl (*p*-tolyl 4,7-*O*-di-trimethylacetyl-5-*O*-acetyl-8-*O*-*tert*-butyldiphenylsilyl-3-deoxy-2-thio-α,β-D-*manno*-oct-2-ulopyranosid)onate (39)

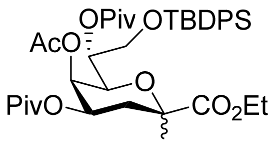

39

To a solution of **S30** (534 mg, 0.87 mmol) and DMAP (428 mg, 3.5 mmol) in dry DCM (15 mL) were added PivCl (1.3 mL, 10.5 mmol) at 0 °C under the N_2 atmosphere. The reaction mixture was stirred 24h at room temperature and then it was quenched by saturated aqueous $NaHCO_3$, diluted with CH_2Cl_2, washed with water, dried by anhydrous Na_2SO_4 and concentrated in vacuo. The obtained residue was purified by silica gel column chromatography (15:1, Petroleum ether/ethyl acetate, v/v) to afford **S32** (468 mg, 69%) as a colorless syrup.

S32α: ^{1}H NMR (400 MHz, $CDCl_3$): δ = 7.65 (ddd, *J* = 11.0, 7.9, 1.5 Hz, 4H), 7.47 – 7.33 (m, 6H), 7.17 (d, *J* = 8.1 Hz, 2H), 6.76 (d, *J* = 7.9 Hz, 2H), 5.34 (td, *J* = 8.1, 2.8 Hz, 1 H), 5.24 (ddd, *J* = 12.3, 4.8, 3.0 Hz, 1H), 4.29 (d, *J* = 8.6 Hz, 1H), 4.01 – 3.91 (m, 3H),

3.84 (d, J = 2.5 Hz, 1H), 3.80 (dd, J = 11.2, 7.7 Hz, 1H), 2.41 (t, J = 7.1 Hz, 1H), 2.27 (dd, J = 13.5, 4.6 Hz, 1H), 2.14 (s, 3H), 1.25 (s, 9H), 1.21 (s, 9H), 1.01 (s, 9H), 0.98 (t, J = 7.1 Hz, 3H).

^{13}C NMR (151 MHz, $CDCl_3$): δ = 179.4, 177.8, 168.4 (C1, $^3J_{C\text{-}1/H\text{-}3ax}$ = 0 Hz, 101 MHz), 1 39.5, 135.8, 135.8, 135.7, 135.6, 133.6, 133.2, 129.8, 129.8, 129.5, 127.8, 127.8, 126.5, 89.0, 72.6, 72.0, 69.0, 64.3, 63.3, 61.7, 39.2, 39.0, 31.3, 27.4, 27.2, 27.2, 27.2, 26.9, 21.2, 19.3, 13.9.

HRMS (ESI) m/z Calcd for $C_{43}H_{59}O_9SiS$ $[M + H]^+$ 779.3643, found: 779.3639.

To a solution of **S32α** (234 mg, 0.3 mmol) and DMAP (79 mg, 0.64 mmol) in dry CH_2Cl_2 (5 mL) were added Ac_2O (121 µL, 1.28 mmol) at room temperature under the N_2 atmosphere. The reaction mixture was stirred 1h and then it was quenched by saturated aqueous $NaHCO_3$, diluted with CH_2Cl_2, washed with water, dried by anhydrous $MgSO_4$ and concentrated in vacuo. The obtained residue was purified by silica gel column chromatography (10:1, Petroleum ether/ethyl acetate, v/v) to afford **39α** (218 mg, 89%) as a white amorphous solid.

39α: ^{1}H NMR (400 MHz, $CDCl_3$): δ = 7.68 – 7.59 (m, 4H, OTBDPS), 7.46 – 7.33 (m, 6H, OTBDPS), 7.16 (d, J = 8.1 Hz, 2H, STol), 6.73 (d, J = 7.9 Hz, 2H, STol), 5.45 – 5.34 (m, 3H, H-4 & H-7 & H-5), 4.55 – 4.46 (m, 1H, H-6), 4.02 (q, J = 7.0 Hz, 2H, CH_2 of Et group), 3.87 (dd, J = 11.1, 2.3 Hz, 1H, H-8), 3.71 (dd, J = 11.1, 8.0 Hz, 1H, H-8), 2.33 – 2.20 (m, 2H, H-3), 2.12 (s, 3H, CH_3 of STol), 2.06 (s, 3H, OAc), 1.21 (s, 9H, OPiv), 1.13 (s, 9H, OPiv), 1.04 (t, J = 7.2 Hz, 3H, CH_3 of Et group), 1.00 (s, 9H, OTBDPS).

^{13}C NMR (101 MHz, $CDCl_3$): δ = 177.3, 177.2, 170.0, 168.3 (C1, $^3J_{C\text{-}1/H\text{-}3ax}$ = 0 Hz, 151 M Hz), 139.7, 135.8, 135.7, 133.8, 133.2, 129.8, 129.7, 129.6, 127.8, 127.7, 88.7, 70.7, 70.1, 6 7.2, 64.5, 64.1, 61.8, 39.0, 38.8, 32.0, 27.3, 27.1, 26.9, 21.2, 21.0, 19.3, 13.9.

HRMS (ESI) m/z Calcd for $C_{45}H_{61}O_{10}SiS$ $[M + H]^+$ 821.3749, found: 821.3756.

Ethyl (*p*-tolyl 4-*O*-acetyl-5,7-di-*O*-*tert*-butylsilyl-8-*O*-*tert*-butyldiphenylsilyl-3-deoxy-2-thio-α,β-D-*manno*-oct-2-ulopyranosid)onate (40)

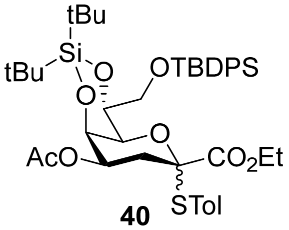

40

To a solution of **S30** (264 mg, 0.4 mmol) and DMAP (29 mg, 0.24 mmol) in dry pyridine (4.4 mL) were added DTBS ditriflate (150 µL, 0.46 mmol) at 0 °C under the N_2 atmosphere. The reaction mixture was stirred 4h at 0 °C and then it was quenched by saturated aqueous NH_4Cl, diluted with

EtOAc, washed with 1 M HCl, saturated aqueous $NaHCO_3$ and saturated aqueous NaCl, dried by anhydrous Na_2SO_4 and concentrated in vacuo. The obtained residue was purified by silica gel column chromatography (10:1, Petroleum ether/ethyl acetate, v/v) to afford **S33** (246 mg, 82%) as a white amorphous solid. To a solution of **S33** (225 mg, 0.3 mmol) and DMAP (79 mg, 0.64 mmol) in dry CH_2Cl_2 (5 mL) were added Ac_2O (121 μL, 1.28 mmol) at room temperature under the N_2 atmosphere. The reaction mixture was stirred 1h and then it was quenched by saturated aqueous $NaHCO_3$, diluted with CH_2Cl_2, washed with water, dried by anhydrous $MgSO_4$ and concentrated in vacuo. The obtained residue was purified by silica gel column chromatography (10:1, Petroleum ether/ethyl acetate, v/v) to afford **40** (228 mg, 96%) as a white amorphous solid.

40α/β: ^{1}H NMR (600 MHz, $CDCl_3$): δ = 7.71 (dd, *J* = 12.2, 4.7 Hz, 4H), 7.67 – 7.62 (m, 2.66H), 7.44 – 7.33 (m, 11.26H), 7.27 (d, *J* = 6.9 Hz, 2H), 7.09 (d, *J* = 7.9 Hz, 1.32H), 6. 99 (d, *J* = 7.9 Hz, 2H), 5.17 (ddd, *J* = 12.2, 4.5, 2.5 Hz, 1H), 4.72 (s, 1H), 4.66 (ddd, *J* = 12.1, 4.5, 2.5 Hz, 0.66H), 4.40 (d, *J* = 1.8 Hz, 1H), 4.36 (dd, *J* = 8.9, 5.6 Hz, 1.66H), 4.29 (t, *J* = 5.5 Hz, 0.66H), 4.04 (dq, *J* = 10.7, 7.1 Hz, 1H), 3.99 – 3.93 (m, 0.66H), 3.93 – 3.87 (m, 1.66H), 3.80 – 3.75 (m, 1.66H), 3.71 (dd, *J* = 10.2, 8.2 Hz, 0.66H), 3.68 (d, *J* = 5.6 Hz, 1H), 2.55 – 2.49 (m, 1.66H), 2.44 (t, *J* = 12.1 Hz, 0.66H), 2.37 – 2.34 (m, 2.98 H), 2.29 (s, 3H), 2.11 (s, 3H), 2.10 (s, 1.98H), 1.18 (s, 5.94H), 1.07 (s, 9H), 1.05 (t, *J* = 7. 1 Hz, 3H), 1.02 (s, 5.94H), 0.98 (t, *J* = 7.5 Hz, 1.98H), 0.97 (s, 9H), 0.94 (s, 5.94H), 0.91 (s, 9H).

^{13}C NMR (151 MHz, $CDCl_3$): δ = 170.3, 170.3, 168.5, 168.3, 139.8, 139.3, 136.5, 135.8, 13 5.8, 135.8, 133.4, 133.4, 133.3, 133.1, 130.0, 130.0, 129.8, 129.8, 129.6, 129.5, 128.0, 128.0, 127.8, 126.8, 126.4, 89.2, 86.8, 77.2, 76.6, 75.0, 71.1, 70.5, 70.1, 66.6, 66.0, 66.0, 65.7, 6 1.7, 61.5, 31.6, 28.1, 27.9, 27.4, 27.3, 27.0, 27.0, 22.2, 22.1, 21.8, 21.6, 21.4, 21.4, 21.2, 2 1.1, 19.3, 14.0, 14.0.

HRMS (ESI) m/z Calcd for $C_{43}H_{60}O_8Si_2SNa$ $[M + Na]^+$ 815.3439, found: 815.3430.

Ethyl (*p*-tolyl 4,5:7,8-di-*O*-isopropylidene-3-deoxy-2-thio-α,β-D-manno-oct-2-ulopyranosid)onate (41)

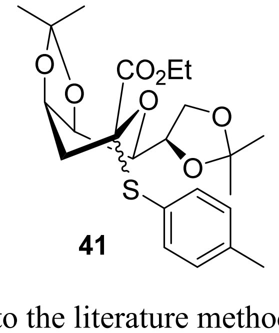

41

Compound **41** was prepared according to the literature method.[3]

41α: ^{1}H NMR (600 MHz, $CDCl_3$): δ = 7.38 (d, J = 8.0 Hz, 2H), 7.09 (d, J = 7.9 Hz, 2H), 4.54 (dt, J = 7.2, 3.0 Hz, 1H), 4.38 – 4.32 (m, 2H), 4.15 (ddd, J = 13.4, 8.5, 5.6 Hz, 2H), 3.97 – 3.84 (m, 2H), 3.82 (dd, J = 7.7, 1.5 Hz, 1H), 3.11 (dd, J = 15.3, 3.7 Hz, 1H), 2.33 (s, 3H), 1.94 (dd, J = 15.3, 2.3 Hz, 1H), 1.49 (s, 3H), 1.40 (s, 3H), 1.35 (s, 3H), 1.31 (s, 3H), 1.01 (t, J = 7.1 Hz, 3H).

The spectroscopic data coincided with the previous report.[3]

3.2 Preparation of acceptors

S1 (1-Octanol) was purchased from Alfa Aesar of Thermo Fisher Scientific Inc.

S2 (Benzyl (3-Hydroxypropyl) Carbamate) was purchased from Adamas-beta of Shanghai Titan Scientific Co., Ltd.

S3 (3-Chloro-1-propanol) was purchased from Shanghai Aladdin Biochemical Technology Co., Ltd.

S4 (2-Azidoethanol) was purchased from Innochem (Beijing) Technology Co., Ltd.

S5 (N-Boc-L-Serine methyl ester) was purchased from Innochem (Beijing) Technology Co., Ltd.

S6 (1,6-Hexanediol) was purchased from Innochem (Beijing) Technology Co., Ltd.

S7 (3-Butyn-1-ol) was purchased from Innochem (Beijing) Technology Co., Ltd.

S8 (5-Chloropentanol) was purchased from Innochem (Beijing) Technology Co., Ltd.

S9 (Benzyl alcohol) was purchased from Innochem (Beijing) Technology Co., Ltd.

S10 (3,5-Di-Tert-Butylbenzyl Alcohol) was purchased from Adamas-beta of Shanghai Titan Scientific Co., Ltd.

S11 (L-(-)-Menthol) was purchased from Innochem (Beijing) Technology Co., Ltd.

S12 (2-Adamanthanol) was purchased from Innochem (Beijing) Technology Co., Ltd.

S13 (Epiandrosterone) was purchased from Innochem (Beijing) Technology Co., Ltd.

S14 (3β-Cholestanol) was purchased from Alfa Aesar of Thermo Fisher Scientific Inc.

S15 (1-Adamanthanol) was purchased from Acros Organics of Thermo Fisher Scientific Inc.

S16 was synthesized according to the previously published procedure.[3, 22, 23]

Methyl 2,3,4-tri-*O*-benzyl-α-D-glucopyranoside (S16)[3, 22, 23]

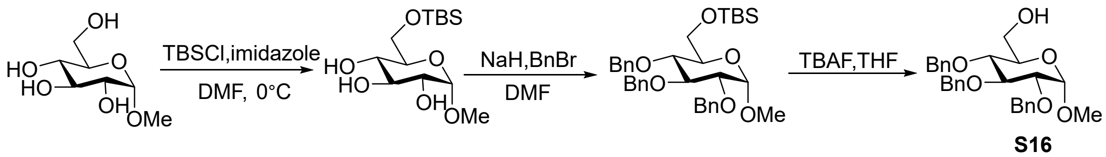

^{1}H NMR (600 MHz, $CDCl_3$): δ = 7.39 – 7.28 (m, 15H), 4.99 (d, *J* = 10.9 Hz, 1H), 4.89 (d, *J* = 11.0 Hz, 1H), 4.84 (d, *J* = 10.9 Hz, 1H), 4.80 (d, *J* = 12.0 Hz, 1H), 4.65 (dd, *J* = 14.2, 11.6 Hz, 2H), 4.57 (d, *J* = 3.4 Hz, 1H), 4.01 (t, *J* = 9.3 Hz, 1H), 3.77 (dd, *J* = 11.7, 2.7 Hz, 1H), 3.69 (dd, *J* = 11.7, 4.1 Hz, 1H), 3.65 (dt, *J* = 9.9, 3.3 Hz, 1H), 3.53 (d, *J* = 9.4 Hz, 1H), 3.50 (dd, *J* = 9.7, 3.6 Hz, 1H), 3.37 (s, 3H). The spectroscopic data coincided with the previous report.[3, 22, 23]

S17 (Methyl 2,3,4-Tri-*O*-benzoyl-α-D-Glucopyranoside) was purchased from Adamas-beta of Shanghai Titan Scientific Co., Ltd.

S18 was synthesized according to the previously published procedure.[2]

Methyl 2-acetamido-3,4-di-*O*-benzyl-2-deoxy-α-D-glucopyranoside (S18)[2]

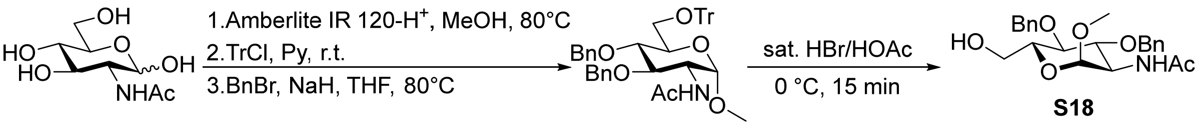

H NMR (400 MHz, $CDCl_3$): δ = 7.36 – 7.28 (m, 10H), 5.29 (t, *J* = 9.8 Hz, 1H), 4.87 (dd, *J* = 11.3, 6.6 Hz, 2H), 4.70 – 4.62 (m, 3H), 4.19 (td, *J* = 9.7, 3.5 Hz, 1H), 3.82 (dd, *J* = 11.9, 2.0 Hz, 1H), 3.76 – 3.62 (m, 4H), 3.31 (s, 3H), 2.07 – 1.96 (m, 1H), 1.84 (s, 3H).
The spectroscopic data coincided with the previous report.[2]

S19 (1,2:3,4-Di-*O*-isopropylidene-D-galactopyranose) was purchased from TCI (Shanghai) Development Co., Ltd.

S20 (Diacetonefructose) was purchased from Innochem (Beijing) Technology Co., Ltd.

S21 (Diacetone-D-glucose) was purchased from Innochem (Beijing) Technology Co., Ltd.

S22 (D-Glucurono-6,3-Lactone Acetonide) was purchased from Adamas-beta of Shanghai Titan Scientific Co., Ltd.

S23 (Methyl 2,3-*O*-isopropylidene-α-L-rhamnopyranoside) was purchased from Shanghai Aladdin

Biochemical Technology Co., Ltd.

Ethyl (2-*O*-acetyl-7,8-di-*O*-isopropylidene-3-deoxy-α-D-manno-oct-2-ulopyranosyl)onate (S24)

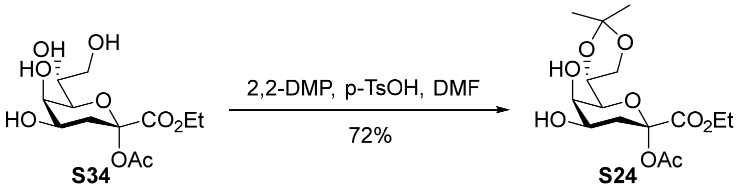

To a solution of **S34**[3] (2.0 g, 6.5 mmol) in dry DMF (60 mL) were added 2,2-dimethoxypropane (1.0 mL, 8 mmol) and a catalytic amount of *p*-toluenesulfonic acid (112 mg, 0.65 mmol) at room temperature. The reaction mixture was stirred overnight at room temperature and then it was quenched by Et_3N. The resulting mixture was concentrated in vacuo. The obtained residue was purified by silica gel column chromatography (50:1, CH_2Cl_2-MeOH, v/v) to afford **S24** (1.62 g, 72%) as a white amorphous solid.

S24: ^1H NMR (600 MHz, $CDCl_3$): δ = 4.39 (dt, *J* = 9.5, 5.2 Hz, 1H, H-7), 4.24 (q, *J* = 7. 0 Hz, 2H, CH_2CH_3), 4.12 (dd, *J* = 8.7, 6.5 Hz, 1H, H-8), 4.08 – 4.01 (m, 2H, H-4 and H-5), 3.88 (dd, *J* = 9.0, 3.9 Hz, 1H, H-8), 3.53 (d, *J* = 8.4 Hz, 1H, H-6), 3.15 (broad, 2H, O H), 2.22 (dd, *J* = 12.2, 3.4 Hz, 1H, H-3eq), 2.11 (s, 3H, OAc) 2.01 (t, *J* = 12.3 Hz, 1H, H-3ax), 1.42 (s, 3H, CH_3), 1.36 (s, 3H, CH_3), 1.27 (t, *J* = 7.1 Hz, 3H, CH_2CH_3).

^{13}C NMR (151 MHz, $CDCl_3$): δ = 168.6 (C1, $^3J_{C\text{-}1/H\text{-}3ax}$ = 0 Hz, 151 MHz), 167.2, 109.7, 9 8.0, 74.9, 73.1, 67.5, 66.4, 65.7, 62.4, 33.8, 27.0, 25.1, 20.9, 14.0.

HRMS (ESI) m/z Calcd for $C_{15}H_{24}O_9Na$ $[M + Na]^+$ 371.1312, found: 371.1319.

S25 was synthesized according to the previously published procedure.[3, 24]

Methyl 2-*O*-benzyl-4,6-*O*-benzylidene-α-D-mannopyranoside (S25)[3, 24]

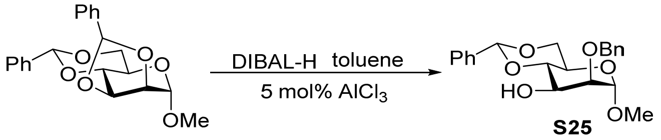

^1H NMR (600 MHz, CD_3CN): δ = 7.56 – 7.29 (m, 10H), 5.59 (s, 1H), 4.79 (s, 1H), 4.76 – 4.66 (m, 2H), 4.19 (dd, *J* = 10.0, 4.7 Hz, 1H), 3.90 (ddd, *J* = 10.6, 7.3, 3.5 Hz, 1H), 3.83 (t, *J* = 9.6 Hz, 1H), 3.77 – 3.71 (m, 2H), 3.67 (dd, *J* = 9.5, 4.7 Hz, 1H), 3.35 (s, 3H), 3.15 (d, *J* = 7.4 Hz, 1H).

The spectroscopic data coincided with the previous report.[3, 24]

S26 (1,2:5,6-Di-*O*-isopropylidene-α-D-allofuranose) was purchased from Shanghai Haohong

Scientific Co., Ltd.

2-Azidoethyl 2-*O*-benzoyl-4,6-*O*-benzylidene-β-D-galactopyranoside (S27)

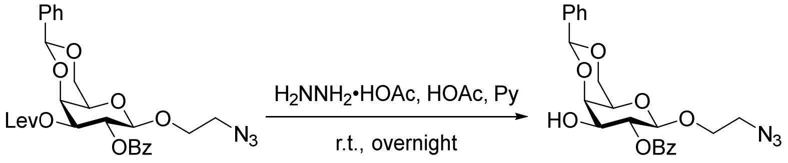

S35 **S27**

To a solution of **S35**[3] (287 mg, 0.53 mmol) in pyridine-HOAc mixed solvent (2.4 mL, Py/HOAc = 3:1, v/v) were added H_2NNH_2·HOAc (398 mg, 4.3 mmol). The reaction mixture was stirred overnight at room temperature and then it was quenched by saturated aqueous $NaHCO_3$, diluted with CH_2Cl_2, washed with saturated aqueous $NaHCO_3$, dried by anhydrous Na_2SO_4 and concentrated in vacuo. The obtained residue was purified by silica gel column chromatography (1:1, Petroleum ether/ethyl acetate, v/v) to afford **S27** (178 mg, 76%) as a white amorphous solid.

^{1}H NMR (600 MHz, $CDCl_3$): δ = 8.08 (d, *J* = 7.8 Hz, 2H), 8.10 – 8.06 (m, 3H), 7.44 (t, *J* = 7.6 Hz, 2H), 7.40 (d, *J* = 5.9 Hz, 3H), 5.60 (s, 1H), 5.41 (t, *J* = 8.5 Hz, 1H), 4.72 (d, *J* = 8.0 Hz, 1H), 4.39 (d, *J* = 12.3 Hz, 1H), 4.29 (d, *J* = 3.7 Hz, 1H), 4.14 (d, *J* = 12.4 Hz, 1H), 4.10 – 4.04 (m, 1H), 3.92 (dd, *J* = 9.9, 3.6 Hz, 1H), 3.76 – 3.72 (m, 1H), 3.59 (s, 1H), 3.48 – 3.42 (m, 1H), 3.32 (dt, *J* = 13.1, 4.2 Hz, 1H), 1.55 (s, 1H).

^{13}C NMR (151 MHz, $CDCl_3$): δ = 166.5, 137.4, 133.2, 130.1, 130.0, 129.5, 128.5, 128.4, 12 6.6, 101.7, 100.9, 75.7, 72.6, 72.0, 69.1, 67.7, 66.9, 50.9.

HRMS (ESI) m/z Calcd for $C_{22}H_{27}O_7N_4$ $[M + NH_4]^+$ 459.1874, found: 459.1878.

Note: **S35** was synthesized by the (*p*-Tol)$_2$SO/Tf_2O preactivation strategy.

S28 was synthesized according to the previously published procedure.[11]

Methyl 2-*O*-acetyl-4-*O*-benzyl-α-L-rhamnopyranoside (S28)[11]

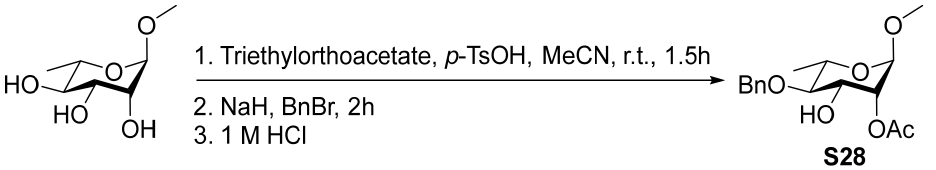

S28

^{1}H NMR (600 MHz, $CDCl_3$): δ = 7.36 (d, *J* = 4.2 Hz, 4H), 7.33 – 7.28 (m, 1H), 5.09 (dd, *J* = 3.4, 1.3 Hz, 1H), 4.83 (d, *J* = 11.1 Hz, 1H), 4.72 (d, *J* = 11.1 Hz, 1H), 4.62 (s, 1H), 4.09 (dd, *J* = 9.4, 3.6 Hz, 1H), 3.72 (dq, *J* = 9.5, 6.3 Hz, 1H), 3.37 – 3.32 (m, 4H), 2.15 (s, 3H), 1.36 (d, *J* = 6.3 Hz, 3H).

The spectroscopic data coincided with the previous report.[11]

Copper(II) trifluoromethanesulfonate was purchased from Shanghai Aladdin Biochemical Technology Co., Ltd.

Umemoto's reagent (S-(Trifluoromethyl)dibenzothiophenium trifluoromethanesulfonate) was purchased from Innochem (Beijing) Technology Co., Ltd.

***p*-Tolyl Sulfoxide** was purchased from TCI (Shanghai) Development Co., Ltd.

All photocatalysts were purchased from Shanghai Macklin Biochemical Technology Co., Ltd.

Solvents used for column chromatography were purchased from Bei Jing TongGuang Fine Chemicals Company.

3.3 General procedure (GP) for glycosidation reactions

GP1: To a mixture of thioglycoside donor **18** (1.0 equiv., 0.060 mmol), Umemoto's reagent (1.5 equiv.), Ir[dF(CF_3)ppy]$_2$(dtbbpy)PF_6 (0.04 equiv.), Cu(OTf)$_2$ (0-1.5 equiv.), acceptor **S1**-**S28** (1.5 equiv.) and activated 4Å powdered sieves (400 mg) in flame-dried glass vessel was added anhydrous MeCN/CH_2Cl_2 mixed solvent (1.8 mL) under the N_2 atmosphere. The resulting mixture was stirred at -78°C for 15 min, followed by the irradiation of blue LED light (465nm) for 12h or overnight. The reaction mixture was quenched with the cessation of irradiation and Et_3N (0.1 mL), diluted with CH_2Cl_2, filtered through diatomite, washed with water, dried by anhydrous $MgSO_4$ and concentrated to leave a residue which was purified by column chromatography on silica gel to afford target glycosides.

GP2: To a mixture of thioglycoside donor **18** (1.0 equiv., 0.060 mmol), Umemoto's reagent (1.5 equiv.), Ir[dF(CF_3)ppy]$_2$(dtbbpy)PF_6 (0.04 equiv.), Cu(OTf)$_2$ (0.5 or 1.5 equiv.), acceptor **S1**-**S28** (1.5 equiv.), (*p*-Tol)$_2$SO (0-4.0 equiv.) and activated 4Å powdered sieves (400 mg) in flame-dried glass vessel was added anhydrous MeCN/CH_2Cl_2 mixed solvent (1.8 mL) under the N_2 atmosphere. The resulting mixture was stirred at -30°C for 15 min, followed by the irradiation of blue LED light (465nm) for 12h or overnight. The reaction mixture was quenched with the cessation of irradiation and Et_3N (0.3 mL), diluted with CH_2Cl_2, filtered through diatomite, washed with water, dried by anhydrous $MgSO_4$ and concentrated to leave a residue which was purified by column chromatography on silica gel to afford target glycosides.

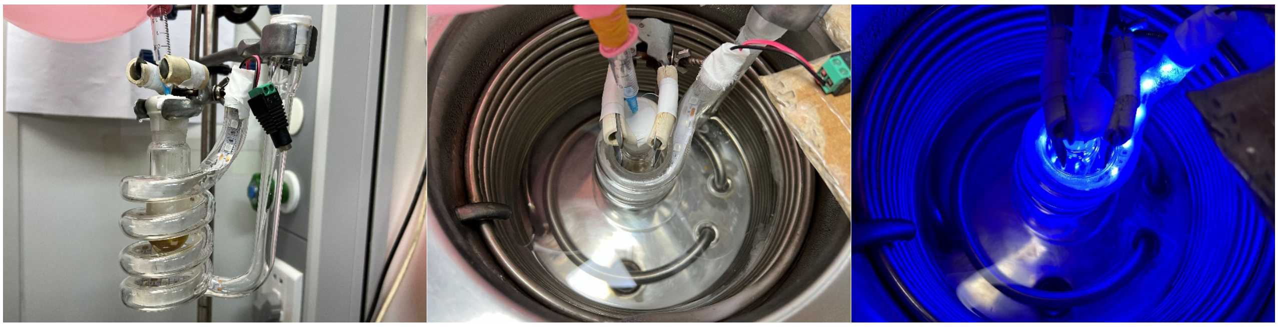

(LED: 460-465nm, 0.2W per 5050 LED lamp bead)

Ethyl (1-Octyl 4,5,7,8-tetra-*O*-acetyl-3-deoxy-β-D-manno-oct-2-ulopyranosid)onate (36aβ)

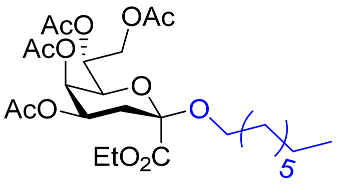

36aβ

GP1: 32.4 mg, 99% yield from **18**: a colorless oil. (Petroleum ether/ethyl acetate = 6:1, v/v)

36aβ: ^{1}H NMR (400 MHz, $CDCl_3$): δ = 5.29 – 5.26 (m, 1H), 5.17 (dt, *J* = 9.6, 3.4 Hz, 1H), 4.87 (ddd, *J* = 13.2, 4.6, 3.0 Hz, 1H), 4.36 (d, *J* = 3.4 Hz, 2H), 4.25 (q, *J* = 7.1 Hz, 2H), 4.19 (dd, *J* = 9.6, 1.2 Hz, 1H), 3.73 (dt, *J* = 9.2, 6.5 Hz, 1H), 3.28 (dt, *J* = 9.2, 6.8 Hz, 1H), 2.36 (dd, *J* = 12.5, 4.4 Hz, 1H), 2.10 (s, 3H), 2.09 (s, 3H), 2.08 (t, *J* = 12.8 Hz, 1H), 1.99 (s, 3H), 1.98 (s, 3H), 1.54 (p, *J* = 6.8 Hz, 2H), 1.32 (t, *J* = 7.1 Hz, 3H), 1.26 (s, 10H), 0.87 (t, *J* = 6.8 Hz, 3H).

^{13}C NMR (101 MHz, $CDCl_3$): δ = 170.9, 170.7, 170.1, 169.9, 168.0 (C1, $^3J_{C\text{-}1/H\text{-}3ax}$ = 5.2 Hz, 151 MHz), 99.5, 70.7, 68.2, 67.3, 64.9, 64.2, 62.6, 62.1, 32.6, 31.9, 29.7, 29.4, 29.3, 26.0, 22.8, 21.0, 20.9, 20.8, 20.8, 14.3, 14.2.

HRMS (ESI) m/z Calcd for $C_{26}H_{42}O_{12}Na$ $[M + Na]^+$ 569.2568, found: 569.2570.

Ethyl (1-Octyl 4,5,7,8-tetra-*O*-acetyl-3-deoxy-α-D-manno-oct-2-ulopyranosid)onate (36aα)

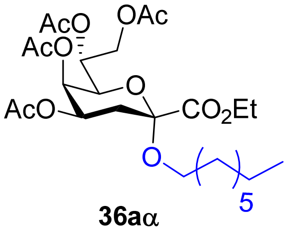

36aα

GP2: 21.1 mg, 64% yield from **18** to generate the mixture of **36aβ** and **36aα** (1:1.4): a pale-yellow oil. (Petroleum ether/ethyl acetate = 6:1, v/v)

The mixture of **36aβ** and **36aα** (1:1.4)**:** ^{1}H NMR (600 MHz, $CDCl_3$): δ = 5.38 – 5.32 (m, 2.4H), 5.28 (s, 1H, **β**), 5.23 (d, *J* = 9.8 Hz, 1.4H, **α**), 5.17 (dd, *J* = 8.1, 3.7 Hz, 1H, **β**), 4.88 (dt, *J* = 13.2, 3.6 Hz, 1H, **β**), 4.61 (d, *J* = 12.3 Hz, 1.4H, **α**), 4.40 – 4.32 (m, 2H, **β**), 4.26 (q, *J* = 6.8 Hz, 4.8H), 4.19 (d, *J* = 9.5 Hz, 1H, **β**), 4.15 (dd, *J* = 12.3, 3.4 Hz, 1.4H, **α**), 4.09 (d, *J* = 9.8 Hz, 1.4H, **α**), 3.74 (dd, *J* = 15.5, 6.7 Hz, 1H, **β**), 3.46 (dd, *J* = 15.6, 6.7 Hz, 1.4H, **α**), 3.29 (dd, *J* = 15.4, 7.1 Hz, 2.4H), 2.37 (dd, *J* = 12.5, 4.6 Hz, 1H, **β**), 2.20 – 2.16 (m, 1.4H, **α**), 2.10 (s, 3H), 2.09 (s, 3H), 2.07 (s, 4.2H), 2.06 (s, 4.2H), 2.07 – 2.00 (m, 2.4H), 2.00 (s, 3H), 1.99 (s, 4.2H), 1.98 (s, 3H), 1.97 (s, 4.2H), 1.59 – 1.52 (m, 4.8H), 1.36 – 1.24 (m, 31.2H), 0.88 (t, *J* = 6.9 Hz, 7.2H).

^{13}C NMR (151 MHz, $CDCl_3$): δ = 170.9, 170.6, 170.6, 170.6, 170.1, 170.1, 169.9, 169.8, 168.0 (**β**, C1,

${}^3J_{C\text{-}1/H\text{-}3ax}$ = 6.1 Hz, 151 MHz), 167.5 (**α**, C1, ${}^3J_{C\text{-}1/H\text{-}3ax}$ = 0 Hz, 151 MHz), 99.5, 98.8, 70.8, 68.3, 68.1, 67.9, 67.3, 66.7, 64.9, 64.6, 64.3, 64.2, 62.6, 62.2, 62.1, 62.0, 32.6, 32.3, 32.0, 29.7, 29.7, 29.6, 29.4, 29.4, 26.3, 26.1, 22.8, 22.8, 20.9, 20.9, 20.9, 20.9, 20.8, 20.8, 20.8, 20.8, 14.3, 14.3, 14.2, 14.2.
HRMS (ESI) m/z Calcd for $C_{26}H_{42}O_{12}Na$ $[M + Na]^+$ 569.2568, found: 569.2572.

Ethyl [3-(carbobenzoxyamino)-1-propyl 4,5,7,8-tetra-*O*-acetyl-3-deoxy-β-D-manno-oct-2-ulopyranosid]onate (36bβ)

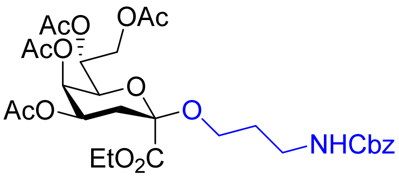

36bβ

GP1: 35.3 mg, 94% yield from **18**: a colorless syrup. (Petroleum ether/ethyl acetate = 2:1, v/v)
36bβ: ^{1}H NMR (600 MHz, $CDCl_3$): δ = 7.37 – 7.32 (m, 4H), 7.32 – 7.29 (m, 1H), 5.29 – 5.27 (m, 1H), 5.22 (t, *J* = 5.1 Hz, 1H), 5.17 (ddd, *J* = 9.6, 4.3, 2.4 Hz, 1H), 5.13 – 5.07 (m, 2H), 4.89 (ddd, *J* = 13.2, 4.6, 3.0 Hz, 1H), 4.34 (qd, *J* = 12.4, 3.3 Hz, 2H), 4.24 (ddd, *J* = 14.2, 7.1, 2.7 Hz, 2H), 4.20 (d, *J* = 8.8 Hz, 1H), 3.85 (dt, *J* = 9.7, 5.7 Hz, 1H), 3.43 (dt, *J* = 9.8, 5.8 Hz, 1H), 3.35 – 3.27 (m, 2H), 2.34 (dd, *J* = 12.5, 4.5 Hz, 1H), 2.11 – 2.08 (m, 4H), 2.07 (s, 3H), 2.01 (s, 3H), 1.99 (s, 3H), 1.81 – 1.75 (m, 2H), 1.30 (t, *J* = 7.1 Hz, 3H).
^{13}C NMR (101 MHz, $CDCl_3$): δ = 170.9, 170.6, 170.0, 169.9, 167.8 (C1, ${}^3J_{C\text{-}1/H\text{-}3ax}$ = 5.4 Hz, 101 MHz), 156.6, 136.9, 128.6, 128.3, 128.2, 99.4, 77.4, 70.9, 68.2, 67.2, 66.7, 64.1, 62.8, 62.5, 62.4, 38.7, 32.4, 29.4, 21.0, 20.9, 20.9, 20.8, 14.3.
HRMS (ESI) m/z Calcd for $C_{29}H_{40}NO_{14}$ $[M + H]^+$ 626.2443, found: 626.2437.

Ethyl (3-chloro-1-propyl 4,5,7,8-tetra-*O*-acetyl-3-deoxy-β-D-manno-oct-2-ulopyranosid)onate (36cβ)

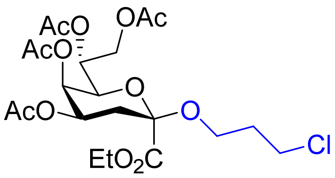

36cβ

GP1: 28.3 mg, 93% yield from **18**: a colorless oil. (Petroleum ether/ethyl acetate = 4:1, v/v)
36cβ: ^{1}H NMR (600 MHz, $CDCl_3$): δ = 5.29 – 5.26 (m, 1H), 5.17 (ddd, *J* = 9.5, 4.0, 2.8 Hz, 1H), 4.88 (ddd, *J* = 13.2, 4.6, 3.0 Hz, 1H), 4.40 – 4.34 (m, 2H), 4.27 (dtt, *J* = 10.8, 7.3, 3.6 Hz, 2H), 4.21 (dd, *J*

= 9.6, 1.3 Hz, 1H), 3.90 – 3.85 (m, 1H), 3.63 – 3.60 (m, 2H), 3.51 (ddd, J = 9.7, 7.6, 4.4 Hz, 1H), 2.36 (dd, J = 12.5, 4.6 Hz, 1H), 2.10 (s, 3H), 2.10 (s, 3H), 2.09 – 2.05 (m, 1H), 2.00 (s, 4H), 1.98 (s, 4H), 1.33 (t, J = 7.1 Hz, 3H).

^{13}C NMR (151 MHz, $CDCl_3$): δ = 170.9, 170.6, 170.0, 169.9, 167.8 (C1, $^3J_{C\text{-}1/H\text{-}3ax}$ = 6.1 Hz, 151 MHz), 99.5, 70.9, 68.2, 67.2, 64.2, 62.5, 62.3, 61.1, 41.6, 32.7, 32.4, 20.9, 20.9, 20.8, 20.8, 14.3.

HRMS (ESI) m/z Calcd for $C_{21}H_{31}O_{12}ClNa$ $[M + Na]^+$ 533.1396, found: 533.1390.

Ethyl (2-azidoethyl 4,5,7,8-tetra-*O*-acetyl-3-deoxy-β-D-manno-oct-2-ulopyranosid)onate (36dβ)

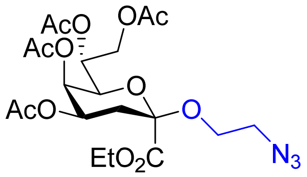

36dβ

GP1: 28.4 mg, 94% yield from **18**: a colorless oil. (Petroleum ether/ethyl acetate = 4:1, v/v)

30dβ: ^{1}H NMR (600 MHz, $CDCl_3$): δ = 5.28 (s, 1H), 5.18 (ddd, J = 9.6, 4.3, 2.0 Hz, 1H), 4.91 (ddd, J = 13.1, 4.4, 3.2 Hz, 1H), 4.41 (dd, J = 12.4, 4.5 Hz, 1H), 4.32 (dd, J = 12.5, 2.0 Hz, 1H), 4.28 (q, J = 7.2 Hz, 2H), 4.20 (d, J = 9.6 Hz, 1H), 3.97 (ddd, J = 10.3, 5.7, 3.2 Hz, 1H), 3.57 (ddd, J = 10.6, 7.4, 3.2 Hz, 1H), 3.39 (ddd, J = 13.1, 7.4, 3.2 Hz, 1H), 3.30 (ddd, J = 13.3, 5.6, 3.2 Hz, 1H), 2.42 (dd, J = 12.6, 4.6 Hz, 1H), 2.14 (t, J = 12.9 Hz, 1H), 2.11 (s, 3H), 2.09 (s, 3H), 2.01 (s, 3H), 1.98 (s, 3H), 1.34 (t, J = 7.2 Hz, 3H).

^{13}C NMR (151 MHz, $CDCl_3$): δ = 170.9, 170.6, 170.0, 169.9, 167.5 (C1, $^3J_{C\text{-}1/H\text{-}3ax}$ = 6.1 Hz, 151 MHz), 99.4, 71.0, 68.2, 67.1, 64.1, 63.9, 62.4, 62.4, 50.6, 32.4, 21.0, 20.9, 20.8, 20.8, 14.3.

HRMS (ESI) m/z Calcd for $C_{20}H_{29}N_3O_{12}Na$ $[M + Na]^+$ 526.1643, found: 526.1640.

Ethyl [methyl-*N*-(*tert*-butoxycarbonyl)-L-serinate 4,5,7,8-tetra-*O*-acetyl-3-deoxy-β-D-manno-oct-2-ulopyranosid]onate (36eβ)

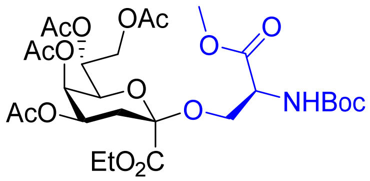

36eβ

GP1: 35.8 mg, 94% yield from **18**: a colorless syrup. (Petroleum ether/ethyl acetate = 1:1, v/v)

36eβ: ^{1}H NMR (600 MHz, $CDCl_3$): δ = 5.35 (d, J = 8.8 Hz, 1H), 5.26 (s, 1H), 5.12 (dt, J = 9.4, 3.2 Hz,

1H), 4.88 (dd, J = 9.7, 3.3 Hz, 1H), 4.41 (d, J = 8.8 Hz, 1H), 4.35 (d, J = 2.7 Hz, 2H), 4.25 (dd, J = 14.3, 7.1 Hz, 2H), 4.20 (dd, J = 13.5, 6.0 Hz, 2H), 3.77 (s, 3H), 3.59 (dd, J = 9.9, 2.7 Hz, 1H), 2.32 (dd, J = 12.6, 4.4 Hz, 1H), 2.09 (s, 3H), 2.07 (s, 3H), 2.07 (t, J = 9.2 Hz, 1H), 1.99 (s, 3H), 1.96 (s, 3H), 1.44 (s, 9H), 1.33 (t, J = 7.2 Hz, 3H).

^{13}C NMR (101 MHz, $CDCl_3$): δ = 170.8, 170.7, 170.5, 170.0, 169.9, 167.4 (C1, $^3J_{C-1/H-3ax}$ = 5.8 Hz, 101 MHz), 155.4, 99.1, 80.2, 71.0, 68.0, 67.0, 65.1, 64.0, 62.5, 62.3, 60.5, 53.6, 52.6, 32.2, 28.4, 20.9, 20.8, 20.8, 20.8, 14.3, 14.2.

HRMS (ESI) m/z Calcd for $C_{27}H_{42}NO_{16}$ $[M + H]^+$ 636.2498, found: 636.2491.

Ethyl (6-hydroxyl-hexyl 4,5,7,8-tetra-*O*-acetyl-3-deoxy-β-D-manno-oct-2-ulopyranosid)onate (36fβ)

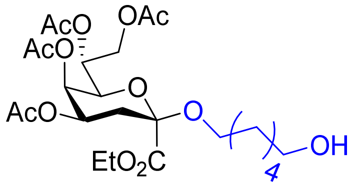

36fβ

GP1: 25.0 mg, 78% yield from **18** to generate **36fβ**: a pale-yellow oil (Petroleum ether/ethyl acetate = 1:1, v/v); while 6.0 mg, 21% yield from **18** to generate **36uβ**: a colorless syrup (Petroleum ether/ethyl acetate = 3:1, v/v).

36fβ: ^{1}H NMR (600 MHz, $CDCl_3$): δ = 5.28 (s, 1H), 5.18 (ddd, J = 9.6, 4.1, 3.0 Hz, 1H), 4.92 – 4.85 (m, 1H), 4.37 (t, J = 2.8 Hz, 2H), 4.27 (qd, J = 7.1, 1.6 Hz, 2H), 4.19 (dd, J = 9.6, 1.2 Hz, 1H), 3.75 (dq, J = 9.1, 6.4 Hz, 1H), 3.64 (t, J = 6.5 Hz, 2H), 3.31 (ddd, J = 11.1, 9.4, 5.6 Hz, 1H), 2.37 (dd, J = 12.2, 4.2 Hz, 1H), 2.11 (s, 3H), 2.10 (s, 3H), 2.09 – 2.07 (m, 1H), 2.01 (s, 3H), 1.98 (s, 3H), 1.60 – 1.54 (m, 4H), 1.39 (ddd, J = 17.5, 8.6, 5.2 Hz, 4H), 1.33 (t, J = 7.1 Hz, 3H).

^{13}C NMR (151 MHz, $CDCl_3$): δ = 171.0, 170.6, 170.1, 170.0, 168.0 (C1, $^3J_{C-1/H-3ax}$ = 6.2 Hz, 151 MHz), 99.5, 70.8, 68.3, 67.3, 64.7, 64.3, 63.0, 62.1, 32.8, 32.6, 29.6, 25.8, 25.5, 21.0, 20.9, 20.8, 20.8, 14.4.

HRMS (ESI) m/z Calcd for $C_{24}H_{38}O_{13}Na$ $[M + Na]^+$ 557.2204, found: 557.2210.

Ethyl (3-butynyl 4,5,7,8-tetra-*O*-acetyl-3-deoxy-β-D-manno-oct-2-ulopyranosid)onate (36gβ)

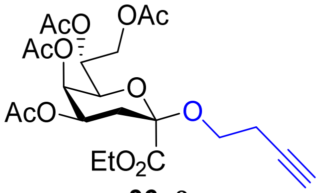

36gβ

GP1: 28.9 mg, 99% yield from **18**: a colorless oil. (Petroleum ether/ethyl acetate = 4:1, v/v)

36gβ: ^{1}H NMR (600 MHz, $CDCl_3$): δ = 5.28 (s, 1H), 5.17 (ddd, *J* = 9.4, 4.2, 2.4 Hz, 1H), 4.89 (ddd, *J* = 13.1, 4.2, 3.3 Hz, 1H), 4.36 (qd, *J* = 12.3, 3.3 Hz, 2H), 4.27 (q, *J* = 7.1 Hz, 2H), 4.18 (d, *J* = 9.4 Hz, 1H), 3.86 (dt, *J* = 9.0, 6.9 Hz, 1H), 3.50 (dt, *J* = 9.1, 7.3 Hz, 1H), 2.49 – 2.44 (m, 2H), 2.39 (dd, *J* = 12.5, 4.6 Hz, 1H), 2.10 (s, 3H), 2.12 – 2.08 (m, 1H), 2.10 (s, 3H), 2.00 (s, 3H), 1.98 (s, 3H), 1.99 – 1.96 (m, 1H), 1.33 (t, *J* = 7.1 Hz, 3H).

^{13}C NMR (151 MHz, $CDCl_3$): δ = 170.9, 170.6, 170.0, 169.9, 167.7 (C1, $^3J_{C\text{-}1/H\text{-}3ax}$ = 6.2 Hz, 151 MHz), 99.5, 80.7, 71.0, 69.7, 68.2, 67.2, 64.2, 62.9, 62.5, 62.3, 32.4, 20.9, 20.9, 20.8, 20.1, 14.3.

HRMS (ESI) m/z Calcd for $C_{22}H_{30}O_{12}Na$ $[M + Na]^+$ 509.1629, found: 509.1633.

Ethyl (5-chloro-pentyl 4,5,7,8-tetra-*O*-acetyl-3-deoxy-β-D-manno-oct-2-ulopyranosid)onate (36hβ)

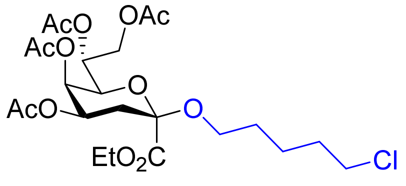

36hβ

GP1: 30.6 mg, 95% yield from **18**: a colorless oil. (Petroleum ether/ethyl acetate = 4:1, v/v)

36hβ: ^{1}H NMR (600 MHz, $CDCl_3$): δ = 5.28 – 5.26 (m, 1H), 5.17 (dt, *J* = 9.5, 3.4 Hz, 1H), 4.87 (ddd, *J* = 13.2, 4.6, 3.0 Hz, 1H), 4.35 (d, *J* = 3.5 Hz, 2H), 4.26 (qd, *J* = 7.1, 1.4 Hz, 2H), 4.19 (dd, *J* = 9.6, 1.4 Hz, 1H), 3.76 (dt, *J* = 9.3, 6.2 Hz, 1H), 3.53 (t, *J* = 6.7 Hz, 2H), 3.31 (dt, *J* = 9.3, 6.4 Hz, 1H), 2.36 (ddd, *J* = 12.4, 4.5, 0.7 Hz, 1H), 2.10 (s, 3H), 2.09 (s, 3H), 2.09 – 2.06 (m, 1H), 2.00 (s, 3H), 1.98 (s, 3H), 1.81 – 1.75 (m, 2H), 1.61 – 1.55 (m, 2H), 1.53 – 1.44 (m, 2H), 1.32 (t, *J* = 7.2 Hz, 3H).

^{13}C NMR (151 MHz, $CDCl_3$): δ = 170.9, 170.6, 170.0, 169.9, 167.9 (C1, $^3J_{C\text{-}1/H\text{-}3ax}$ = 6.2 Hz, 151 MHz), 99.5, 70.8, 68.2, 67.3, 64.4, 64.2, 62.6, 62.1, 45.0, 32.6, 32.4, 28.9, 23.6, 20.9, 20.9, 20.8, 20.8, 14.3.

HRMS (ESI) m/z Calcd for $C_{23}H_{35}O_{12}ClNa$ $[M + Na]^+$ 561.1709, found: 561.1708.

Ethyl (benzyl 4,5,7,8-tetra-*O*-acetyl-3-deoxy-β-D-manno-oct-2-ulopyranosid)onate (36iβ)

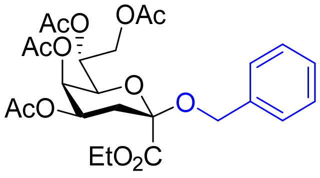

36iβ

GP1: 31.2 mg, 99% yield from **18**: a colorless oil. (Petroleum ether/ethyl acetate = 4:1, v/v)

36iβ: ^{1}H NMR (400 MHz, $CDCl_3$): δ = 7.35 – 7.27 (m, 5H), 5.32 – 5.29 (m, 1H), 5.21 (ddd, *J* = 9.6, 4.4, 2.4 Hz, 1H), 4.92 (ddd, *J* = 13.2, 4.6, 3.0 Hz, 1H), 4.81 (d, *J* = 11.6 Hz, 1H), 4.48 – 4.36 (m, 3H), 4.28 – 4.24 (m, 1H), 4.24 – 4.14 (m, 2H), 2.45 (ddd, *J* = 12.5, 4.6, 0.9 Hz, 1H), 2.18 (t, *J* = 12.9 Hz, 1H), 2.12 (s, 3H), 2.10 (s, 3H), 2.01 (s, 3H), 1.99 (s, 3H), 1.29 (t, *J* = 7.1 Hz, 3H).

^{13}C NMR (101 MHz, $CDCl_3$): δ = 170.9, 170.6, 170.1, 169.9, 167.9 (C1, $^3J_{C\text{-}1/H\text{-}3ax}$ = 5.4 Hz, 101 MHz), 137.2, 128.5, 128.0, 128.0, 99.4, 70.9, 68.2, 67.2, 66.7, 64.2, 62.5, 62.2, 32.6, 21.0, 20.9, 20.9, 20.8, 14.3.

HRMS (ESI) m/z Calcd for $C_{25}H_{32}O_{12}Na$ $[M + Na]^+$ 547.1785, found: 547.1785.

Ethyl (benzyl 4,5,7,8-tetra-*O*-acetyl-3-deoxy-α-D-manno-oct-2-ulopyranosid)onate (36iα)

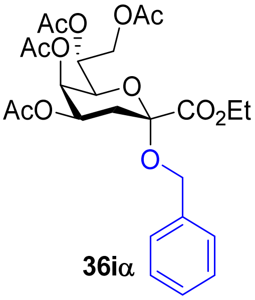

36iα

GP2: 14.1 mg, 44% yield from **18** to generate **36iβ**: a colorless oil (Petroleum ether/ethyl acetate = 4:1, v/v); while 13.6 mg, 44% yield from **18** to generate **36iα**: a colorless oil (Petroleum ether/ethyl acetate = 4:1, v/v).

36iα: ^{1}H NMR (600 MHz, $CDCl_3$): δ = 7.39 – 7.30 (m, 5H), 5.40 (t, *J* = 7.4 Hz, 2H), 5.26 (dd, *J* = 9.9, 2.5 Hz, 1H), 4.64 (dd, *J* = 12.3, 2.1 Hz, 1H), 4.57 (d, *J* = 11.6 Hz, 1H), 4.45 (d, *J* = 11.5 Hz, 1H), 4.27 – 4.19 (m, 4H), 2.29 (dd, *J* = 12.7, 4.5 Hz, 1H), 2.11 – 2.09 (m, 4H), 1.99 (s, 3H), 1.98 (s, 3H), 1.91 (s, 3H), 1.30 (t, *J* = 7.1 Hz, 3H).

^{13}C NMR (151 MHz, $CDCl_3$): δ = 170.6, 170.6, 170.1, 169.8, 167.2 (C1, $^3J_{C\text{-}1/H\text{-}3ax}$ = 0 Hz, 151 MHz), 136.9, 128.7, 128.1, 127.4, 98.9, 68.4, 67.8, 66.6, 65.8, 64.5, 62.2, 62.1, 32.2, 20.9, 20.8, 20.7, 14.2.

HRMS (ESI) m/z Calcd for $C_{25}H_{32}O_{12}Na$ $[M + Na]^+$ 547.1785, found: 547.1790.

Ethyl (3,5-di-tert-butylbenzyl 4,5,7,8-tetra-*O*-acetyl-3-deoxy-β-D-manno-oct-2-ulopyranosid)onate (36jβ)

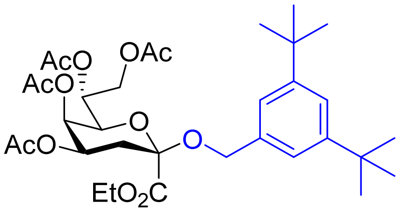

36jβ

GP1: 38.0 mg, 99% yield from **18**: a pale-yellow oil. (Petroleum ether/ethyl acetate = 6:1, v/v)

36jβ: ^{1}H NMR (600 MHz, $CDCl_3$): δ = 7.35 (t, *J* = 1.8 Hz, 1H), 7.17 (d, *J* = 1.8 Hz, 2H), 5.32 – 5.30 (m, 1H), 5.22 (ddd, *J* = 9.6, 4.4, 2.3 Hz, 1H), 4.94 (ddd, *J* = 13.2, 4.6, 3.0 Hz, 1H), 4.79 (d, *J* = 11.5 Hz, 1H), 4.47 (d, *J* = 11.5 Hz, 1H), 4.44 (dd, *J* = 12.3, 4.5 Hz, 1H), 4.38 (dd, *J* = 12.3, 2.3 Hz, 1H), 4.27 (dd, *J* = 9.6, 1.4 Hz, 1H), 4.25 – 4.17 (m, 2H), 2.50 – 2.44 (m, 1H), 2.20 (t, *J* = 12.9 Hz, 1H), 2.12 (s, 3H), 2.10 (s, 3H), 2.02 (s, 3H), 1.99 (s, 3H), 1.32 (s, 18H), 1.30 (t, *J* = 7.2 Hz, 3H).

^{13}C NMR (151 MHz, $CDCl_3$): δ = 170.8, 170.6, 170.0, 169.9, 168.0 (C1, $^3J_{C\text{-}1/H\text{-}3ax}$ = 6.1 Hz, 151 MHz), 150.8, 136.3, 122.3, 122.1, 99.5, 70.9, 68.3, 67.4, 67.3, 64.3, 62.5, 62.1, 34.9, 32.7, 31.6, 20.9, 20.9, 20.8, 20.8, 14.3.

HRMS (ESI) m/z Calcd for $C_{33}H_{48}O_{12}Na$ $[M + Na]^+$ 659.3037, found: 659.3033.

Ethyl (L-menthyl 4,5,7,8-tetra-*O*-acetyl-3-deoxy-β-D-manno-oct-2-ulopyranosid)onate (36kβ)

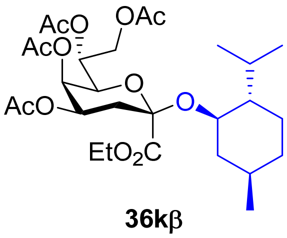

36kβ

GP1: 34.0 mg, 99% yield from **18**: a pale-yellow syrup. (Petroleum ether/ethyl acetate = 6:1, v/v)

36kβ: ^{1}H NMR (400 MHz, $CDCl_3$): δ = 5.26 (s, 1H), 5.14 – 5.03 (m, 1H), 4.90 – 4.80 (m, 1H), 4.47 (d, *J* = 12.2 Hz, 1H), 4.34 – 4.17 (m, 3H), 3.98 (d, *J* = 9.5 Hz, 1H), 3.83 (td, *J* = 10.5, 4.0 Hz, 1H), 2.42 (dd, *J* = 12.4, 4.6 Hz, 1H), 2.17 (dd, *J* = 13.3, 6.4 Hz, 1H), 2.11 – 2.04 (m, 7H), 1.98 (s, 3H), 1.98 (s, 3H), 1.71 – 1.60 (m, 2H), 1.33 (t, *J* = 7.0 Hz, 3H), 1.28 – 1.12 (m, 2H), 0.98 – 0.82 (m, 10H), 0.76 (d, *J* = 6.8 Hz, 3H).

^{13}C NMR (101 MHz, $CDCl_3$): δ = 170.8, 170.6, 170.1, 169.9, 168.7 (C1, $^3J_{C\text{-}1/H\text{-}3ax}$ = 5.7 Hz, 101 MHz), 98.0, 74.1, 70.8, 68.1, 67.4, 64.2, 62.7, 62.1, 48.5, 42.2, 34.3, 32.9, 31.6, 25.3, 23.4, 22.4, 21.3, 20.9, 20.9, 20.8, 20.7, 16.5, 14.2.

HRMS (ESI) m/z Calcd for $C_{28}H_{44}O_{12}Na$ $[M + Na]^+$ 595.2724, found: 595.2720.

Ethyl (L-menthyl 4,5,7,8-tetra-*O*-acetyl-3-deoxy-α-D-manno-oct-2-ulopyranosid)onate (36kα)

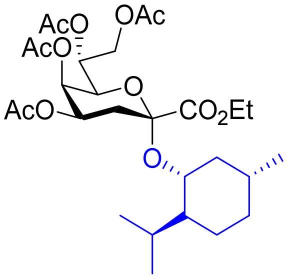

36kα

GP2: 5.2 mg, 15% yield from **18** to generate **36kβ**: a colorless oil (Petroleum ether/ethyl acetate = 6:1, v/v); while 25.8 mg, 75% yield from **18** to generate **36kα**: a colorless oil (Petroleum ether/ethyl acetate = 6:1, v/v).

36kα: ^{1}H NMR (600 MHz, $CDCl_3$): δ = 5.39 – 5.32 (m, 2H), 5.24 (ddd, *J* = 9.4, 5.2, 2.6 Hz, 1H), 4.70 (dd, *J* = 12.1, 2.4 Hz, 1H), 4.41 – 4.34 (m, 1H), 4.31 (d, *J* = 9.6 Hz, 1H), 4.19 (dq, *J* = 11.0, 7.1 Hz, 1H), 4.03 (dd, *J* = 12.1, 5.3 Hz, 1H), 3.31 (td, *J* = 10.5, 4.2 Hz, 1H), 2.25 (dd, *J* = 11.8, 4.2 Hz, 1H), 2.16 – 2.10 (m, 1H), 2.06 (s, 3H), 2.06 (s, 3H), 2.01 – 1.95 (m, 7H), 1.66 – 1.58 (m, 2H), 1.33 (t, *J* = 7.1 Hz, 3H), 1.30 – 1.20 (m, 2H), 1.01 – 0.91 (m, 2H), 0.88 (dd, *J* = 6.7, 2.8 Hz, 6H), 0.86 – 0.75 (m, 2H), 0.71 (d, *J* = 6.9 Hz, 3H).

^{13}C NMR (151 MHz, $CDCl_3$): δ = 170.7, 170.6, 170.3, 169.8, 167.0 (C1, $^3J_{C\text{-}1/H\text{-}3ax}$ = 0 Hz, 151 MHz), 100.1, 78.5, 68.7, 68.4, 66.9, 64.9, 62.5, 61.9, 48.3, 42.2, 34.4, 33.7, 31.4, 25.8, 23.5, 22.3, 21.0, 21.0, 21.0, 20.8, 20.8, 16.3, 14.2.

HRMS (ESI) m/z Calcd for $C_{28}H_{44}O_{12}Na$ $[M + Na]^+$ 595.2724, found: 595.2730.

Ethyl (adamantan-2-yl 4,5,7,8-tetra-*O*-acetyl-3-deoxy-β-D-manno-oct-2-ulopyranosid)onate (36lβ)[25]

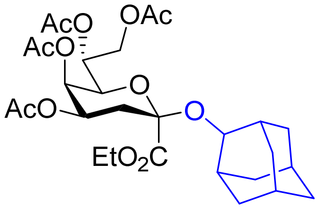

36lβ

GP1: 28.5 mg, 84% yield from **18**: a pale-yellow syrup. (Petroleum ether/ethyl acetate = 6:1, v/v)

36lβ: ^{1}H NMR (600 MHz, $CDCl_3$): δ = 5.26 (s, 1H), 5.13 (ddd, *J* = 9.4, 4.6, 2.2 Hz, 1H), 4.90 – 4.84 (m, 1H), 4.37 (dd, *J* = 12.2, 2.0 Hz, 1H), 4.29 (dd, *J* = 12.3, 4.7 Hz, 1H), 4.23 (dddd, *J* = 17.9, 10.8, 7.2, 3.6 Hz, 2H), 4.13 (d, *J* = 9.6 Hz, 1H), 3.91 (s, 1H), 2.40 (dd, *J* = 12.5, 4.5 Hz, 1H), 2.15 – 2.08 (m, 9H), 2.00 (s, 3H), 1.98 (s, 3H), 1.82 – 1.75 (m, 4H), 1.70 (d, *J* = 14.5 Hz, 4H), 1.62 (d, *J* = 11.5 Hz, 2H), 1.50 – 1.44 (m, 2H), 1.31 (t, *J* = 7.1 Hz, 3H). The spectroscopic data coincided with the previous report.[25]

Ethyl (adamantan-2-yl 4,5,7,8-tetra-*O*-acetyl-3-deoxy-α-D-manno-oct-2-ulopyranosid)onate (36lα)[25]

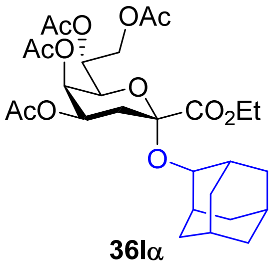

36lα

GP2: 22.9 mg, 67% yield from **18** to generate the mixture of **36lβ** and **36lα** (1:4): a pale-yellow syrup. (Petroleum ether/ethyl acetate = 6:1, v/v)

The mixture of **36lβ** and **36lα** (1:4)**:** ^{1}H NMR (600 MHz, CDCl3): δ = 5.39 – 5.33 (m, 2H, **α**), 5.26 (s, 0.25H, **β**), 5.18 (dt, *J* = 9.7, 2.9 Hz, 1H, **α**), 5.13 (ddd, *J* = 9.4, 4.5, 2.1 Hz, 0.25H, **β**), 4.90 – 4.84 (m, 0.25H, **β**), 4.68 (dd, *J* = 12.3, 2.6 Hz, 1H, **α**), 4.37 (dd, *J* = 12.2, 1.9 Hz, 0.25H, **β**), 4.31 – 4.08 (m, 5H), 3.92 (s, 0.25H, **β**), 3.89 (s, 1H, **α**), 2.40 (dd, *J* = 12.4, 4.6 Hz, 0.25H, **β**), 2.33 (dd, *J* = 12.7, 4.7 Hz, 1H, **α**), 2.13 – 1.94 (m, 18.75H), 1.84 – 1.41 (m, 15H), 1.29 (t, *J* = 7.3 Hz, 3.75H).

The spectroscopic data coincided with the previous report.[25]

Ethyl (epiandrosteroneyl 4,5,7,8-tetra-*O*-acetyl-3-deoxy-β-D-manno-oct-2-ulopyranosid)onate (36mβ)

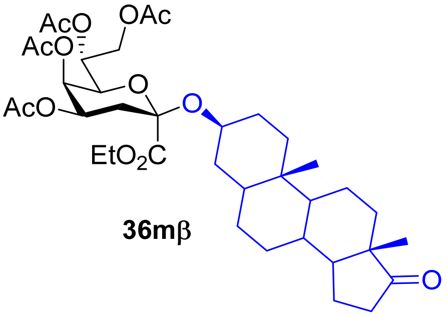

36mβ

GP1: 38.2 mg, 90% yield from **18**: a pale-yellow syrup. (Petroleum ether/ethyl acetate = 4:1, v/v)

36mβ: ^{1}H NMR (600 MHz, CDCl3): δ = 5.25 (d, *J* = 1.0 Hz, 1H), 5.15 (ddd, *J* = 9.5, 4.2, 2.5 Hz, 1H), 4.87 – 4.82 (m, 1H), 4.39 – 4.31 (m, 2H), 4.29 – 4.21 (m, 2H), 4.14 (d, *J* = 9.6 Hz, 1H), 3.76 – 3.64 (m, 1H), 2.42 (dd, *J* = 19.3, 8.8 Hz, 1H), 2.36 (dd, *J* = 12.4, 4.5 Hz, 1H), 2.10 (s, 3H), 2.09 – 2.07 (m, 4H), 2.04 – 2.02 (m, 1H), 1.99 (s, 3H), 1.98 (s, 3H), 1.94 – 1.85 (m, 2H), 1.79 – 1.74 (m, 2H), 1.69 (dt, *J* = 13.2, 3.3 Hz, 1H), 1.64 (dd, *J* = 13.3, 3.1 Hz, 1H), 1.56 – 1.44 (m, 3H), 1.32 (t, *J* = 7.2 Hz, 3H), 1.29 – 1.19 (m, 6H), 1.09 – 1.04 (m, 1H), 0.97 (dt, *J* = 11.1, 9.1 Hz, 2H), 0.86 (dd, *J* = 13.3, 6.0 Hz, 1H), 0.84 (s, 3H), 0.80 (s, 3H), 0.68 (td, *J* = 11.8, 3.8 Hz, 1H).

^{13}C NMR (151 MHz, CDCl3): δ = 221.5, 170.8, 170.7, 170.1, 170.0, 168.4 (C1, $^3J_{C\text{-}1/H\text{-}3ax}$ = 6.3 Hz, 151 MHz), 99.7, 75.3, 70.7, 68.3, 67.3, 64.2, 62.6, 62.0, 54.3, 51.5, 47.9, 45.0, 37.1, 36.0, 35.8, 35.7, 35.1, 33.0, 31.6, 30.9, 30.6, 28.6, 21.9, 21.0, 20.9, 20.8, 20.6, 14.3, 13.9, 12.3.

HRMS (ESI) m/z Calcd for $C_{37}H_{54}O_{13}Na$ [M + Na]$^+$ 729.3456, found: 729.3454.

Ethyl (dihydrocholesteryl 4,5,7,8-tetra-*O*-acetyl-3-deoxy-β-D-manno-oct-2-ulopyranosid)onate (36nβ)

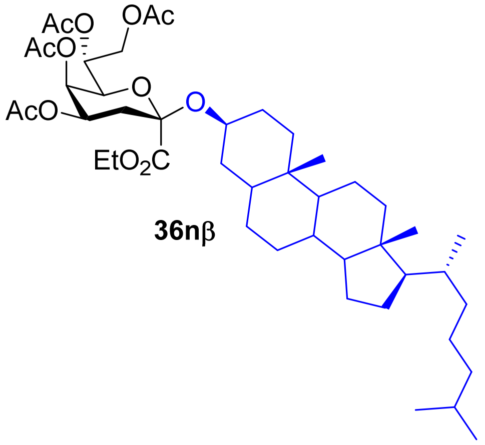

36nβ

GP1: 22.9 mg, 47% yield from **18**: a white amorphous solid. (Petroleum ether/ethyl acetate = 6:1, v/v)

36nβ: ^{1}H NMR (400 MHz, $CDCl_3$): δ = 5.26 (s, 1H), 5.15 (ddd, *J* = 9.5, 4.5, 2.4 Hz, 1H), 4.85 (ddd, *J* = 13.1, 4.5, 3.0 Hz, 1H), 4.36 (qd, *J* = 12.3, 3.4 Hz, 2H), 4.30 – 4.22 (m, 2H), 4.16 (dd, *J* = 9.5, 1.2 Hz, 1H), 3.68 (ddd, *J* = 15.7, 10.7, 5.4 Hz, 1H), 2.36 (dd, *J* = 12.4, 4.5 Hz, 1H), 2.11 (s, 3H), 2.09 (s, 3H), 2.08 – 2.02 (m, 2H), 2.00 (s, 3H), 1.98 (s, 3H), 1.33 (t, *J* = 7.1 Hz, 3H).

^{13}C NMR (101 MHz, $CDCl_3$): δ = 170.8, 170.7, 170.1, 170.0, 168.4 (C1, $^3J_{C\text{-}1/H\text{-}3ax}$ = 5.9 Hz, 101 MHz), 99.7, 77.4, 75.6, 70.7, 68.3, 67.3, 64.2, 62.6, 62.0, 56.5, 56.4, 54.2, 45.1, 42.7, 40.1, 39.7, 37.2, 36.3, 36.0, 35.6, 35.5, 33.0, 32.1, 30.6, 29.0, 28.4, 28.2, 24.4, 24.0, 23.0, 22.7, 21.3, 21.0, 20.9, 20.9, 18.8, 14.3, 12.3, 12.2.

HRMS (ESI) m/z Calcd for $C_{45}H_{73}O_{12}$ [M + H]$^+$ 805.5096, found: 805.5096.

Ethyl (adamantan-1-yl 4,5,7,8-tetra-*O*-acetyl-3-deoxy-β-D-manno-oct-2-ulopyranosid)onate (36oβ)[25]

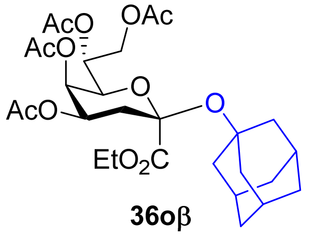

36oβ

GP1: 21.8 mg, 64% yield from **18** to generate **36oβ**: a colorless oil (Petroleum ether/ethyl acetate = 6:1, v/v); while 7.8 mg, 23% yield from **18** to generate **36oα**: a colorless oil (Petroleum ether/ethyl acetate = 5:1, v/v).

36oβ: ^{1}H NMR (600 MHz, $CDCl_3$): δ = 5.26 (d, J = 1.0 Hz, 1H), 5.17 (ddd, J = 7.0, 4.6, 1.5 Hz, 1H), 4.82 – 4.75 (m, 1H), 4.47 (t, J = 10.6 Hz, 2H), 4.31 (dd, J = 12.4, 5.2 Hz, 1H), 4.29 – 4.24 (m, 1H), 4.23 – 4.18 (m, 1H), 2.29 (dd, J = 12.5, 4.4 Hz, 1H), 2.13 – 2.09 (m, 6H), 2.09 (s, 4H), 2.00 (s, 3H), 1.96 (s, 3H), 1.88 (d, J = 2.2 Hz, 6H), 1.58 (s, 6H), 1.34 (t, J = 7.1 Hz, 3H).

^{13}C NMR (151 MHz, $CDCl_3$): δ = 170.8, 170.7, 170.7, 170.1, 170.0 (C1, $^{3}J_{C\text{-}1/H\text{-}3ax}$ = 5.8 Hz, 101 MHz), 98.6, 78.9, 71.2, 68.4, 67.0, 64.4, 63.1, 61.8, 43.6, 36.3, 35.3, 31.2, 20.9, 20.9, 20.9, 20.8, 14.2.

The spectroscopic data coincided with the previous report.[25]

Ethyl (adamantan-1-yl 4,5,7,8-tetra-*O*-acetyl-3-deoxy-α-D-manno-oct-2-ulopyranosid)onate (36oα)[3]

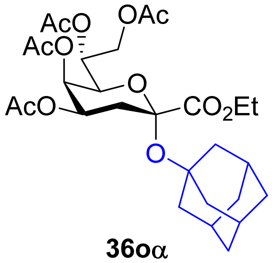

36oα

GP2: 2.4 mg, 7% yield from **18** to generate **36oβ**: a colorless oil (Petroleum ether/ethyl acetate = 6:1, v/v); while 31.4 mg, 92% yield from **18** to generate **36oα**: a colorless oil (Petroleum ether/ethyl acetate = 5:1, v/v).

36oα: ^{1}H NMR (400 MHz, $CDCl_3$): δ = 5.38 – 5.32 (m, 2H), 5.22 (dt, J = 9.3, 2.7 Hz, 1H), 4.66 (dd, J = 12.2, 2.2 Hz, 1H), 4.34 (d, J = 9.7 Hz, 1H), 4.32 – 4.18 (m, 2H), 4.14 (dd, J = 12.2, 3.5 Hz, 1H), 2.18 (dd, J = 12.3, 4.2 Hz, 1H), 2.11 – 2.05 (m, 9H), 1.99 – 1.94 (m, 6H), 1.86 (s, 6H), 1.58 (q, J = 12.4 Hz, 6H), 1.34 (t, J = 7.1 Hz, 3H).

^{13}C NMR (101 MHz, $CDCl_3$): δ = 170.6, 170.6, 170.2, 169.9, 169.7 (C1, $^{3}J_{C\text{-}1/H\text{-}3ax}$ = 0 Hz, 151 MHz), 97.4, 78.0, 68.7, 68.6, 66.8, 65.0, 61.9, 61.8, 42.8, 36.2, 35.0, 31.1, 21.0, 20.9, 20.8, 20.8, 14.1.

The spectroscopic data coincided with the previous report.[3]

Ethyl (4,5,7,8-tetra-*O*-acetyl-3-deoxy-β-D-manno-oct-2-ulopyranosyl)onate-(2→6)-methyl 2,3,4-tri-*O*-benzyl-α-D-glucopyranoside (36pβ)

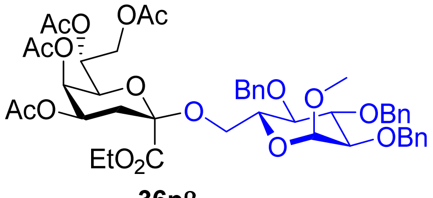

36pβ

GP1: 48.6 mg, 92% yield from **18**: a white amorphous solid. (Petroleum ether/ethyl acetate = 3:1, v/v)

36pβ: ^{1}H NMR (400 MHz, $CDCl_3$): δ = 7.39 – 7.27 (m, 15H), 5.26 (d, *J* = 1.0 Hz, 1H), 5.16 (ddd, *J* = 9.5, 4.1, 2.4 Hz, 1H), 4.96 (d, *J* = 10.7 Hz, 1H), 4.92 – 4.85 (m, 2H), 4.79 (dd, *J* = 11.4, 4.3 Hz, 2H), 4.68 (s, 1H), 4.66 – 4.59 (m, 2H), 4.32 (dd, *J* = 12.3, 2.3 Hz, 1H), 4.26 (dd, *J* = 12.3, 4.3 Hz, 1H), 4.18 (ddd, *J* = 14.5, 9.0, 5.5 Hz, 1H), 4.08 – 4.04 (m, 1H), 4.00 (dt, *J* = 12.8, 8.6 Hz, 3H), 3.78 (dd, *J* = 10.2, 3.7 Hz, 1H), 3.57 (dd, *J* = 10.4, 5.2 Hz, 1H), 3.54 – 3.49 (m, 1H), 3.49 – 3.41 (m, 1H), 3.39 (s, 3H), 2.40 (dd, *J* = 12.3, 4.6 Hz, 1H), 2.13 (dd, *J* = 11.7, 5.1 Hz, 1H), 2.07 (s, 6H), 1.98 (s, 3H), 1.98 (s, 3H), 1.19 (t, *J* = 7.2 Hz, 3H).

^{13}C NMR (101 MHz, $CDCl_3$): δ = 170.7, 170.6, 170.0, 169.8, 167.3 (C1, $^3J_{C\text{-}1/H\text{-}3ax}$ = 5.6 Hz, 151 MHz), 138.8, 138.5, 138.3, 128.6, 128.5, 128.5, 128.2, 128.1, 128.0, 127.8, 127.7, 127.6, 99.6, 98.0, 82.3, 80.0, 77.4, 75.9, 74.6, 73.5, 70.8, 69.6, 68.1, 67.3, 64.1, 63.5, 62.3, 62.2, 55.2, 32.3, 20.9, 20.9, 20.8, 20.8, 14.2.

HRMS (ESI) m/z Calcd for $C_{46}H_{57}O_{17}$ $[M + H]^+$ 881.3590, found: 881.3597.

Ethyl (4,5,7,8-tetra-*O*-acetyl-3-deoxy-α-D-manno-oct-2-ulopyranosyl)onate-(2→6)-methyl 2,3,4-tri-*O*-benzyl-α-D-glucopyranoside (36pα)[3]

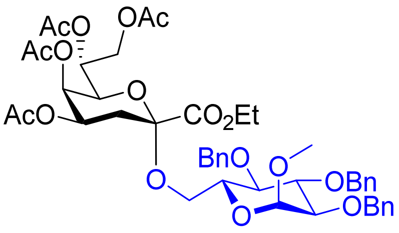

36pα

GP2: 37.0 mg, 70% yield from **18**: a white amorphous solid. (Petroleum ether/ethyl acetate = 3:1, v/v)

36pα: ^{1}H NMR (600 MHz, $CDCl_3$): δ = 7.37 – 7.26 (m, 15H), 5.34 – 5.29 (m, 2H), 5.18 (ddd, *J* = 9.4, 4.3, 2.4 Hz, 1H), 4.99 (d, *J* = 10.8 Hz, 1H), 4.92 (d, *J* = 11.2 Hz, 1H), 4.80 (dd, *J* = 11.4, 8.7 Hz, 1H), 4.67 (d, *J* = 12.1 Hz, 1H), 4.57 (d, *J* = 3.6 Hz, 1H), 4.54 – 4.49 (m, 2H), 4.20 – 4.11 (m, 3H), 4.08 (dd, *J* = 12.3, 4.4 Hz, 1H), 3.99 (t, *J* = 9.2 Hz, 1H), 3.86 – 3.79 (m, 1H), 3.66 (d, *J* = 9.9 Hz, 1H), 3.49 – 3.43 (m, 2H), 3.42 (s, 3H), 3.27 – 3.21 (m, 1H), 2.16 (dd, *J* = 12.5, 5.2 Hz, 1H), 2.09 – 2.02 (m, 4H), 1.98 (s, 3H), 1.96 (s, 3H), 1.88 (s, 3H), 1.21 (t, *J* = 7.1 Hz, 3H).

^{13}C NMR (151 MHz, $CDCl_3$): δ = 170.6, 170.5, 170.0, 169.8, 166.8 (C1, $^3J_{C\text{-}1/H\text{-}3ax}$ = 0 Hz, 151 MHz), 138.7, 138.2, 128.6, 128.6, 128.5, 128.2, 128.1, 127.9, 127.8, 127.8, 98.4, 97.8, 82.4, 79.9, 78.5, 75.9, 75.2, 73.4, 69.7, 68.4, 68.1, 66.5, 64.7, 63.6, 62.2, 62.0, 55.2, 32.0, 20.9, 20.8, 20.8, 14.2.

The spectroscopic data coincided with the previous report.[3]

Ethyl (4,5,7,8-tetra-*O*-acetyl-3-deoxy-β-D-manno-oct-2-ulopyranosyl)onate-(2→6)-methyl 2,3,4-tri-*O*-benzoyl-α-D-glucopyranoside (36qβ)

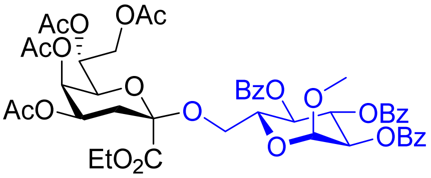

36qβ

GP1: 48.6 mg, 88% yield from **18**: a white amorphous solid. (Petroleum ether/ethyl acetate = 2:1, v/v)

36qβ: ^{1}H NMR (600 MHz, $CDCl_3$): δ = 7.99 – 7.95 (m, 2H), 7.94 – 7.91 (m, 2H), 7.88 – 7.84 (m, 2H), 7.50 (t, *J* = 7.4 Hz, 2H), 7.43 – 7.39 (m, 1H), 7.38 – 7.35 (m, 4H), 7.28 (t, *J* = 7.9 Hz, 2H), 6.11 (t, *J* = 9.7 Hz, 1H), 5.56 – 5.50 (m, 1H), 5.24 (dt, *J* = 6.7, 3.7 Hz, 3H), 5.11 (ddd, *J* = 9.6, 4.2, 2.3 Hz, 1H), 4.88 (ddd, *J* = 13.1, 4.6, 3.0 Hz, 1H), 4.29 – 4.23 (m, 2H), 4.19 (dd, *J* = 12.3, 4.3 Hz, 1H), 4.13 – 4.09 (m, 1H), 4.03 (dd, *J* = 9.6, 1.3 Hz, 1H), 4.00 (dd, *J* = 11.1, 2.3 Hz, 1H), 3.92 (dq, *J* = 10.7, 7.1 Hz, 1H), 3.67 (dd, *J* = 11.1, 5.8 Hz, 1H), 3.49 (s, 3H), 2.46 – 2.39 (m, 1H), 2.10 (t, *J* = 12.6 Hz, 1H), 2.07 (s, 3H), 2.03 (s, 3H), 1.97 (s, 3H), 1.96 (s, 3H), 1.17 (t, *J* = 7.2 Hz, 3H).

^{13}C NMR (101 MHz, $CDCl_3$): δ = 170.6, 170.6, 167.0, 169.8, 167.0, 166.0, 165.9, 165.2 (C1, $^3J_{C\text{-}1/H\text{-}3ax}$ = 6.3 Hz, 151 MHz), 133.5, 133.2, 130.0, 129.9, 129.8, 129.4, 129.2, 129.2, 128.5, 128.4, 99.5, 97.0, 77.4, 72.2, 70.8, 70.8, 69.3, 68.5, 68.1, 67.2, 64.1, 63.3, 62.2, 62.1, 55.6, 32.4, 20.9, 20.8, 20.8, 14.1.

HRMS (ESI) m/z Calcd for $C_{46}H_{50}O_{20}Na$ $[M + Na]^+$ 945.2787, found: 945.2782.

Ethyl (4,5,7,8-tetra-*O*-acetyl-3-deoxy-α-D-manno-oct-2-ulopyranosyl)onate-(2→6)-methyl 2,3,4-tri-*O*-benzoyl-α-D-glucopyranoside (36qα)

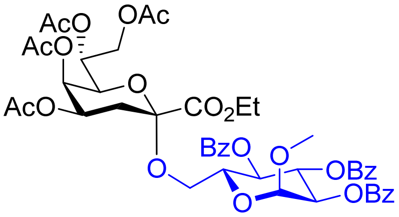

36qα

GP2: 10.0 mg, 18% yield from **18** to generate **36qβ**: a white amorphous solid (Petroleum ether/ethyl acetate = 2:1, v/v); while 39.8 mg, 72% yield from **18** to generate **36qα**: a colorless syrup (Petroleum ether/ethyl acetate = 3:1, v/v).

36qα: ^{1}H NMR (600 MHz, $CDCl_3$): δ = 7.97 (ddd, *J* = 13.1, 8.1, 1.0 Hz, 4H), 7.85 (dd, *J* = 8.2, 1.0 Hz, 2H), 7.53 – 7.50 (m, 2H), 7.42 (t, *J* = 7.5 Hz, 1H), 7.39 – 7.36 (m, 4H), 7.28 (t, *J* = 7.8 Hz, 2H), 6.16 (t, *J* = 9.8 Hz, 1H), 5.45 – 5.38 (m, 2H), 5.36 (d, *J* = 1.1 Hz, 1H), 5.26 – 5.21 (m, 2H), 5.19 (ddd, *J* = 9.4, 4.4, 2.5 Hz, 1H), 4.63 (dd, *J* = 12.3, 2.4 Hz, 1H), 4.28 – 4.25 (m, 1H), 4.20 (dd, *J* = 9.5, 1.3

Hz, 1H), 4.15 (dq, J = 10.8, 3.7 Hz, 2H), 4.04 (dd, J = 12.3, 4.5 Hz, 1H), 3.72 (dd, J = 10.8, 7.4 Hz, 1H), 3.62 (dd, J = 10.8, 2.0 Hz, 1H), 3.51 (s, 3H), 2.13 (dd, J = 12.7, 5.2 Hz, 1H), 2.08 (s, 3H), 2.08 – 2.06 (m, 4H), 2.02 (s, 3H), 1.97 (s, 3H), 1.16 (t, J = 7.1 Hz, 3H).

^{13}C NMR (101 MHz, $CDCl_3$): δ = 170.6, 170.6, 167.0, 169.9, 166.8 (C1, $^3J_{C\text{-}1/H\text{-}3ax}$ = 0 Hz, 101 MHz), 166.0, 165.9, 165.5, 133.6, 133.6, 133.3, 130.1, 130.0, 129.8, 129.2, 129.1, 128.9, 128.6, 128.4, 98.6, 96.9, 72.2, 70.4, 69.8, 68.8, 68.7, 68.0, 66.4, 64.7, 63.1, 62.2, 62.1, 55.8, 31.9, 20.9, 20.9, 20.8, 14.1.

HRMS (ESI) m/z Calcd for $C_{46}H_{50}O_{20}Na$ $[M + Na]^+$ 945.2787, found: 945.2782.

Ethyl (4,5,7,8-tetra-*O*-acetyl-3-deoxy-β-D-manno-oct-2-ulopyranosyl)onate-(2→6)-methyl 2-acetamido-3,4-di-*O*-benzyl-2-deoxy-α-D-glucopyranoside (36rβ)

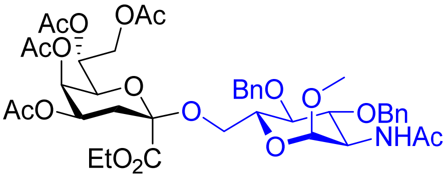

36rβ

GP1: 41.4 mg, 83% yield from **18**: a colorless syrup. (CH_2Cl_2/MeOH = 30:1, v/v)

36rβ: ^{1}H NMR (600 MHz, $CDCl_3$): δ = 7.36 – 7.23 (m, 10H), 5.34 (d, J = 9.5 Hz, 1H), 5.28 (s, 1H), 5.20 – 5.16 (m, 1H), 4.94 – 4.88 (m, 1H), 4.85 – 4.79 (m, 2H), 4.67 – 4.57 (m, 3H), 4.38 – 4.33 (m, 1H), 4.29 (dd, J = 12.3, 4.5 Hz, 1H), 4.26 – 4.18 (m, 2H), 4.11 – 4.03 (m, 3H), 3.76 (dd, J = 9.8, 4.9 Hz, 1H), 3.71 – 3.65 (m, 1H), 3.61 – 3.58 (m, 2H), 3.34 (s, 3H), 2.42 (dd, J = 11.6, 3.8 Hz, 1H), 2.13 (t, J = 12.8 Hz, 1H), 2.10 (s, 3H), 2.09 (s, 3H), 2.00 (s, 3H), 1.99 (s, 3H), 1.84 (s, 3H), 1.22 (t, J = 7.1 Hz, 3H).

^{13}C NMR (151 MHz, $CDCl_3$): δ = 170.7, 170.6, 170.0, 169.9, 169.9, 167.4 (C1, $^3J_{C\text{-}1/H\text{-}3ax}$ = 5.4 Hz, 151 MHz), 138.5, 138.3, 128.6, 128.6, 128.5, 128.3, 127.9, 127.8, 127.8, 99.4, 98.6, 80.5, 78.2, 75.0, 74.7, 70.8, 70.3, 68.1, 67.2, 64.1, 63.4, 62.3, 62.2, 54.9, 52.6, 32.3, 23.5, 20.9, 20.8, 20.8, 14.2.

HRMS (ESI) m/z Calcd for $C_{41}H_{54}NO_{17}$ $[M + H]^+$ 832.3386, found: 832.3379.

Ethyl (4,5,7,8-tetra-*O*-acetyl-3-deoxy-α-D-manno-oct-2-ulopyranosyl)onate-(2→6)-methyl 2-acetamido-3,4-di-*O*-benzyl-2-deoxy-α-D-glucopyranoside (36rα)

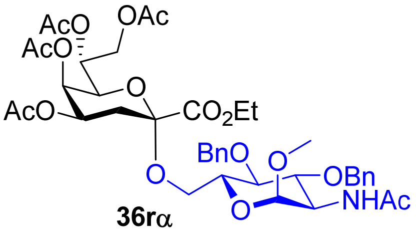

36rα

GP2: 8.0 mg, 16% yield from **18** to generate **36rβ**: a colorless syrup (CH_2Cl_2/MeOH = 30:1, v/v); while 27.5 mg, 55% yield from **18** to generate **36rα**: a colorless syrup (CH_2Cl_2/MeOH = 30:1, v/v).

36rα: ^{1}H NMR (600 MHz, $CDCl_3$): δ = 7.35 – 7.27 (m, 10H), 5.37 – 5.25 (m, 3H), 5.22 – 5.14 (m, 1H), 4.89 (d, *J* = 11.2 Hz, 1H), 4.81 (d, *J* = 11.6 Hz, 1H), 4.64 – 4.57 (m, 3H), 4.55 (d, *J* = 11.2 Hz, 1H), 4.23 – 4.14 (m, 4H), 4.06 (dd, *J* = 12.3, 4.6 Hz, 1H), 3.84 – 3.77 (m, 1H), 3.71 (d, *J* = 10.1 Hz, 1H), 3.67 (t, *J* = 9.6 Hz, 1H), 3.53 – 3.45 (m, 1H), 3.41 – 3.34 (m, 4H), 2.15 (dd, *J* = 12.7, 4.8 Hz, 1H), 2.09 – 2.05 (m, 4H), 2.02 (s, 3H), 2.00 (s, 3H), 1.95 (s, 3H), 1.83 (s, 3H), 1.24 (t, *J* = 7.1 Hz, 3H).

^{13}C NMR (151 MHz, $CDCl_3$): δ = 170.7, 170.6, 170.0, 170.0, 169.9, 166.9 (C1, $^3J_{C\text{-}1/H\text{-}3ax}$ = 0 Hz, 151 MHz), 138.3, 137.9, 128.7, 128.6, 128.4, 128.1, 127.9, 98.4, 98.4, 80.7, 79.3, 75.2, 75.2, 70.3, 68.6, 68.1, 66.6, 64.8, 63.4, 62.2, 62.0, 55.1, 52.8, 32.0, 23.6, 21.0, 20.9, 20.9, 20.8, 14.2.

HRMS (ESI) m/z Calcd for $C_{41}H_{54}NO_{17}$ $[M + H]^+$ 832.3386, found: 832.3394.

Ethyl (4,5,7,8-tetra-*O*-acetyl-3-deoxy-β-D-manno-oct-2-ulopyranosyl)onate-(2→6)-1,2:3,4-di-*O*-isopropylidene-α-D-galactopyranose (36sβ)[25]

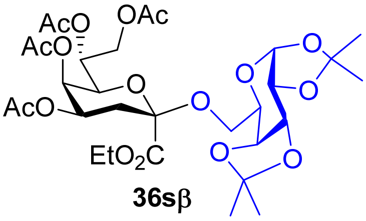

36sβ

GP1: 40.4 mg, 98% yield from **18**: a white amorphous solid. (Petroleum ether/ethyl acetate = 3:1, v/v)

36sβ: ^{1}H NMR (400 MHz, $CDCl_3$): δ = 5.50 (d, *J* = 4.9 Hz, 1H), 5.27 (s, 1H), 5.14 (ddd, *J* = 9.5, 4.5, 2.3 Hz, 1H), 4.90 (ddd, *J* = 13.2, 4.6, 3.2 Hz, 1H), 4.59 (dd, *J* = 7.9, 2.1 Hz, 1H), 4.40 (d, *J* = 12.2 Hz, 1H), 4.34 (d, *J* = 4.6 Hz, 1H), 4.31 – 4.22 (m, 4H), 4.18 (d, *J* = 9.5 Hz, 1H), 3.93 (q, *J* = 5.4 Hz, 2H), 3.59 (dd, *J* = 12.2, 8.4 Hz, 1H), 2.40 (dd, *J* = 12.6, 4.6 Hz, 1H), 2.11 (t, *J* = 12.9 Hz, 1H), 2.09 (s, 3H), 2.07 (s, 3H), 1.99 (s, 3H), 1.97 (s, 3H), 1.55 (s, 3H), 1.42 (s, 3H), 1.32 (t, *J* = 6.4 Hz, 9H).

^{13}C NMR (101 MHz, $CDCl_3$): δ = 170.8, 170.6, 170.1, 169.9, 167.8 (C1, $^3J_{C\text{-}1/H\text{-}3ax}$ = 5.5 Hz, 101 MHz), 109.5, 108.8, 99.7, 96.4, 71.0, 70.9, 70.8, 70.7, 68.2, 67.6, 67.3, 64.2, 63.9, 62.5, 62.2, 32.4, 26.3, 26.1, 25.2, 24.6, 20.9, 20.9, 20.8, 20.8, 14.3.

The spectroscopic data coincided with the previous report.[25]

Ethyl (4,5,7,8-tetra-*O*-acetyl-3-deoxy-α-D-manno-oct-2-ulopyranosyl)onate-(2→6)-1,2:3,4-di-*O*-isopropylidene-α-D-galactopyranose (36sα)[3]

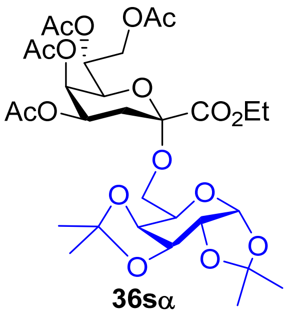

36sα

GP2: 9.9 mg, 18% yield from **18** to generate **36sβ**: a white amorphous solid (Petroleum ether/ethyl acetate = 3:1, v/v); while 30.2 mg, 72% yield from **18** to generate **36sα**: a colorless syrup (Petroleum ether/ethyl acetate = 3:1, v/v).

36sα: ^{1}H NMR (400 MHz, $CDCl_3$): δ = 5.51 (d, *J* = 4.9 Hz, 1H), 5.37 – 5.33 (m, 2H), 5.27 (ddd, *J* = 9.7, 5.5, 2.3 Hz, 1H), 4.60 – 4.55 (m, 2H), 4.34 (dd, *J* = 9.8, 0.8 Hz, 1H), 4.30 – 4.22 (m, 3H), 4.20 (dd, *J* = 8.0, 1.7 Hz, 1H), 4.14 (dd, *J* = 12.3, 5.6 Hz, 1H), 3.99 – 3.96 (m, 1H), 3.67 – 3.60 (m, 2H), 2.20 – 2.17 (m, 1H), 2.10 – 2.07 (m, 4H), 2.06 (s, 3H), 2.00 (s, 3H), 1.97 (s, 3H), 1.56 (s, 3H), 1.41 (s, 3H), 1.34 – 1.30 (m, 9H).

The spectroscopic data coincided with the previous report.[3]

Ethyl (4,5,7,8-tetra-*O*-acetyl-3-deoxy-β-D-manno-oct-2-ulopyranosyl)onate-(2→1)-Diacetone-β-D-fructopyranose (36tβ)

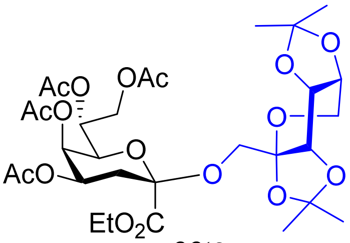

36tβ

GP1: 34.0 mg, 83% yield from **18** to generate **36tβ**: a colorless syrup (Petroleum ether/ethyl acetate = 3:1, v/v); while 6.1 mg, 15% yield from **18** to generate **36tα**: a white amorphous solid (Petroleum ether/ethyl acetate = 3:1, v/v).

36tβ: ^{1}H NMR (600 MHz, $CDCl_3$): δ = 5.29 (s, 1H), 5.17 (ddd, *J* = 9.3, 4.5, 2.1 Hz, 1H), 4.96 – 4.88 (m, 1H), 4.62 (dd, *J* = 7.8, 2.5 Hz, 1H), 4.51 – 4.42 (m, 2H), 4.33 (dd, *J* = 12.3, 4.7 Hz, 1H), 4.28 – 4.19 (m, 3H), 4.16 (d, *J* = 9.5 Hz, 1H), 3.91 – 3.82 (m, 2H), 3.69 (d, *J* = 12.9 Hz, 1H), 3.65 (d, *J* = 10.5 Hz, 1H), 2.45 (dd, *J* = 12.5, 4.5 Hz, 1H), 2.13 – 2.08 (m, 4H), 2.07 (s, 3H), 1.99 (s, 3H), 1.98 (s, 3H), 1.53 (s, 3H), 1.45 (s, 3H), 1.42 (s, 3H), 1.35 – 1.30 (m, 6H).

^{13}C NMR (101 MHz, $CDCl_3$): δ = 170.7, 170.5, 170.0, 170.0, 167.3 (C1, $^3J_{C\text{-}1/H\text{-}3ax}$ = 5.3 Hz, 151 MHz), 109.2, 108.9, 102.0, 99.3, 71.1, 71.0, 70.4, 69.9, 68.1, 67.2, 65.2, 64.2, 62.5, 62.4, 61.4, 32.3, 26.7,

25.9, 25.3, 24.2, 20.9, 20.8, 20.8, 14.3.

HRMS (ESI) m/z Calcd for $C_{30}H_{44}O_{17}Na$ $[M + Na]^+$ 699.2470, found: 699.2463.

Ethyl (4,5,7,8-tetra-*O*-acetyl-3-deoxy-α-D-manno-oct-2-ulopyranosyl)onate-(2→1)-Diacetone-β-D-fructopyranose (36tα)[3]

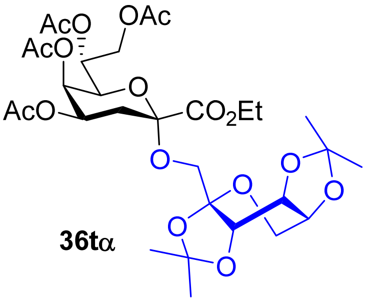

36tα

GP2: 7.7 mg, 19% yield from **18** to generate **36tβ**: a colorless syrup (Petroleum ether/ethyl acetate = 3:1, v/v); while 26.1 mg, 64% yield from **18** to generate **36tα**: a white amorphous solid (Petroleum ether/ethyl acetate = 3:1, v/v).

36tβ: ^{1}H NMR (600 MHz, $CDCl_3$): δ = 5.41 (ddd, *J* = 12.3, 5.0, 3.1 Hz, 1H), 5.36 (d, *J* = 3.0 Hz, 1H), 5.22 (ddd, *J* = 9.7, 3.2, 2.2 Hz, 1H), 4.62 – 4.56 (m, 2H), 4.47 (d, *J* = 2.7 Hz, 1H), 4.27 (dt, *J* = 8.5, 4.7 Hz, 2H), 4.23 – 4.19 (m, 2H), 4.16 (dd, *J* = 12.5, 3.3 Hz, 1H), 3.90 (dd, *J* = 12.9, 1.8 Hz, 1H), 3.71 – 3.64 (m, 2H), 3.35 (d, *J* = 10.0 Hz, 1H), 2.26 – 2.20 (m, 1H), 2.10 (s, 3H), 2.09 (s, 3H), 2.06 (t, *J* = 12.5 Hz, 1H), 2.02 (s, 3H), 1.98 (s, 3H), 1.55 (s, 3H), 1.50 (s, 3H), 1.41 (s, 3H), 1.35 – 1.29 (m, 6H). The spectroscopic data coincided with the previous report.[3]

2,2'-(1,6-Hexanediyl)bis-[Ethyl (4,5,7,8-tetra-*O*-acetyl-3-deoxy-β-D-manno-oct-2-ulopyranosyl) onat]yl ester (36uβ)

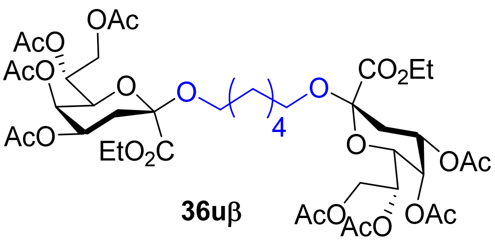

36uβ

GP1(0.5 equiv. of **S6**): 19.2 mg, 67% yield from **18** to generate **36uβ**: a colorless syrup. (Petroleum ether/ethyl acetate = 3:1, v/v)

36uβ: ^{1}H NMR (400 MHz, $CDCl_3$): δ = 5.30 – 5.25 (m, 2H), 5.17 (dt, *J* = 9.6, 3.4 Hz, 2H), 4.87 (ddd, *J* = 13.2, 4.6, 3.0 Hz, 2H), 4.36 (d, *J* = 3.5 Hz, 4H), 4.26 (q, *J* = 7.1 Hz, 4H), 4.19 (dd, *J* = 9.6, 1.3 Hz, 2H), 3.74 (dt, *J* = 9.1, 6.4 Hz, 2H), 3.28 (dt, *J* = 9.2, 6.7 Hz, 2H), 2.37 (dd, *J* = 12.1, 4.8 Hz, 2H), 2.14 – 2.07 (m, 14H), 2.00 (s, 6H), 1.98 (s, 6H), 1.60 – 1.50 (m, 4H), 1.33 (t, *J* = 7.1 Hz, 10H).

^{13}C NMR (101 MHz, $CDCl_3$): δ = 170.9, 170.7, 170.1, 170.0, 168.0 (C1, $^3J_{C\text{-}1/H\text{-}3ax}$ = 5.8 Hz, 101 MHz),

99.5, 70.7, 68.2, 67.3, 64.8, 64.2, 62.5, 62.1, 32.6, 29.7, 25.9, 21.0, 20.9, 20.9, 14.4.

HRMS (ESI) m/z Calcd for $C_{42}H_{63}O_{24}$ $[M + H]^+$ 951.3703, found: 951.3708.

Ethyl (4,5,7,8-tetra-*O*-acetyl-3-deoxy-β-D-manno-oct-2-ulopyranosyl)onate-(2→5)-D-glucurono-6,3-lactone acetonide (36vβ)

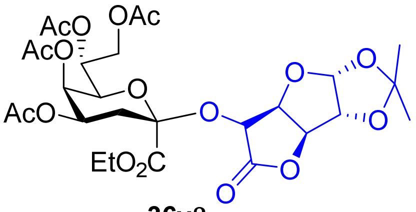

36vβ

GP1: 27.4 mg, 72% yield from **18** to generate **36vβ**: a colorless syrup (Petroleum ether/ethyl acetate = 2:1, v/v); while 10.2 mg, 27% yield from **18** to generate **36vα**: a white amorphous solid (Petroleum ether/ethyl acetate = 2:1, v/v).

36vβ: ^{1}H NMR (600 MHz, $CDCl_3$): δ = 6.01 (d, *J* = 3.7 Hz, 1H), 5.27 (s, 1H), 5.16 (dd, *J* = 9.5, 1.9 Hz, 1H), 4.99 (d, *J* = 4.3 Hz, 1H), 4.95 – 4.89 (m, 2H), 4.86 (d, *J* = 2.6 Hz, 1H), 4.79 (d, *J* = 3.7 Hz, 1H), 4.64 (dd, *J* = 12.6, 3.7 Hz, 1H), 4.29 (q, *J* = 7.2 Hz, 2H), 4.12 (d, *J* = 11.2 Hz, 1H), 3.97 (d, *J* = 9.5 Hz, 1H), 2.62 (dd, *J* = 12.3, 4.4 Hz, 1H), 2.19 (t, *J* = 12.8 Hz, 1H), 2.14 (s, 3H), 2.13 (s, 3H), 2.03 (s, 3H), 2.00 (s, 3H), 1.56 (s, 3H), 1.38 – 1.32 (m, 6H).

^{13}C NMR (101 MHz, $CDCl_3$): δ = 171.2, 171.2, 170.7, 170.0, 170.0, 166.5 (C1, $^3J_{C\text{-}1/H\text{-}3ax}$ = 5.0 Hz, 101 MHz), 113.4, 107.1, 99.4, 82.7, 81.9, 78.9, 71.0, 70.7, 68.3, 67.1, 63.8, 63.2, 61.8, 32.0, 27.0, 26.6, 21.2, 20.9, 14.1.

HRMS (ESI) m/z Calcd for $C_{27}H_{36}O_{17}Na$ $[M + Na]^+$ 655.1844, found: 655.1841.

Ethyl (4,5,7,8-tetra-*O*-acetyl-3-deoxy-α-D-manno-oct-2-ulopyranosyl)onate-(2→5)-D-glucurono-6,3-lactone acetonide (36vα)

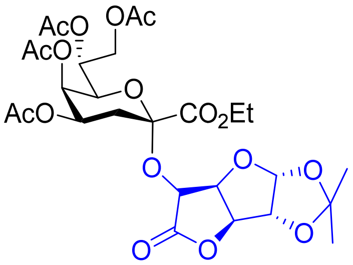

36vα

GP1: 23.1 mg, 61% yield from **18** to generate **36vα**: a white amorphous solid. (Petroleum ether/ethyl acetate = 2:1, v/v)

36vα: ^{1}H NMR (600 MHz, $CDCl_3$): δ = 6.00 (d, *J* = 3.6 Hz, 1H), 5.49 – 5.41 (m, 2H), 5.13 (dt, *J* =

9.8, 2.8 Hz, 1H), 4.89 (dd, J = 4.1, 2.9 Hz, 1H), 4.84 (d, J = 4.3 Hz, 1H), 4.80 – 4.75 (m, 3H), 4.40 (dd, J = 12.4, 2.5 Hz, 1H), 4.34 – 4.21 (m, 3H), 2.33 – 2.24 (m, 2H), 2.07 (s, 3H), 2.02 (s, 3H), 2.01 (s, 3H), 1.96 (s, 3H), 1.48 (s, 3H), 1.36 (t, J = 7.2 Hz, 3H), 1.33 (s, 3H).

^{13}C NMR (151 MHz, $CDCl_3$): δ = 171.7, 171.2, 170.3, 170.1, 169.5, 166.9 (C1, $^3J_{C\text{-}1/H\text{-}3ax}$ = 0 Hz, 151 MHz), 113.3, 107.1, 98.7, 82.8, 82.0, 77.7, 70.4, 68.8, 68.0, 66.2, 64.7, 62.7, 61.4, 30.8, 27.0, 26.7, 20.8, 20.8, 20.8, 20.7, 14.0.

HRMS (ESI) m/z Calcd for $C_{27}H_{36}O_{17}Na$ $[M + Na]^+$ 655.1844, found: 655.1842.

Ethyl (4,5,7,8-tetra-*O*-acetyl-3-deoxy-β-D-manno-oct-2-ulopyranosyl)onate-(2→3)-1,2:5,6-di-*O*-isopropylidene-α-D-glucofuranose (36wβ)

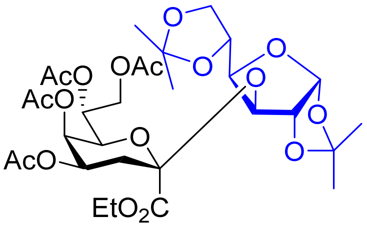

36wβ

GP1: 34.4 mg, 85% yield from **18** to generate **36wβ**: a colorless syrup (Petroleum ether/ethyl acetate = 4:1, v/v); while 5.7 mg, 14% yield from **18** to generate **36wα**: a white amorphous solid (Petroleum ether/ethyl acetate = 3:1, v/v).

36wβ: ^{1}H NMR (600 MHz, $CDCl_3$): δ = 5.98 (d, J = 3.2 Hz, 1H), 5.28 (s, 1H), 5.15 (dd, J = 5.9, 3.4 Hz, 1H), 4.92 (d, J = 13.1 Hz, 1H), 4.57 (d, J = 3.3 Hz, 1H), 4.41 (d, J = 12.3 Hz, 1H), 4.34 (ddd, J = 10.4, 9.6, 3.7 Hz, 2H), 4.30 – 4.24 (m, 2H), 4.19 (dd, J = 13.1, 6.5 Hz, 2H), 3.98 (d, J = 11.0 Hz, 1H), 3.70 (t, J = 6.5 Hz, 1H), 3.54 (dd, J = 10.8, 6.4 Hz, 1H), 2.42 (dd, J = 12.6, 4.4 Hz, 1H), 2.16 – 2.09 (m, 4H), 2.09 (s, 3H), 2.00 (s, 3H), 1.98 (s, 3H), 1.49 (s, 3H), 1.36 (s, 3H), 1.35 (s, 3H), 1.32 (s, 3H), 1.25 (s, 3H).

^{13}C NMR (151 MHz, $CDCl_3$): δ = 170.8, 170.6, 170.0, 169.9, 167.7 (C1, $^3J_{C\text{-}1/H\text{-}3ax}$ = 6.9 Hz, 151 MHz), 112.2, 106.5, 101.0, 99.7, 84.2, 79.1, 75.2, 71.4, 71.0, 68.2, 67.3, 65.1, 64.2, 62.5, 62.3, 32.5, 29.8, 27.3, 26.7, 24.2, 24.1, 21.0, 20.9, 20.8, 20.8, 14.3.

HRMS (ESI) m/z Calcd for $C_{30}H_{44}O_{17}Na$ $[M + Na]^+$ 699.2470, found: 699.2468.

Ethyl (4,5,7,8-tetra-*O*-acetyl-3-deoxy-α-D-manno-oct-2-ulopyranosyl)onate-(2→3)-1,2:5,6-di-*O*-isopropylidene-α-D-glucofuranose (36wα)[2b]

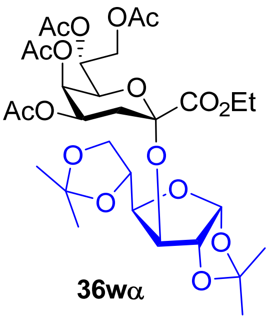

36wα

GP2: 23.2 mg, 57% yield from **18**: a pale-yellow syrup. (Petroleum ether/ethyl acetate = 4:1, v/v)

36wα: ^{1}H NMR (600 MHz, $CDCl_3$): δ = 5.92 (d, J = 3.6 Hz, 1H), 5.38 (s, 1H), 5.28 – 5.26 (m, 1H), 5.18 (dt, J = 9.5, 3.3 Hz, 1H), 4.78 (dd, J = 12.3, 2.5 Hz, 1H), 4.64 (d, J = 3.6 Hz, 1H), 4.32 – 4.21 (m, 6H), 4.10 – 4.05 (m, 2H), 4.01 (dd, J = 8.5, 5.8 Hz, 1H), 2.29 (dd, J = 13.2, 4.6 Hz, 1H), 2.14 (t, J = 12.9 Hz, 1H), 2.10 (s, 3H), 2.07 (s, 3H), 1.98 (s, 3H), 1.97 (s, 3H), 1.48 (s, 3H), 1.41 (s, 3H), 1.36 – 1.34 (m, 6H), 1.30 (s, 3H).

The spectroscopic data coincided with the previous report.[2b]

Ethyl (4,5,7,8-tetra-*O*-acetyl-3-deoxy-β-D-manno-oct-2-ulopyranosyl)onate-(2→4)-methyl-2,3-*O*-isopropylidene-α-L-rhamnopyranoside (36xβ)

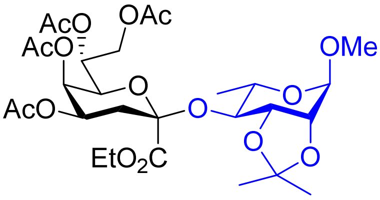

36xβ

GP1: 30.5 mg, 80% yield from **18** to generate **36xβ**: a colorless syrup (Petroleum ether/ethyl acetate = 4:1, v/v); while 7.2 mg, 19% yield from **18** to generate **36xα**: a colorless syrup (Petroleum ether/ethyl acetate = 4:1, v/v).

36xβ: ^{1}H NMR (600 MHz, $CDCl_3$): δ = 5.27 (d, J = 1.1 Hz, 1H), 5.13 (ddd, J = 9.5, 4.8, 2.3 Hz, 1H), 4.83 (ddd, J = 13.3, 4.5, 3.0 Hz, 1H), 4.80 (s, 1H), 4.40 (dd, J = 12.3, 2.3 Hz, 1H), 4.35 (dd, J = 12.3, 4.9 Hz, 1H), 4.27 – 4.15 (m, 3H), 4.08 (d, J = 6.4 Hz, 1H), 4.03 (d, J = 5.9 Hz, 1H), 3.71 (dd, J = 9.8, 6.7 Hz, 1H), 3.57 (dq, J = 12.6, 6.3 Hz, 1H), 3.35 (s, 3H), 2.45 (dd, J = 12.4, 4.3 Hz, 1H), 2.07 (s, 3H), 2.07 (s, 3H), 2.02 (t, J = 12.9 Hz, 1H), 1.99 (s, 3H), 1.98 (s, 3H), 1.42 (s, 3H), 1.34 – 1.28 (m, 6H), 1.26 (s, 3H).

^{13}C NMR (101 MHz, $CDCl_3$): δ = 170.7, 170.5, 170.0, 167.0, 167.2 (C1, $^{3}J_{C\text{-}1/H\text{-}3ax}$ = 5.0 Hz, 101 MHz), 109.3, 98.6, 98.0, 77.7, 76.2, 75.8, 71.2, 68.2, 67.2, 64.5, 64.3, 62.8, 62.0, 55.0, 33.6, 27.7, 26.5, 20.9, 20.9, 20.7, 18.1, 14.0.

HRMS (ESI) m/z Calcd for $C_{28}H_{42}O_{16}Na$ $[M + Na]^+$ 657.2365, found: 657.2363.

Ethyl (4,5,7,8-tetra-*O*-acetyl-3-deoxy-α-D-manno-oct-2-ulopyranosyl)onate-(2→4)-methyl-2,3-*O*-isopropylidene-α-L-rhamnopyranoside (36xα)

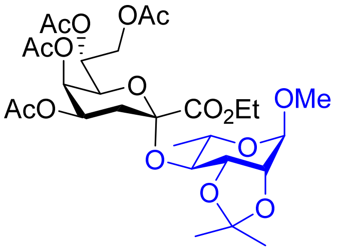

36xα

GP2: 2.8 mg, 6% yield from **18** to generate **36xβ**: a colorless syrup (Petroleum ether/ethyl a cetate = 4:1, v/v); while 31.2 mg, 94% yield from **18** to generate the mixture of **36xα** and **glycal** (1:0.6): a colorless syrup (Petroleum ether/ethyl acetate = 4:1, v/v).

The mixture of **36xα** and **glycal** (1:0.6)**:** ^{1}H NMR (600 MHz, $CDCl_3$): δ = 5.88 (d, *J* = 1.7 Hz, 0.6H, **glycal**), 5.71 (dd, *J* = 2.8, 1.7 Hz, 0.6H, **glycal**), 5.47 (d, *J* = 4.5 Hz, 0.6H, **glycal**), 5.36 – 5.29 (m, 3H, **α**), 5.28 – 5.25 (m, 0.6H, **glycal**), 4.81 (s, 1H, **α**), 4.65 – 4.60 (m, 1H&0.6H), 4.34 (d, *J* = 9.6 Hz, 0.6H, **glycal**), 4.30 – 4.20 (m, 4H&1.8H), 4.05 (dd, *J* = 17.7, 6.0 Hz, 2H, **α**), 3.62 (dt, *J* = 12.4, 6.2 Hz, 1H, **α**), 3.55 (dd, *J* = 9.6, 6.5 Hz, 1H, **α**), 3.36 (s, 3H, **α**), 2.19 (dd, *J* = 12.6, 4.7 Hz, 1H, **α**), 2.12 – 2.07 (m, 1H, **α**), 2.08 (s, 3.6H, **glycal**), 2.07 (s, 3H, **α**), 2.05 (s, 3H, **α**), 2.03 (s, 1.8H, **glycal**), 2.03 (s, 1.8H, **glycal**), 1.98 (s, 3H, **α**), 1.97 (s, 3H, **α**), 1.49 (s, 3H, **α**), 1.35 – 1.31 (m, 6H&1.8H), 1.22 (d, *J* = 6.2 Hz, 3H, **α**).

^{13}C NMR (101 MHz, $CDCl_3$): δ = 170.8, 170.7, 170.6, 170.4, 170.2, 170.2, 169.8, 169.6, 166.5 (C1, $^3J_{C\text{-}1/H\text{-}3ax}$ = 0 Hz, 101 MHz), 161.2, 145.0, 109.7, 107.4, 99.8, 97.9, 79.3, 76.8, 75.6, 73.5, 69.4, 68.3, 67.4, 66.5, 64.9, 64.7, 64.1, 63.8, 62.0, 62.0, 61.8, 60.9, 54.9, 33.5, 27.7, 26.2, 21.0, 20.9, 20.8, 20.8, 20.8, 20.7, 17.8, 14.2, 14.1.

HRMS (ESI) m/z Calcd for $C_{28}H_{42}O_{16}Na$ $[M + Na]^+$ 657.2365, found: 657.2369.

Ethyl (4,5,7,8-tetra-*O*-acetyl-3-deoxy-β-D-manno-oct-2-ulopyranosyl)onate-(2→4)-[ethyl (2-*O*-acetyl-7,8-di-*O*-isopropylidene-3-deoxy-α-D-manno-oct-2-ulopyranosyl)onate] (36yβ)

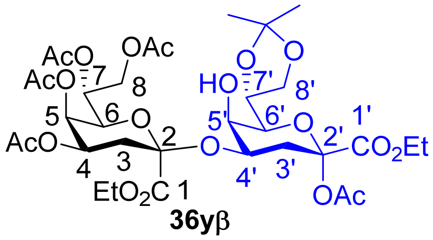

36yβ

GP1: 29.7 mg, 65% yield from **18** to generate **36yβ**: a colorless syrup (Petroleum ether/ethyl acetate =

1:1, v/v).

36yβ: ^{1}H NMR (600 MHz, $CDCl_3$): δ = 5.27 (s, 1H, H-5), 5.12 (d, *J* = 8.8 Hz, 1H, H-7), 4.89 – 4.83 (m, 1H, H-4), 4.70 (dd, *J* = 12.5, 3.3 Hz, 1H, H-8), 4.43 – 4.38 (m, 1H, H-7'), 4.29 – 4.25 (m, 3H, H-6 & CH_2 of Et group on Kdo donor), 4.22 (q, *J* = 7.1 Hz, 3H, H-4' & CH_2 of Et group on Kdo acceptor), 4.17 (s, 1H, H-5'), 4.10 (dd, *J* = 9.0, 6.3 Hz, 1H, H-8'), 3.97 – 3.91 (m, 2H, H-8 & H-8'), 3.75 (d, *J* = 8.5 Hz, 1H, H-6'), 2.37 (dd, *J* = 12.5, 4.4 Hz, 1H, H-3), 2.24 (t, *J* = 12.6 Hz, 1H, H-3'), 2.14 (s, 3H, OAc), 2.13 (s, 3H, OAc), 2.11 (s, 3H, OAc), 2.07 (t, *J* = 13.0 Hz, 1H, H-3), 2.02 (s, 3H, OAc), 2.01 – 1.99 (m, 1H, H-3'), 1.98 (s, 3H, OAc), 1.43 (s, 3H, CH_3), 1.36 (s, 3H, CH_3), 1.35 (t, *J* = 7.1 Hz, 3H, CH_2 of Et group on Kdo donor), 1.25 (t, *J* = 7.1 Hz, 3H, CH_2 of Et group on Kdo acceptor).

^{13}C NMR (151 MHz, $CDCl_3$): δ = 170.6, 170.3, 170.0, 169.9, 168.8, 167.6 (C1, $^3J_{C\text{-}1/H\text{-}3ax}$ = 5.5 Hz, 151 MHz), 166.6, 109.7, 100.1, 98.4, 74.3, 73.1, 70.7, 70.3, 68.4, 67.4, 66.9, 64.0, 62.6, 62.2, 61.5, 32.9, 31.1, 26.8, 25.3, 21.0, 20.9, 20.8, 14.2, 14.0.

HRMS (ESI) m/z Calcd for $C_{33}H_{48}O_{20}Na$ $[M + Na]^+$ 787.2631, found: 787.2625.

Ethyl (4,5,7,8-tetra-*O*-acetyl-3-deoxy-α-D-manno-oct-2-ulopyranosyl)onate-(2→4)-[ethyl (2-*O*-acetyl-7,8-di-*O*-isopropylidene-3-deoxy-α-D-manno-oct-2-ulopyranosyl)onate] (36yα)

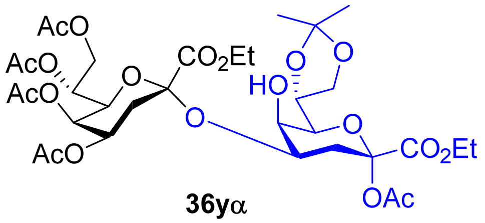

36yα

GP2: 5.0 mg, 11% yield from **18** to generate **36yβ**: a colorless syrup (Petroleum ether/ethyl acetate = 1:1, v/v); while 24.4 mg, 53% yield from **18** to generate **36yα**: a colorless syrup (Petroleum ether/ethyl acetate = 1:1, v/v).

36yα: ^{1}H NMR (600 MHz, $CDCl_3$): δ = 5.39 (s, 1H), 5.27 (s, 2H), 4.77 (dd, *J* = 5.7, 3.5 Hz, 1H), 4.38 (s, 1H), 4.34 – 4.21 (m, 6H), 4.11 (d, *J* = 7.2 Hz, 2H), 4.04 (d, *J* = 4.0 Hz, 1H), 3.85 (s, 2H), 3.42 (s, 1H), 2.30 (dd, *J* = 9.1, 3.5 Hz, 1H), 2.22 (t, *J* = 12.3 Hz, 1H), 2.14 (s, 3H), 2.11 – 2.02 (m, 8H), 1.99 (s, 6H), 1.38 (s, 6H), 1.33 (s, 3H), 1.34 – 1.20 (m, 3H).

^{13}C NMR (151 MHz, $CDCl_3$): δ = 170.9, 170.4, 170.1, 169.7, 168.4, 167.5 (C1, $^3J_{C\text{-}1/H\text{-}3ax}$ = 0 Hz, 151 MHz), 166.8, 109.5, 98.0, 97.9, 74.3, 72.9, 71.9, 69.8, 68.5, 67.8, 67.5, 66.2, 64.3, 64.2, 62.7, 62.5, 62.0, 32.3, 32.1, 29.8, 27.0, 25.1, 22.8, 20.9, 20.8, 14.1, 14.1.

HRMS (ESI) m/z Calcd for $C_{33}H_{52}O_{20}N$ $[M + NH_4]^+$ 782.3077, found: 782.3069.

Ethyl (4,5,7,8-tetra-*O*-acetyl-3-deoxy-β-D-manno-oct-2-ulopyranosyl)onate-(2→3)-methyl 2-*O*-

benzyl-4,6-*O*-benzylidene-α-D-mannopyranoside (36zβ)[11]

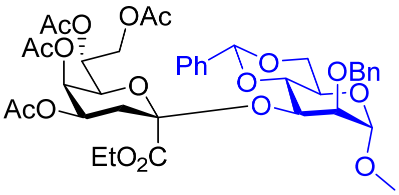

36zβ

GP1 (**S25** 3.0 equiv.): 24.5 mg, 51% yield from **18** to generate **36zβ**: a white amorphous solid (Petroleum ether/ethyl acetate = 3:1, v/v); while 11.1 mg, 23% yield from **18** to generate **36zα**: a white amorphous solid (Petroleum ether/ethyl acetate = 3:1, v/v).

36zβ: ^{1}H NMR (400 MHz, $CDCl_3$): δ = 7.49 (dd, *J* = 8.2, 1.3 Hz, 2H), 7.40 (d, *J* = 7.3 Hz, 2H), 7.37 – 7.32 (m, 6H), 5.61 (s, 1H), 5.31 – 5.28 (m, 1H), 5.11 – 5.05 (m, 2H), 4.95 (d, *J* = 12.1 Hz, 1H), 4.77 (d, *J* = 12.0 Hz, 1H), 4.63 (d, *J* = 1.6 Hz, 1H), 4.53 (dd, *J* = 10.3, 3.2 Hz, 1H), 4.39 (dd, *J* = 12.3, 2.3 Hz, 1H), 4.29 – 4.21 (m, 2H), 4.18 – 4.10 (m, 2H), 3.97 (dd, *J* = 9.5, 0.9 Hz, 1H), 3.89 (t, *J* = 10.3 Hz, 1H), 3.82 (dd, *J* = 9.4, 4.5 Hz, 1H), 3.73 (dd, *J* = 3.1, 1.6 Hz, 1H), 3.33 (s, 3H), 2.43 – 2.37 (m, 2H), 2.07 (s, 3H), 2.06 (s, 3H), 2.01 (s, 3H), 1.97 (s, 3H), 1.29 (t, *J* = 7.1 Hz, 3H).

The spectroscopic data coincided with the previous report.[11]

Ethyl (4,5,7,8-tetra-*O*-acetyl-3-deoxy-α-D-manno-oct-2-ulopyranosyl)onate-(2→3)-methyl 2-*O*-benzyl-4,6-*O*-benzylidene-α-D-mannopyranoside (36zα)[3]

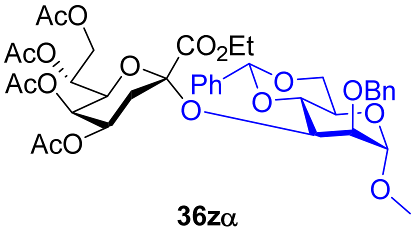

36zα

GP2: 41.2 mg, 87% yield from **18**: a colorless syrup. (Petroleum ether/ethyl acetate = 3:1, v /v)

36zα: ^{1}H NMR (400 MHz, $CDCl_3$): δ = 7.49 – 7.40 (m, 6H), 7.36 – 7.31 (m, 4H), 5.49 (s, 1H), 5.35 – 5.26 (m, 2H), 5.13 (ddd, *J* = 9.6, 4.3, 2.7 Hz, 1H), 4.84 (d, *J* = 6.8 Hz, 2H), 4.73 (d, *J* = 1.6 Hz, 1H), 4.51 (ddd, *J* = 13.3, 11.2, 2.9 Hz, 2H), 4.22 (dd, *J* = 9.9, 4.5 Hz, 1H), 4.14 – 4.09 (m, 1H), 4.04 – 3.97 (m, 2H), 3.81 (dt, *J* = 12.0, 7.6 Hz, 2H), 3.72 (ddd, *J* = 10.8, 7.2, 3.0 Hz, 2H), 3.35 – 3.26 (m, 4H), 2.39 (dd, *J* = 13.0, 5.2 Hz, 1H), 2.07 – 2.03 (m, 4H), 1.98 (s, 3H), 1.88 (s, 3H), 1.87 (s, 3H), 0.90 (t, *J* = 7.1 Hz, 3H).

The spectroscopic data coincided with the previous report.[3]

Ethyl (4,5,7,8-tetra-*O*-acetyl-3-deoxy-β-D-manno-oct-2-ulopyranosyl)onate-(2→3)-1,2:5,6-Di-*O*-isopropylidene-alpha-D-allofuranose (36aaβ)

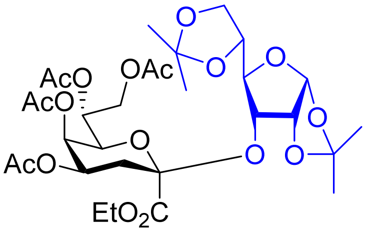

36aaβ

GP1 (**S26** 3.0 equiv.): 21.5 mg, from **18** to generate the mixture of **36aaβ** (44% yield) and **glycal** (1:0.33): a colorless syrup (Petroleum ether/ethyl acetate = 4:1, v/v); while 8.2 mg, 20% yield from **18** to generate **36aaα**: a white amorphous solid (Petroleum ether/ethyl acetate = 4:1, v/v).

36aaβ: ^{1}H NMR (600 MHz, $CDCl_3$): δ = 5.40 (s, 1H), 5.32 (s, 1H), 5.23 – 5.20 (m, 1H), 5.14 – 5.08 (m, 1H), 4.86 (d, *J* = 6.0 Hz, 1H), 4.74 (d, *J* = 5.9 Hz, 1H), 4.38 (dd, *J* = 12.4, 1.8 Hz, 1H), 4.32 – 4.23 (m, 5H), 4.15 – 4.11 (m, 1H), 4.06 – 4.01 (m, 2H), 2.24 (t, *J* = 12.6 Hz, 1H), 2.10 (s, 3H), 2.09 (s, 3H), 2.06 (s, 3H), 2.04 – 2.03 (m, 4H), 1.48 (s, 3H), 1.46 (s, 3H), 1.37 (s, 3H), 1.34 (t, *J* = 7.1 Hz, 3H), 1.31 (s, 3H).

^{13}C NMR (151 MHz, $CDCl_3$): δ = 171.0, 170.5, 170.0, 169.9, 167.1, 112.6, 110.3, 107.4, 104.4, 99.5, 88.6, 86.5, 81.5, 75.4, 69.7, 68.0, 67.1, 66.3, 64.6, 62.7, 62.4, 31.5, 29.8, 27.2, 26.6, 24.9, 24.9, 20.9, 20.8, 14.6, 14.1.

HRMS (ESI) m/z Calcd for $C_{30}H_{45}O_{17}$ $[M + H]^+$ 677.2651, found: 677.2654.

Ethyl (4,5,7,8-tetra-*O*-acetyl-3-deoxy-α-D-manno-oct-2-ulopyranosyl)onate-(2→3)-1,2:5,6-Di-*O*-isopropylidene-alpha-D-allofuranose (36aaα)[3]

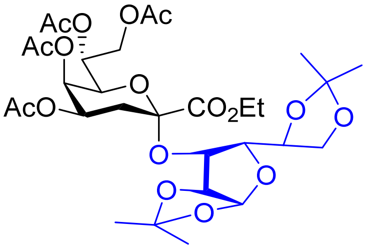

36aaα

GP2: 33.9 mg, 83% yield from **18**: a colorless syrup. (Petroleum ether/ethyl acetate = 3:1, v/v)

36aaα: ^{1}H NMR (400 MHz, $CDCl_3$): δ = 5.66 (d, *J* = 3.8 Hz, 1H), 5.41 (ddd, *J* = 12.4, 4.8, 2.9 Hz, 1H), 5.35 – 5.30 (m, 1H), 5.25 (ddd, *J* = 9.8, 4.5, 2.8 Hz, 1H), 4.71 (dd, *J* = 12.2, 2.8 Hz, 1H), 4.64 (dd, *J* = 9.8, 1.5 Hz, 1H), 4.53 – 4.47 (m, 1H), 4.28 – 4.14 (m, 4H), 4.08 – 3.98 (m, 2H), 3.95 – 3.84

(m, 2H), 2.39 (ddd, J = 13.1, 4.8, 0.9 Hz, 1H), 2.14 – 2.05 (m, 7H), 1.97 (s, 3H), 1.96 (s, 3H), 1.55 (s, 3H), 1.50 (s, 3H), 1.45 (s, 3H), 1.33 (t, J = 7.2 Hz, 3H), 1.28 (s, 3H).

The spectroscopic data coincided with the previous report.[3]

Ethyl (4,5,7,8-tetra-*O*-acetyl-3-deoxy-α-D-manno-oct-2-ulopyranosyl)onate-(2→3)-2-azidoethyl-2-*O*-benzoyl-4,6-*O*-benzylidene-β-D-galactopyranoside (36abα)

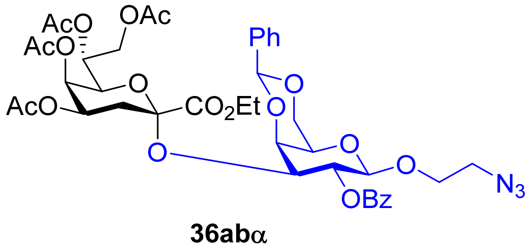

36abα

GP1: 43.3 mg, 84% yield from **18**: a colorless syrup. (Petroleum ether/ethyl acetate = 1:1, v/v).

GP2: 30.9 mg, 60% yield from **18**: a colorless syrup. (Petroleum ether/ethyl acetate = 1:1, v/v).

36abα: ^{1}H NMR (400 MHz, $CDCl_3$): δ = 8.15 (d, J = 7.5 Hz, 2H), 7.61 (t, J = 7.4 Hz, 1H), 7.57 – 7.53 (m, 2H), 7.49 (t, J = 7.7 Hz, 2H), 7.45 – 7.36 (m, 3H), 5.76 – 5.69 (m, 1H), 5.44 (s, 1H), 5.16 (dt, J = 9.7, 3.3 Hz, 1H), 4.89 – 4.81 (m, 1H), 4.77 (s, 1H), 4.72 (d, J = 8.1 Hz, 1H), 4.65 (dd, J = 12.2, 3.0 Hz, 1H), 4.35 (dd, J = 12.3, 3.8 Hz, 2H), 4.28 (d, J = 10.1 Hz, 1H), 4.22 (dd, J = 11.8, 6.0 Hz, 2H), 4.16 (dd, J = 10.2, 3.6 Hz, 1H), 4.12 – 4.01 (m, 2H), 3.94 – 3.83 (m, 1H), 3.74 – 3.65 (m, 1H), 3.53 (s, 1H), 3.41 (ddd, J = 12.2, 8.2, 3.8 Hz, 1H), 3.30 (dt, J = 13.2, 4.1 Hz, 1H), 2.35 (dd, J = 13.1, 4.7 Hz, 1H), 2.21 (s, 3H), 1.98 (s, 3H), 1.93 (s, 3H), 1.88 (t, J = 12.8 Hz, 1H), 1.82 (s, 3H), 1.09 (t, J = 7.1 Hz, 3H). ^{13}C NMR (101 MHz, $CDCl_3$): δ = 170.8, 170.2, 169.6, 168.9, 168.3 (C1, $^3J_{C\text{-}1/H\text{-}3ax}$ = 0 Hz, 151 MHz), 165.2, 137.5, 133.5, 130.1, 129.4, 129.2, 128.6, 128.4, 126.4, 101.2, 100.9, 96.5, 72.7, 71.6, 69.4, 69.2, 68.9, 67.6, 67.0, 66.6, 65.8, 63.9, 62.4, 62.3, 50.9, 31.9, 21.3, 20.8, 20.7, 20.6, 13.8.

HRMS (ESI) m/z Calcd for $C_{40}H_{51}O_{18}N_4$ [M + NH_4]$^+$ 875.3192, found: 875.3199.

Ethyl (4,5,7,8-tetra-*O*-acetyl-3-deoxy-α-D-manno-oct-2-ulopyranosyl)onate-(2→3)-methyl 2-*O*-acetyl-4-*O*-benzyl-α-L-rhamnopyranoside (36acα)

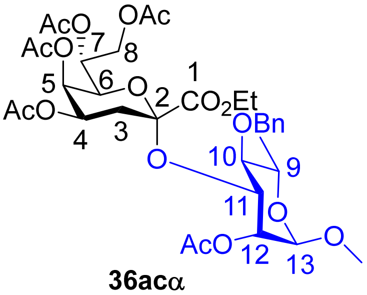

36acα

GP1: 38.4 mg, 88% yield from **18**: a colorless syrup. (Petroleum ether/ethyl acetate = 2:1, v/v).

GP2: 37.8 mg, 87% yield from **18**: a colorless syrup. (Petroleum ether/ethyl acetate = 2:1, v/v).

36acα: ^{1}H NMR (400 MHz, $CDCl_3$): δ = 7.45 (d, *J* = 7.4 Hz, 2H, OBn), 7.37 (t, *J* = 7.4 Hz, 2H, OBn), 7.31 (d, *J* = 7.3 Hz, 1H, OBn), 5.38 – 5.30 (m, 1H, H-4), 5.13 – 5.03 (m, 1H, H-7), 4.93 (s, 1H, H-5), 4.80 (q, *J* = 10.7 Hz, 3H, H-12 & OCH_2Ph), 4.53 (d, *J* = 10.0 Hz, 2H, H-6 & H-13), 4.44 (dd, *J* = 12.0, 2.9 Hz, 1H, H-8), 4.37 – 4.30 (m, 1H, CH_2 of Et group), 4.28 (dd, *J* = 9.8, 3.1 Hz, 1H, H-11), 4.12 (dq, *J* = 10.8, 7.2 Hz, 1H, CH_2 of Et group), 3.99 (dd, *J* = 12.1, 5.8 Hz, 1H, H-8), 3.77 (dq, *J* = 12.4, 6.2 Hz, 1H, H-9), 3.49 (t, *J* = 9.7 Hz, 1H, H-10), 3.34 (s, 3H, OMe), 2.28 (dd, *J* = 13.1, 4.9 Hz, 1H, H-3), 2.16 (s, 3H, OAc), 2.05 – 2.02 (m, 4H, H-3 & OAc), 1.99 (s, 3H, OAc), 1.98 (s, 3H, OAc), 1.82 (s, 3H, OAc), 1.39 (d, *J* = 6.2 Hz, 3H, Me), 1.30 (t, *J* = 7.1 Hz, 3H, CH_3 of Et group).

^{13}C NMR (151 MHz, $CDCl_3$): δ = 170.8, 170.5, 170.5, 170.0, 169.8, 167.6 (C1, $^{3}J_{C\text{-}1/H\text{-}3ax}$ = 0 Hz, 151 MHz), 137.5, 128.6, 128.0, 127.9, 98.2, 96.6, 80.0 76.1, 70.3, 70.2, 68.6, 68.4, 67.7, 66.6, 64.4, 62.8, 62.6, 55.1, 32.2, 21.1, 21.0, 20.9, 20.8, 20.8, 18.2, 13.8.

HRMS (ESI) m/z Calcd for $C_{34}H_{46}O_{17}Na$ $[M + Na]^+$ 749.2627, found: 749.2632.

Ethyl (4,5,7-tri-*O*-acetyl-8-*O*-*tert*-butyldiphenylsilyl-3-deoxy-β-D-manno-oct-2-ulopyranosyl)onate-(2→6)-methyl 2,3,4-tri-*O*-benzyl-α-D-glucopyranoside (42pβ)

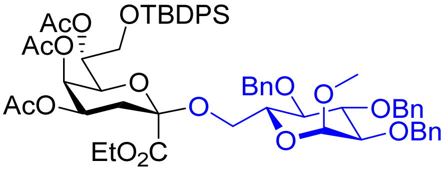

42pβ

GP1: 51.0 mg, 79% yield from **18**: a colorless syrup. (Petroleum ether/ethyl acetate = 4:1, v/v)

42pβ: ^{1}H NMR (600 MHz, $CDCl_3$): δ = 7.64 (dd, *J* = 12.0, 7.7 Hz, 4H), 7.39 – 7.28 (m, 16H), 7.26 – 7.21 (m, 2H), 7.19 (t, *J* = 8.2 Hz, 3H), 5.29 (s, 1H), 5.17 – 5.12 (m, 1H), 4.99 – 4.92 (m, 2H), 4.82 – 4.76 (m, 3H), 4.67 (d, *J* = 12.2 Hz, 1H), 4.62 (d, *J* = 3.1 Hz, 1H), 4.55 (d, *J* = 11.0 Hz, 1H), 4.14 – 4.06 (m, 2H), 3.98 (t, *J* = 9.3 Hz, 1H), 3.95 – 3.90 (m, 2H), 3.87 (d, *J* = 11.2 Hz, 1H), 3.81 (dd, *J* = 11.4, 5.2 Hz, 1H), 3.75 (dd, *J* = 10.0, 4.0 Hz, 1H), 3.63 (dd, *J* = 10.4, 4.5 Hz, 1H), 3.54 – 3.48 (m, 2H), 3.37 (s, 3H), 2.37 (dd, *J* = 12.4, 4.5 Hz, 1H), 2.13 (t, *J* = 12.7 Hz, 1H), 2.06 (s, 3H), 1.98 (s, 3H), 1.95 (s, 3H), 1.07 (t, *J* = 7.1 Hz, 3H), 1.01 (s, 9H).

^{13}C NMR (151 MHz, $CDCl_3$): δ = 170.7, 170.0, 169.9, 167.4 (C1, $^{3}J_{C\text{-}1/H\text{-}3ax}$ = 6.2 Hz, 151 MHz), 138.8, 138.4, 138.3, 135.7, 135.7, 133.6, 133.4, 129.8, 129.7, 128.6, 128.5, 128.5, 128.2, 128.1, 128.0, 127.8, 127.7, 127.7, 127.6, 99.5, 98.1, 82.3, 80.0, 77.5, 75.9, 74.6, 73.5, 71.2, 70.5, 69.6, 67.4, 64.4, 63.1, 62.8, 62.1, 55.2, 32.1, 26.8, 20.9, 20.9, 19.4, 14.1.

HRMS (ESI) m/z Calcd for $C_{60}H_{72}O_{16}SiNa$ $[M + Na]^+$ 1099.4481, found: 1099.4476.

Ethyl (4-*O*-trimethylacetyl-5,7-di-*O*-acetyl-8-*O*-*tert*-butyldiphenylsilyl-3-deoxy-β-D-manno-oct-

2-ulopyranosyl)onate-(2→6)-methyl 2,3,4-tri-*O*-benzyl-α-D-glucopyranoside (43pβ)

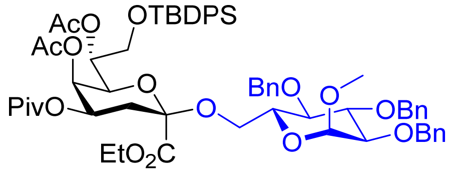

43pβ

GP1: 54.2 mg, 81% yield from **18**: a colorless syrup. (Petroleum ether/ethyl acetate = 6:1, v/v)

43pβ: ^{1}H NMR (600 MHz, $CDCl_3$): δ = 7.64 (dd, *J* = 11.9, 6.8 Hz, 4H), 7.39 – 7.27 (m, 16H), 7.26 – 7.17 (m, 5H), 5.36 (s, 1H), 5.16 – 5.12 (m, 1H), 4.97 (d, *J* = 10.8 Hz, 1H), 4.95 – 4.89 (m, 1H), 4.83 – 4.76 (m, 3H), 4.67 (d, *J* = 12.2 Hz, 1H), 4.62 (d, *J* = 3.3 Hz, 1H), 4.55 (d, *J* = 10.9 Hz, 1H), 4.15 (d, *J* = 9.5 Hz, 1H), 4.10 (dt, *J* = 14.3, 7.7 Hz, 1H), 3.96 (dt, *J* = 7.2, 6.4 Hz, 2H), 3.92 (d, *J* = 10.3 Hz, 1H), 3.89 – 3.85 (m, 1H), 3.82 (dd, *J* = 11.4, 5.2 Hz, 1H), 3.76 – 3.72 (m, 1H), 3.64 (dd, *J* = 10.4, 4.4 Hz, 1H), 3.55 – 3.50 (m, 2H), 3.37 (s, 3H), 2.39 (dd, *J* = 12.3, 4.5 Hz, 1H), 2.11 (t, *J* = 12.6 Hz, 1H), 2.04 (s, 3H), 1.96 (s, 3H), 1.13 (s, 9H), 1.08 (t, *J* = 7.1 Hz, 3H), 1.01 (s, 9H).

^{13}C NMR (151 MHz, $CDCl_3$): δ = 177.1, 170.4, 170.0, 167.5 (C1, $^{3}J_{C\text{-}1/H\text{-}3ax}$ = 5.3 Hz, 151 MHz), 138.9, 138.4, 138.3, 135.7, 135.7, 133.6, 133.4, 129.8, 129.7, 128.6, 128.5, 128.5, 128.2, 128.1, 128.0, 127.8, 127.7, 127.6, 99.4, 98.1, 82.3, 80.0, 77.5, 75.9, 74.6, 73.4, 71.3, 70.5, 69.5, 67.4, 64.3, 63.0, 62.8, 62.1, 55.2, 38.8, 32.3, 27.1, 26.8, 21.0, 20.9, 19.4, 14.1.

HRMS (ESI) m/z Calcd for $C_{63}H_{79}O_{16}Si$ $[M + H]^{+}$ 1119.5131, found: 1119.5127.

Ethyl (4,7-di-*O*-trimethylacetyl-5-*O*-acetyl-8-*O*-*tert*-butyldiphenylsilyl-3-deoxy-β-D-manno-oct-2-ulopyranosyl)onate-(2→6)-methyl 2,3,4-tri-*O*-benzyl-α-D-glucopyranoside (44pβ)

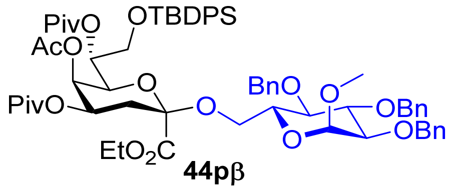

44pβ

GP1: 56.3 mg, 81% yield from **18**: a colorless syrup. (Petroleum ether/ethyl acetate = 10:1, v/v)

44pβ: ^{1}H NMR (600 MHz, $CDCl_3$): δ = 7.68 – 7.61 (m, 4H), 7.40 – 7.35 (m, 4H), 7.34 – 7.26 (m, 12H), 7.25 – 7.19 (m, 3H), 7.16 (d, *J* = 6.8 Hz, 2H), 5.26 (s, 1H), 5.19 (ddd, *J* = 9.1, 4.9, 2.5 Hz, 1H), 4.97 (d, *J* = 10.8 Hz, 1H), 4.94 – 4.88 (m, 3H), 4.82 – 4.75 (m, 2H), 4.67 (d, *J* = 12.2 Hz, 1H), 4.62 (d, *J* = 3.5 Hz, 1H), 4.50 (d, *J* = 11.0 Hz, 1H), 4.18 (d, *J* = 9.3 Hz, 1H), 4.07 (dq, *J* = 10.9, 7.1 Hz, 1H), 3.98 (t, *J* = 9.2 Hz, 1H), 3.94 – 3.88 (m, 1H), 3.87 – 3.81 (m, 2H), 3.71 (dd, *J* = 10.0, 2.1 Hz, 1H), 3.60 (dd, *J* = 10.4, 4.2 Hz, 1H), 3.53 (dd, *J* = 9.6, 3.4 Hz, 2H), 3.36 (s, 3H), 2.37 (dd, *J* = 12.3, 4.5 Hz, 1H), 2.09 (t, *J* = 12.6 Hz, 1H), 2.04 (s, 3H), 1.18 (s, 9H), 1.12 (s, 9H), 1.08 (t, *J* = 7.1 Hz, 3H), 1.03 (s, 9H).

^{13}C NMR (151 MHz, $CDCl_3$): δ = 177.3, 177.1, 170.0, 167.5 (C1, $^{3}J_{C\text{-}1/H\text{-}3ax}$ = 5.5 Hz, 151 MHz), 138.9,

138.5, 138.3, 135.8, 135.8, 133.6, 133.1, 129.8, 129.7, 128.6, 128.6, 128.5, 128.4, 128.2, 128.2, 128.1, 128.0, 127.7, 127.7, 127.6, 127.6, 127.4, 99.3, 98.1, 82.3, 80.0, 77.4, 75.8, 74.6, 73.4, 71.4, 70.4, 69.5, 67.5, 64.4, 62.9, 62.8, 62.1, 55.3, 39.0, 38.8, 35.6, 32.6, 27.2, 27.2, 27.1, 26.9, 21.0, 19.3, 14.1.

HRMS (ESI) m/z Calcd for $C_{66}H_{84}O_{16}SiNa$ $[M + Na]^+$ 1183.5420, found: 1183.5404.

Ethyl (4-*O*-acetyl-5,7-di-*O*-*tert*-butylsilyl-8-*O*-*tert*-butyldiphenylsilyl-3-deoxy-α-D-manno-oct-2-ulopyranosyl)onate-(2→6)-methyl 2,3,4-tri-*O*-benzyl-α-D-glucopyranoside (45pα)

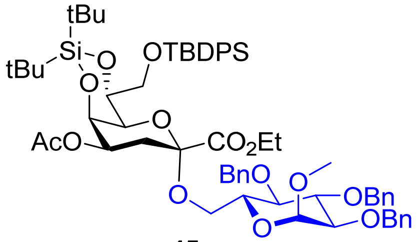

45pα

GP1: 57.8 mg, 85% yield from **18**: a colorless syrup. (Petroleum ether/ethyl acetate = 8:1, v/v)

45pα: ^{1}H NMR (600 MHz, $CDCl_3$): δ = 7.66 (d, *J* = 7.0 Hz, 4H), 7.44 – 7.34 (m, 6H), 7.33 – 7.20 (m, 15H), 5.18 – 5.08 (m, 1H, H-4), 4.95 (d, *J* = 10.9 Hz, 1H), 4.83 (d, *J* = 11.0 Hz, 1H), 4.76 (d, *J* = 10.9 Hz, 1H), 4.64 (d, *J* = 12.1 Hz, 1H), 4.55 (d, *J* = 12.1 Hz, 1H), 4.45 (d, *J* = 11.0 Hz, 1H), 4.40 (d, *J* = 3.5 Hz, 1H), 4.36 (s, 1H), 4.31 (t, *J* = 5.9 Hz, 1H), 4.16 – 4.07 (m, 2H), 4.00 (s, 1H), 3.92 (t, *J* = 9.2 Hz, 1H), 3.71 – 3.65 (m, 2H), 3.68 (dd, *J* = 10.4, 5.1 Hz, 1H), 3.62 (dd, *J* = 10.3, 7.0 Hz, 1H), 3.42 (t, *J* = 9.2 Hz, 1H), 3.37 (s, 3H), 3.32 (dd, *J* = 9.6, 3.6 Hz, 1H), 3.06 (t, *J* = 9.5 Hz, 1H), 2.30 (t, *J* = 12.1 Hz, 1H, H-3), 2.10 (dd, *J* = 12.6, 4.1 Hz, 1H, H-3), 2.07 (s, 3H), 1.18 (t, *J* = 7.2 Hz), 1.06 (s, 9H), 0.99 (s, 9H), 0.93 (s, 9H).

^{13}C NMR (151 MHz, $CDCl_3$): δ = 170.4, 167.7 (C1, $^3J_{C\text{-}1/H\text{-}3ax}$ = 0 Hz, 151 MHz), 139.0, 138.3, 138.2, 135.7, 135.6, 133.4, 133.2, 129.9, 129.9, 128.5, 128.5, 128.5, 128.4, 128.1, 128.1, 128.0, 128.0, 127.9, 127.9, 127.8, 127.7, 127.7, 98.6, 97.3, 82.3, 80.1, 78.9, 77.0, 75.8, 75.1, 73.2, 70.9, 70.0, 69.5, 66.3, 66.2, 63.6, 61.7, 55.0, 31.4, 27.8, 27.2, 27.0, 22.1, 21.7, 21.2, 19.3, 14.2.

HRMS (ESI) m/z Calcd for $C_{64}H_{88}O_{14}NSi_2$ $[M + NH_4]^+$ 1150.5737, found: 1150.5744.

Ethyl (4,5:7,8-di-*O*-isopropylidene-3-deoxy-α/β-D-manno-oct-2-ulopyranosyl)onate-(2→6)-methyl 2,3,4-tri-*O*-benzyl-α-D-glucopyranoside (46p)[8]

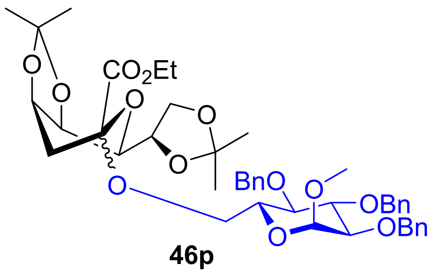

46p

GP1: 31.9 mg, 67% yield from **18** to generate the mixture of **46pβ** and **46pα** (1:1): a pale-yellow oil. (Petroleum ether/ethyl acetate = 4:1, v/v)

46 (α/β = 1:1): ^{1}H NMR (600 MHz, $CDCl_3$): δ = 7.38 – 7.26 (m, 30H), 4.96 (dd, *J* = 10.8, 5.8 Hz, 2H), 4.83 (d, *J* = 10.9 Hz, 2H), 4.81 – 4.74 (m, 4H), 4.70 – 4.63 (m, 3H), 4.60 (d, *J* = 3.5 Hz, 1H), 4.57 – 4.53 (m, 2H), 4.50 – 4.45 (m, 2H), 4.36 (dd, *J* = 12.3, 6.1 Hz, 1H), 4.27 – 4.20 (m, 3H), 4.19 – 4.08 (m, 6H), 4.00 – 3.94 (m, 3H), 3.89 (dd, *J* = 8.7, 6.3 Hz, 1H), 3.78 – 3.72 (m, 2H), 3.71 – 3.64 (m, 2H), 3.61 (d, *J* = 9.4 Hz, 2H), 3.54 – 3.48 (m, 3H), 3.39 (d, *J* = 10.3 Hz, 1H), 3.37 – 3.35 (m, 6H), 3.30 (t, *J* = 9.4 Hz, 1H), 2.76 (dd, *J* = 15.3, 4.1 Hz, 1H), 2.22 – 2.20 (m, 2H), 1.85 (dd, *J* = 15.3, 2.9 Hz, 1H), 1.47 (s, 3H), 1.41 (s, 3H), 1.38 (s, 3H), 1.35 (s, 3H), 1.33 (s, 3H), 1.31 (s, 3H), 1.24 – 1.18 (m, 6H). The spectroscopic data coincided with the previous report.[8]

Ethyl (4,5:7,8-di-*O*-isopropylidene-3-deoxy-β-D-manno-oct-2-ulopyranosyl)onate-(2→6)-methyl 2-*O*-benzyl-4,6-*O*-benzylidene-α-D-mannopyranoside (46zβ)[11]

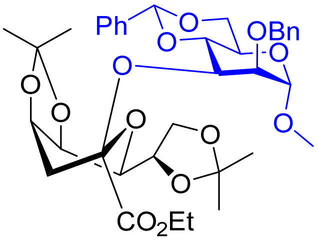

46zβ

GP1: 35.7 mg, 85% yield from **18**: a colorless syrup. (Petroleum ether/ethyl acetate = 4:1, v/v)

46zβ: ^{1}H NMR (500 MHz, $CDCl_3$): δ = 7.50 (d, *J* = 6.4 Hz, 2H), 7.44 (d, *J* = 7.2 Hz, 2H), 7.37 – 7.27 (m, 6H), 5.52 (s, 1H), 4.73 (s, 2H), 4.67 (s, 1H), 4.56 (dd, *J* = 10.3, 3.1 Hz, 1H), 4.46 (d, *J* = 7.4 Hz, 1H), 4.29 (q, *J* = 5.8 Hz, 1H), 4.21 (dd, *J* = 9.9, 4.3 Hz, 1H), 4.16 – 4.08 (m, 2H), 4.07 – 4.01 (m, 2H), 3.86 (d, *J* = 1.3 Hz, 1H), 3.80 (t, *J* = 10.2 Hz, 1H), 3.73 (td, *J* = 9.7, 4.4 Hz, 1H), 3.62 (dq, *J* = 10.9, 7.2 Hz, 1H), 3.38 (dd, *J* = 6.1, 1.3 Hz, 1H), 3.34 (s, 3H), 3.26 (dq, *J* = 10.9, 7.2 Hz, 1H), 3.19 (dd, *J* = 15.4, 3.2 Hz, 1H), 1.64 (dd, *J* = 15.1, 1.5 Hz, 1H), 1.42 (s, 3H), 1.37 (s, 3H), 1.28 (s, 3H), 1.25 (s, 3H), 0.85 (t, *J* = 7.1 Hz, 3H).

The spectroscopic data coincided with the previous report.[11]

Ethyl (4,5:7,8-di-*O*-isopropylidene-3-deoxy-α-D-manno-oct-2-ulopyranosyl)onate-(2→6)-methyl 2-*O*-benzyl-4,6-*O*-benzylidene-α-D-mannopyranoside (46zα)[11]

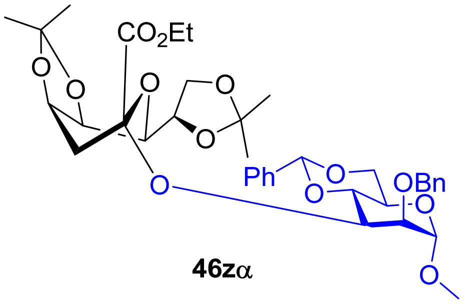

46zα

GP1: 4.3 mg, 10% yield from **18**: a colorless syrup. (Petroleum ether/ethyl acetate = 6:1, v/v)

46zα: ^{1}H NMR (600 MHz, $CDCl_3$): δ = 7.51 (d, *J* = 7.3 Hz, 2H), 7.40 (d, *J* = 7.6 Hz, 2H), 7.37 – 7.33 (m, 6H), 5.57 (s, 1H), 4.99 (d, *J* = 12.1 Hz, 1H), 4.85 (dd, *J* = 10.1, 2.9 Hz, 1H), 4.78 (d, *J* = 12.1 Hz, 1H), 4.59 (s, 1H), 4.50 (dd, *J* = 11.0, 5.2 Hz, 1H), 4.30 (dd, *J* = 11.0, 6.8 Hz, 1H), 4.25 – 4.19 (m, 3H), 4.13 (dd, *J* = 10.4, 6.3 Hz, 2H), 4.06 – 3.99 (m, 2H), 3.85 (t, *J* = 10.1 Hz, 1H), 3.81 – 3.77 (m, 2H), 3.31 (d, *J* = 9.7 Hz, 1H), 3.27 (s, 3H), 2.54 (dd, *J* = 16.0, 5.4 Hz, 1H), 2.46 (dd, *J* = 15.9, 4.6 Hz, 1H), 1.50 (s, 3H), 1.40 (s, 3H), 1.36 (s, 3H), 1.35 (s, 3H), 1.24 (t, *J* = 7.1 Hz, 3H).

The spectroscopic data coincided with the previous report.[11]

Ethyl (4,5,7,8-tetra-*O*-acetyl-2,3-dideoxy-D-manno-oct-2-enopyranosyl)onate (Glycal)[11]

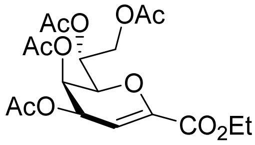

by-product
Glycal

Glycal: ^{1}H NMR (400 MHz, $CDCl_3$): δ = 5.88 (s, 1H), 5.75 – 5.68 (m, 1H), 5.47 (d, *J* = 3.9 Hz, 1H), 5.27 (ddd, *J* = 9.6, 3.9, 2.5 Hz, 1H), 4.62 (dd, *J* = 12.3, 2.3 Hz, 1H), 4.34 (d, *J* = 9.7 Hz, 1H), 4.31 – 4.21 (m, 3H), 2.08 (s, 6H), 2.03 (s, 3H), 2.03 (s, 3H), 1.32 (t, *J* = 7.1 Hz, 3H).

^{13}C NMR (101 MHz, $CDCl_3$): δ = 170.7, 170.5, 170.2, 169.6, 161.2, 107.4, 73.5, 67.5, 64.9, 62.0, 61.9, 60.9, 29.8, 20.9, 20.8, 20.8, 20.7, 14.2.

The spectroscopic data coincided with the previous report.[11]

4 X-ray Crystallography

Appropriate crystals for X-ray diffraction study were obtained by slow evaporation of petroleum ether/dichloromethane (4:1, v/v) solutions of compounds **36pα** and **36tα**, respectively.

Single crystal X-ray diffraction data of **36pα** were collected on a Rigaku Super Nova, Dual, Mo at zero, AtlasS2 diffractometer. The crystal was kept at 100 K during data collection

Single crystal X-ray diffraction data of **36tα** were collected on a Rigaku Super Nova, Dual, Mo at zero, AtlasS2 diffractometer. The crystal was kept at 100 K during data collection.

Using Olex2, the structure was solved with the ShelXT structure solution program using Direct Methods and refined with the ShelXL refinement package using Least Squares minimization. The disordered solvent molecules were removed with the SQUEEZE routine in PLATONS and the solvent-free model was employed for the final refinement. All non-hydrogen atoms were refined anisotropically. All hydrogen atoms were positioned by geometric idealization. Details of the crystal data and a summary of the intensity data collection parameters for **36pα** and **36tα**, are listed in **Section 5** and **6**. Crystallographic data were deposited at the Cambridge Crystallographic Data Center (CCDC 2382908 for **36pα**, 2382909 for **36tα**). The data can be obtained free of charge from The Cambridge Crystallographic Data Centre via www.ccdc.cam.ac.uk/structures.

4.1 Crystallographic Information of Ethyl (4,5,7,8-tetra-*O*-acetyl-deoxy-α-D-manno-oct-2-ulopyranosyl)onate-(2→6)-methyl 2,3,4-tri-*O*-benzyl-α-D-glucopyranoside (CCDC:2382908) :

**Empirical formula**	$C_{46}H_{56}O_{17}$
**Formula weight**	880.90
**Temperature/K**	99.99(10)
**Crystal system**	monoclinic
**Space group**	$P2_1$
**a/Å**	11.0048(2)
**b/Å**	9.98913(19)
**c/Å**	20.5935(4)
**α/°**	90.00
**β/°**	99.8007(19)
**γ/°**	90.00
**Volume/Å^3**	2230.76(8)
**Z**	2

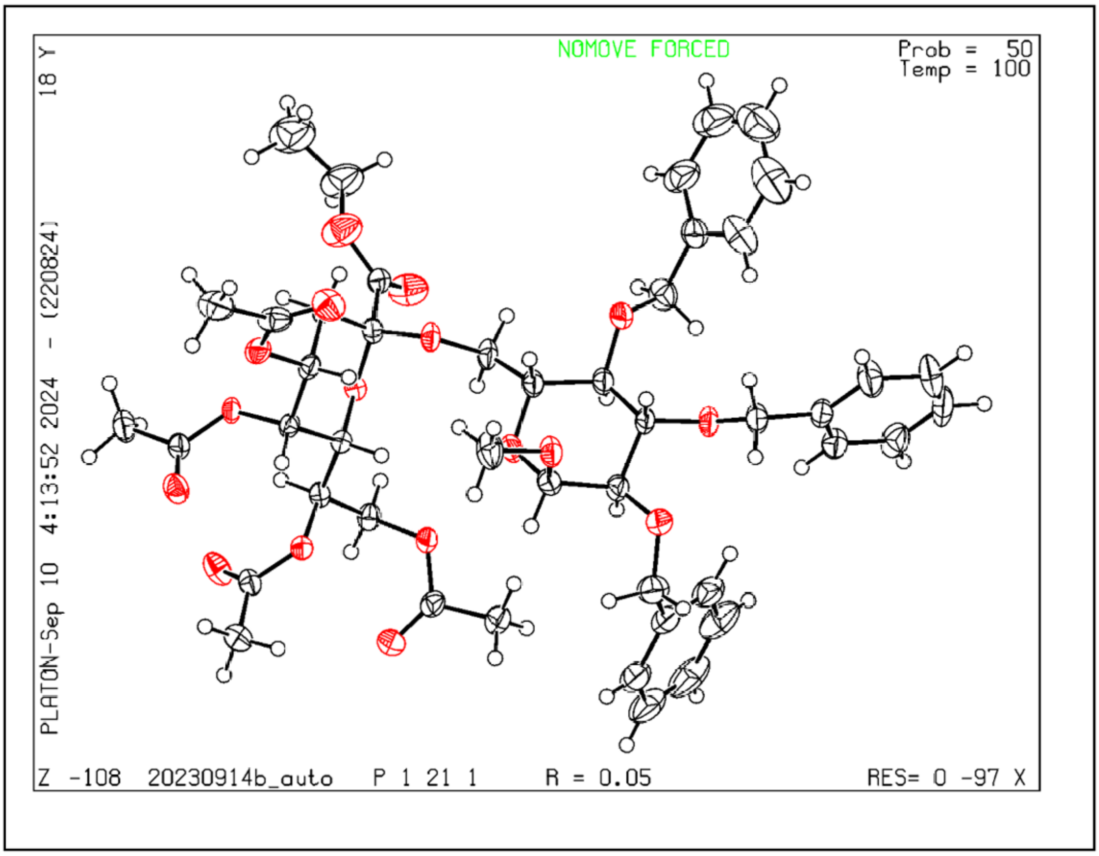

4.2 Crystallographic Information of Ethyl (4,5,7,8-tetra-*O*-acetyl-3-deoxy-α-D-manno-oct-2-ulopyranosyl)onate-(2→1)-Diacetone-β-D-fructopyranose (CCDC:2382909) :

Empirical formula	$C_{30}H_{44}O_{17}$
Formula weight	676.65
Temperature/K	100.00(10)
Crystal system	orthorhombic
Space group	$P2_12_12_1$
a/Å	10.19414(11)
b/Å	11.29721(14)
c/Å	28.9840(3)
α/°	90
β/°	90
γ/°	90
Volume/Å^3	3337.95(7)
Z	4

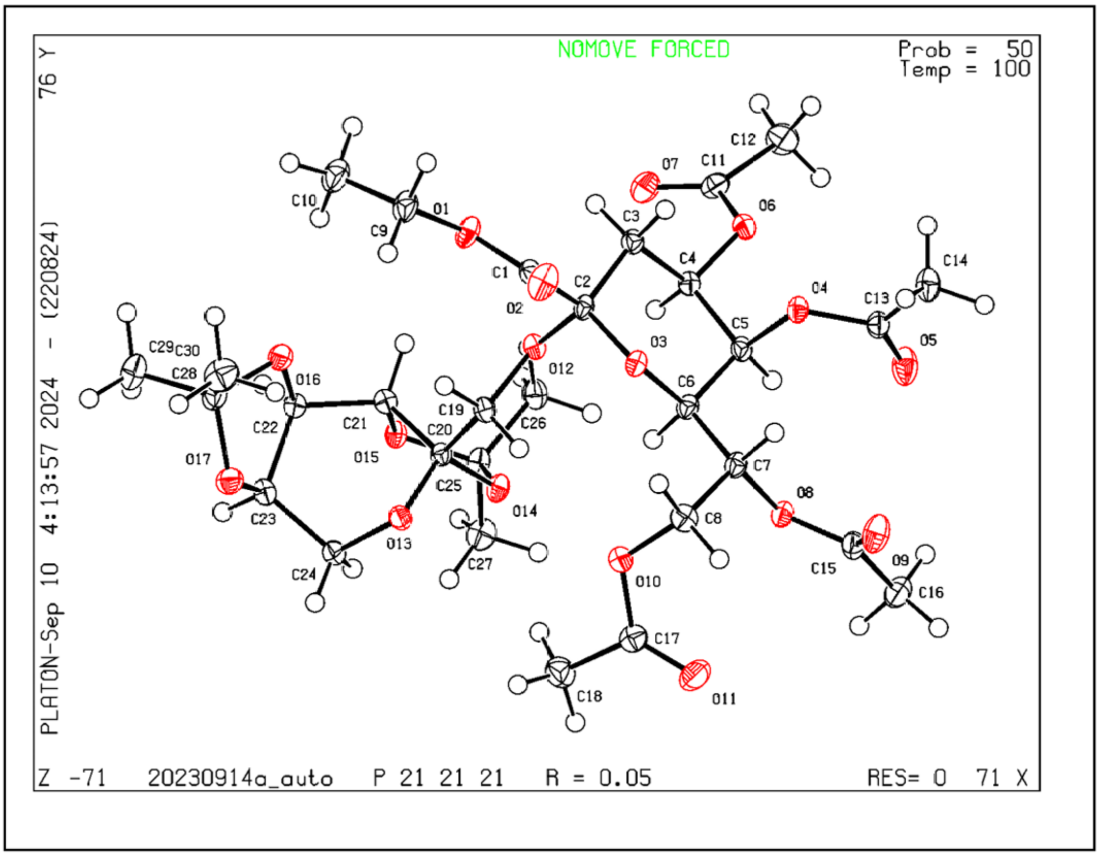

5 Computational methods and data

Computational methods. All calculations were carried out utilizing the Gaussian 16 program package.[26] All geometries of reagents, intermediates, transition states, and products were optimized by employing the B3LYP-D3(BJ) functional method[27-29], with the 6-311+g(d,p) basis set[30-34] for all elements. The solvent effect of the 1:1 mixture of dichloromethane (DCM) and acetonitrile (MeCN) was considered by the SMD model[35] (Eps=22.309, EpsInf=1.91761, HBondAcidity=0.085, HBondBasicity=0.185, SurfaceTensionAtInterface=40.18, CarbonAromaticity=0.000, ElectronegativeHalogenicity=0.333). The vibrational frequencies of all optimized stationary point structures were calculated at the same level of theory to obtain zero-point energies and Gibbs free energies. The optimized stationary point structures were subsequently characterized by frequency analyses at the same level of theory, from which zero-point energies and relative free-energies were obtained, in addition to conforming the stationary points to be either local minima (no imaginary frequencies) or transition states (one imaginary frequency). Moreover, intrinsic reaction coordinate (IRC)[36] calculations were carried out to confirm that the optimized transition states connect to their respective reactants and products. The vertical electronic excitations were calculated using time-dependent (TD)-B3LYP-D3(BJ) method based on the ground-state geometry optimized at the same level. Using SHERMO program to calculate the solutional free energy for all species (sclZPE= 0.9887).[21]

Table S8 The calculated total free energies in solution G_s in a.u. in the DCM/MeCN = 1:1 solvent (1M) with the temperature of 195.15K or 243.15K or 298.15K at B3LYP-D3(BJ)/6-311+g(d,p)/SMD level of theory.

Species	Gs (195.15K)	Gs (243.15K)	Gs (298.15K)
**18 (Kdo-STol)**	-2197.8900359	-2197.9029336	-2197.9195699
**31 (Umemoto's reagent)**	-1197.8775736	-1197.8844240	-1197.8930142
**$\cdot CF_3$**	-337.6730806	-337.6770714	-337.6818092
**$R_1S^{\cdot+}$**	-860.1557736	-860.1615760	-860.1687879
**47 (R_1S)**	-860.3725746	-860.3782363	-860.3852741
**32**	-2535.5624430	-2535.5769012	-2535.5954980
**$18^{\cdot+}$**	-2197.6759662	-2197.6889964	-2197.7058143
**48 (CF_3STol)**	-1006.9284413	-1006.9347790	-1006.9426146
**·33**	-1528.6649174	-1528.6763498	-1528.6909176
**33**	-1528.4836024	-1528.4947911	-1528.5090836
**34α**	-1661.2699824	-1661.2821373	-1661.2977007
**34'α**	-2623.0842285	-2623.0983855	-2623.1166663
**34β**	-1661.2645813	-1661.2765824	-1661.2919831
**35α**	-2543.8789626	-2543.8934984	-2543.9124082
**35β**	-2543.8779263	-2543.8924308	-2543.9113141
**35'β**	-3505.6913746	-3505.7079052	-3505.7295104
**36iβ**	-1874.8961041	-1874.9089049	-1874.9253841
**36iα**	-1874.8986682	-1874.911417	-1874.9278319
**MeCN**	-132.7794377	-132.7830016	-132.7872550
**$^{-}$OTf**	-961.8099933	-961.8150779	-961.8212866
**S9**	-346.8014448	-346.8065795	-346.8128453
**TfOH**	-962.2200518	-962.2254561	-962.2320705
**(*p*-Tol)$_2$SO**	-1015.3574194	-1015.3647046	-1015.3738369
**TS1**	-2969.8732014	-2969.8890643	-2969.9096697
**TS2**	-2676.6155262	-2676.6312120	-2676.6516488
**49**	-2521.7397694	-2521.7553672	-2521.7759529
**50α**	-2654.5226038	-2654.5391082	-2654.5609078
**50β**	-2654.5181296	-2654.5344880	-2654.5561365
**51**	-2584.5977885	-2584.6126451	-2584.6323135

**52α**	-2717.3811940	-2717.3969660	-2717.4178623
**52β**	-2717.3781080	-2717.3938250	-2717.4146472
**53**	-1151.1840970	-1151.1929799	-1151.2043784
**54α**	-1283.9635329	-1283.9732496	-1283.9857950
**54β**	-1283.9638094	-1283.9736698	-1283.9863716

Table S9 Coordinates of all Stationary points for the reaction, computed at B3LYP-D3(BJ)/6-311+g(d,p)/SMD level in DCM/MeCN = 1:1 solvent.

**18**			
0 1			
C	-0.91820400	-1.33243600	-0.09442300
O	-0.34058600	-0.19221400	-0.66979700
C	0.12170100	-2.43695100	0.15561800
C	0.77762400	0.37127900	0.01847600
H	-0.32845900	-3.26747400	0.69588400
H	0.45930600	-2.80336300	-0.81681800
C	1.31297000	-1.88599200	0.92316300
C	1.88880000	-0.64962500	0.24027700
H	0.45983200	0.73503100	1.00040500
C	1.22973300	1.53812600	-0.85903000
H	1.04010100	-1.61399800	1.94288700
H	2.67753000	-0.21064800	0.84479400
H	1.46674200	1.16899400	-1.85631200
C	0.19569800	2.64120900	-0.99127100
H	-0.73460100	2.22788400	-1.37628400
H	0.55602700	3.42450500	-1.65470700
C	-2.00026900	-1.78875700	-1.08348400
O	-2.37862600	-1.13306000	-2.02263700
O	-2.46209100	-2.99468000	-0.75947800
C	-3.54021400	-3.53123000	-1.58218500
H	-4.35207700	-2.80255800	-1.59737200
H	-3.16286200	-3.65683500	-2.59870800
C	-3.96529200	-4.84314400	-0.96721300
H	-4.33150300	-4.69794200	0.05208800
H	-4.77319000	-5.27368900	-1.56488200
H	-3.13666100	-5.55537200	-0.94702400
C	-3.10901000	0.09544300	1.01019200
C	-4.37622300	-0.45210100	0.79000600
C	-2.90514700	1.46391800	0.81920700
C	-5.42209000	0.36418100	0.36847400
H	-4.54198700	-1.51124000	0.94632000
C	-3.95683600	2.26846400	0.39012400
H	-1.93284100	1.90051400	1.00210400
C	-5.22894000	1.73390200	0.15307300
H	-6.40248600	-0.06981500	0.20249600
H	-3.78639800	3.32965300	0.24045900
C	-6.35282400	2.60420900	-0.34312100
H	-7.32379100	2.22122800	-0.02128300

H	-6.24681100	3.63114600	0.01369700
H	-6.35853900	2.63639100	-1.43847400
S	-1.76659400	-0.95646200	1.55255400
O	2.30166500	-2.93502700	0.96887900
O	2.42512100	-1.02036400	-1.04771800
O	2.42391300	2.05279700	-0.23478200
O	-0.11174500	3.19815700	0.30373500
C	3.19813900	-2.91040100	1.98467600
O	3.16391200	-2.09383700	2.87676900
C	3.76696200	-1.15838200	-1.16736200
O	4.54002400	-0.97698700	-0.25460200
C	3.38872300	2.56752200	-1.03407800
O	3.30785600	2.58369300	-2.24089800
C	0.50153500	4.34592000	0.67465800
O	1.24514500	4.97120100	-0.04677100
C	0.12956200	4.71335800	2.07996200
H	0.56409500	3.98062300	2.76606500
H	0.51018400	5.70471900	2.31827000
H	-0.95458500	4.68395200	2.20622400
C	4.52535300	3.10267700	-0.21902500
H	4.83170700	2.37331900	0.53293500
H	5.36255100	3.35124900	-0.86849100
H	4.19100100	4.00267400	0.30469700
C	4.13397900	-1.55647000	-2.56447000
H	3.72245500	-0.83729400	-3.27624100
H	5.21649700	-1.60443200	-2.66465400
H	3.69770600	-2.53342700	-2.78910200
C	4.20446000	-4.01025900	1.83516300
H	4.81963000	-3.81229100	0.95328600
H	4.83822100	-4.05528900	2.71876900
H	3.70084100	-4.96701200	1.68206200
**31**			
1 1			
C	1.27694000	-0.20968300	-0.53290100
C	0.69756900	-1.36487200	0.01691500
C	2.63830000	-0.00699700	-0.67391900
C	1.55448400	-2.37024100	0.45667900
C	3.47287100	-1.02663600	-0.21346700
H	3.04209100	0.89377300	-1.11704300
C	2.93355500	-2.18826300	0.34289300
H	1.15053300	-3.28119300	0.88094700
H	4.54599900	-0.91166300	-0.29833800

H	3.59958500	-2.96926600	0.68901100
C	-1.29926900	-0.14652700	-0.50915200
C	-2.65187000	0.10816700	-0.65509800
C	-0.76631200	-1.33137800	0.02435700
C	-3.52613000	-0.88418300	-0.20859500
H	-3.01998400	1.02782400	-1.09111100
C	-1.66251700	-2.30575900	0.45344900
C	-3.03321800	-2.07105400	0.33740800
H	-4.59387200	-0.72912900	-0.29751800
H	-1.29436400	-3.23558100	0.86797900
H	-3.72896600	-2.83130000	0.67097400
S	0.01148900	0.95781800	-1.03148400
C	0.06116400	2.17684100	0.44745200
F	-1.05578500	2.88691700	0.41244900
F	1.11321400	2.96181200	0.26961800
F	0.15832100	1.53293200	1.59379000
**·CF_3**			
0 2			
C	0.00000000	0.00000000	0.32782200
F	0.00000000	1.26235100	-0.07284900
F	1.09322800	-0.63117500	-0.07284900
F	-1.09322800	-0.63117500	-0.07284900
**$R_1S^{·+}$**			
1 2			
C	1.25535400	-0.71388700	0.00016400
C	0.73097700	0.60354900	-0.00022000
C	2.63118600	-0.98399100	0.00031400
C	1.61931200	1.66459400	-0.00028700
C	3.50184300	0.10042500	0.00040300
H	3.00122700	-2.00172200	0.00023600
C	3.00302200	1.40496500	0.00012000
H	1.25913100	2.68574500	-0.00043700
H	4.57055300	-0.07089700	0.00050500
H	3.69294000	2.23998400	0.00009900
C	-1.25535400	-0.71388700	0.00016500
C	-2.63118400	-0.98399200	0.00031400
C	-0.73097800	0.60354800	-0.00022000
C	-3.50184300	0.10042400	0.00040400
H	-3.00122500	-2.00172300	0.00023600
C	-1.61931400	1.66459300	-0.00028800
C	-3.00302500	1.40496300	0.00011900
H	-4.57055400	-0.07090100	0.00050500

H	-1.25913400	2.68574300	-0.00043800
H	-3.69294300	2.23998200	0.00009800
S	0.00000200	-1.91337700	-0.00042100
**47**			
0 1			
C	1.26039400	-0.73127500	0.00026300
C	0.72656600	0.57566100	0.00035300
C	2.63597200	-0.96049500	-0.00011600
C	1.60576600	1.66654900	0.00012200
C	3.48900400	0.13859600	-0.00031600
H	3.03383900	-1.96829500	-0.00062100
C	2.97717500	1.44458100	-0.00018900
H	1.21333000	2.67702300	0.00014300
H	4.56108600	-0.02038300	-0.00062200
H	3.65811100	2.28768100	-0.00020800
C	-1.26039400	-0.73127500	0.00026300
C	-2.63597200	-0.96049500	-0.00011600
C	-0.72656600	0.57566100	0.00035300
C	-3.48900400	0.13859600	-0.00031500
H	-3.03383900	-1.96829500	-0.00062100
C	-1.60576600	1.66654900	0.00012200
C	-2.97717500	1.44458100	-0.00018900
H	-4.56108600	-0.02038300	-0.00062200
H	-1.21333000	2.67702300	0.00014200
H	-3.65811000	2.28768100	-0.00020800
S	0.00000000	-1.97221500	0.00007600
**32**			
0 2			
C	-0.94415900	-1.50453000	-0.35801300
O	-0.37361500	-0.42756200	-1.04605800
C	0.09929000	-2.56847900	0.01748100
C	0.74212500	0.21054200	-0.42118000
H	-0.35023500	-3.33883500	0.64118500
H	0.44799800	-3.03590600	-0.90662500
C	1.27835800	-1.92680100	0.73139800
C	1.85547700	-0.77355200	-0.08256000
H	0.41668100	0.67581900	0.51189700
C	1.18923800	1.28112100	-1.41534300
H	0.99053700	-1.53894700	1.70890900
H	2.63844300	-0.26685500	0.47426500
H	1.39082700	0.81613400	-2.37967500
C	0.16900400	2.38722500	-1.61472500

H	-0.78430300	1.95917700	-1.91921400
H	0.51332100	3.09583800	-2.36474000
C	-2.02296400	-2.06828200	-1.29397200
O	-2.40046600	-1.51780800	-2.29860600
O	-2.48199400	-3.23289700	-0.84167400
C	-3.55451800	-3.86195800	-1.60474700
H	-4.36719000	-3.14149200	-1.70799100
H	-3.17003000	-4.10154900	-2.59786500
C	-3.98191500	-5.09613400	-0.84705400
H	-4.35397900	-4.83642100	0.14709200
H	-4.78632100	-5.59266200	-1.39627400
H	-3.15323900	-5.80071700	-0.74155100
C	-3.10378000	0.08060600	0.60054100
C	-4.38527100	-0.44186400	0.40295200
C	-2.85183000	1.42369600	0.31370200
C	-5.39786300	0.37433700	-0.09266400
H	-4.58794600	-1.48076400	0.63422000
C	-3.87095600	2.22773900	-0.18798200
H	-1.86963700	1.84204300	0.48146000
C	-5.15679800	1.71787900	-0.40408500
H	-6.38986800	-0.03899500	-0.24109000
H	-3.66396400	3.26930600	-0.41093300
C	-6.24540800	2.58477600	-0.97836100
H	-7.23176700	2.26116300	-0.63868100
H	-6.10643100	3.63207400	-0.70104300
H	-6.24079600	2.53152700	-2.07288200
S	-1.80624200	-0.96959900	1.24073500
C	-0.05107100	1.06890000	3.06065400
F	-0.99171200	1.99001700	3.23368500
F	-0.05283800	0.20241800	4.06725600
F	1.14554800	1.63894900	2.94107200
O	2.27067200	-2.95672500	0.91142900
O	2.39928100	-1.28089100	-1.31947500
O	2.40767700	1.83208100	-0.87600500
O	-0.07209000	3.07187000	-0.36683300
C	3.16212000	-2.79760500	1.92006700
O	3.11981200	-1.87243300	2.69832200
C	3.74171600	-1.43457200	-1.41417000
O	4.50867900	-1.16240200	-0.51921000
C	3.35519700	2.25283500	-1.74792500
O	3.24198100	2.14740500	-2.94758600
C	0.58593000	4.23103700	-0.13215500

O	1.31157400	4.76397400	-0.94097600
C	0.30109200	4.73755900	1.24989700
H	0.92195000	4.17951100	1.95682700
H	0.55493500	5.79437600	1.31474100
H	-0.74335900	4.57758500	1.51902100
C	4.51531900	2.86008400	-1.02175200
H	4.79913600	2.24506900	-0.16640600
H	5.35685700	2.97906200	-1.70177700
H	4.21369900	3.84160100	-0.64436600
C	4.11751600	-1.97483600	-2.76037700
H	3.73507200	-1.31675400	-3.54412600
H	5.19980900	-2.05748200	-2.83782400
H	3.65938500	-2.95764600	-2.89878200
C	4.16602400	-3.90970200	1.92600700
H	4.71438300	-3.91154000	0.98099000
H	4.85884600	-3.77839300	2.75492600
H	3.65473300	-4.87122200	2.01597200
**18**$^{\cdot+}$			
1 2			
C	-0.27037800	-1.70149900	-0.28209300
O	-0.12686000	-0.46107400	-0.85953500
C	1.05163100	-2.32049600	0.19874300
C	0.61627000	0.51744400	-0.10573000
H	0.86441500	-3.21966300	0.78533400
H	1.60150200	-2.61372100	-0.69921900
C	1.87703900	-1.31725900	0.99201700
C	2.00467200	0.00900500	0.24610500
H	0.08713200	0.73110900	0.82744800
C	0.62834900	1.77363000	-0.97358000
H	1.43189600	-1.11362200	1.96736700
H	2.51593800	0.74134500	0.86570100
H	1.14210500	1.57508600	-1.91332800
C	-0.75812500	2.30345500	-1.28060800
H	-1.28975300	1.60588600	-1.92637000
H	-0.68871900	3.27303900	-1.76994800
C	-0.94711100	-2.63047300	-1.31532600
O	-1.15233400	-2.30793200	-2.45662100
O	-1.22112100	-3.80036700	-0.76445900
C	-1.84758600	-4.81783900	-1.61821900
H	-2.76109400	-4.38744200	-2.03011400
H	-1.15586800	-5.03731000	-2.43247800
C	-2.11875000	-6.02204100	-0.75169200

H	-2.79833300	-5.77255100	0.06653300
H	-2.58690800	-6.79802200	-1.36289000
H	-1.19247800	-6.42516200	-0.33572300
C	-2.78619300	-0.69723200	0.82307100
C	-3.65775000	-0.36492400	1.90388100
C	-3.10477200	-0.27718100	-0.49666800
C	-4.78363700	0.38047100	1.66603800
H	-3.41701600	-0.69037600	2.90874000
C	-4.24166600	0.45599000	-0.70991600
H	-2.45860100	-0.51429100	-1.32568500
C	-5.10225200	0.81496000	0.35871700
H	-5.43634400	0.64934500	2.48715600
H	-4.48573200	0.78200000	-1.71395600
C	-6.30973000	1.64019300	0.09355700
H	-6.84493400	1.88813300	1.00925600
H	-6.02925400	2.56282300	-0.42683900
H	-6.98651300	1.10327400	-0.58240700
S	-1.40372300	-1.61470900	1.27095100
O	3.17138000	-1.91167200	1.18104300
O	2.73809000	-0.19417500	-0.97418700
O	1.36148100	2.75651400	-0.21586900
O	-1.51319700	2.41270300	-0.05730900
C	3.89863500	-1.50283700	2.25431500
O	3.48023900	-0.71070800	3.06569900
C	4.02828800	0.23090300	-1.02085700
O	4.57099300	0.79465700	-0.09995800
C	2.37598500	3.42052500	-0.83198900
O	2.69324700	3.22114000	-1.98074900
C	-1.93226400	3.63146700	0.35151500
O	-1.72894300	4.65225200	-0.26581400
C	-2.70185700	3.52813900	1.63464600
H	-2.27276800	2.77053900	2.29057300
H	-2.72155400	4.49748000	2.13029600
H	-3.72849900	3.23045300	1.40147400
C	3.01115700	4.39890800	0.10568100
H	3.37656900	3.87561600	0.99237700
H	3.83426500	4.90754600	-0.39197200
H	2.26532200	5.12766800	0.43313500
C	4.64816700	-0.10763900	-2.34011500
H	3.99221300	0.19768500	-3.15727600
H	5.61707300	0.37951900	-2.42971000
H	4.77716300	-1.19219000	-2.40222900

C	5.24093500	-2.16514500	2.27064100
H	5.79905800	-1.87658600	1.37634100
H	5.79021100	-1.86522200	3.16080700
H	5.12095500	-3.25087900	2.25165500
**48**			
0 1			
C	-0.27886900	-0.03477700	-0.65169500
C	-0.94890900	-1.23386600	-0.40574100
C	-0.93963600	1.18649500	-0.49441500
C	-2.27753400	-1.20469500	0.01002400
H	-0.43837600	-2.18024100	-0.53438900
C	-2.26582800	1.19925300	-0.07724700
H	-0.42049400	2.11595900	-0.69320300
C	-2.95600200	0.00706600	0.17961900
H	-2.79389600	-2.13822400	0.20468500
H	-2.77405100	2.14915200	0.04934000
C	-4.39972600	0.03558200	0.60300000
H	-4.71376500	-0.92788300	1.00848400
H	-4.57341700	0.80599400	1.35863900
H	-5.04549700	0.26718300	-0.25091600
S	1.41957200	-0.06411200	-1.23215300
C	2.28564400	0.01848300	0.36466600
F	2.00689700	-1.01779100	1.18358600
F	3.61318900	0.00023500	0.12623300
F	2.01674800	1.13895500	1.06824100
**·33**			
0 2			
C	-1.34684800	-1.65248600	-0.77605000
C	-0.40429200	-2.55263500	0.01037600
C	0.92930400	-1.90826000	0.19572900
C	0.09149600	0.30094400	-0.24999600
C	-1.33109200	-0.23166100	-0.22201800
H	-0.84755200	-2.74861300	0.99635300
H	-0.28218800	-3.51287700	-0.48859600
H	-1.06249000	-1.61114900	-1.82779500
H	0.39340200	0.39485700	-1.29626000
H	-1.96869800	0.41745800	-0.81695300
O	1.03213300	-0.58151100	0.40055500
O	-1.79671300	-0.24542200	1.14200500
O	-2.66746500	-2.21705700	-0.65929300
C	-3.05091700	0.20871300	1.39168100
O	-3.77749900	0.65173700	0.53261800

C	-3.38764900	0.06691200	2.84333800
H	-3.52382600	-0.99473500	3.07053200
H	-2.57075500	0.43894000	3.46376400
H	-4.30757100	0.60504400	3.06390100
C	0.24733600	1.64440800	0.46165300
H	-0.07128800	1.55148800	1.49904200
C	1.66571900	2.17009200	0.45645600
H	1.68855000	3.18700700	0.85247000
H	2.29697800	1.53269800	1.07385200
C	-3.57377500	-1.90258000	-1.61632200
O	-0.62978000	2.56048400	-0.22625900
O	2.15151600	2.16824700	-0.90185200
C	3.47878100	2.32851900	-1.07769100
C	-1.53025500	3.26474300	0.50758400
O	4.24860800	2.50889000	-0.15974500
O	-1.61851800	3.17759600	1.71020000
O	-3.30742400	-1.21817300	-2.57788600
C	-4.90889100	-2.50839300	-1.30780700
H	-4.80373400	-3.57836600	-1.11607300
H	-5.31152500	-2.04609600	-0.40248700
H	-5.59248100	-2.34057900	-2.13782300
C	-2.36453100	4.13897400	-0.37658200
H	-1.72415000	4.87854000	-0.86484600
H	-2.83388400	3.53778000	-1.15831900
H	-3.12534500	4.64422600	0.21498400
C	2.13133800	-2.68945000	0.36504000
O	2.14582700	-3.91668100	0.30665800
O	3.22837700	-1.93674100	0.58547000
C	4.48343300	-2.64440600	0.74789200
H	4.67240100	-3.23443900	-0.15187700
H	4.39506200	-3.32781200	1.59549200
C	5.55941800	-1.60653900	0.97325400
H	6.52417100	-2.10621800	1.09670300
H	5.63163900	-0.92475500	0.12199400
H	5.35792100	-1.02140000	1.87414500
C	3.84651600	2.24141400	-2.52909800
H	4.90719900	2.44920400	-2.65609100
H	3.62140800	1.23774200	-2.90007600
H	3.25369900	2.95124600	-3.11043800
**33**			
1 1			
C	-1.56134500	-1.35719000	-1.13470600

C	-0.65473400	-2.46538800	-0.62706000
C	0.69463600	-2.00473900	-0.26641300
C	0.12916700	0.35170500	-0.49644100
C	-1.31453200	-0.06835900	-0.35359100
H	-1.04884200	-2.92521200	0.29053800
H	-0.55254200	-3.29251600	-1.33717100
H	-1.39066300	-1.17243700	-2.19357700
H	0.35941300	0.59921200	-1.52945700
H	-1.94329600	0.73437800	-0.74385700
O	1.05150300	-0.80479200	-0.20256900
O	-1.58296600	-0.29593200	1.03408300
O	-2.89952900	-1.80714900	-0.91676200
C	-2.73681600	0.23201300	1.54941400
O	-3.49195800	0.91170700	0.90100700
C	-2.89739700	-0.18021400	2.98545800
H	-3.18609300	-1.23069900	3.01301100
H	-1.95913400	-0.06758700	3.51815000
H	-3.67746200	0.42539700	3.45101600
C	0.58876500	1.44500000	0.46527900
H	0.42867300	1.11825600	1.49258500
C	2.03956300	1.83693600	0.31360500
H	2.24453500	2.71776300	0.92687400
H	2.69308600	1.02817600	0.63289100
C	-3.87899400	-1.24661500	-1.68893500
O	-0.27416400	2.55496100	0.17041300
O	2.27000900	2.14232700	-1.07428800
C	3.52117800	2.52959300	-1.40954700
C	-0.95930200	3.12858400	1.20606600
O	4.42551400	2.57886900	-0.60741600
O	-0.87089800	2.74660700	2.34345600
O	-3.64047300	-0.41570800	-2.53096300
C	-5.22076500	-1.78431900	-1.32276200
H	-5.23016700	-2.87155100	-1.42013600
H	-5.43380800	-1.55264300	-0.27609300
H	-5.98449400	-1.34548800	-1.96143000
C	-1.78030900	4.27970100	0.71531300
H	-1.12228600	5.06896000	0.34145800
H	-2.41179900	3.95262100	-0.11322900
H	-2.39319500	4.66515700	1.52743100
C	1.77145800	-3.03630800	0.06100900
O	1.62856900	-4.18370100	-0.27671400
O	2.76956100	-2.48254600	0.69830500

C	3.90304100	-3.35557200	1.07514400
H	4.28387700	-3.80327200	0.15195200
H	3.50839600	-4.14561300	1.71490300
C	4.92004200	-2.49184800	1.76424100
H	5.77286000	-3.11587800	2.04371100
H	5.27772600	-1.69502300	1.10838600
H	4.50478400	-2.05051400	2.67292400
C	3.61011800	2.94333900	-2.84471800
H	4.69134600	2.68458800	-3.21073900
H	2.72379200	2.41413600	-3.51785600
H	3.42131500	4.16475300	-2.86882900
**34α**			
1 1			
C	1.83594400	-0.80916100	-0.12351000
C	-0.39926300	-0.17613200	0.47985700
C	-0.10010000	1.29907700	0.25540800
C	1.37524500	1.56555800	0.54730200
C	2.26886300	0.65681800	-0.28388500
H	-0.72796000	1.89869400	0.90960100
H	1.54486000	1.41856800	1.61471600
H	2.16579600	0.90045700	-1.34288700
H	3.31408300	0.76721400	0.00250200
O	0.48500400	-1.02308700	-0.28873300
C	2.58044100	-1.69977900	-1.16152600
O	3.83681200	-1.86422900	-0.79576000
O	2.04808500	-2.10417900	-2.15834700
C	4.71482700	-2.60604300	-1.71351700
H	4.70475400	-2.08438900	-2.67113000
H	4.29170400	-3.60274800	-1.84215600
C	6.08709000	-2.63949200	-1.08962000
H	6.76185000	-3.18588800	-1.75375800
H	6.48285300	-1.63031100	-0.95342900
H	6.06868400	-3.15032500	-0.12402200
O	1.71979500	2.91374600	0.19149400
C	1.49248900	3.88513700	1.11511600
O	1.06117600	3.65072100	2.21962400
C	1.84762600	5.23234000	0.56807000
H	1.18278100	5.46916400	-0.26670600
H	2.87084700	5.22425400	0.18595200
H	1.74262500	5.98642400	1.34560300
O	-0.37914100	1.62257500	-1.11712300
C	-1.04118100	2.78172700	-1.37367900

O	-1.43498500	3.52164400	-0.50284900
C	-1.18127700	3.00008300	-2.84684600
H	-1.44042400	2.07018600	-3.35380000
H	-0.21803700	3.34130100	-3.23918900
H	-1.93459300	3.76243500	-3.03639200
C	-1.80776900	-0.58367600	0.04329700
H	-1.90738400	-0.46304300	-1.03396100
O	-2.72402000	0.30351100	0.71220600
C	-3.61228600	1.01355200	-0.03493800
O	-3.65227100	0.96778400	-1.24094800
C	-4.50533000	1.82831400	0.84827500
H	-3.91109700	2.39730300	1.56577600
H	-5.15726100	1.15592300	1.41335300
H	-5.11073000	2.49969400	0.24259200
C	-2.12299900	-2.00472400	0.45742000
H	-1.37923700	-2.69117800	0.05246000
H	-2.14524800	-2.09518900	1.54523200
O	-3.42099200	-2.31724100	-0.08328900
C	-3.92115900	-3.53538000	0.20849200
O	-3.32106700	-4.34787000	0.87786000
C	-5.28259900	-3.72098800	-0.39112600
H	-5.25119900	-3.51680200	-1.46365600
H	-5.97906000	-3.00999500	0.06160000
H	-5.63014600	-4.73696200	-0.21402100
H	-0.26442900	-0.38934600	1.54622700
C	2.50881700	-1.63652300	2.26722900
C	2.87433200	-2.08761500	3.57394200
H	2.60231600	-3.14159300	3.67498400
H	3.95450800	-1.96465200	3.69472900
H	2.34438000	-1.48751900	4.31859200
N	2.22961800	-1.27246200	1.21907400
**34’α**			
0 1			
C	-1.43526100	-1.68162100	0.88725600
C	-0.55278800	0.46372000	0.26431000
C	-1.79049300	0.79804700	-0.55092100
C	-2.37743800	-0.49191100	-1.11695900
C	-2.67353100	-1.48694300	-0.00494500
H	-1.52197800	1.47604000	-1.35683800
H	-1.67081200	-0.90149700	-1.83985700
H	-3.46660900	-1.09808200	0.63642900
H	-2.98949700	-2.44676300	-0.41170900

O	-0.84504700	-0.50708000	1.30006400
C	-1.83372300	-2.47446000	2.16710800
O	-1.99865500	-3.75210100	1.87942600
O	-2.00535900	-1.93852800	3.22781600
C	-2.44597600	-4.63244600	2.96772700
H	-3.40969000	-4.26066600	3.31776700
H	-1.71724000	-4.55581000	3.77533000
C	-2.53630300	-6.02854600	2.40494900
H	-2.86868300	-6.70616300	3.19569900
H	-3.25732900	-6.07749500	1.58555500
H	-1.56323500	-6.37160800	2.04562200
O	-3.63019400	-0.23053600	-1.77251700
C	-3.58441000	0.20746400	-3.05806300
O	-2.55086300	0.33178800	-3.67213600
C	-4.96083900	0.49829600	-3.57074700
H	-5.39430900	1.32036000	-2.99538300
H	-5.60330000	-0.37515500	-3.43829100
H	-4.91276300	0.77228700	-4.62279100
O	-2.75998600	1.42376800	0.31013200
C	-3.42982800	2.50447100	-0.16342300
O	-3.22938600	2.98000800	-1.25698200
C	-4.43567100	2.98644400	0.83455500
H	-4.02068300	2.96716800	1.84270400
H	-5.29793200	2.31253700	0.81144100
H	-4.76128500	3.99185400	0.57407000
C	0.03007900	1.66526100	1.01044000
H	-0.67163300	2.00781000	1.76894000
O	0.22238000	2.70729400	0.03315300
C	-0.36110400	3.91611700	0.23656300
O	-1.06833600	4.16505400	1.18446800
C	0.00391900	4.86460800	-0.86362300
H	-0.15941100	4.39645800	-1.83614300
H	1.06749500	5.10785000	-0.78556900
H	-0.58596800	5.77508100	-0.77823900
C	1.37159300	1.33251300	1.62731200
H	1.27744800	0.47507100	2.29399600
H	2.11057000	1.10919900	0.85812300
O	1.78077500	2.49392200	2.37917400
C	2.97153500	2.42012500	3.00519900
O	3.66724800	1.42797900	2.98251300
C	3.29974800	3.70721000	3.70219600
H	2.48085400	3.99642500	4.36458000

H	3.42168700	4.50018400	2.95927500
H	4.22021700	3.59575100	4.27210300
H	0.20222700	0.04525700	-0.40568800
C	0.35580100	-3.09013000	-0.39438900
C	1.30884400	-3.90984700	-1.07481900
H	2.16009500	-4.08310900	-0.41188700
H	0.82740400	-4.85960500	-1.32522400
H	1.64720000	-3.39364600	-1.97858400
N	-0.44958300	-2.49123800	0.15638800
C	4.66061400	-1.02081000	-1.24873400
S	2.92996700	-0.71810400	-1.91530400
O	2.97434800	0.67971000	-2.37190300
O	2.79145700	-1.73617100	-2.97697100
O	2.08301600	-0.96743600	-0.72498800
F	5.58438700	-0.85496900	-2.21029000
F	4.94549800	-0.17100000	-0.24859800
F	4.77733300	-2.27382700	-0.77458000
**34β**			
1 1			
C	1.25411000	1.82715900	0.68021700
C	-0.25958200	0.17792800	-0.29709500
C	0.76496300	-0.92861000	-0.10206300
C	2.16975900	-0.34615200	-0.22326200
C	2.37803900	0.77675900	0.78387300
H	0.61131000	-1.69765600	-0.85468800
H	2.31720400	-0.00443300	-1.24523000
H	2.34943200	0.36073000	1.79137700
H	3.34107800	1.26599900	0.63291800
O	-0.04075900	1.28229500	0.61282400
C	1.48862300	2.87599100	-0.45263900
O	1.45218600	2.26573200	-1.62079800
O	1.66305400	4.04988900	-0.25112400
C	1.63077300	3.08922000	-2.82859500
H	2.60456300	3.57385500	-2.75333900
H	0.84801700	3.84829500	-2.82659300
C	1.53582900	2.16244800	-4.01428200
H	1.66995800	2.74700400	-4.92816000
H	2.31431500	1.39680400	-3.97981700
H	0.55844400	1.67673300	-4.05861900
O	3.15320100	-1.34691000	0.09245200
C	3.51816800	-2.19629300	-0.90430200
O	3.08737500	-2.10845500	-2.03014800

C	4.49216400	-3.21740300	-0.40459400
H	3.98910700	-3.86968100	0.31442300
H	5.32113200	-2.72893800	0.11173500
H	4.86429400	-3.81160300	-1.23694800
O	0.59643800	-1.49204600	1.21173400
C	0.64761300	-2.84411700	1.33468600
O	0.79158400	-3.58694200	0.39211500
C	0.52403300	-3.25369600	2.76834400
H	-0.23133300	-2.65550100	3.27853500
H	1.48522500	-3.07934600	3.26241600
H	0.27925900	-4.31254600	2.82913500
C	-1.69530100	-0.27222500	-0.01336000
H	-1.80238300	-0.52614200	1.03953300
O	-1.92740200	-1.44719500	-0.81444000
C	-2.34299300	-2.58704600	-0.19983200
O	-2.49541900	-2.68079400	0.99471600
C	-2.58121100	-3.66881000	-1.20695800
H	-1.73346800	-3.74575100	-1.88998800
H	-3.46532400	-3.41376900	-1.79863600
H	-2.74522600	-4.61694600	-0.69864500
C	-2.69819200	0.78699100	-0.41692900
H	-2.48457300	1.72735300	0.09198000
H	-2.67867100	0.94785200	-1.49642700
O	-3.99472000	0.29988700	-0.01953700
C	-5.05653200	1.06723000	-0.33958700
O	-4.95037500	2.12623200	-0.91872700
C	-6.33892900	0.43274000	0.10911500
H	-6.29865000	0.22670800	1.18129900
H	-6.47417500	-0.52160400	-0.40667200
H	-7.17601000	1.09193800	-0.11231700
H	-0.17789100	0.53457600	-1.32607200
C	1.24801400	3.19117900	2.87579700
C	1.24926200	3.95645800	4.08257300
H	1.73981600	4.91433100	3.88694900
H	0.21361000	4.12236000	4.39258000
H	1.79086500	3.40390900	4.85481900
N	1.24530100	2.58191500	1.90932300
**35α**			
1 1			
C	-0.50049500	-1.19247900	-1.11094900
C	1.39501600	-0.04190600	-0.13926800
C	2.01318300	-1.36393200	0.29185800

C	0.90801400	-2.37668500	0.57721700
C	0.02027100	-2.54857200	-0.64375700
H	2.62104700	-1.20249100	1.17833800
H	0.33807600	-2.03600000	1.44121100
H	0.59544600	-2.97651700	-1.46496600
H	-0.82330300	-3.20463600	-0.42864600
O	0.46858700	-0.20755600	-1.24128400
C	-1.15247600	-1.30921100	-2.51029700
O	-2.39707800	-0.86177800	-2.50832600
O	-0.55419500	-1.75849900	-3.45360700
C	-3.13764300	-0.88216400	-3.77701100
H	-3.21215800	-1.92288400	-4.09425300
H	-2.55315000	-0.32409400	-4.50898700
C	-4.48716800	-0.25974500	-3.51772200
H	-5.06169600	-0.27117300	-4.44760600
H	-5.04212100	-0.81974700	-2.76251100
H	-4.38648900	0.77676800	-3.18932000
O	1.47786300	-3.66730200	0.85904700
C	1.89589200	-3.90402000	2.12875200
O	1.76468900	-3.10156800	3.02389300
C	2.51825500	-5.26136100	2.24032400
H	3.41319700	-5.30070400	1.61405600
H	1.82438100	-6.02267200	1.87654000
H	2.78519600	-5.46183900	3.27603600
O	2.83792700	-1.85802000	-0.77943900
C	4.05044900	-2.38012500	-0.46235800
O	4.48375800	-2.40521700	0.66615800
C	4.73305700	-2.91913300	-1.67986300
H	4.59747100	-2.24885700	-2.52891800
H	4.27811900	-3.88193000	-1.93355000
H	5.79150600	-3.06752000	-1.47388700
C	2.43370300	0.96113500	-0.64827800
H	2.88668600	0.58315900	-1.56282100
O	3.44276100	1.07404900	0.37521800
C	4.74400800	0.86300000	0.04134700
O	5.10373200	0.54600900	-1.06712400
C	5.63236900	1.08768800	1.22568500
H	5.23978700	0.56777700	2.10126000
H	5.65516700	2.15740600	1.45383300
H	6.63997700	0.74343100	1.00110500
C	1.82983300	2.32973100	-0.86997600
H	0.95947000	2.26517300	-1.52347100

H	1.53903900	2.77779600	0.07971400
O	2.84628500	3.13853100	-1.49464700
C	2.55613600	4.44406000	-1.66671100
O	1.50035100	4.93219200	-1.32602300
C	3.68828500	5.17074700	-2.32854400
H	3.81763100	4.78894000	-3.34504900
H	4.61945700	4.99232100	-1.78656900
H	3.47335200	6.23701100	-2.36432300
H	0.86110700	0.37266000	0.72109700
S	-2.20518900	0.66137600	-0.20213100
O	-1.47265000	-0.82284000	-0.07949400
C	-3.90800300	0.17661700	0.07387100
C	-4.26465300	-1.13935900	0.33786400
C	-4.84790800	1.19924600	-0.01984400
C	-5.61329500	-1.42854600	0.52009400
H	-3.51890900	-1.91883700	0.39552400
C	-6.18833900	0.88313800	0.16822900
H	-4.55265300	2.22053400	-0.23304300
C	-6.59190300	-0.43088200	0.43678100
H	-5.90737900	-2.45091700	0.72910900
H	-6.93089400	1.66968400	0.09975700
C	-1.71555200	1.41157800	1.31911900
C	-1.05124800	2.63253000	1.21554000
C	-1.95240300	0.79724800	2.55338300
C	-0.61080600	3.25154700	2.38129200
H	-0.88297400	3.09277500	0.24994400
C	-1.49130100	1.42509600	3.69655500
H	-2.48101600	-0.14563000	2.61438100
C	-0.81521100	2.65787700	3.63020300
H	-0.09664100	4.20302300	2.31567000
H	-1.65839700	0.96115400	4.66208000
C	-0.32405300	3.31233200	4.88896600
H	0.42996700	2.68538800	5.37542800
H	0.11811400	4.28812600	4.68469500
H	-1.14325700	3.44121400	5.60230100
C	-8.04760300	-0.77008900	0.60049500
H	-8.62286400	0.09915600	0.92454300
H	-8.46755300	-1.10989000	-0.35258700
H	-8.18642800	-1.57535500	1.32504000
**35β**			
1 1			
C	0.49476200	-1.66411000	-0.47028100

C	-1.57989400	-0.35486700	-0.82563500
C	-2.26674800	-1.14420400	0.27809900
C	-1.73789500	-2.57441300	0.28501800
C	-0.23108900	-2.59034400	0.50868400
H	-3.34132500	-1.13500100	0.11466600
H	-2.01454400	-3.05414700	-0.65184400
H	-0.01987000	-2.22692800	1.51464100
H	0.16263500	-3.60272300	0.41369400
O	-0.14815300	-0.44180500	-0.72665000
C	0.83871500	-2.33799900	-1.82104600
O	-0.27605000	-2.67511400	-2.44838000
O	1.96453100	-2.49646700	-2.22700200
C	-0.15335400	-3.29619100	-3.77287500
H	0.44028100	-4.20406600	-3.66007700
H	0.38004600	-2.59717900	-4.41831200
C	-1.55138100	-3.58045300	-4.26333400
H	-1.49189900	-4.04502400	-5.25103300
H	-2.07211600	-4.26712600	-3.59182700
H	-2.13258600	-2.65957900	-4.35177500
O	-2.30811000	-3.30877800	1.38502900
C	-3.54269700	-3.84383400	1.20641900
O	-4.15145800	-3.75884000	0.16498900
C	-4.00769500	-4.53709300	2.44970600
H	-4.00259400	-3.83604500	3.28742200
H	-3.32122300	-5.35204300	2.69362300
H	-5.01085000	-4.93137200	2.30008100
O	-1.95975200	-0.52725000	1.54273100
C	-2.96536800	-0.36214200	2.43846200
O	-4.10952200	-0.68262200	2.21518700
C	-2.44916000	0.23759300	3.70900100
H	-1.72684700	1.02643500	3.49785300
H	-1.93910500	-0.54497400	4.27962100
H	-3.27864100	0.62488100	4.29780200
C	-1.85985400	1.14868100	-0.74474500
H	-1.46931200	1.53625000	0.19360900
O	-3.29148500	1.31692800	-0.77264700
C	-3.88689100	2.01006800	0.23364700
O	-3.28141700	2.48076000	1.16723700
C	-5.36496300	2.09539100	0.00894400
H	-5.77656900	1.09735600	-0.15518000
H	-5.56043500	2.68815300	-0.88888600
H	-5.84296300	2.56187200	0.86798000

C	-1.25173200	1.89484900	-1.91108500
H	-0.19548800	1.65201100	-2.00583000
H	-1.76383100	1.65189600	-2.84506100
O	-1.40382200	3.30025100	-1.62983300
C	-0.64733800	4.15310700	-2.34756500
O	0.11205800	3.78923800	-3.21923000
C	-0.89540200	5.57576100	-1.94335500
H	-1.05490100	5.65643600	-0.86814300
H	-1.80165800	5.92664100	-2.44746100
H	-0.05855200	6.19881500	-2.25496000
H	-1.90675000	-0.75770900	-1.78824900
S	2.58969900	-0.06778500	-0.51382500
O	1.71259100	-1.31217800	0.20033800
C	1.95799100	1.36624100	0.33642600
C	1.30988800	1.27415900	1.56589200
C	2.07077500	2.57011700	-0.35394100
C	0.76990200	2.43014500	2.11109500
H	1.20378600	0.32368000	2.06966200
C	1.53495400	3.71724500	0.22237200
H	2.55104700	2.61919600	-1.32423000
C	0.86978600	3.66423600	1.45139000
H	0.24682400	2.37092200	3.05879500
H	1.61426600	4.66034300	-0.30509200
C	4.16967700	-0.41693100	0.19131700
C	5.07188200	-1.07663100	-0.64724200
C	4.50717800	-0.05515000	1.49835800
C	6.33317000	-1.38677600	-0.15478200
H	4.79689400	-1.34053300	-1.66110700
C	5.77046700	-0.37821000	1.96588100
H	3.80798600	0.47359800	2.13159600
C	6.70019000	-1.04767300	1.15297300
H	7.04358700	-1.89394900	-0.79677400
H	6.04730300	-0.10177100	2.97682700
C	8.05916800	-1.39410200	1.68916000
H	7.97838200	-2.18894100	2.43829200
H	8.72428900	-1.73951300	0.89670100
H	8.51524400	-0.53163100	2.18220800
C	0.23156700	4.89288000	2.03565000
H	0.62087700	5.80095600	1.57305800
H	-0.85146100	4.86875200	1.87485200
H	0.39795200	4.94753600	3.11427300
**35’β**			

0 1			
C	1.18326300	1.88954700	-0.51075100
C	2.44985600	-0.16550100	-1.02495900
C	3.54418900	0.24121100	-0.05209200
C	3.68209900	1.76147400	-0.07295800
C	2.36671600	2.44213600	0.28874100
H	4.48420600	-0.22968300	-0.32859500
H	4.03455700	2.06044500	-1.05817600
H	2.15009000	2.24825400	1.33931900
H	2.44055300	3.51992100	0.14083800
O	1.21598800	0.49050800	-0.69168200
C	0.96490300	2.53252100	-1.90038600
O	2.09337500	2.52200900	-2.59353800
O	-0.10674400	2.92987700	-2.29040000
C	2.04931500	3.00906300	-3.97702900
H	1.71889700	4.04819600	-3.95311900
H	1.31275900	2.41207300	-4.51624400
C	3.43873000	2.86101100	-4.54623900
H	3.43861100	3.21949100	-5.57890700
H	4.16197900	3.45099100	-3.97820100
H	3.75483800	1.81520400	-4.54619900
O	4.63263200	2.18765600	0.92328600
C	5.94774300	2.13676100	0.59629200
O	6.34247200	1.79413300	-0.49459800
C	6.80219800	2.55281900	1.75410100
H	6.69690100	1.82084300	2.55934900
H	6.47132600	3.52000700	2.13850400
H	7.84396300	2.60794000	1.44422400
O	3.14857600	-0.18460600	1.26536200
C	4.07363200	-0.77961600	2.05837700
O	5.21849900	-0.96500400	1.71574400
C	3.46661300	-1.16810100	3.37051300
H	2.67246400	-1.89641500	3.19461200
H	3.01751600	-0.29356700	3.84631700
H	4.22767600	-1.59864300	4.01825400
C	2.09071200	-1.65260000	-0.96386400
H	1.68479700	-1.87828400	0.01936800
O	3.30393600	-2.40652900	-1.15885900
C	3.68204100	-3.29325800	-0.20008100
O	3.05494700	-3.48272200	0.81509500
C	4.95580600	-3.97730000	-0.58972700
H	5.72320800	-3.23466100	-0.81804600

H	4.78713300	-4.57070300	-1.49237500
H	5.28938800	-4.62419600	0.21913800
C	1.09420800	-2.01693300	-2.04404300
H	0.33691700	-1.23998100	-2.13950800
H	1.59467400	-2.14651300	-3.00539900
O	0.46037900	-3.25042900	-1.64537600
C	-0.59492700	-3.65100800	-2.38768500
O	-0.93988000	-3.07706700	-3.39740200
C	-1.28366900	-4.83275000	-1.77708500
H	-2.06732300	-4.44894600	-1.11529400
H	-0.59472300	-5.44143000	-1.19192700
H	-1.75525400	-5.42801500	-2.55852900
H	2.76235200	0.11342700	-2.03565300
S	-1.28159500	1.10918900	0.01202300
O	0.03368800	2.14586200	0.29417900
C	-1.03519200	-0.04079900	1.34762000
C	-0.82364600	0.41246000	2.64926500
C	-0.89631800	-1.38356000	1.00840700
C	-0.47864400	-0.51245500	3.62443800
H	-0.90854900	1.46267700	2.89611000
C	-0.55344400	-2.29264700	1.99960300
H	-1.04776500	-1.71226100	-0.00922400
C	-0.33686100	-1.87353800	3.31736400
H	-0.30354100	-0.17232000	4.63879800
H	-0.43780400	-3.33879000	1.74170300
C	-2.53018400	2.24778700	0.60018400
C	-3.51692400	1.82183900	1.48557500
C	-2.53952900	3.52774000	0.04767900
C	-4.51815600	2.71799200	1.84028200
H	-3.52705800	0.81319400	1.87319900
C	-3.54827100	4.40649000	0.42538600
H	-1.77386600	3.83308900	-0.65271400
C	-4.55091900	4.01869100	1.32252600
H	-5.28947000	2.39606900	2.53100300
H	-3.55608600	5.40923400	0.01352500
C	-5.65822100	4.96390600	1.69861400
H	-6.56079800	4.74130100	1.11896600
H	-5.91845200	4.86650900	2.75526800
H	-5.38097500	6.00047600	1.49872000
C	0.03585000	-2.86177200	4.38585100
H	-0.86197200	-3.21468100	4.90561400
H	0.53560200	-3.73400400	3.96046800

H	0.68979500	-2.40756400	5.13314300
O	-3.28498400	-0.46103600	-0.79476600
S	-4.14501100	-1.49612200	-0.16802800
O	-3.89147400	-2.87066000	-0.63328900
O	-4.34425800	-1.34014600	1.28411000
C	-5.83630100	-1.08772500	-0.88092600
F	-6.77028300	-1.93602900	-0.41647600
F	-5.83044400	-1.17209600	-2.22166800
F	-6.20794800	0.15970100	-0.54553400
**36iβ**			
0 1			
C	-1.09909000	-1.24350900	0.62165600
C	0.81942500	0.21178800	0.47415700
C	1.74767600	-0.92830900	0.07420100
C	1.21144000	-2.22781900	0.67063700
C	-0.24018900	-2.46160300	0.28245100
H	2.75507600	-0.73306900	0.43227800
H	1.32808700	-2.17154800	1.75419800
H	-0.31636700	-2.61165300	-0.79523700
H	-0.63293900	-3.33929000	0.79327300
O	-0.52964000	-0.05661500	0.08951900
C	-1.33876700	-1.12702200	2.15976500
O	-1.33080200	0.13137100	2.58141000
O	-1.55258500	-2.09296500	2.85592700
C	-1.61104600	0.36649000	3.99614600
H	-0.88249600	-0.19421400	4.58389000
H	-2.60887800	-0.01949600	4.21116300
C	-1.51136700	1.85480100	4.22996100
H	-1.72864500	2.06712200	5.28014800
H	-0.50679600	2.22170200	4.00649500
H	-2.23083800	2.39841800	3.61298900
O	1.96742500	-3.35248800	0.17693000
C	3.13315000	-3.64990800	0.79904400
O	3.53543300	-3.05231600	1.77141600
C	3.82259700	-4.79475800	0.12129000
H	4.09754900	-4.49988900	-0.89472400
H	3.14625900	-5.64937600	0.04762500
H	4.71620200	-5.07116000	0.67753300
O	1.75962900	-1.03041100	-1.36372500
C	2.94701200	-1.23489200	-1.97991200
O	4.00228200	-1.29706000	-1.39030200
C	2.76113400	-1.39268500	-3.45767600

H	2.01232300	-0.69467200	-3.83290000
H	2.40396000	-2.40882300	-3.65347900
H	3.71159900	-1.24574800	-3.96769900
C	1.17016200	1.54337800	-0.19045500
H	1.01512000	1.47093400	-1.26516800
O	2.56500800	1.80374400	0.07261300
C	3.40194000	2.01238400	-0.97488100
O	3.04957900	1.97385000	-2.13051100
C	4.79089600	2.29428300	-0.48899500
H	5.10906000	1.51906900	0.21091800
H	4.79808600	3.24864900	0.04490500
H	5.47550200	2.34201600	-1.33352700
C	0.35223000	2.67148500	0.39760700
H	-0.71074500	2.44758000	0.31371900
H	0.60565300	2.82939400	1.44717500
O	0.66293300	3.85913000	-0.36020900
C	0.09646700	5.00821400	0.05645000
O	-0.65574800	5.06504800	1.00534100
C	0.53058700	6.16749200	-0.79034600
H	0.38927900	5.93792300	-1.84849600
H	1.59669600	6.35149600	-0.62991200
H	-0.03608700	7.05650300	-0.51995600
H	0.89006800	0.32274900	1.56217500
O	-2.32941500	-1.43368400	-0.01115000
C	-3.33848400	-0.41670000	0.22733900
H	-2.87787800	0.56879300	0.15265100
H	-3.75310700	-0.54691200	1.23115300
C	-4.40742800	-0.58766300	-0.81189800
C	-5.58565100	-1.27728600	-0.51656000
C	-4.22054300	-0.07628000	-2.10065800
C	-6.56642500	-1.45292300	-1.49303100
H	-5.73455800	-1.67503700	0.48180600
C	-5.19657500	-0.25230800	-3.07839000
H	-3.30767700	0.46193800	-2.33366600
C	-6.37237500	-0.94176600	-2.77550700
H	-7.47878400	-1.98717700	-1.25275600
H	-5.04417900	0.14997400	-4.07364900
H	-7.13398400	-1.07631300	-3.53537600
**36iα**			
0 1			
C	1.28462100	-0.58084300	0.95883200
C	-0.71004800	0.19087500	-0.10252900

C	-1.20389700	-1.22564600	-0.37347200
C	-0.00226200	-2.15947200	-0.49202800
C	0.90718600	-2.04916000	0.72150200
H	-1.79388000	-1.24795800	-1.28583400
H	0.53476500	-1.91027600	-1.40609700
H	0.38768500	-2.42181800	1.60651300
H	1.81328900	-2.63497400	0.57308200
O	0.13758300	0.23823300	1.04926700
C	1.99775200	-0.44319000	2.32852100
O	3.19915500	-1.01193500	2.26997500
O	1.51941600	0.07174500	3.30912800
C	3.99943500	-0.99771300	3.49061400
H	3.44180300	-1.52123800	4.26917100
H	4.13548800	0.04135300	3.79527600
C	5.31256700	-1.67406800	3.17752500
H	5.93586400	-1.67808400	4.07574600
H	5.15704200	-2.70907000	2.86330600
H	5.85044200	-1.14212000	2.38900700
O	-0.44739500	-3.53094000	-0.56331800
C	-0.83672400	-4.00466600	-1.77061900
O	-0.77113600	-3.35504200	-2.78946300
C	-1.34362700	-5.41015000	-1.65314000
H	-2.23672600	-5.41955200	-1.02298100
H	-0.59174600	-6.04111500	-1.17360900
H	-1.58607100	-5.80092800	-2.63951900
O	-2.02365200	-1.64218500	0.73827100
C	-3.17256000	-2.30534600	0.47180700
O	-3.56261300	-2.53931100	-0.64991700
C	-3.85817400	-2.71578600	1.73864700
H	-3.78736700	-1.93020000	2.49128900
H	-3.35608900	-3.60609500	2.13073300
H	-4.89967900	-2.95716600	1.53353900
C	-1.84359200	1.17510500	0.18991300
H	-2.34237000	0.89818000	1.11656600
O	-2.78316100	1.08430400	-0.90233600
C	-4.08131000	0.79896500	-0.62932600
O	-4.50061200	0.59138400	0.48518200
C	-4.89288500	0.78734100	-1.88877300
H	-4.41053300	0.16363000	-2.64378400
H	-4.95307500	1.80500000	-2.28516900
H	-5.89496500	0.41831400	-1.67923500
C	-1.32902200	2.59543700	0.26148900

H	-0.53557900	2.66981100	1.00474600
H	-0.94932200	2.92111100	-0.70868200
O	-2.44104400	3.42854000	0.64975300
C	-2.20599500	4.75233100	0.73407900
O	-1.12284000	5.24464800	0.50218700
C	-3.43861400	5.49792800	1.15192200
H	-3.72509600	5.18954900	2.16104200
H	-4.26820000	5.25647600	0.48360000
H	-3.24639100	6.56905300	1.13760100
H	-0.15679200	0.51925800	-0.98915500
O	2.11766300	-0.19371200	-0.10522900
C	2.58798800	1.17263800	-0.05390000
H	3.13027200	1.33593100	0.88331200
H	1.73756200	1.85652500	-0.08806900
C	3.49169800	1.38409900	-1.23266800
C	3.00823600	1.98314200	-2.39853300
C	4.82123900	0.95229400	-1.18779800
C	3.84021800	2.14979100	-3.50548900
H	1.97852400	2.32263800	-2.43651900
C	5.65439100	1.11634000	-2.29205300
H	5.20121400	0.48869700	-0.28363300
C	5.16426500	1.71545000	-3.45383100
H	3.45626700	2.61839800	-4.40463000
H	6.68446400	0.78099600	-2.24703900
H	5.81297000	1.84565700	-4.31272300
**MeCN**			
0 1			
C	0.00000000	0.00000000	0.27669400
N	0.00000000	0.00000000	1.43051400
C	0.00000000	0.00000000	-1.17364200
H	0.00000000	1.02734200	-1.54396900
H	0.88970500	-0.51367100	-1.54396900
H	-0.88970500	-0.51367100	-1.54396900
**$^{-}$OTf**			
-1 1			
O	1.25263200	-0.20727300	1.42393700
S	0.92311400	-0.00003500	-0.00007100
O	1.25275800	1.33660500	-0.53311300
O	1.25148700	-1.13014700	-0.89130100
C	-0.95954000	0.00016300	0.00012500
F	-1.44718400	0.17412600	-1.24226700
F	-1.44645200	0.98922800	0.77213700

F	-1.44720900	-1.16267500	0.47059500
**S9**			
0 1			
C	2.30422700	-0.00000800	0.20550000
C	1.61557600	-1.20692600	0.08091000
C	0.24363400	-1.20460400	-0.16675200
C	-0.45532600	0.00000300	-0.29261300
C	0.24364600	1.20460800	-0.16674300
C	1.61558400	1.20691900	0.08091800
H	3.37183300	-0.00001200	0.39469100
H	2.14677300	-2.14768300	0.17314700
H	-0.29072900	-2.14366200	-0.26681600
H	-0.29071600	2.14366700	-0.26680400
H	2.14679200	2.14766900	0.17316500
C	-1.94101900	0.00001800	-0.52303300
H	-2.23336100	-0.88880200	-1.09232400
H	-2.23334500	0.88886200	-1.09229400
O	-2.60252500	0.00000100	0.75903300
H	-3.55497800	-0.00011000	0.59584300
**TfOH**			
0 1			
O	1.23342300	-1.19952000	-0.86084900
S	0.86471400	-0.14369000	0.05249800
O	1.25371700	-0.13887100	1.44797700
C	-1.01683000	0.00855500	-0.00225600
F	-1.39425800	1.06788600	0.70839900
F	-1.54031700	-1.09421000	0.52326200
F	-1.42119300	0.14432100	-1.25920100
O	1.27582000	1.24042500	-0.65821600
H	1.36380200	1.96945700	-0.00987000
**(*p*-Tol)$_2$SO**			
0 1			
C	-1.36588800	0.69755700	0.15761800
C	-1.47716600	-0.01779000	-1.03540400
C	-2.30930500	0.55779200	1.16811100
C	-2.54507800	-0.88718700	-1.20326700
H	-0.73574500	0.10009900	-1.81702800
C	-3.37363500	-0.32792200	0.98821200
H	-2.21950900	1.12554900	2.08757700
C	-3.50793100	-1.05880600	-0.19405600
H	-2.63671300	-1.44837600	-2.12733500
H	-4.10988700	-0.44422800	1.77580700

C	1.36080100	0.69429900	0.24860300
C	2.20992000	0.79299100	-0.84275900
C	1.56603000	-0.26348800	1.24087300
C	3.27600300	-0.10143300	-0.95419800
H	2.03478500	1.55626200	-1.59123900
C	2.63128900	-1.14649300	1.11518300
H	0.90016400	-0.32917700	2.09468600
C	3.50176900	-1.08138900	0.01577800
H	3.94130100	-0.03211100	-1.80811200
H	2.79328200	-1.89760600	1.88110500
C	4.64945000	-2.04893000	-0.10483200
H	4.28491200	-3.07772600	-0.18741400
H	5.25871700	-1.83266900	-0.98434800
H	5.29403600	-2.00517400	0.77822200
C	-4.65494600	-2.01205200	-0.39531600
H	-5.31771700	-2.02337100	0.47157900
H	-5.24346900	-1.73321500	-1.27473000
H	-4.28987100	-3.03031200	-0.56160600
S	-0.01311900	1.87632000	0.41091700
O	0.06096700	2.78350700	-0.81739100
**TS1**			
0 1			
C	8.18328600	2.85329400	-7.58420000
C	7.73703400	5.18550800	-7.23025200
C	9.19144000	5.50249200	-7.53633300
C	10.07658600	4.36375700	-7.03852500
C	9.66554300	3.06764600	-7.71736500
H	9.45653200	6.43908600	-7.05013200
H	9.98990400	4.29689100	-5.95410900
H	9.88654600	3.14518300	-8.78174400
H	10.19645200	2.21399200	-7.30583200
O	7.33980000	3.84881100	-7.67147800
C	7.53596500	1.50732900	-7.94428800
O	8.46574300	0.57026000	-8.03694000
O	6.34094500	1.35327700	-7.98042500
C	8.01291200	-0.76800200	-8.40537800
H	7.45642200	-0.68688700	-9.34064100
H	7.34147100	-1.12618300	-7.62300600
C	9.23967300	-1.63568500	-8.54828600
H	8.92787000	-2.65211100	-8.80296100
H	9.89092000	-1.26686400	-9.34266000
H	9.80538600	-1.67439700	-7.61411100

O	11.44661400	4.59495900	-7.39763900
C	12.16840600	5.42885900	-6.60895700
O	11.72464200	5.92859500	-5.60042200
C	13.54414900	5.62738200	-7.16465100
H	13.46984400	6.17212800	-8.10954400
H	14.01092000	4.66256100	-7.37355400
H	14.14975000	6.19570300	-6.46131500
O	9.33445700	5.62248000	-8.95728500
C	10.16657000	6.57888000	-9.44478200
O	10.75615300	7.36053500	-8.73482700
C	10.22708000	6.49971500	-10.93515500
H	9.22288500	6.60898900	-11.34848400
H	10.59805300	5.51229200	-11.22106200
H	10.87950400	7.27984000	-11.32289100
C	6.76291500	6.14804800	-7.91094700
H	6.93468300	6.15253400	-8.98500300
O	7.06406900	7.45025700	-7.37189600
C	7.40740600	8.45228200	-8.22759700
O	7.48838300	8.30650700	-9.42265200
C	7.66967500	9.71713200	-7.47007700
H	8.49398100	9.55837000	-6.77007200
H	6.78633700	9.99046900	-6.88796600
H	7.92258600	10.51690800	-8.16311200
C	5.31709100	5.81839900	-7.61047600
H	5.09178700	4.79229900	-7.89716000
H	5.09232100	5.95374600	-6.55028200
O	4.52615200	6.73130400	-8.39620800
C	3.19577400	6.51243000	-8.42710500
O	2.66330200	5.61514000	-7.81134100
C	2.50541200	7.49905000	-9.32031600
H	2.79632900	7.30694500	-10.35703700
H	2.81323700	8.51678400	-9.07214100
H	1.42640700	7.39702800	-9.22107600
H	7.59343800	5.19732800	-6.14643500
C	7.82570400	2.13061100	-4.66150300
C	7.45903100	1.69115000	-3.33848200
H	6.39241100	1.45548100	-3.32267100
H	8.03745100	0.80109000	-3.08087800
H	7.67031700	2.48789600	-2.62179400
N	8.12523100	2.48922400	-5.71798600
C	6.83522400	2.75373400	-10.68163800
H	6.01501400	2.88399700	-9.97458400

H	6.63067000	1.84543400	-11.25986500
O	8.01779200	2.57003800	-9.90801000
H	8.71526600	2.10051500	-10.48156000
C	6.91189800	3.95043900	-11.59954600
C	7.82759100	3.98041900	-12.65767900
C	6.06639200	5.04502900	-11.40747400
C	7.89777900	5.08489500	-13.50199300
H	8.48736500	3.13519000	-12.81876900
C	6.13252900	6.15369900	-12.25229400
H	5.35103400	5.02847300	-10.59244800
C	7.05003700	6.17633900	-13.30075600
H	8.61474100	5.09727100	-14.31549500
H	5.47479300	6.99930000	-12.08531400
H	7.10775200	7.03838400	-13.95587000
S	11.23380600	1.95922600	-11.50197300
O	11.35227600	3.26951800	-10.84225400
O	9.88032500	1.34321300	-11.39140400
O	11.84560500	1.83569300	-12.83211200
C	12.27879900	0.83079500	-10.42026400
F	12.22138500	-0.44003100	-10.85050800
F	11.85919300	0.85884000	-9.14589100
F	13.56541500	1.21877600	-10.44169200
**TS2**			
1 1			
C	7.89906200	3.16283200	-7.67144500
C	7.51538600	5.41566600	-6.98149800
C	8.93911000	5.79144100	-7.34595200
C	9.85447300	4.60345200	-7.06559200
C	9.38796900	3.36624600	-7.81593600
H	9.24025800	6.64622100	-6.74445200
H	9.87165300	4.43081500	-5.98997600
H	9.57764700	3.49206700	-8.88164300
H	9.91059000	2.47726700	-7.47344700
O	7.07723900	4.17938600	-7.61106800
C	7.21789300	1.84340200	-8.04404000
O	8.11873500	0.88907100	-8.20155000
O	6.01887500	1.72207600	-8.08723400
C	7.61379700	-0.43940500	-8.54561100
H	7.04715800	-0.35210000	-9.47386500
H	6.94201900	-0.76222500	-7.74863300
C	8.80875000	-1.34955300	-8.69036900
H	8.46191700	-2.35378000	-8.94768600

H	9.47336500	-1.00166700	-9.48473900
H	9.37281000	-1.41015200	-7.75643900
O	11.18855800	4.87916900	-7.52324600
C	11.99655100	5.59247400	-6.69645300
O	11.65545500	5.94933400	-5.59278700
C	13.31878200	5.86126800	-7.34456400
H	13.17198000	6.56289300	-8.17026200
H	13.73454300	4.94069600	-7.75874700
H	14.00524100	6.29353400	-6.61906800
O	8.99264500	6.13370300	-8.73904400
C	9.74213300	7.20918100	-9.09624500
O	10.35678800	7.87871100	-8.29936900
C	9.67318100	7.43881600	-10.57298600
H	8.67873400	7.81793300	-10.82064400
H	9.82053400	6.50245400	-11.11319800
H	10.42368100	8.17009600	-10.86689800
C	6.47771000	6.47136400	-7.36309300
H	6.50259200	6.66881000	-8.43288700
O	6.85928800	7.65842700	-6.64116100
C	7.11974300	8.79671300	-7.33894400
O	7.07334900	8.86199200	-8.54334600
C	7.47366400	9.91237700	-6.40510800
H	8.36510900	9.64028000	-5.83413900
H	6.65943700	10.07342700	-5.69464500
H	7.66178900	10.82224600	-6.97138300
C	5.08802600	6.06291300	-6.92027200
H	4.77491400	5.16183000	-7.44454800
H	5.05790600	5.88381700	-5.84264100
O	4.20923900	7.15514200	-7.24520300
C	2.88949000	6.92591900	-7.10505900
O	2.44048200	5.85611400	-6.75371000
C	2.07207600	8.14848300	-7.39895700
H	2.64953000	8.91046200	-7.91749100
H	1.71913600	8.55580300	-6.44647100
H	1.19746800	7.86671700	-7.98640200
H	7.47818200	5.23946800	-5.90238000
C	7.77274700	2.22732900	-4.78567900
C	7.60020900	1.68547700	-3.46178000
H	6.53914900	1.49535400	-3.28520300
H	8.16066000	0.75176000	-3.37838600
H	7.97211200	2.40368200	-2.72749100
N	7.92045100	2.67145200	-5.83515000

S	6.54501900	3.11595700	-10.86212100
C	5.72291600	4.71599500	-10.82768000
C	6.37424900	5.87716300	-11.24422300
C	4.41069500	4.75062800	-10.36780600
C	5.69104800	7.08226200	-11.20330600
H	7.39409300	5.83644700	-11.60239100
C	3.73572000	5.97043700	-10.34024600
H	3.91643600	3.84301200	-10.04101900
C	4.36094900	7.14737300	-10.75820500
H	6.19017000	7.98935200	-11.52450600
H	2.71034300	6.00340000	-9.99027900
C	7.29421800	3.18368200	-12.50598400
C	6.47132900	3.07717100	-13.62591400
C	8.66922800	3.32185700	-12.62676500
C	7.04829300	3.12727900	-14.88898200
H	5.39747900	2.96960800	-13.51936000
C	9.23066800	3.36785500	-13.90264900
H	9.28251500	3.39559700	-11.73798100
C	8.43480500	3.27272200	-15.04791900
H	6.41465300	3.05208800	-15.76606300
H	10.30416000	3.48240900	-14.00633800
C	9.04623900	3.30020300	-16.42311600
H	9.05834700	2.29570100	-16.85962400
H	8.47085900	3.93945500	-17.09776600
H	10.07493900	3.66394400	-16.39214200
C	3.64134300	8.46684500	-10.72343300
H	3.76040400	9.00022700	-11.67062200
H	2.57600300	8.33540700	-10.53042500
H	4.05327700	9.10781400	-9.93806400
O	7.75777600	3.18583500	-9.90264900
**49**			
1 1			
C	3.54135000	1.29048500	-1.02700500
C	2.99014800	2.61709200	-0.52308400
C	1.53238700	2.71245300	-0.57597500
C	1.23463900	0.38458300	-1.22876800
C	2.60539700	0.14833900	-0.63736300
H	3.24761900	2.78497000	0.53621800
H	3.40685700	3.48364100	-1.04751200
H	3.64858600	1.30131800	-2.11159100
H	1.27012100	0.38287900	-2.31852200
H	2.98181100	-0.80380000	-1.00527800

O	0.75466200	1.77788300	-0.88016600
O	2.47826300	0.12291900	0.79105100
O	4.81913900	1.13313100	-0.40767000
C	3.08116500	-0.90187400	1.46910500
O	3.68725300	-1.77874000	0.90699200
C	2.89318200	-0.74454000	2.94200200
H	3.58981900	0.02033900	3.29929400
H	1.88022800	-0.41298900	3.17033400
H	3.11094500	-1.68647500	3.44156800
C	0.13225400	-0.51553200	-0.67681300
H	0.03721800	-0.34970600	0.39320700
C	-1.20816600	-0.31479900	-1.36634600
H	-1.50731000	0.73165000	-1.26254200
H	-1.10783400	-0.53672300	-2.43382900
C	5.69864300	0.27481200	-1.00029500
O	0.57664800	-1.85842700	-0.91234300
O	-2.14500100	-1.20343700	-0.79148600
C	0.55296300	-2.73426800	0.13184700
O	0.31353600	-2.39019900	1.26395400
O	5.42012200	-0.32756700	-2.00856800
C	7.02051000	0.22802800	-0.24643000
C	0.84541400	-4.15436700	-0.33384500
C	0.85634900	4.04076000	-0.21754000
O	1.47433900	5.07107000	-0.30664600
O	-0.37882400	3.85114100	0.16878700
C	-1.14147100	5.04589400	0.58800300
H	-1.21703300	5.69941100	-0.28170900
H	-0.55947400	5.54268600	1.36496400
C	-2.48134700	4.56886200	1.08323800
H	-3.05035500	5.43477400	1.43171000
H	-3.05002800	4.08339300	0.28850200
H	-2.36818800	3.87415600	1.91705900
C	-4.40219400	-2.46398900	0.31848700
Si	-3.61442800	-0.78527900	-0.08882600
C	-3.29528200	0.20573500	1.48121300
C	-2.13781300	-0.00555700	2.24855100
C	-4.24279800	1.12362900	1.96272600
C	-1.92651100	0.68545000	3.44113100
H	-1.39724100	-0.72702000	1.92403300
C	-4.04247600	1.80814700	3.16060100
H	-5.14778200	1.31379800	1.39585000
C	-2.87995900	1.59390400	3.90056500

H	-1.02308600	0.50881800	4.01494100
H	-4.78760800	2.51349500	3.51192600
H	-2.71849100	2.13087400	4.82881500
C	-4.56857700	0.33015400	-1.27363300
C	-5.61670600	-0.11275900	-2.09625300
C	-4.18274000	1.67876100	-1.38295800
C	-6.25202200	0.75111000	-2.98847700
H	-5.95531800	-1.13940100	-2.04346100
C	-4.80761300	2.54501900	-2.27755000
H	-3.38866100	2.06490000	-0.75403800
C	-5.84736400	2.08175100	-3.08345800
H	-7.06354500	0.38472100	-3.60761400
H	-4.48882000	3.57972700	-2.34123100
H	-6.34047600	2.75355300	-3.77726100
C	6.76501400	-0.06956900	1.24344300
H	6.16055000	0.70964600	1.70987100
H	6.25711900	-1.02772500	1.37278100
H	7.72514900	-0.11746200	1.76347300
C	7.69818300	1.60655000	-0.39978700
H	8.67107900	1.58250500	0.09767100
H	7.85912300	1.85096600	-1.45351200
H	7.09848700	2.39781300	0.05407100
C	7.90560500	-0.86387400	-0.85687800
H	8.10854900	-0.66967900	-1.91200700
H	8.85852200	-0.89272600	-0.32291200
H	7.43567400	-1.84666500	-0.77309000
C	-0.37380200	-4.63300300	-1.15106700
H	-1.28427600	-4.61349700	-0.54751300
H	-0.52921200	-4.01139000	-2.03456400
H	-0.20053000	-5.66179100	-1.47751200
C	2.11045800	-4.17553800	-1.21164200
H	1.97959600	-3.58167800	-2.11734200
H	2.97485600	-3.79134900	-0.66537600
H	2.32001900	-5.20740200	-1.50509500
C	1.03892600	-5.05335200	0.89224900
H	0.14895600	-5.06141700	1.52450900
H	1.23412000	-6.07604100	0.56031400
H	1.88623900	-4.72148200	1.49700100
C	-3.53612200	-3.14554500	1.39836400
H	-3.52486700	-2.57594500	2.33100500
H	-3.94428200	-4.13927700	1.61982600
H	-2.50253200	-3.27515100	1.06825800

C	-4.43840900	-3.37827600	-0.92073100
H	-3.43802500	-3.52970700	-1.33285200
H	-4.83842900	-4.36076600	-0.64055300
H	-5.07284200	-2.98212600	-1.71661600
C	-5.82376700	-2.26280700	0.87344100
H	-6.49823600	-1.82390100	0.13393200
H	-6.24498300	-3.23015200	1.17388500
H	-5.82558100	-1.61610400	1.75643900
**50α**			
1 1			
C	-1.93535600	2.40477500	0.17196600
C	-1.05750800	0.18100700	0.38872800
C	-2.40322600	-0.43935300	0.03876800
C	-3.52467000	0.49116700	0.49774000
C	-3.35593600	1.88430100	-0.09107700
H	-2.49565400	-1.40809400	0.52266900
H	-3.51855900	0.52079000	1.58854100
H	-3.48138500	1.84835200	-1.17518300
H	-4.08713100	2.57694500	0.32411700
O	-0.93703100	1.51724500	-0.15549100
C	-1.68024100	3.71306000	-0.63216100
O	-2.38030900	4.70222000	-0.11109100
O	-0.97492300	3.74672700	-1.60261900
C	-2.30588300	6.00766300	-0.78416300
H	-2.67005100	5.87312900	-1.80329100
H	-1.25714700	6.30486500	-0.81358500
C	-3.15489000	6.96885600	0.00899100
H	-3.11932400	7.94943200	-0.47270400
H	-4.19638100	6.64062700	0.04178600
H	-2.78090200	7.07494500	1.03007100
O	-4.79917800	0.01460900	0.04210700
C	-5.40669800	-0.95400500	0.77448800
O	-4.91683000	-1.40363000	1.78406900
C	-6.76140100	-1.33161300	0.18535900
O	-2.47533800	-0.59969500	-1.38882900
C	-2.95551200	-1.77389400	-1.87460400
O	-3.30669900	-2.68703400	-1.16536000
C	-3.00617100	-1.75035100	-3.36957100
H	-2.11170900	-1.28136700	-3.78057200
H	-3.87201700	-1.15555200	-3.67704700
H	-3.11680800	-2.76402300	-3.75008600
C	0.13523700	-0.57911900	-0.18713700

H	0.08263200	-0.57924000	-1.27414900
O	0.03657900	-1.93110000	0.29989600
C	0.05354300	-2.95992100	-0.58311000
O	0.06765500	-2.80364700	-1.78107400
C	0.04865500	-4.30035300	0.14797600
C	1.46048900	-0.00415200	0.27194800
H	1.45005400	1.07912600	0.12835200
H	1.59266700	-0.20814900	1.34063200
O	2.49016300	-0.61176800	-0.49115200
H	-0.97706100	0.23084700	1.48015800
C	-1.68277500	2.96184600	2.71940000
C	-1.52693000	3.23869800	4.11385600
H	-0.46198200	3.34701000	4.33655000
H	-2.05548300	4.16725400	4.34781300
H	-1.95042400	2.41073400	4.68869100
N	-1.81593800	2.74443800	1.60393800
C	-7.28250200	-2.58805300	0.89146100
H	-7.39578600	-2.42486800	1.96498200
H	-8.25892200	-2.85397100	0.47898900
H	-6.60596500	-3.43327600	0.74293800
C	-7.71867500	-0.14684800	0.44038400
H	-7.80023100	0.07151900	1.50894500
H	-7.38081400	0.75446600	-0.07517500
H	-8.71365800	-0.40421600	0.06790700
C	-6.63922800	-1.58831000	-1.32781000
H	-6.29890700	-0.69836600	-1.85883200
H	-5.94237900	-2.40332700	-1.53413600
H	-7.62055400	-1.86824500	-1.71975400
C	1.34114700	-4.40748100	0.98212300
H	1.35446400	-5.37088600	1.49890300
H	2.22734300	-4.34945700	0.34615100
H	1.39816500	-3.61451700	1.72930300
C	-0.00748900	-5.43478800	-0.88015100
H	0.85263900	-5.40524900	-1.55253500
H	-0.00230200	-6.39486900	-0.35782300
H	-0.91582000	-5.37817300	-1.48437800
C	-1.17975600	-4.36911700	1.07668600
H	-1.13536900	-3.60244800	1.85199800
H	-2.10682300	-4.24018900	0.51402900
H	-1.20346600	-5.34804600	1.56296500
Si	4.12317700	-0.27447300	-0.30965500
C	5.00917700	-1.54727500	-1.40598700

C	4.64166300	-1.27558500	-2.87703900
H	3.56364300	-1.35521500	-3.04474000
H	4.96161100	-0.28071400	-3.19909600
H	5.13506900	-2.01077300	-3.52488000
C	6.53159600	-1.41451000	-1.22396800
H	6.83413200	-1.58975400	-0.18697900
H	7.04575800	-2.15473700	-1.84933000
H	6.89429000	-0.42654000	-1.52112200
C	4.57220800	-2.97462900	-1.03349300
H	4.84748700	-3.23154400	-0.00731600
H	3.49283300	-3.10456600	-1.14041500
H	5.06491500	-3.69620700	-1.69720700
C	4.44544100	1.47248500	-0.93271800
C	5.69048500	2.10910300	-0.79212100
C	3.43720600	2.15671500	-1.63224700
C	5.91462300	3.38201600	-1.31501500
H	6.50403000	1.60885500	-0.27929300
C	3.65506200	3.43100400	-2.15502200
H	2.47109300	1.68853400	-1.78270600
C	4.89480300	4.04847500	-1.99436200
H	6.88455900	3.85202900	-1.19437000
H	2.85918200	3.93842900	-2.68933600
H	5.06736900	5.03906400	-2.40041500
C	4.52364200	-0.45444000	1.52251700
C	4.03066600	-1.58040400	2.20716200
C	5.26080200	0.48585700	2.25809500
C	4.27598800	-1.76639200	3.56596400
H	3.43816100	-2.31813100	1.67849200
C	5.51248000	0.30297100	3.61843800
H	5.64044400	1.37792300	1.77494300
C	5.02280600	-0.82493200	4.27504900
H	3.88389200	-2.64251200	4.07103100
H	6.08662700	1.04320800	4.16503900
H	5.21708700	-0.96757800	5.33233900
**50β**			
1 1			
C	1.52045100	-2.61887000	-0.11653100
C	1.01649800	-0.34053300	0.64119200
C	2.37898200	0.14531500	0.17199900
C	3.39689900	-0.98465200	0.29435100
C	2.95136100	-2.20017500	-0.50843500
H	2.68230800	0.99729600	0.77474200

H	3.53135100	-1.22117800	1.34855200
H	2.94554400	-1.94309900	-1.56821400
H	3.62898300	-3.04033000	-0.35432300
O	0.61015400	-1.55832600	-0.03207500
C	1.49895100	-3.52131200	1.15582000
O	1.72210500	-2.77384400	2.21844200
O	1.31982500	-4.71166800	1.12766800
C	1.76507800	-3.44210200	3.52921600
H	2.56376200	-4.18371700	3.49355600
H	0.80954000	-3.94710400	3.67266300
C	2.01476200	-2.37297600	4.56299100
H	2.05850100	-2.84169900	5.54952900
H	2.96437000	-1.86393700	4.38267200
H	1.20937200	-1.63504200	4.56942800
O	4.65712400	-0.59113400	-0.27271500
C	5.48857500	0.15311600	0.50049600
O	5.20862600	0.46994900	1.63284600
C	6.79096400	0.47125300	-0.22643800
O	2.28450400	0.53974600	-1.21015200
C	2.84971200	1.71911800	-1.57495900
O	3.40653600	2.45397200	-0.79352900
C	2.70940400	1.94715100	-3.04714800
H	1.73657300	1.60934900	-3.40397500
H	3.48203600	1.36630400	-3.56092800
H	2.85416300	3.00258900	-3.27076200
C	-0.11363100	0.63496400	0.32051400
H	-0.19285700	0.74871700	-0.75701200
O	0.23557600	1.90553100	0.90048200
C	0.20254600	3.00923800	0.11363600
O	0.01807100	2.96492200	-1.08026200
C	0.40435800	4.27694000	0.93719200
C	-1.44356200	0.19215500	0.90269700
H	-1.62624000	-0.84338500	0.60664800
H	-1.39872300	0.23443700	1.99625300
O	-2.45866500	1.06394600	0.43348800
H	1.07184600	-0.52605700	1.71557500
C	0.52325300	-4.05226700	-2.02578000
C	-0.04949000	-4.83256400	-3.07761900
H	0.46260200	-5.79836400	-3.11235700
H	-1.11230900	-4.97678500	-2.86750300
H	0.08191400	-4.30363300	-4.02480600
N	0.98657600	-3.43554700	-1.18292200

C	-0.85358600	4.46614400	1.81108100
H	-0.75192900	5.38706400	2.39152300
H	-1.75255400	4.54784500	1.19432400
H	-0.98466100	3.63311100	2.50417700
C	0.56754400	5.47141300	-0.00874800
H	-0.30938700	5.59555400	-0.64729000
H	0.69628100	6.38314800	0.58010600
H	1.44346200	5.35161100	-0.65079900
C	1.65089700	4.13519300	1.83005200
H	1.53782300	3.32134600	2.54772900
H	2.54392400	3.94633900	1.22984900
H	1.79910400	5.06519300	2.38545500
C	6.50406900	0.99486400	-1.64545300
H	5.98025900	0.25159600	-2.24795200
H	5.90160600	1.90518200	-1.61538300
H	7.45294600	1.22777800	-2.13580700
C	7.60327100	-0.83967900	-0.30522400
H	8.56480600	-0.63714900	-0.78426200
H	7.79690000	-1.24375600	0.69236800
H	7.07929000	-1.59699900	-0.89204700
C	7.56817600	1.52188900	0.57434800
H	7.79717900	1.16763400	1.58122900
H	8.50943700	1.73991000	0.06361600
H	7.00100100	2.45209000	0.65946200
Si	-3.92042000	0.57695500	-0.23216700
C	-4.59613800	2.14994000	-1.05125700
C	-3.57662900	-0.78257300	-1.49467900
C	-4.47512100	-1.84431900	-1.68485000
C	-2.42285200	-0.75173300	-2.29622800
C	-4.23270000	-2.83694500	-2.63469800
H	-5.37475600	-1.90332600	-1.08194100
C	-2.16746500	-1.74674000	-3.23792300
H	-1.71148700	0.05815600	-2.18973500
C	-3.07486300	-2.79234200	-3.41030300
H	-4.94352700	-3.64563400	-2.76529300
H	-1.26518400	-1.70395700	-3.83830200
H	-2.88167200	-3.56647100	-4.14437500
C	-4.98614400	-0.17974200	1.12953000
C	-6.16800400	0.39203700	1.62492000
C	-4.54783400	-1.37992400	1.71980500
C	-6.88316500	-0.20563300	2.66354600
H	-6.54742500	1.31291400	1.20129800

C	-5.25337900	-1.97847800	2.76130600
H	-3.64656500	-1.86370100	1.35816700
C	-6.42658000	-1.39126800	3.23597500
H	-7.79581600	0.25587600	3.02447200
H	-4.89218800	-2.90290900	3.19870300
H	-6.98066900	-1.85622400	4.04383900
C	-3.59530000	2.58644400	-2.14114200
H	-3.94416900	3.51835900	-2.60307700
H	-2.59920000	2.76739400	-1.72981300
H	-3.50669200	1.83927800	-2.93430300
C	-5.95798000	1.87212800	-1.71322400
H	-6.30488700	2.77099500	-2.23783200
H	-5.88970400	1.06687500	-2.45133900
H	-6.72744500	1.59696200	-0.98798800
C	-4.72121900	3.29077600	-0.02341800
H	-5.05965700	4.20495700	-0.52712600
H	-5.43972100	3.06654700	0.76813700
H	-3.75910900	3.50726700	0.44805000
**51**			
1 1			
C	3.49009900	-1.58085200	-0.56339900
O	2.24381500	-1.57202700	-0.67251300
C	4.23927200	-1.14680600	0.61843200
C	4.19307300	-2.10215800	-1.83120200
C	1.32568200	-1.08128800	0.43382600
H	4.84410500	-0.28928200	0.28948100
H	4.96587400	-1.92905200	0.86091400
C	3.35039300	-0.78272800	1.80613300
O	5.49312300	-1.98304200	-1.69765000
O	3.57036000	-2.54093100	-2.76083300
C	2.08783500	-0.09534700	1.30997400
H	1.07942300	-1.99313500	0.97707500
C	0.09206300	-0.54481300	-0.28648700
O	4.02433300	0.13159700	2.67946200
H	3.07685200	-1.68003100	2.36182400
C	6.32906600	-2.42505400	-2.82938300
H	1.44287100	0.13750800	2.16247500
O	0.29944100	0.72955000	-0.86074400
H	-0.13615000	-1.23434000	-1.10398400
C	-1.09600500	-0.56345700	0.67728300
C	4.98903500	-0.38178900	3.48487200
H	6.02475500	-1.84521300	-3.70156800

H	6.10821900	-3.47916800	-3.00102000
C	7.76607000	-2.18632100	-2.44455600
O	-2.23094100	-0.07124400	-0.00245200
H	-1.24132800	-1.59710600	1.01225500
H	-0.88694400	0.05121100	1.55754600
O	5.29475800	-1.55210000	3.46875500
C	5.59111900	0.68302300	4.34681100
H	8.03766600	-2.76156300	-1.55662400
H	7.95378100	-1.12665200	-2.25777800
H	8.40535100	-2.50790100	-3.27079400
H	6.07757800	1.43001600	3.71392000
H	6.32052500	0.24217300	5.02320400
H	4.80837200	1.18958100	4.91584400
O	2.45802900	1.06559600	0.59362700
Si	1.35750300	1.92812200	-0.35934300
C	2.29833300	2.51062400	-1.89623300
C	0.42320500	3.22627800	0.67386900
Si	-3.82586700	-0.41578400	0.39061200
C	-4.25282100	0.24480100	2.12817800
C	-4.79247900	0.49592200	-0.93357500
C	-6.07587800	0.06682900	-1.30645400
C	-4.27123900	1.64210700	-1.55395600
C	-6.81842700	0.76220500	-2.25984500
H	-6.50275400	-0.82254000	-0.85381300
C	-5.00910700	2.33945000	-2.51015500
H	-3.27817800	1.98805400	-1.29287900
C	-6.28562400	1.90176900	-2.86317500
H	-7.80805400	0.41415500	-2.53518700
H	-4.58798500	3.22149900	-2.98048600
H	-6.86028400	2.44252500	-3.60707000
C	-4.06012100	-2.27422500	0.18976100
C	-5.00078200	-3.03270000	0.90363900
C	-3.27616100	-2.93812000	-0.76996600
C	-5.15388500	-4.39943400	0.66991400
H	-5.62492000	-2.56251100	1.65352100
C	-3.42147500	-4.30411800	-1.00492700
H	-2.54263400	-2.37996300	-1.34242300
C	-4.36326500	-5.03869000	-0.28409300
H	-5.88882100	-4.96411100	1.23316600
H	-2.80372500	-4.79424300	-1.74961300
H	-4.48054400	-6.10134900	-0.46550800
C	2.88246400	1.27633900	-2.61144200

H	3.69336800	0.83413100	-2.02668600
H	2.12825300	0.51130800	-2.80929700
H	3.31039400	1.58143100	-3.57347200
C	1.32302700	3.22687000	-2.85161200
H	0.89398900	4.12645900	-2.40411700
H	1.85710500	3.53064300	-3.75996800
H	0.49980300	2.57336600	-3.15514200
C	3.46658900	3.44995200	-1.53842200
H	4.05209600	3.65852200	-2.44219300
H	3.12611900	4.40586100	-1.14048700
H	4.14185100	2.99698800	-0.80608200
C	1.12968000	4.59468000	0.67315000
H	1.16839600	5.03789300	-0.32371200
H	0.57438300	5.28513500	1.31968500
H	2.15087700	4.53188800	1.05988300
C	-0.99550200	3.38621400	0.09074300
H	-1.53618000	2.43940100	0.08973800
H	-1.56243700	4.10549200	0.69400100
H	-0.97193300	3.76211400	-0.93608800
C	0.32067800	2.75141200	2.13733000
H	-0.24904600	3.48731700	2.71701400
H	-0.19719500	1.79546900	2.23644900
H	1.30664600	2.65369600	2.59963400
C	-3.65100100	1.65813900	2.25584600
H	-3.92977000	2.09015800	3.22506700
H	-2.56101300	1.64265000	2.20013700
H	-4.01928400	2.33196900	1.47678600
C	-3.66067300	-0.65212100	3.23140500
H	-2.57264700	-0.73145900	3.15407500
H	-3.88816000	-0.22481400	4.21617600
H	-4.07296300	-1.66340200	3.20646000
C	-5.77974500	0.34785400	2.30985500
H	-6.00288800	0.75033400	3.30584100
H	-6.22785600	1.01964200	1.57312500
H	-6.28217600	-0.61937400	2.22887500
**52α**			
1 1			
O	-2.40513200	0.89442900	-1.03029200
C	-4.02535000	0.33833000	0.72776700
C	-1.28578100	0.72426600	-0.11494000
H	-4.28616200	-0.63401900	0.30735400
H	-4.90084100	0.76372500	1.21533200

C	-2.86335700	0.17538500	1.70025600
C	-1.62632500	-0.31118200	0.96302000
H	-1.09712300	1.68609500	0.37124500
C	-0.05839700	0.37477100	-0.96311400
O	-3.20045500	-0.79349600	2.70916600
H	-2.63122800	1.12163700	2.19054200
H	-0.78907900	-0.35473900	1.66627700
O	0.03861800	-0.99998200	-1.30477700
H	-0.13768000	0.93850200	-1.89765100
C	1.20498700	0.82878100	-0.23804200
C	-3.85025200	-0.34664300	3.80897600
O	2.32765100	0.57721300	-1.06176700
H	1.12207900	1.90155000	-0.02526800
H	1.29392300	0.29940800	0.71616000
O	-4.15119200	0.81504000	3.97280300
C	-4.13556600	-1.47185700	4.75655000
H	-4.82356300	-2.17737800	4.28266700
H	-4.58131800	-1.08312700	5.67007400
H	-3.21422200	-2.01162000	4.98640800
O	-1.89039300	-1.58522600	0.40158100
Si	-0.79130200	-2.29599200	-0.65888600
C	-1.80628300	-3.12833500	-2.02969200
C	0.43014400	-3.38802100	0.30644100
Si	3.91961600	0.58675500	-0.53719200
C	4.91465000	-0.01504300	-2.03904400
C	4.37581100	2.36051900	-0.09797600
C	5.45432300	2.65636900	0.75050600
C	3.66665900	3.43399600	-0.65978200
C	5.81533300	3.97493800	1.02537700
H	6.01801300	1.85035900	1.20892600
C	4.01864400	4.75506300	-0.38427900
H	2.83046800	3.23584600	-1.32203500
C	5.09624900	5.02764000	0.45851500
H	6.65314200	4.18182800	1.68239900
H	3.45619600	5.57000800	-0.82710100
H	5.37341000	6.05383100	0.67334400
C	4.07816900	-0.44004900	1.03625700
C	4.57161900	-1.75352600	1.07173500
C	3.62659500	0.11750800	2.24598500
C	4.61581000	-2.47902800	2.26152000
H	4.93141100	-2.22590800	0.16694700
C	3.65601300	-0.60650400	3.43632500

H	3.24958500	1.13466600	2.26342400
C	4.15402400	-1.90897800	3.44638900
H	5.00412100	-3.49156500	2.26099100
H	3.29596300	-0.15434700	4.35405300
H	4.18131200	-2.47521500	4.37079200
C	-3.60390900	1.23240700	-0.44962300
C	-4.70007000	1.22651200	-1.55464700
O	-4.54829500	0.69809400	-2.62157500
O	-5.78539900	1.83316800	-1.10879200
C	-6.95156500	1.86602400	-2.00227200
H	-6.65340600	2.37911200	-2.91744000
H	-7.21481800	0.83453100	-2.23909100
C	-8.05524600	2.58841600	-1.27125700
H	-7.76417100	3.61360200	-1.03044200
H	-8.93852400	2.62467500	-1.91425200
H	-8.32338000	2.06728000	-0.34923500
C	-3.39819800	3.70192600	0.42279400
C	-3.26056000	5.04279400	0.90330700
H	-2.45106200	5.53312300	0.35624300
H	-4.20125400	5.57445100	0.73487300
H	-3.03075600	5.01433500	1.97166300
N	-3.51670800	2.62870800	0.04451900
C	-2.38681500	-2.02215900	-2.93074100
H	-2.99348500	-1.30797600	-2.37375100
H	-1.59668400	-1.46003800	-3.43526600
H	-3.02264500	-2.47552400	-3.70170800
C	-2.97255500	-3.92964500	-1.41780800
H	-3.58169900	-4.36138900	-2.22181500
H	-2.62716500	-4.75331400	-0.78894500
H	-3.62285200	-3.29263000	-0.81247400
C	-0.93988100	-4.06084400	-2.89654000
H	-0.55007300	-4.91061000	-2.33222300
H	-1.54599500	-4.46103200	-3.71900200
H	-0.09178000	-3.53202400	-3.34190500
C	-0.21404400	-4.73046900	0.69951400
H	-0.47314100	-5.33647800	-0.17096700
H	0.49133800	-5.31080400	1.30753700
H	-1.12081200	-4.58833400	1.29561900
C	1.69351400	-3.64330100	-0.53797300
H	2.17698600	-2.70677400	-0.82195500
H	2.41486100	-4.22910000	0.04479300
H	1.47557800	-4.20286800	-1.45047100

C	0.84639500	-2.65743600	1.59836500
H	1.55612800	-3.27908300	2.15530100
H	1.34338000	-1.70817900	1.39142100
H	-0.00950300	-2.46641600	2.25120100
C	4.36029600	-1.34774600	-2.57698000
H	4.45187100	-2.16296800	-1.85635700
H	3.30570000	-1.25867800	-2.84831200
H	4.91644600	-1.64046700	-3.47653000
C	6.40006000	-0.16825300	-1.66476800
H	6.97875000	-0.45306300	-2.55223700
H	6.82034800	0.76889300	-1.28670100
H	6.55849100	-0.93805300	-0.90561700
C	4.79058400	1.04696100	-3.15101200
H	5.20945400	2.00902100	-2.84458400
H	5.33918500	0.71242200	-4.04010200
H	3.74927100	1.20774900	-3.44413100
**52β**			
1 1			
O	-2.37831500	0.66069400	-1.22689100
C	-4.07562700	0.15517500	0.47491600
C	-3.79243100	2.53066000	-0.53189700
C	-1.31260900	0.65227200	-0.24003500
H	-4.19701500	-0.87880600	0.15240100
H	-5.02116700	0.53394400	0.85643800
C	-2.97641300	0.21730700	1.53020100
O	-5.01848300	2.81808500	-0.14090400
O	-2.88580600	3.30076000	-0.71051000
C	-1.66275200	-0.27081600	0.93654500
H	-1.18364400	1.66743800	0.14103100
C	-0.04946600	0.25843000	-1.00917400
O	-3.31746600	-0.63738900	2.63620700
H	-2.84305700	1.23414900	1.90383300
C	-5.33554800	4.23104900	0.11249700
H	-0.87385700	-0.17282000	1.68911800
O	0.01286800	-1.12839000	-1.31102400
H	-0.07842200	0.79026000	-1.96458400
C	1.19220700	0.72203300	-0.25644400
C	-4.11166900	-0.11882200	3.60170300
H	-5.17988300	4.77573600	-0.81960800
H	-4.63462000	4.59651900	0.86361900
C	-6.76653700	4.28992300	0.58436700
O	2.33994800	0.43583000	-1.03645200

H	1.10755800	1.80075200	-0.07856800
H	1.25394300	0.22463000	0.71580400
O	-4.53797600	1.01408800	3.56929400
C	-4.37635200	-1.12886800	4.67673200
H	-6.89875200	3.72353700	1.50915900
H	-7.44850500	3.89906200	-0.17453200
H	-7.03098400	5.33253500	0.77917600
H	-4.91599800	-1.97945300	4.25172500
H	-4.96739200	-0.67771900	5.47133300
H	-3.43172300	-1.50312800	5.07777800
O	-1.81452500	-1.62328400	0.53982900
Si	-0.72394800	-2.38297800	-0.48962600
C	-1.74943400	-3.38334600	-1.73520900
C	0.60239900	-3.31705700	0.51100800
Si	3.91459000	0.61263800	-0.48617900
C	4.99442400	-0.04827900	-1.90239300
C	4.23117600	2.44767300	-0.20765200
C	5.25972800	2.89739200	0.63501400
C	3.46730800	3.41060900	-0.88589200
C	5.52036100	4.25822900	0.79222200
H	5.86306800	2.17957500	1.18104800
C	3.71818400	4.77329300	-0.72794700
H	2.66690000	3.09301800	-1.54576300
C	4.74784000	5.19945800	0.11116600
H	6.32126300	4.58457100	1.44680600
H	3.11338200	5.50111200	-1.25809900
H	4.94618400	6.25842100	0.23508800
C	4.09449100	-0.25189100	1.18025700
C	4.67568800	-1.51793300	1.35212100
C	3.55793000	0.37687400	2.31846600
C	4.72118800	-2.13038400	2.60395100
H	5.10337300	-2.04217100	0.50720900
C	3.58925600	-0.23481500	3.57015600
H	3.11012100	1.36113000	2.23089500
C	4.17385600	-1.49242000	3.71564500
H	5.17851700	-3.10807500	2.70925700
H	3.16203400	0.26971900	4.42993000
H	4.20288100	-1.97101900	4.68825700
C	-3.64250300	0.98187100	-0.74969000
C	-5.22085200	0.38732400	-2.71674700
C	-6.07885000	0.04659200	-3.80815800
H	-7.11479000	0.23566300	-3.51309700

H	-5.81500500	0.66209700	-4.67229700
H	-5.94380400	-1.01274600	-4.04365200
N	-4.53410500	0.65601200	-1.84321400
C	-2.67593700	-4.38990500	-1.02519700
H	-3.33101600	-4.86665000	-1.76525900
H	-2.12284800	-5.18201500	-0.51943700
H	-3.31585600	-3.89843300	-0.28636700
C	-0.82247100	-4.12933500	-2.71327000
H	-0.20106300	-4.87095800	-2.20583000
H	-1.42442500	-4.65900900	-3.46229000
H	-0.16015300	-3.44239800	-3.24875800
C	-2.63368100	-2.40267000	-2.53000300
H	-3.36259400	-1.91718800	-1.87888000
H	-2.04716500	-1.62379700	-3.02137300
H	-3.18649400	-2.95341700	-3.30129400
C	0.16360100	-4.74380700	0.88627300
H	0.02858300	-5.37769100	0.00744900
H	0.93630700	-5.20815700	1.51184500
H	-0.76909900	-4.75087700	1.45826000
C	1.90272400	-3.38170900	-0.31410600
H	2.25982300	-2.38303500	-0.56664400
H	2.68429300	-3.88723100	0.26566700
H	1.77132600	-3.93779600	-1.24656800
C	0.88255700	-2.54509900	1.81614400
H	1.65753800	-3.06662500	2.38899000
H	1.24855600	-1.53438900	1.62914800
H	-0.00916100	-2.47544000	2.44476200
C	4.83008300	0.89817500	-3.10947000
H	5.17014700	1.91180900	-2.88226400
H	5.42840100	0.52629100	-3.95034900
H	3.78959900	0.95544200	-3.44151700
C	4.55689900	-1.46112200	-2.33155500
H	4.67729900	-2.19919700	-1.53608200
H	3.51026600	-1.47550100	-2.64427200
H	5.16709500	-1.79120000	-3.18181000
C	6.47451600	-0.05647000	-1.47832500
H	7.10015300	-0.37078300	-2.32294200
H	6.81363800	0.93798100	-1.17201000
H	6.66434600	-0.74568200	-0.65181800
**53**			
1 1			
O	-0.15677100	0.32234200	-0.70160800

C	-0.00243400	2.67609100	-0.50878300
C	1.77800400	1.21008200	-1.75507700
C	0.29234500	-1.03247900	-1.18678900
O	0.59412500	3.69022800	-0.77275800
O	-1.11578500	2.53134300	0.16405200
H	1.48542900	0.98261600	-2.79318600
H	2.32886000	2.14728900	-1.75554000
C	2.61429400	0.04133700	-1.20740600
C	1.76319900	-1.21372400	-0.86311900
H	0.15423300	-0.98958400	-2.26857300
C	-0.66206800	-2.03728300	-0.57730300
C	-1.75636400	3.75360200	0.68419800
O	3.15543700	0.44336200	0.04473700
H	3.39762200	-0.19818500	-1.92603100
O	1.92507500	-1.38181600	0.53566000
H	2.11030000	-2.08801300	-1.41945300
O	-1.96701700	-1.81394300	-1.10721500
H	-0.30245700	-3.02518500	-0.88538900
C	-0.86049300	-1.98040800	0.94562900
H	-1.04421500	4.22923500	1.35963300
H	-1.94145000	4.40999600	-0.16669400
C	-3.02528600	3.33116300	1.37882800
C	3.11827200	-0.68453400	0.93177800
C	-2.93433000	-1.82853900	-0.02544200
O	-2.22492700	-2.36769300	1.08856500
H	-0.68890900	-0.97873800	1.34821800
H	-0.24048900	-2.69474500	1.48009300
H	-3.71164000	2.84265600	0.68413900
H	-2.81446300	2.65555300	2.21044700
H	-3.51572000	4.22332000	1.77691900
C	2.93966500	-0.18419800	2.34553100
C	4.34187600	-1.56814400	0.74035200
C	-4.06273700	-2.77207900	-0.38341700
C	-3.40072100	-0.40449700	0.24766800
H	3.81099700	0.40145500	2.64411200
H	2.04616200	0.43915100	2.40904800
H	2.83960500	-1.03015400	3.02793000
H	4.41935800	-1.91521000	-0.29244500
H	5.24499300	-1.00754300	0.98968300
H	4.27501000	-2.43832200	1.39638600
H	-4.59049500	-2.40556600	-1.26624800
H	-3.66341600	-3.76669800	-0.59017600

H	-4.77289700	-2.83452100	0.44406200
H	-2.56428400	0.23097200	0.53725800
H	-3.85715300	0.01514200	-0.65151900
H	-4.14240500	-0.40427300	1.04973900
C	0.53283300	1.32840400	-0.99229700
**54α**			
1 1			
C	0.48215800	1.41566200	-0.66345000
O	-0.31587300	0.34396600	-0.35840200
C	1.29367000	1.87440600	0.58977700
C	1.39762100	1.21240000	-1.88093200
C	-0.50942900	-0.63708300	-1.40604700
O	2.09563200	2.77272600	0.51298100
O	0.96405100	1.19676000	1.66479100
H	0.82258600	1.35393700	-2.79713500
H	2.19434500	1.95390400	-1.85895900
C	1.94583300	-0.20085500	-1.85492400
C	0.82924700	-1.27278100	-1.76758100
H	-0.91326200	-0.14031300	-2.29355100
C	-1.55465200	-1.62296600	-0.90895300
C	1.68929300	1.51817500	2.89808000
O	2.68488800	-0.39004400	-0.64847200
H	2.57765300	-0.36552700	-2.73030300
O	1.26182500	-2.15572100	-0.73534600
H	0.72200700	-1.81388200	-2.71074600
O	-2.83362200	-0.97788200	-0.84713700
H	-1.61180900	-2.43499000	-1.64088700
C	-1.34818000	-2.17955800	0.50946500
H	2.74292100	1.29230400	2.72758300
H	1.57922100	2.58705100	3.08500600
C	1.09134400	0.67879700	3.99985700
C	2.57555900	-1.77096200	-0.29152600
C	-3.40606100	-1.17338300	0.46386000
O	-2.68280600	-2.27019600	1.01655100
H	-0.73964700	-1.51280400	1.12166600
H	-0.91402700	-3.17560000	0.51280700
H	0.03126300	0.90607600	4.13526800
H	1.20195800	-0.38698800	3.78889700
H	1.61246800	0.90018400	4.93502700
C	2.63678100	-1.88671000	1.21562600
C	3.63073800	-2.60169200	-1.01042500
C	-4.85853600	-1.57676800	0.31293900

C	-3.22158100	0.08936400	1.30019300
H	3.60676300	-1.54021400	1.57804400
H	1.84723500	-1.28330600	1.66131300
H	2.50715400	-2.92908200	1.51318500
H	3.54905400	-2.48263000	-2.09321900
H	4.62970900	-2.29138400	-0.69565200
H	3.49798500	-3.65796000	-0.76713400
H	-5.42391400	-0.77150400	-0.16087900
H	-4.93122000	-2.47574100	-0.30202600
H	-5.29600900	-1.77598900	1.29379800
H	-2.16379400	0.33141100	1.40373600
H	-3.72289600	0.93073400	0.81575500
H	-3.65784500	-0.05412900	2.29170400
C	-1.15241000	3.41263400	-1.16441500
C	-2.08553900	4.46765300	-1.42068000
H	-1.53404200	5.40473600	-1.53532400
H	-2.63337600	4.24085800	-2.33917800
H	-2.77768600	4.53996800	-0.57759000
N	-0.40152800	2.57371600	-0.96087600
**54β**			
1 1			
C	0.25886900	1.46809200	-0.00508400
O	-0.26058900	0.18371000	0.09349500
C	1.05922700	1.73950800	-1.28793300
C	-0.18775600	-0.65722000	-1.08234200
H	0.34426900	1.88325700	-2.09945200
H	1.62398700	2.66494100	-1.17173300
C	1.97071100	0.57349300	-1.63450700
C	1.24212600	-0.79941800	-1.57710100
H	-0.77791200	-0.19594000	-1.87881600
C	-0.84931600	-1.98228400	-0.73050800
O	3.00332700	0.45473200	-0.65616200
H	2.39736600	0.74190300	-2.62553200
O	2.01183700	-1.57385500	-0.66032800
H	1.22306400	-1.27180200	-2.56272600
O	-2.26291600	-1.80054200	-0.58574800
H	-0.67597300	-2.65984200	-1.57230500
C	-0.41720100	-2.63610300	0.59265200
C	3.28674200	-0.94046100	-0.49482800
C	-2.68770200	-2.32898900	0.68676400
O	-1.63307800	-3.20223100	1.08798200
H	-0.01102400	-1.90491100	1.29318600

H	0.30033200	-3.44016600	0.44959300
C	3.77130500	-1.18399300	0.91753300
C	4.26831300	-1.41924900	-1.55829200
C	-3.94517400	-3.14943800	0.48482500
C	-2.87543100	-1.18969700	1.68406200
H	4.69094100	-0.62428000	1.09827300
H	3.01015600	-0.87489400	1.63455700
H	3.97949100	-2.24625900	1.05907600
H	3.88962200	-1.21974800	-2.56316300
H	5.22555000	-0.90713000	-1.43855200
H	4.42981100	-2.49430300	-1.45539000
H	-4.75413100	-2.51117300	0.12316900
H	-3.75882800	-3.94004300	-0.24443000
H	-4.25557200	-3.59890000	1.43071300
H	-1.94454000	-0.63933000	1.82169600
H	-3.63266500	-0.49498700	1.31315600
H	-3.20574500	-1.58780700	2.64652400
C	-0.86546600	2.52826400	0.17856300
O	-1.85592000	2.24481000	-0.64112000
O	-0.79386300	3.45812800	0.94275500
C	-3.02268800	3.13544500	-0.62508800
H	-2.68755600	4.12704000	-0.93257000
H	-3.38937900	3.18310800	0.40077000
C	-4.04098000	2.55132800	-1.57201000
H	-4.92632400	3.19256300	-1.57858800
H	-3.64804100	2.49832700	-2.59008700
H	-4.34265100	1.55060700	-1.25417900
C	1.80638500	1.72957500	2.06980100
C	2.66418500	1.83673400	3.20999700
H	3.70332000	1.78486000	2.87529300
H	2.47491300	2.79085800	3.70901000
H	2.44771200	1.01036100	3.89201600
N	1.14036100	1.63030700	1.14778000

6 NMR spectra of compounds

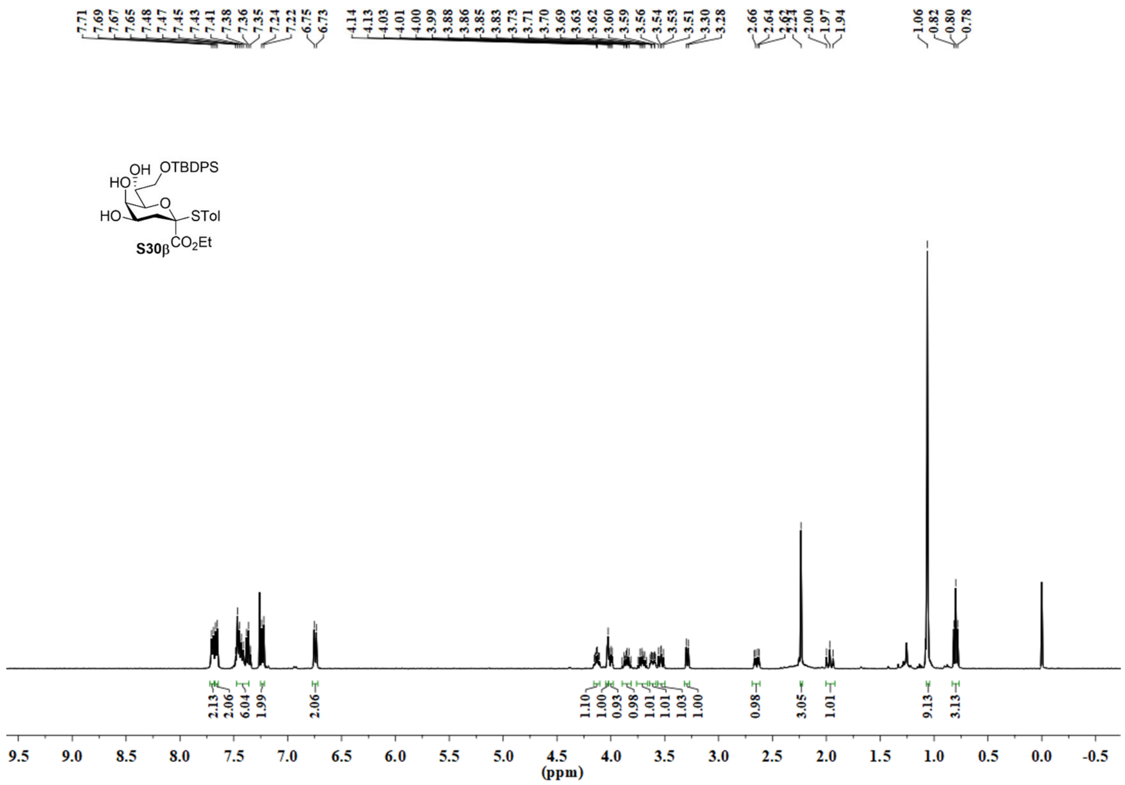

Fig. S7 ^{1}H NMR (400 MHz, $CDCl_3$) of **S30β**

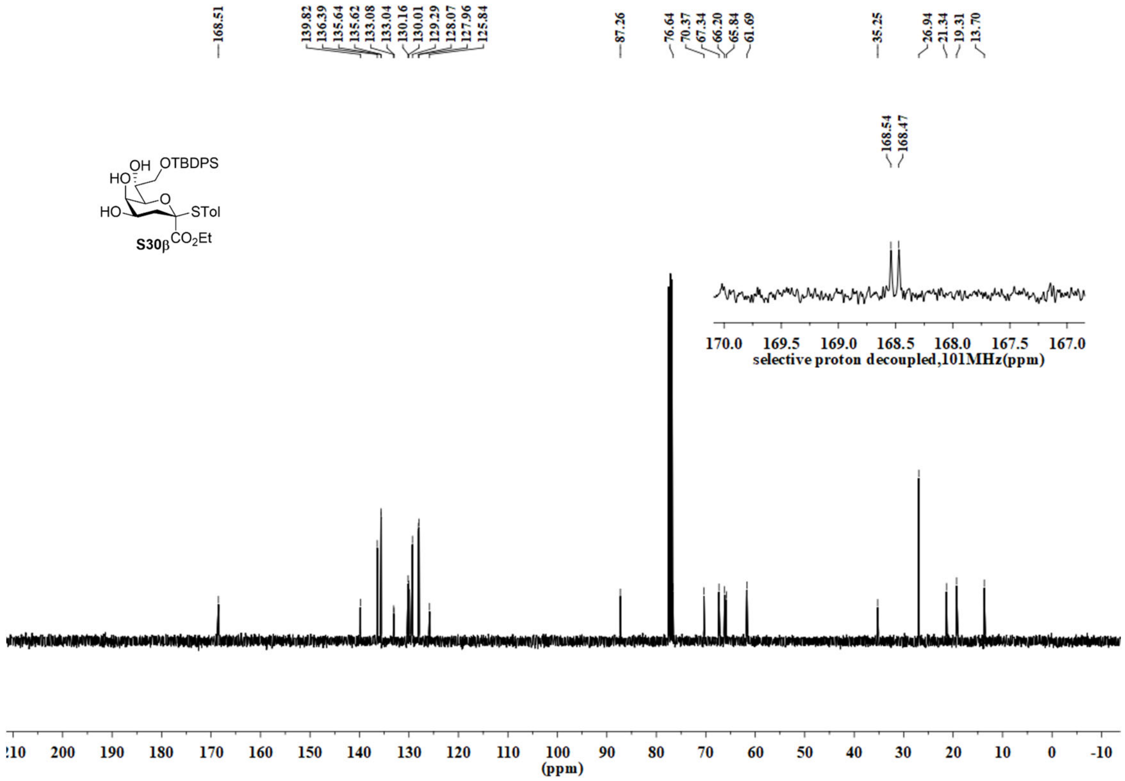

Fig. S8 ^{13}C NMR (101 MHz, $CDCl_3$) and

selective proton decoupled ^{13}C NMR (101 MHz, $CDCl_3$) of **S30β**

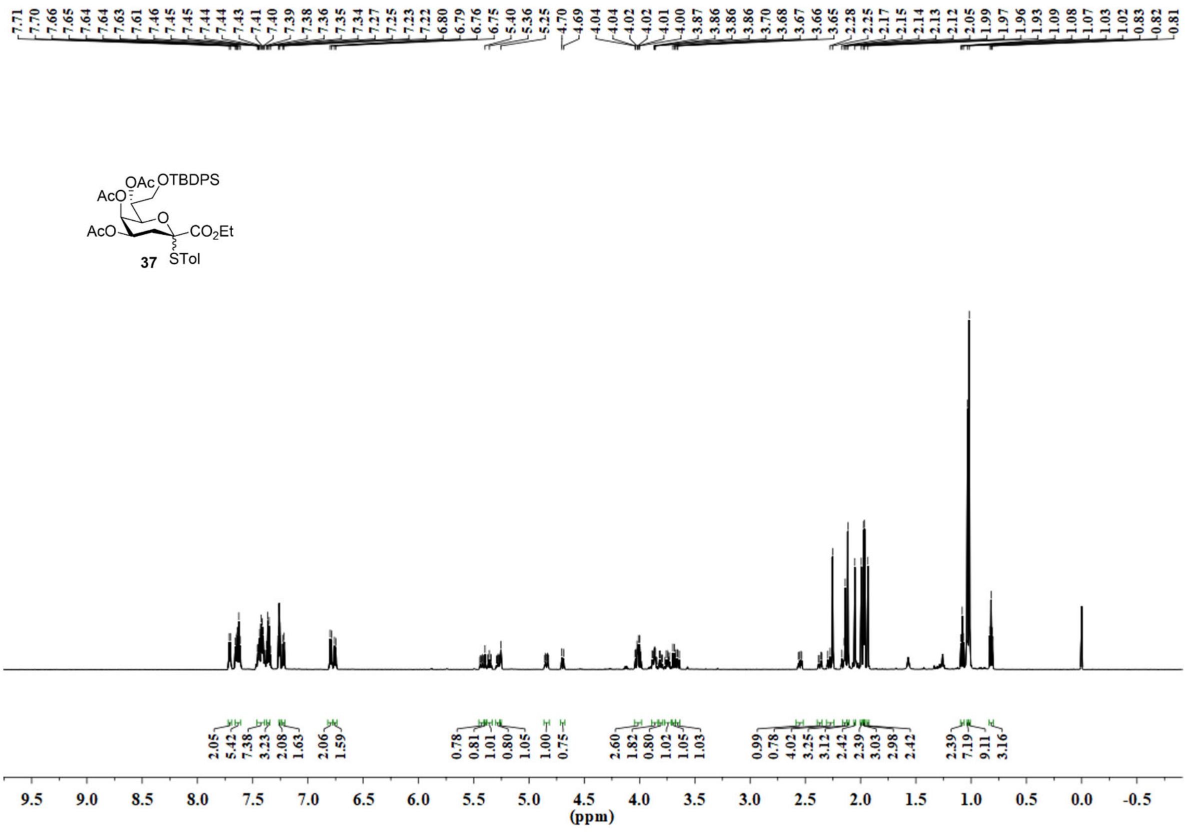

Fig. S9 ^{1}H NMR (600 MHz, $CDCl_3$) of **37**

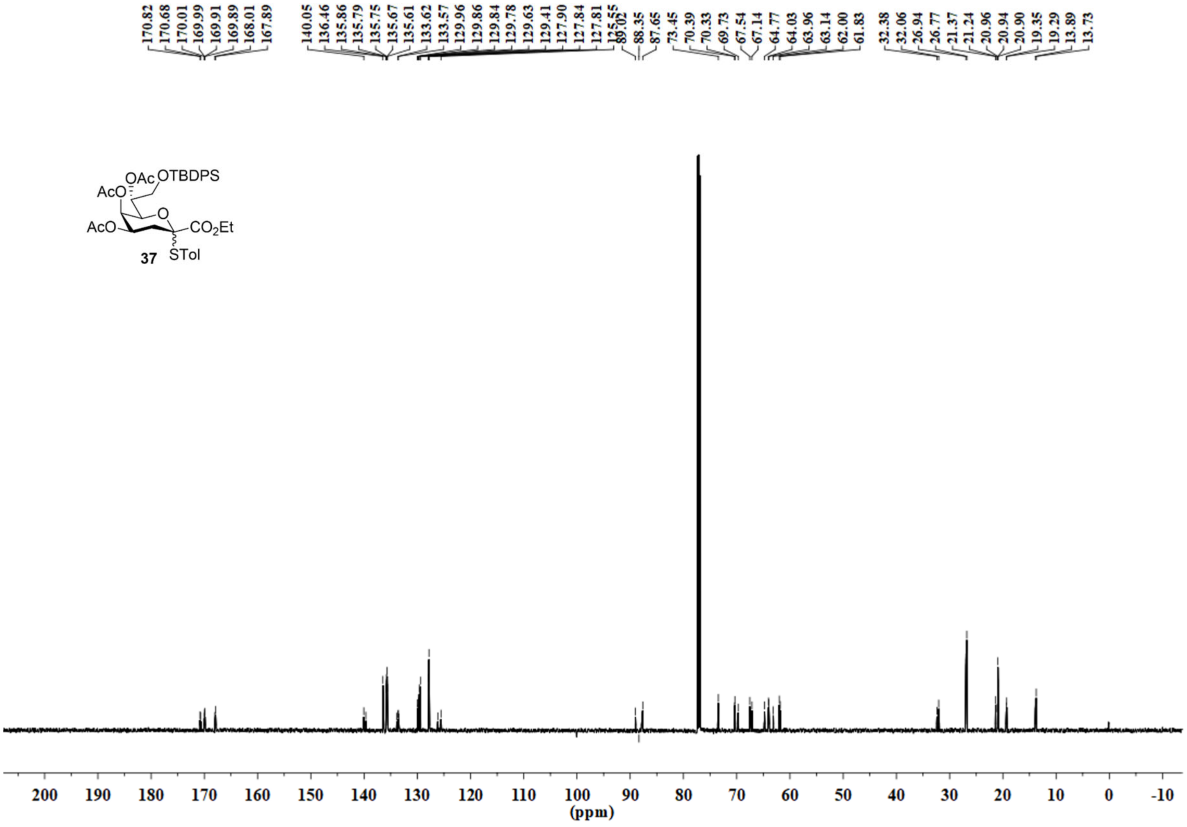

Fig. S10 ^{13}C NMR (151 MHz, $CDCl_3$) of **37**

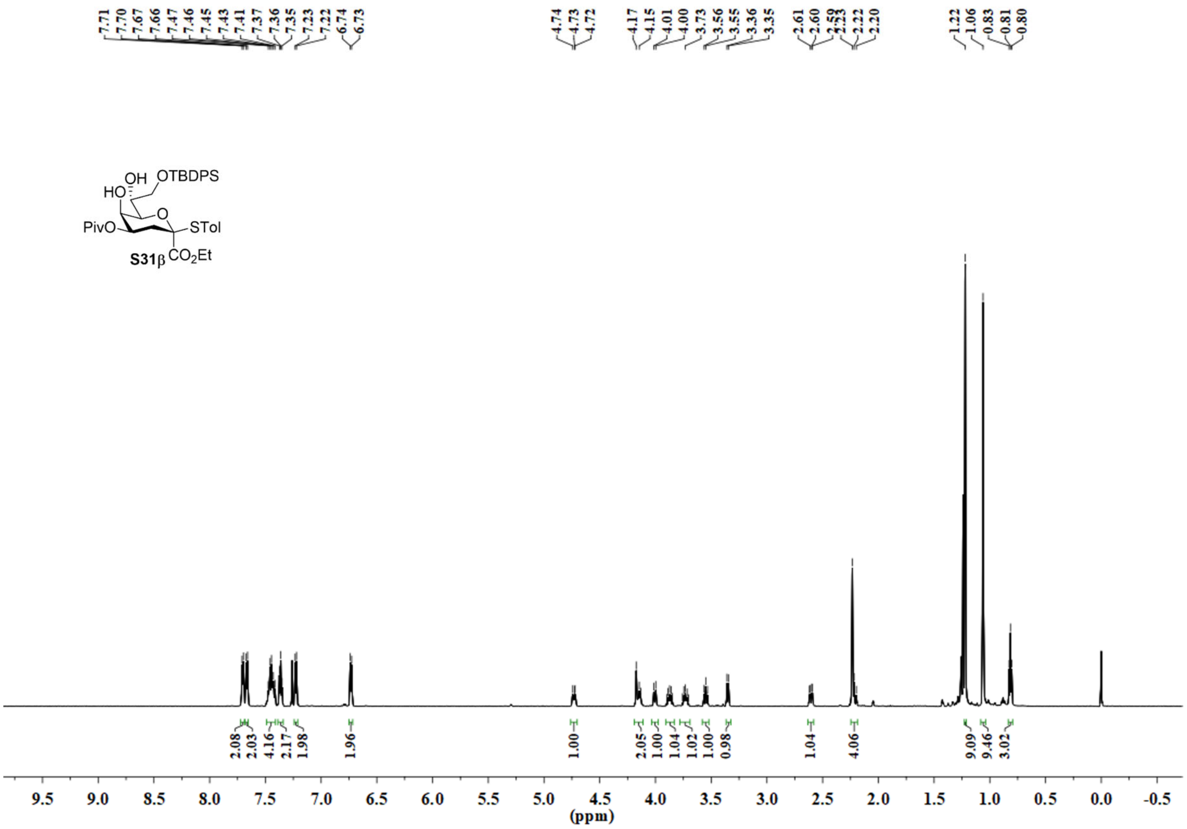

Fig. S11 ^{1}H NMR (600 MHz, $CDCl_3$) of **S31β**

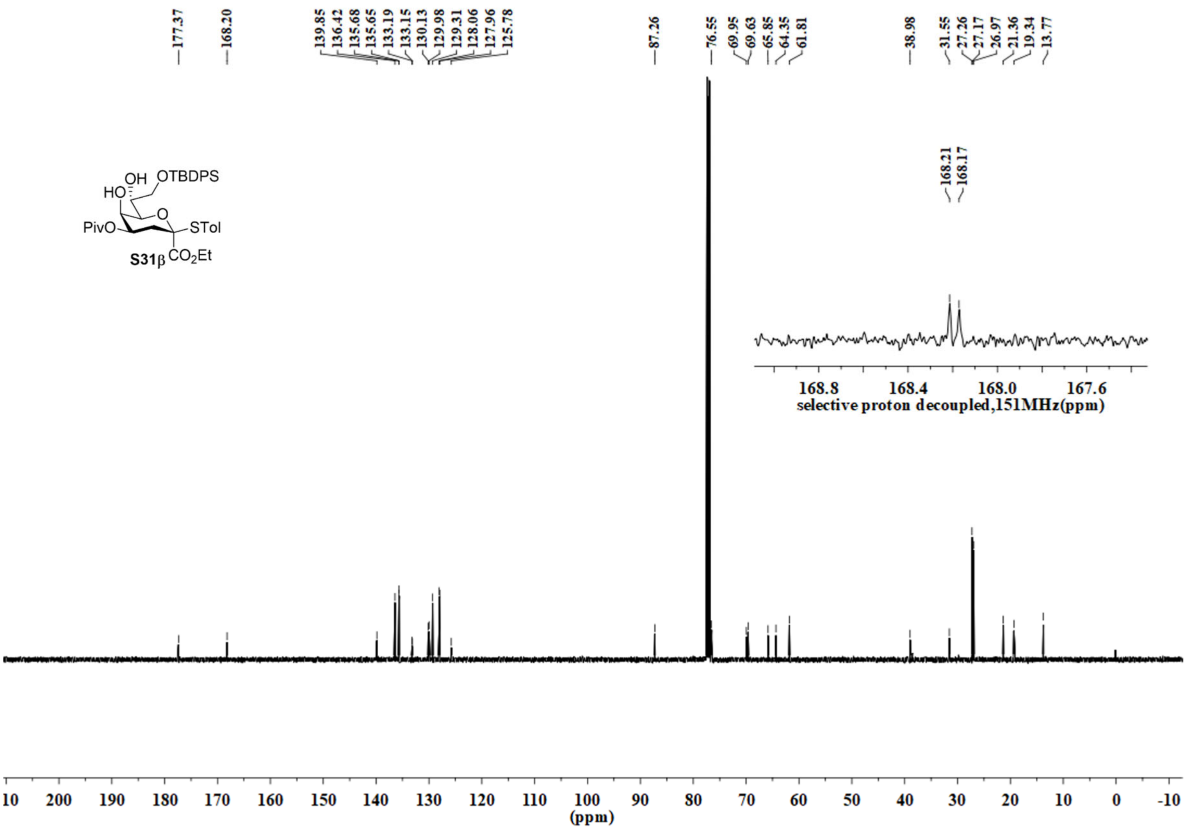

Fig. S12 ^{13}C NMR (151 MHz, $CDCl_3$) and

selective proton decoupled ^{13}C NMR (151 MHz, $CDCl_3$) of **S31β**

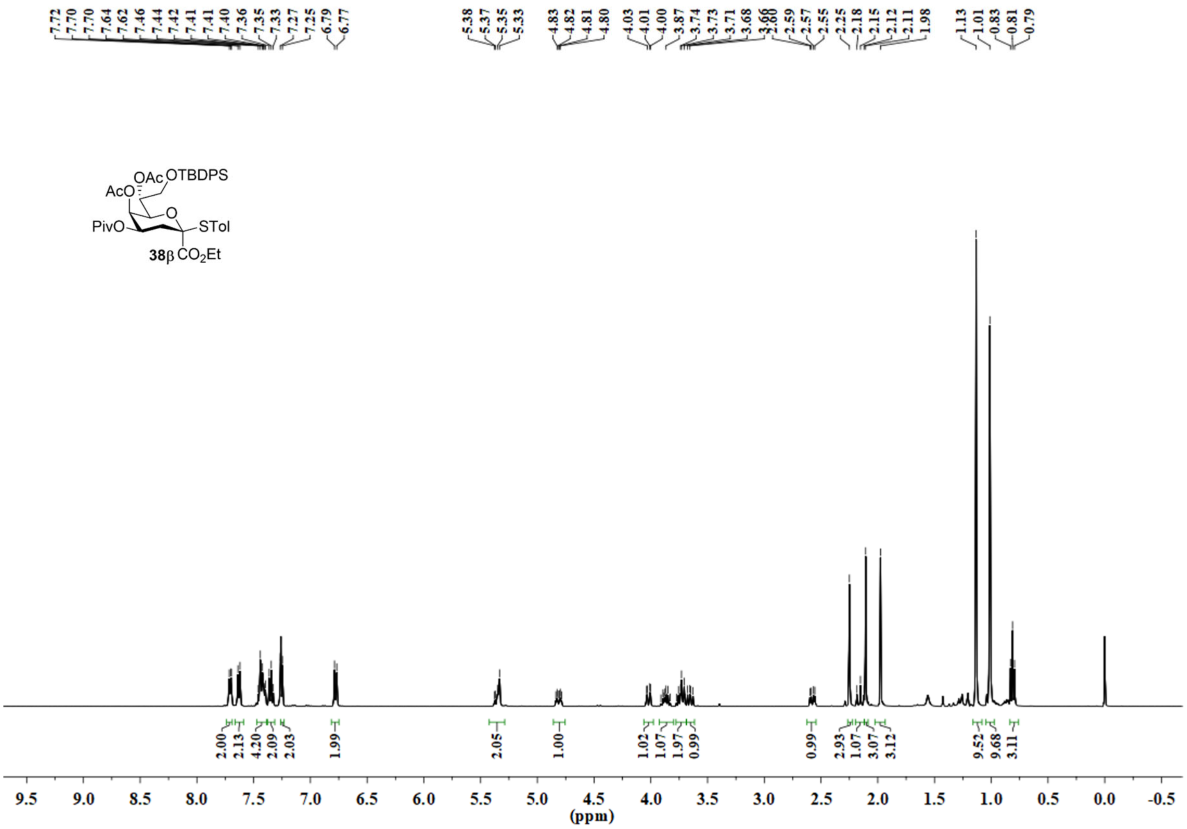

Fig. S13 ^{1}H NMR (400 MHz, CDCl3) of **38β**

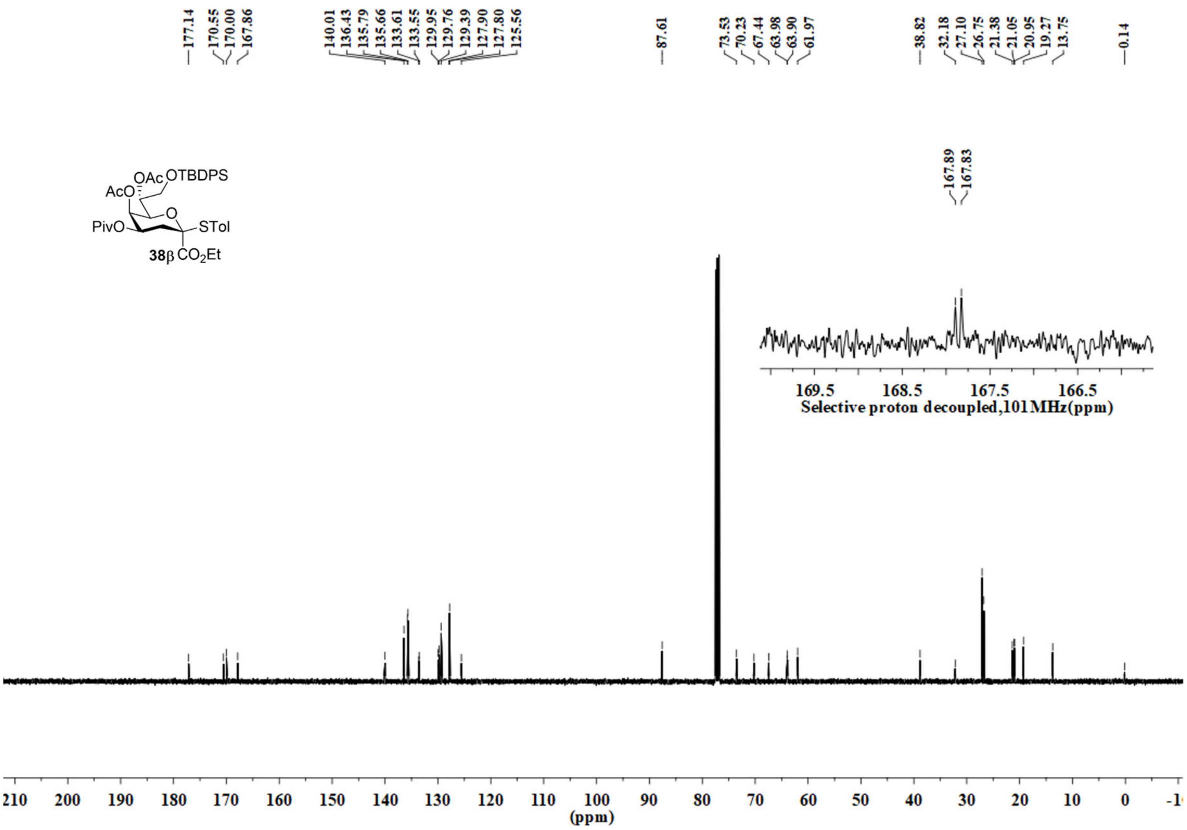

Fig. S14 ^{13}C NMR (101 MHz, CDCl3) and

selective proton decoupled ^{13}C NMR (101 MHz, CDCl3) of **38β**

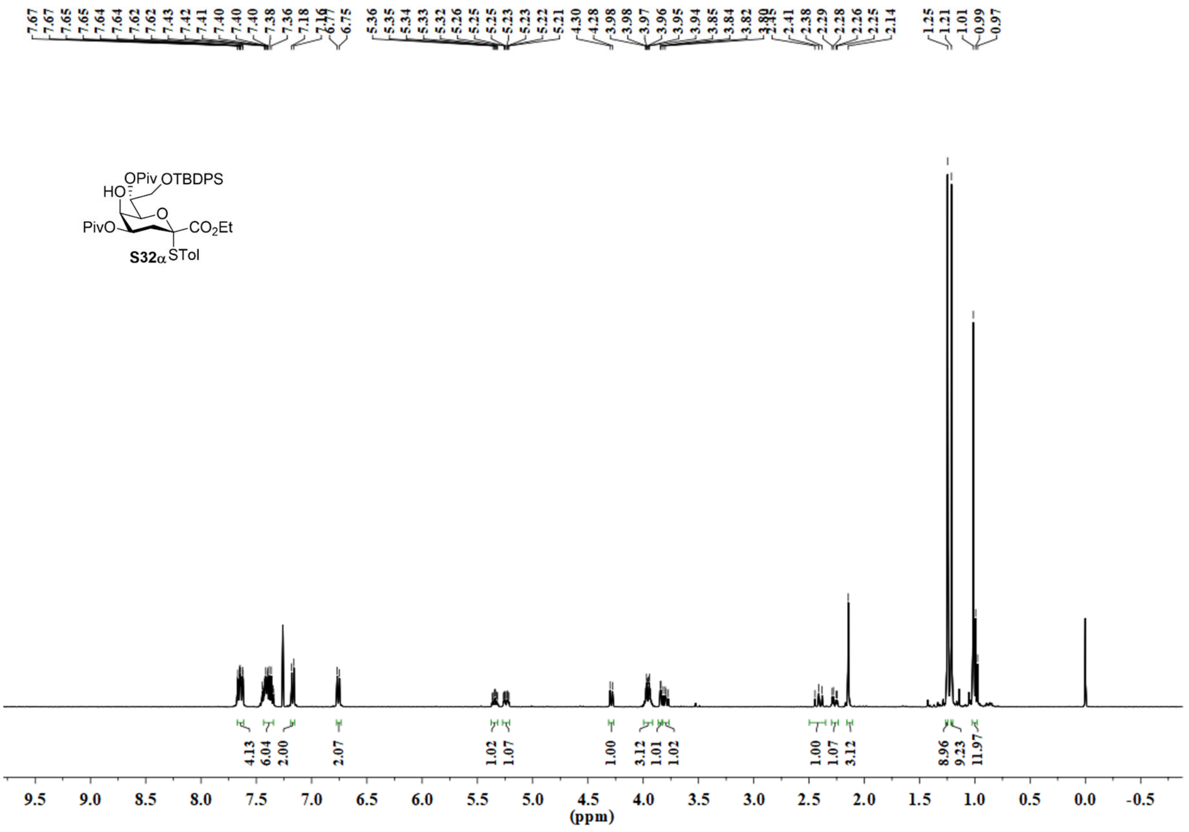

Fig. S15 ^{1}H NMR (400 MHz, $CDCl_3$) of **S30α**

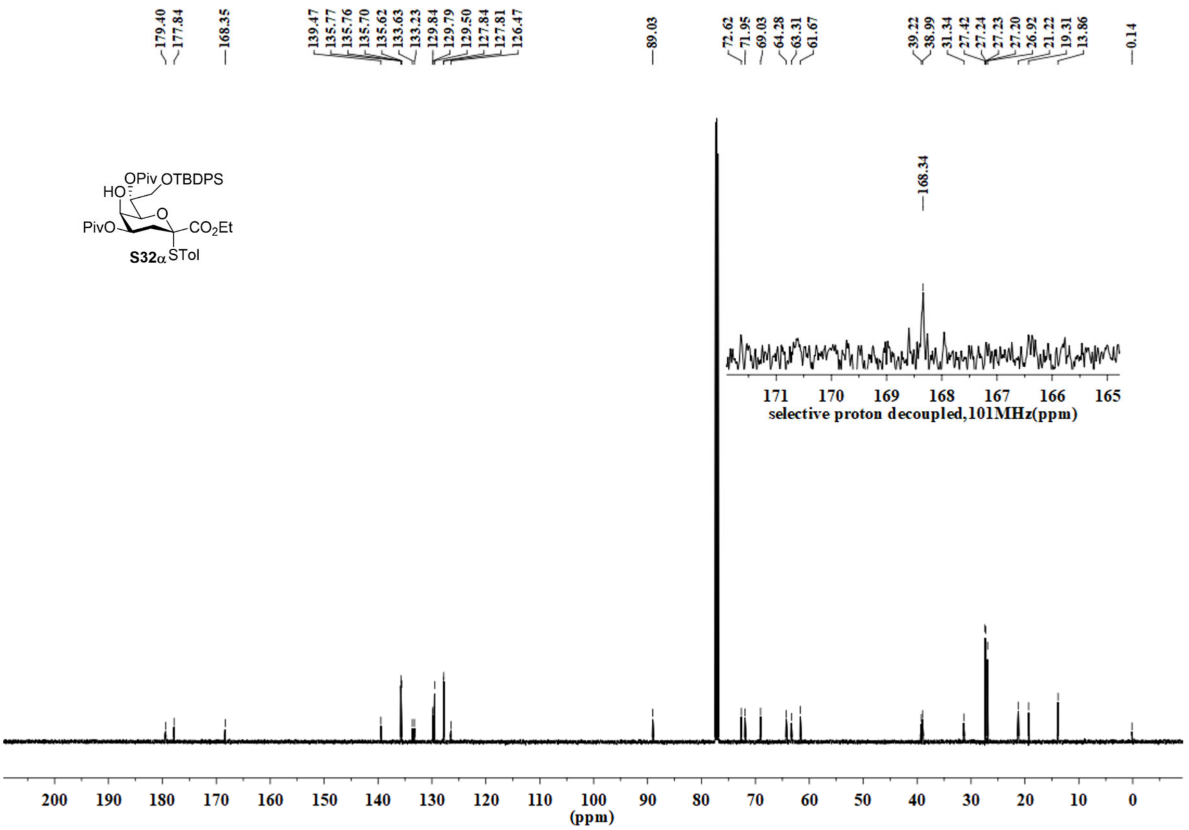

Fig. S16 ^{13}C NMR (151 MHz, $CDCl_3$) and

selective proton decoupled ^{13}C NMR (101 MHz, $CDCl_3$) of **S32α**

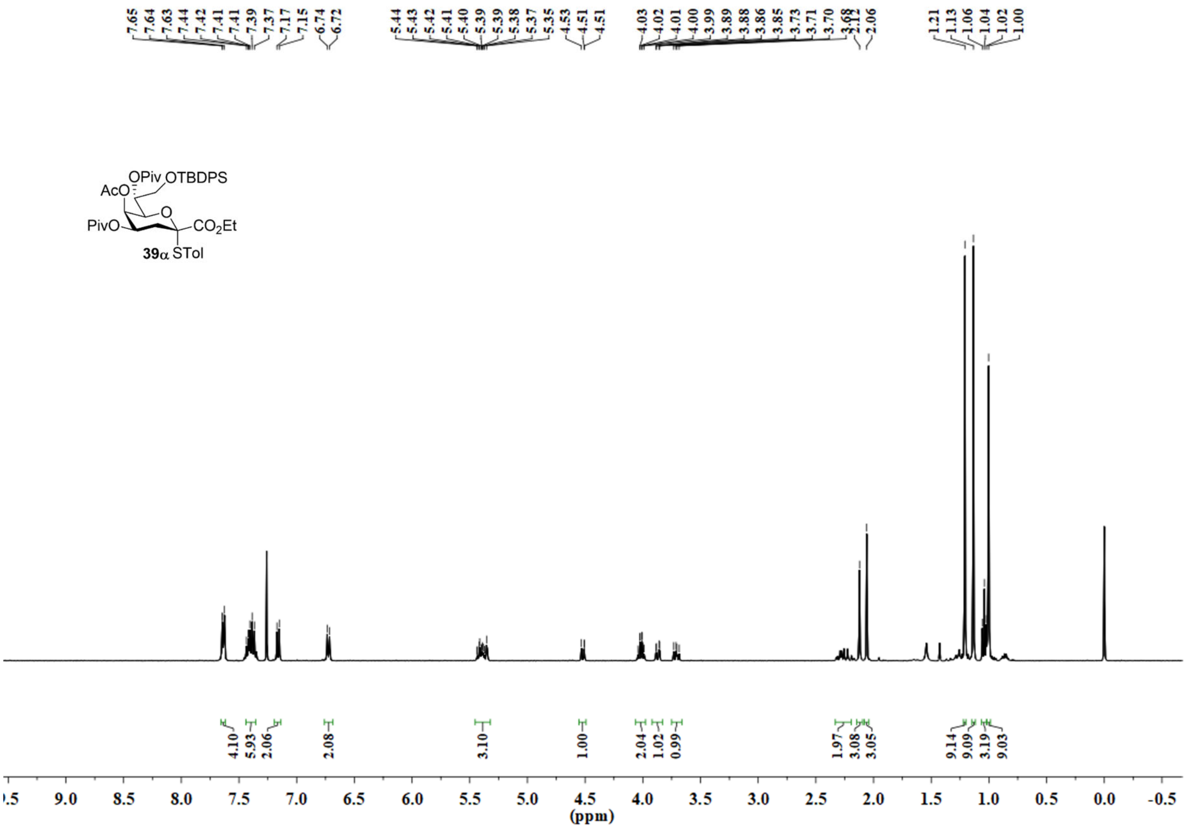

Fig. S17 ^{1}H NMR (400 MHz, CDCl3) of **39α**

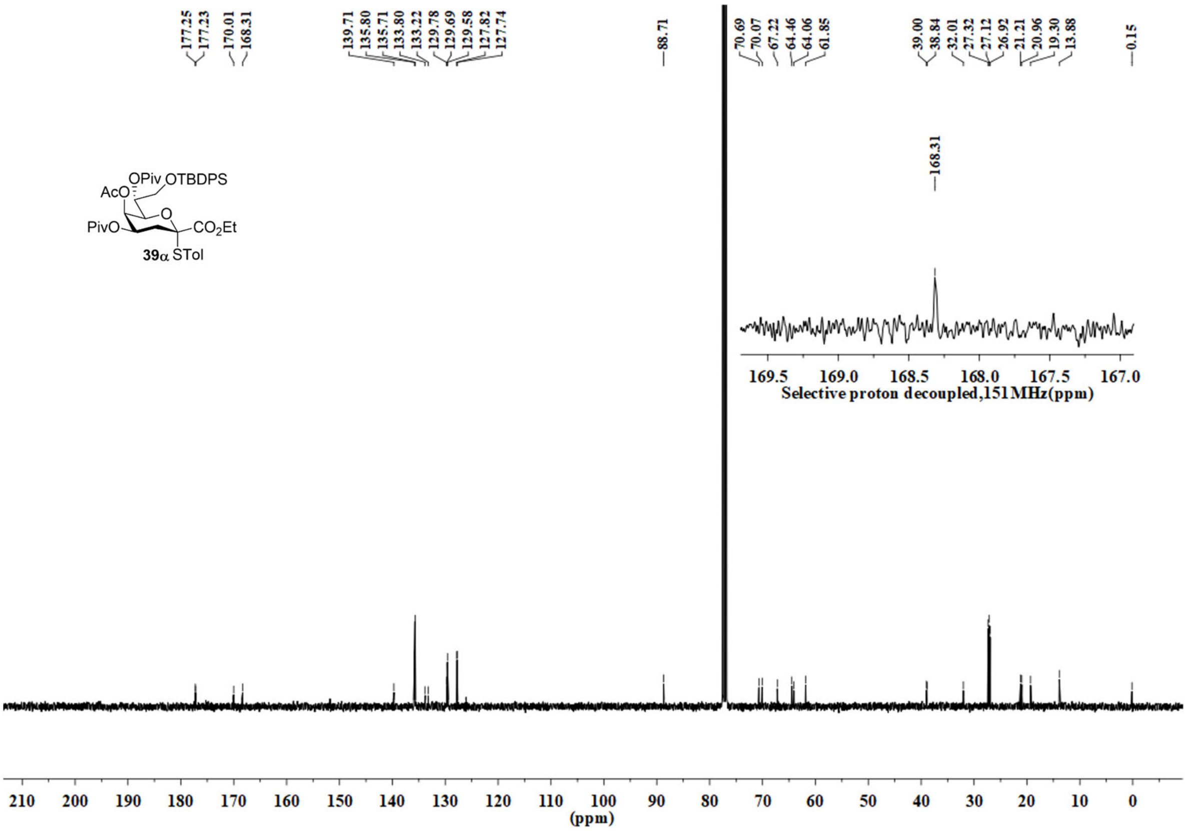

Fig. S18 ^{13}C NMR (101 MHz, CDCl3) and

selective proton decoupled ^{13}C NMR (151 MHz, CDCl3) of **39α**

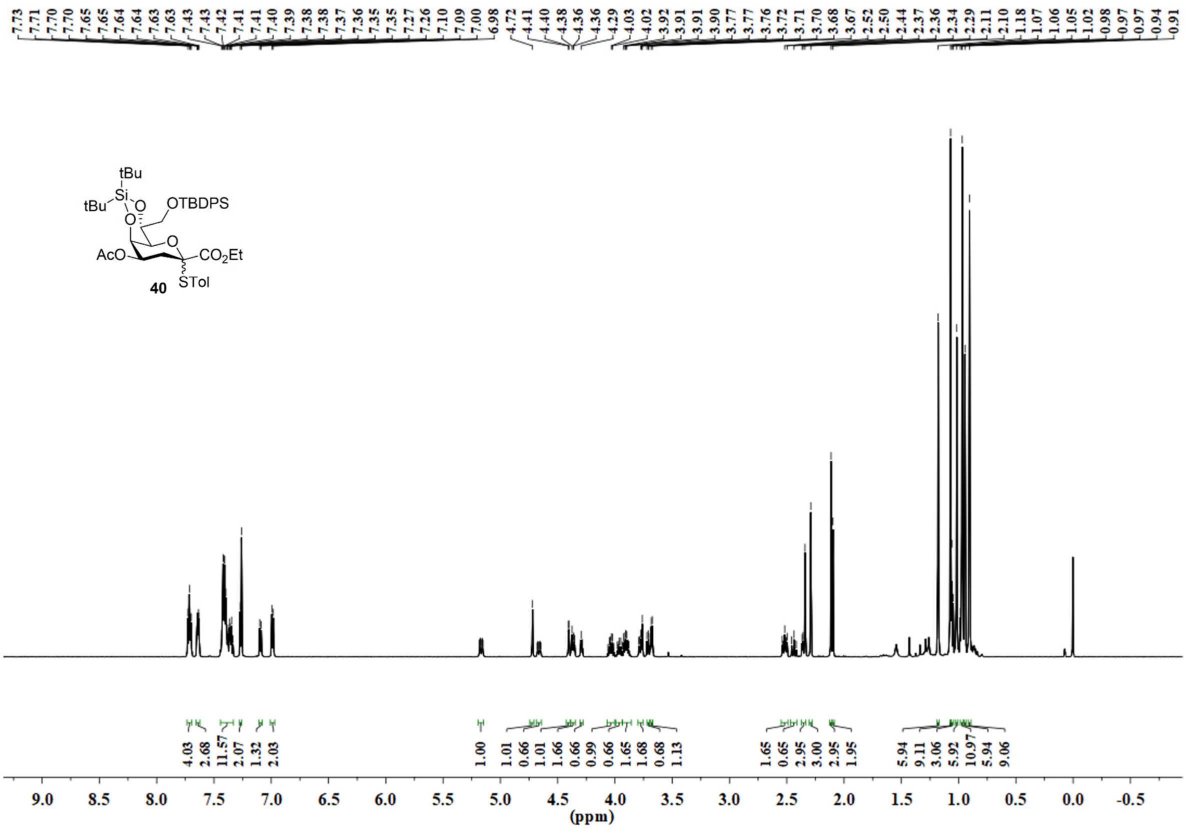

Fig. S19 ^{1}H NMR (600 MHz, $CDCl_3$) of **40**

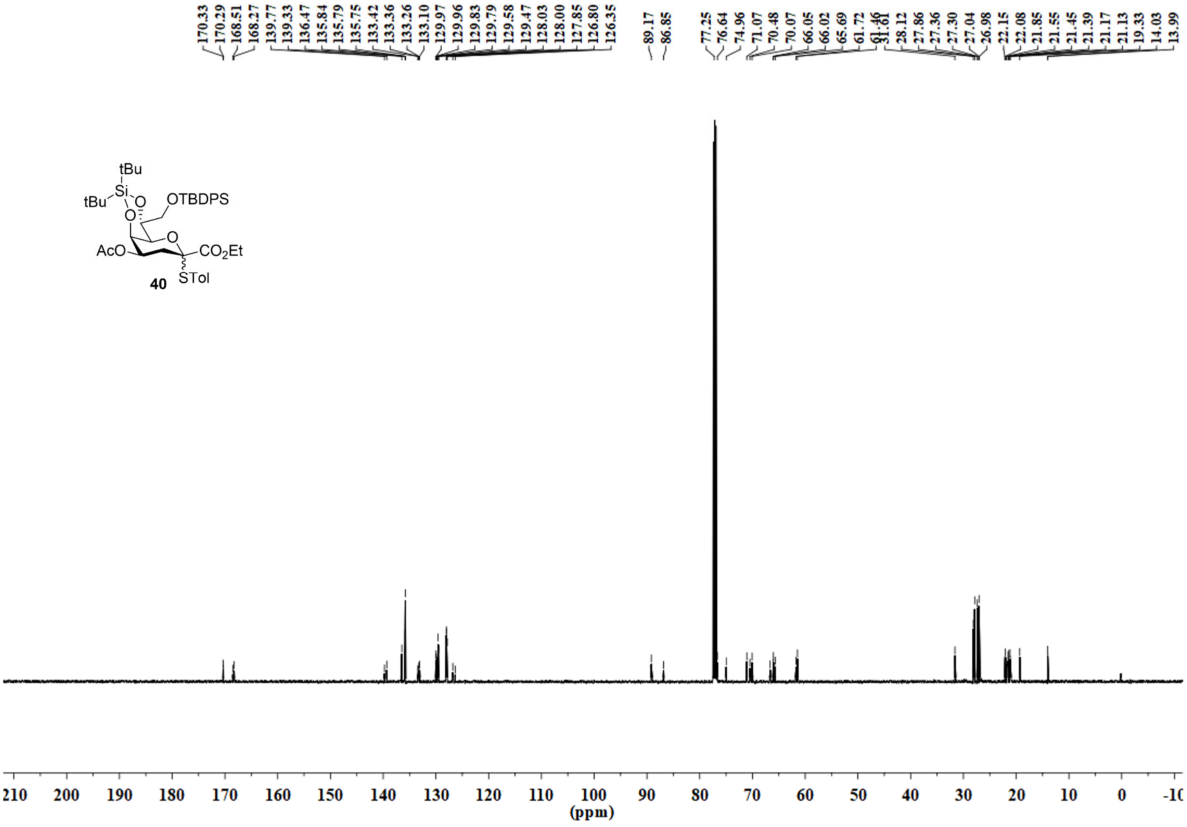

Fig. S20 ^{13}C NMR (151 MHz, $CDCl_3$) of **40**

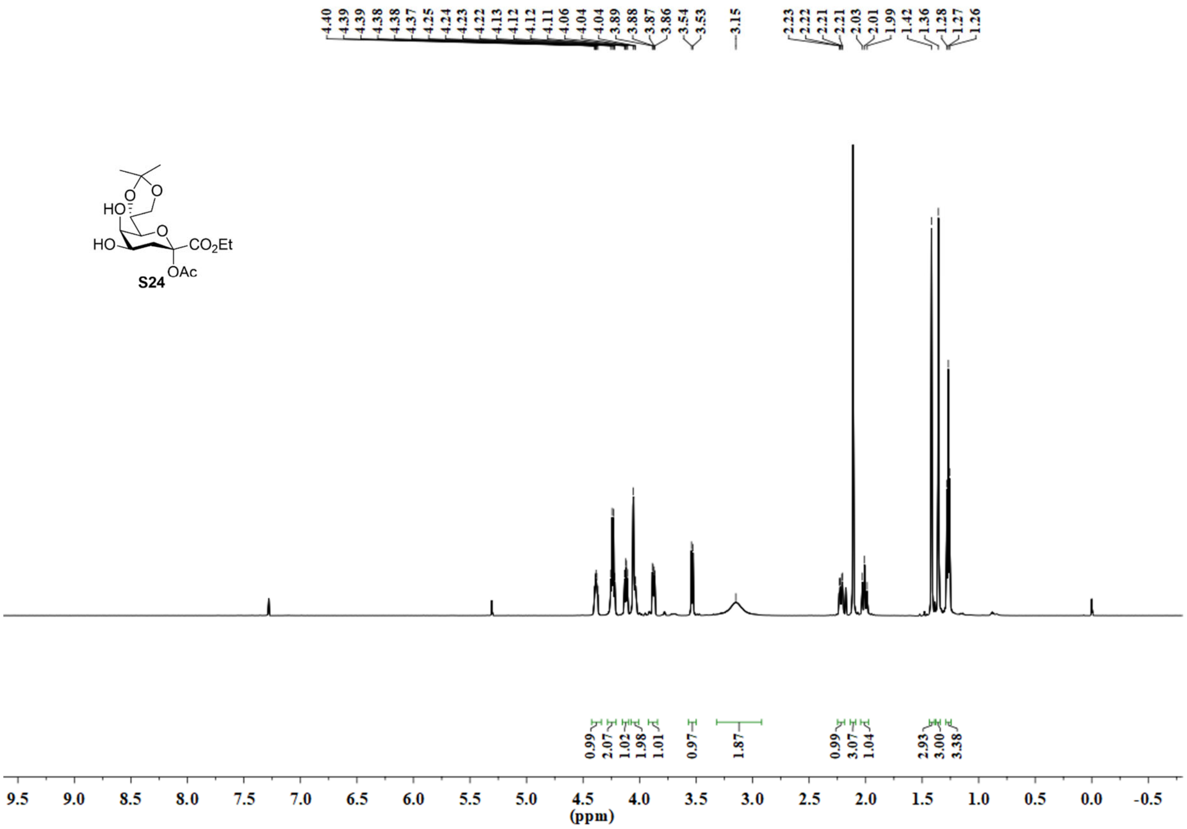

Fig. S21 ^{1}H NMR (600 MHz, $CDCl_3$) of **S24**

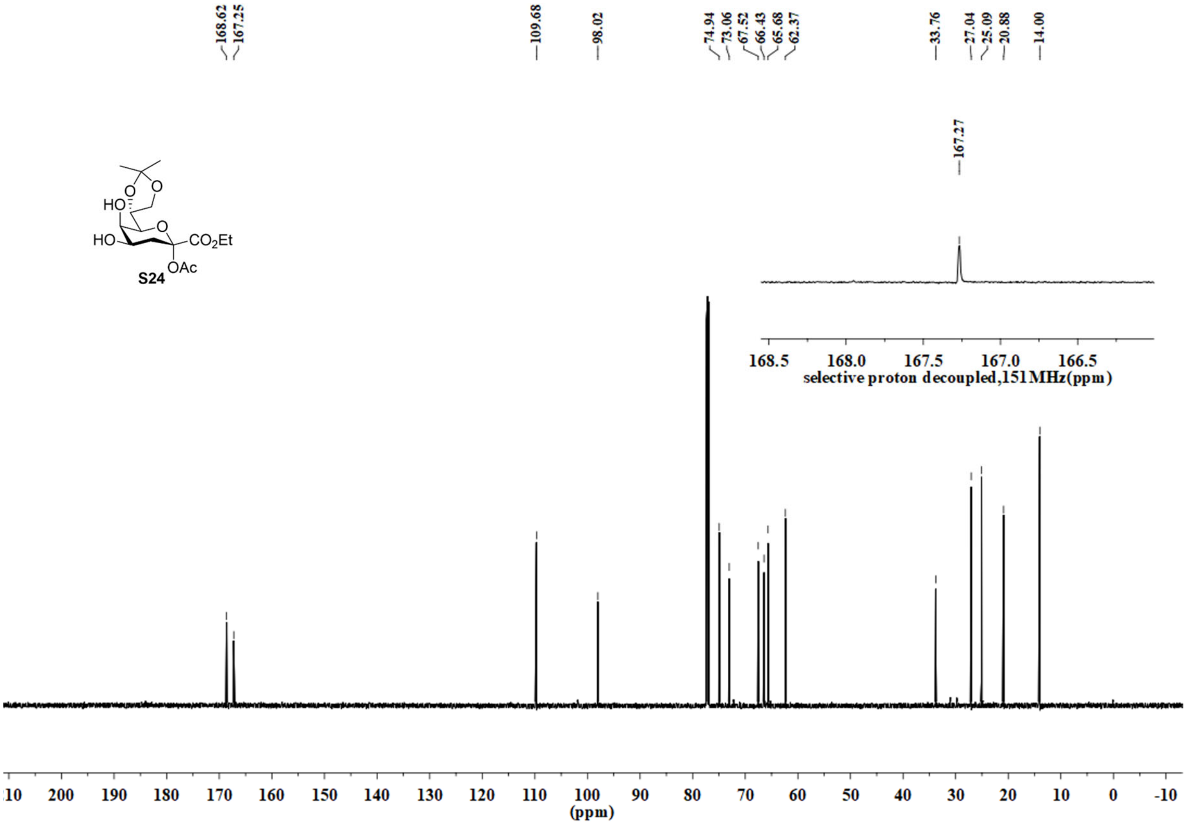

Fig. S22 ^{13}C NMR (151 MHz, $CDCl_3$) and

selective proton decoupled ^{13}C NMR (151 MHz, $CDCl_3$) of **S24**

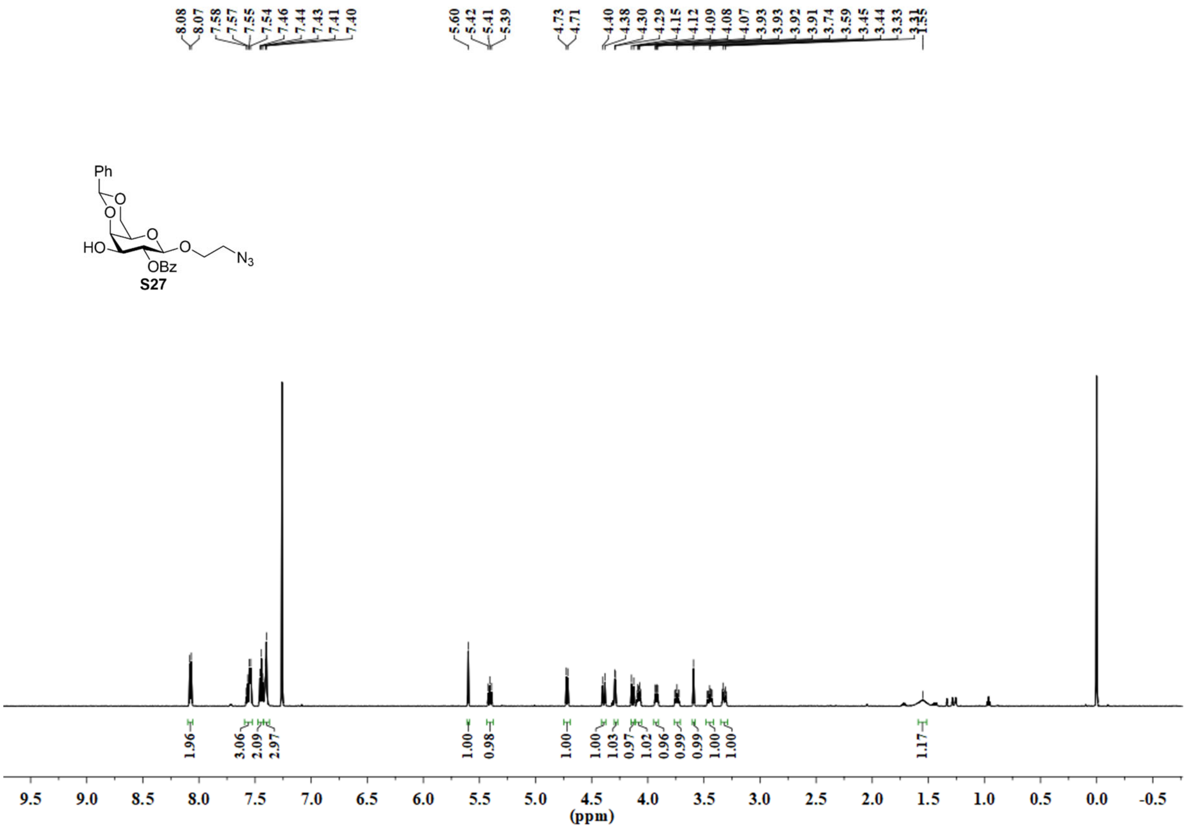

Fig. S23 ^{1}H NMR (600 MHz, $CDCl_3$) of **S27**

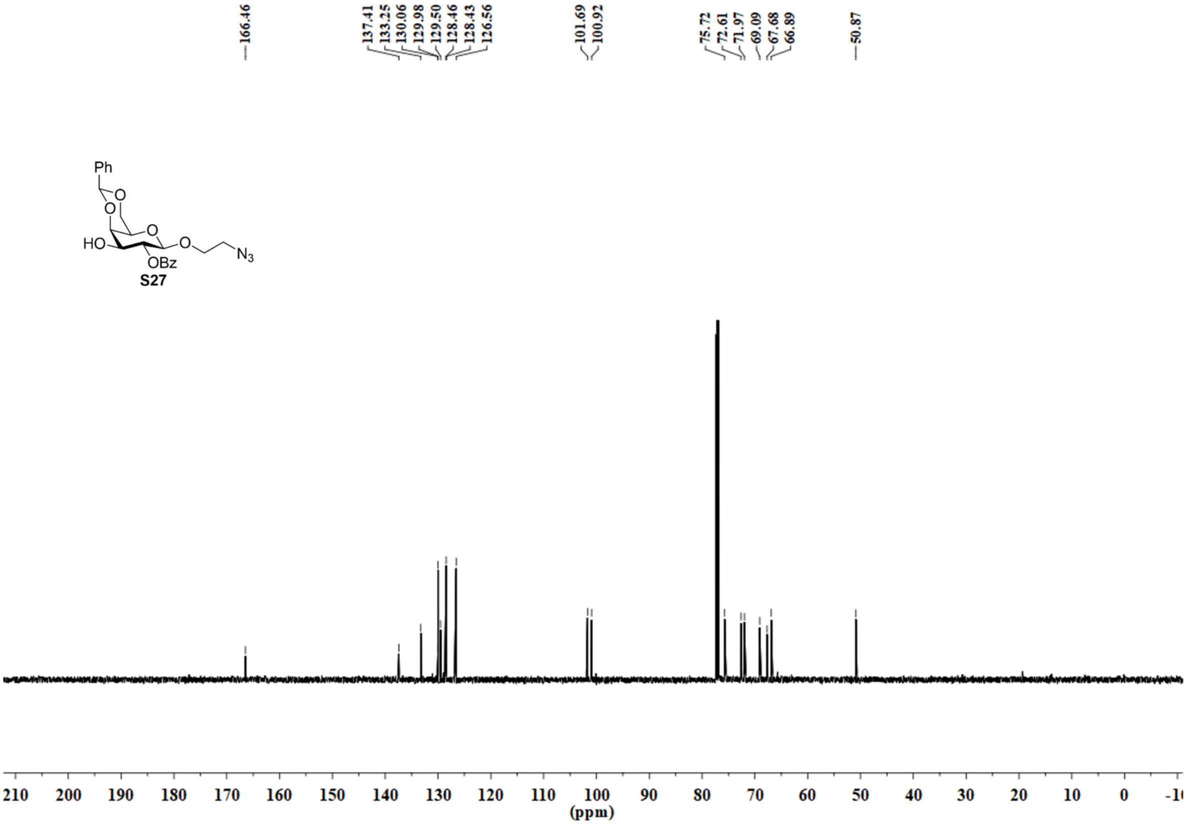

Fig. S24 ^{13}C NMR (151 MHz, $CDCl_3$) of **S27**

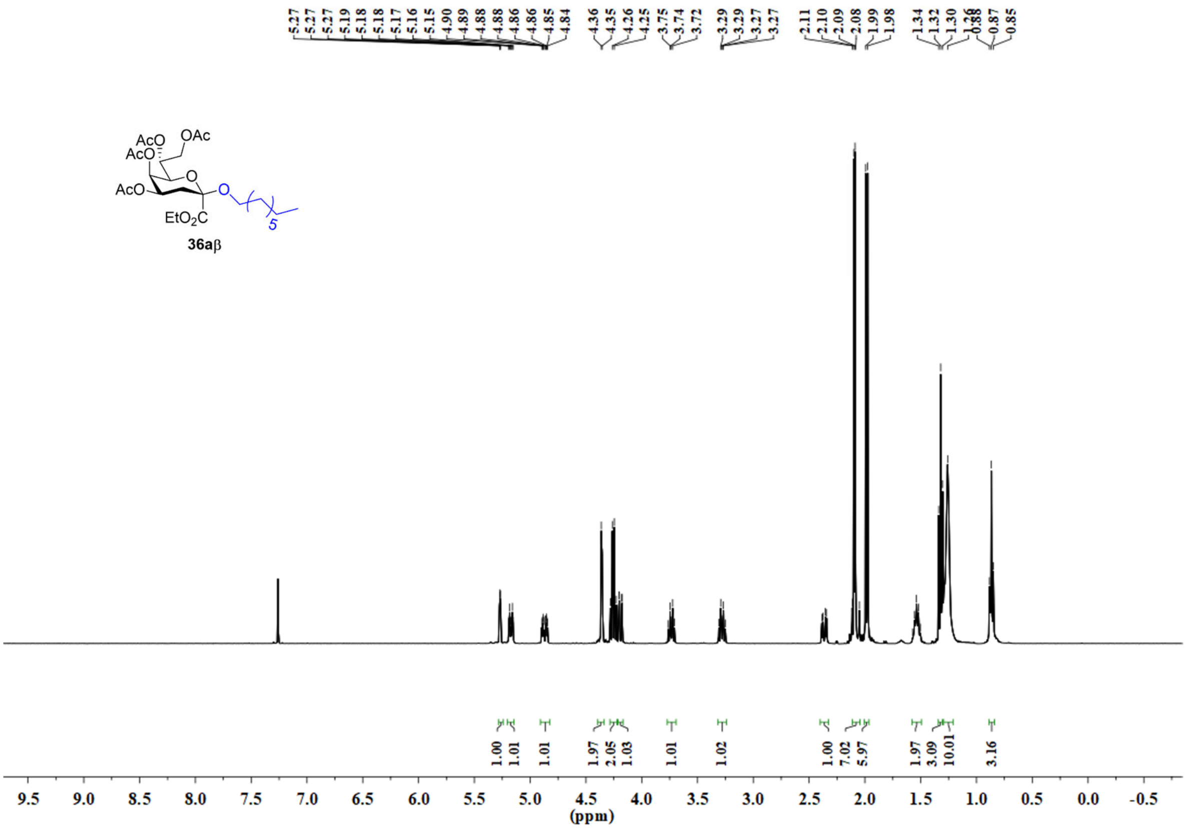

Fig. S25 ^{1}H NMR (400 MHz, CDCl3) of **36aβ**

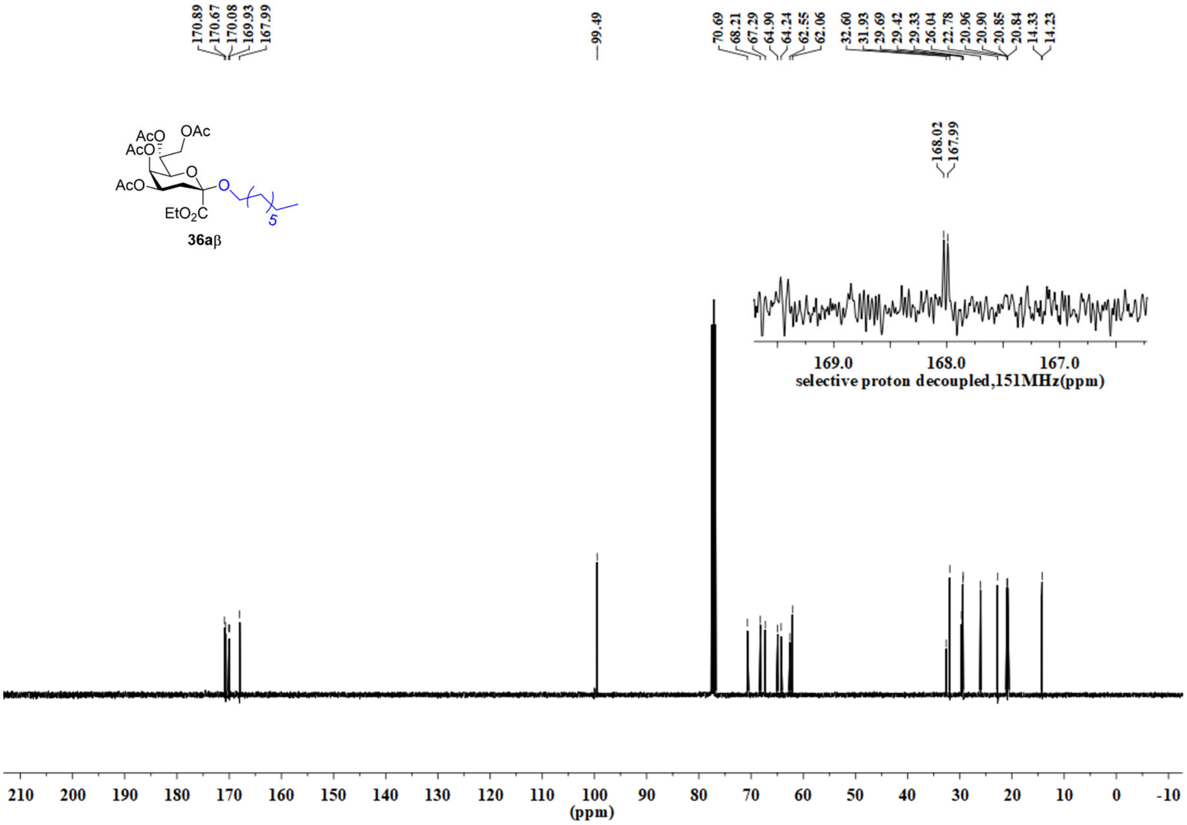

Fig. S26 ^{13}C NMR (101 MHz, CDCl3) and

selective proton decoupled ^{13}C NMR (151 MHz, CDCl3) of **36aβ**

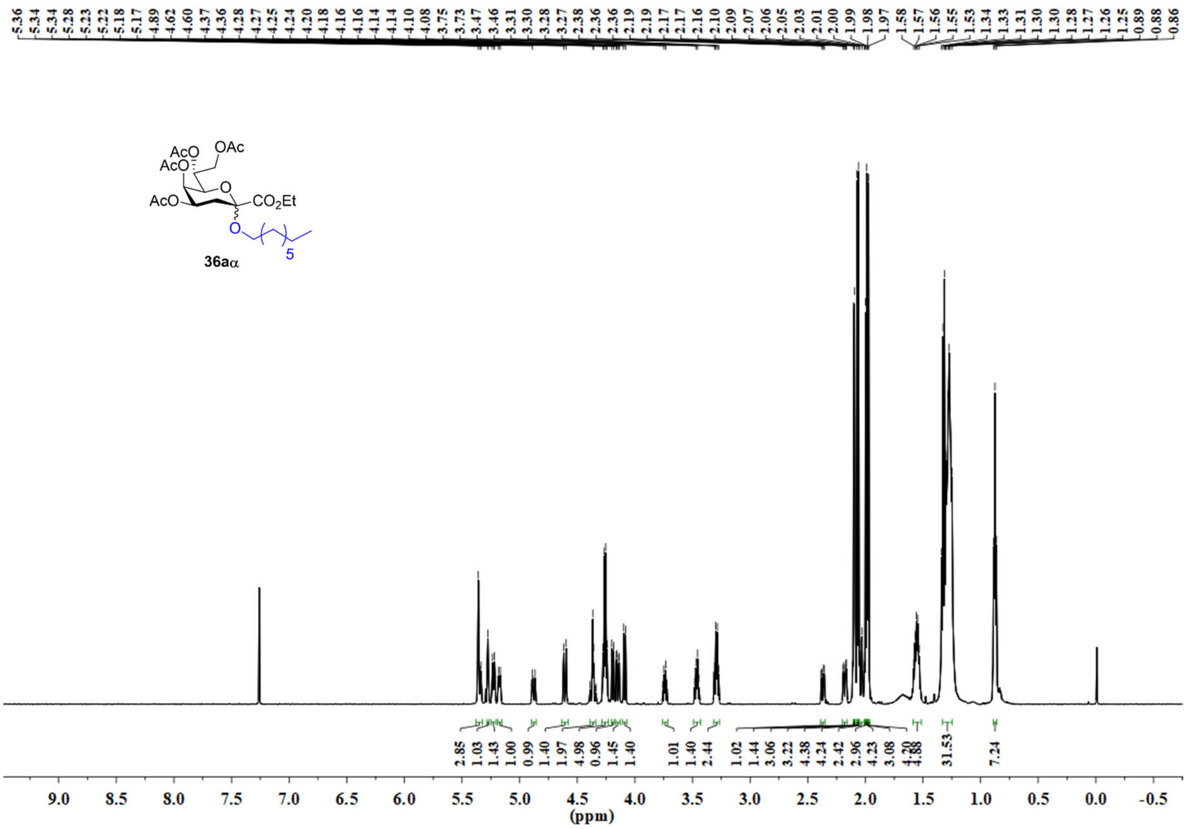

Fig. S27 ^{1}H NMR (600 MHz, $CDCl_3$) of **36aα**

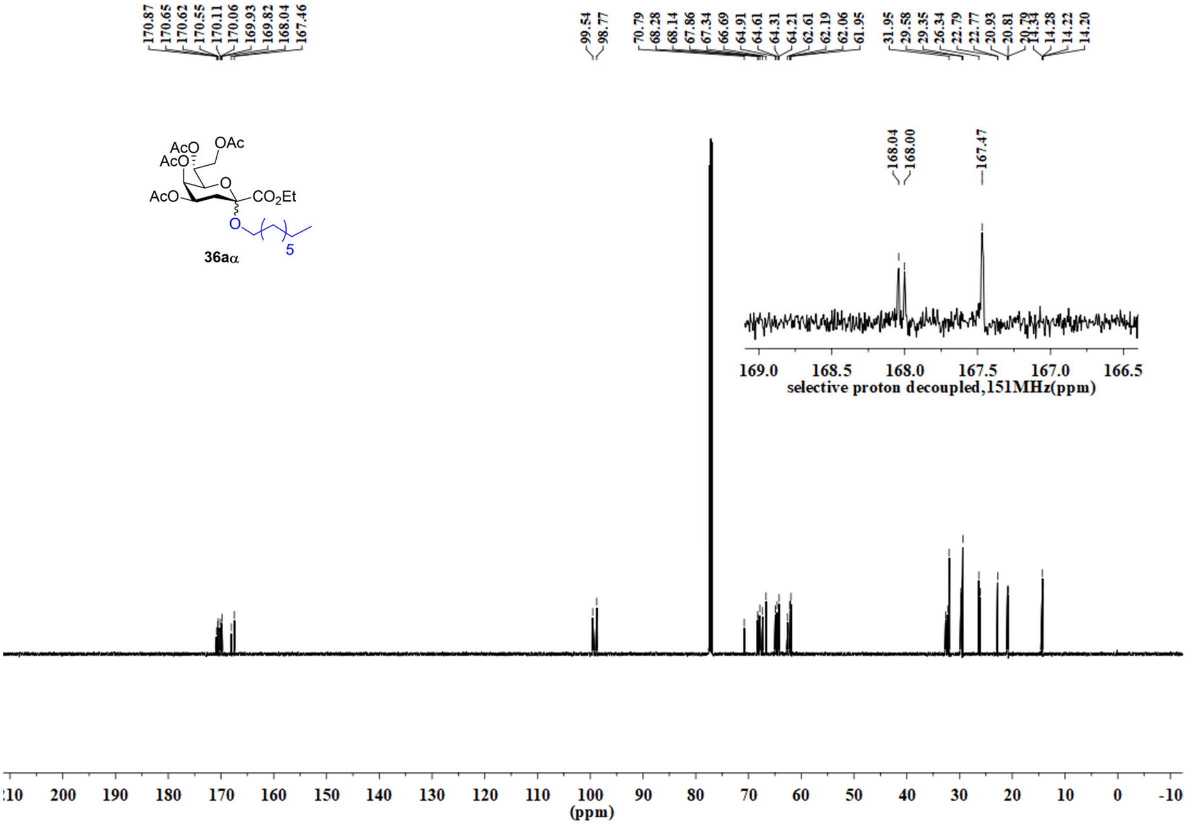

Fig. S28 ^{13}C NMR (151 MHz, $CDCl_3$) and

selective proton decoupled ^{13}C NMR (151 MHz, $CDCl_3$) of **36aα**

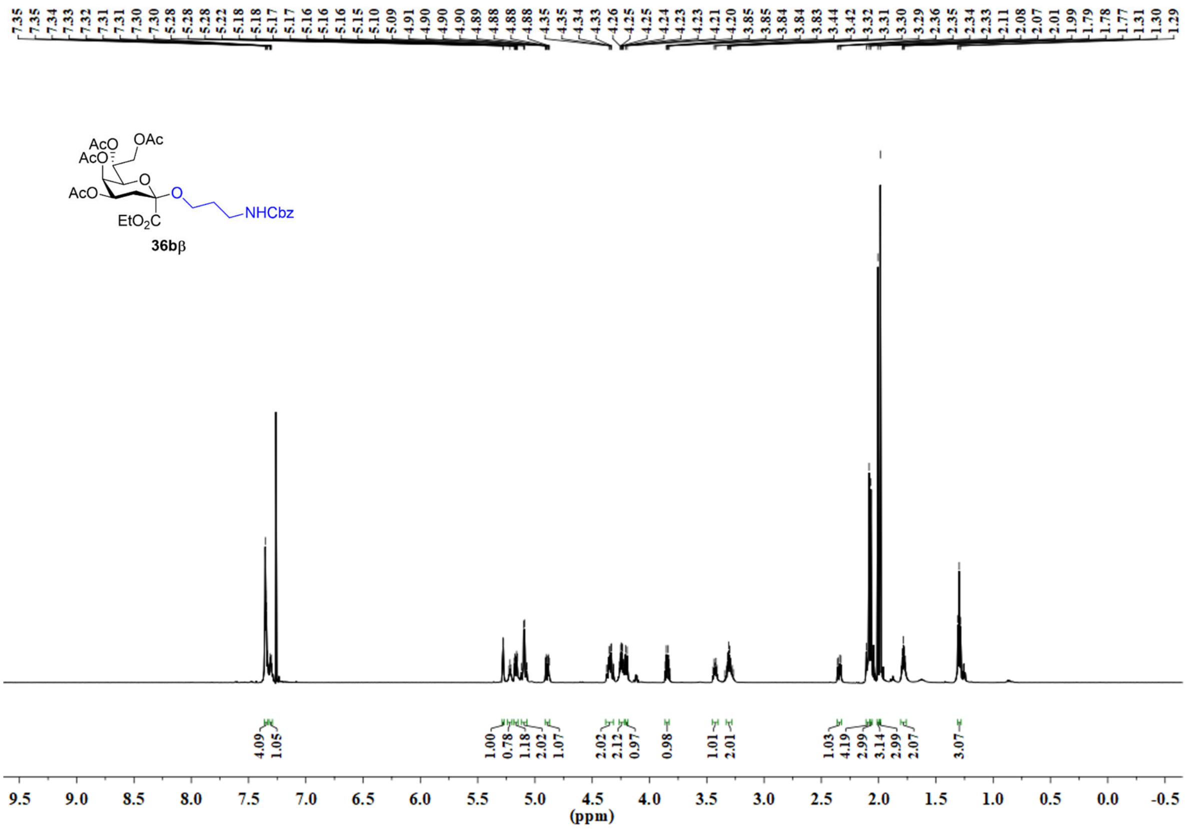

Fig. S29 ^{1}H NMR (600 MHz, $CDCl_3$) of **36bβ**

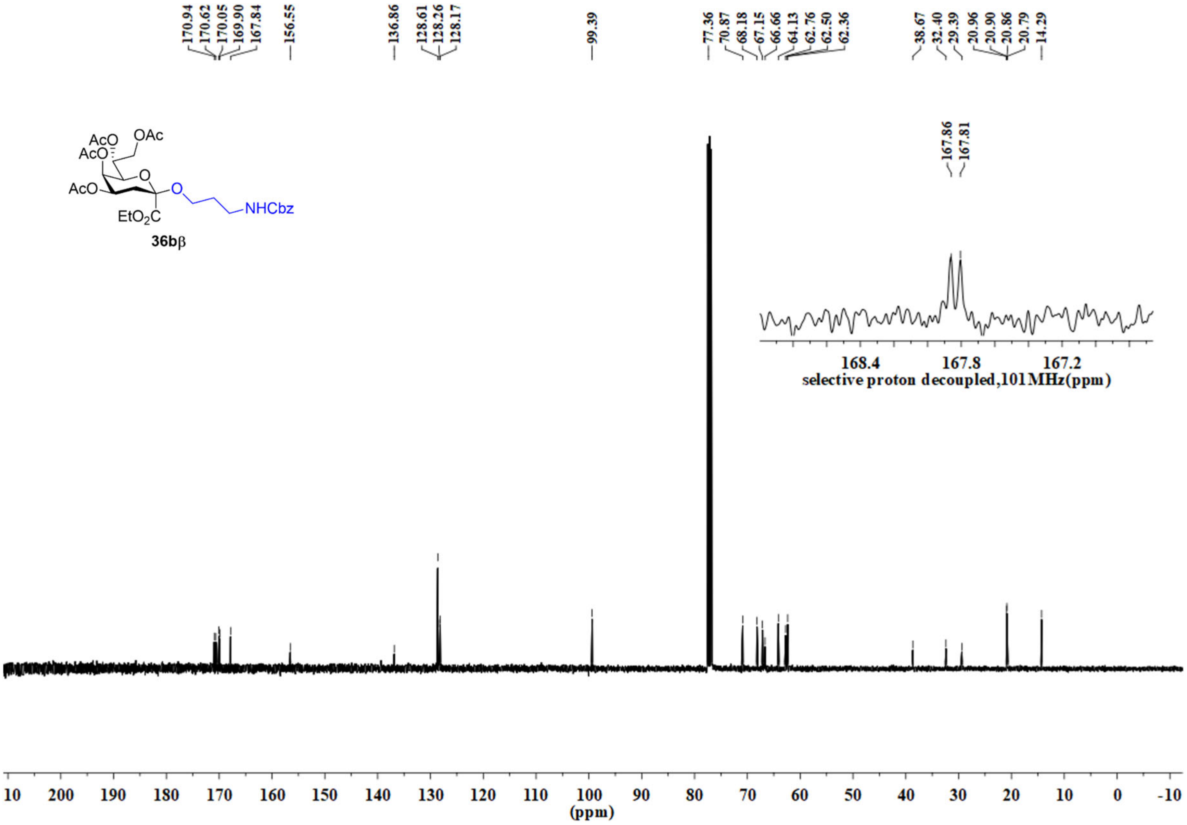

Fig. S30 ^{13}C NMR (101 MHz, $CDCl_3$) and

selective proton decoupled ^{13}C NMR (101 MHz, $CDCl_3$) of **36bβ**

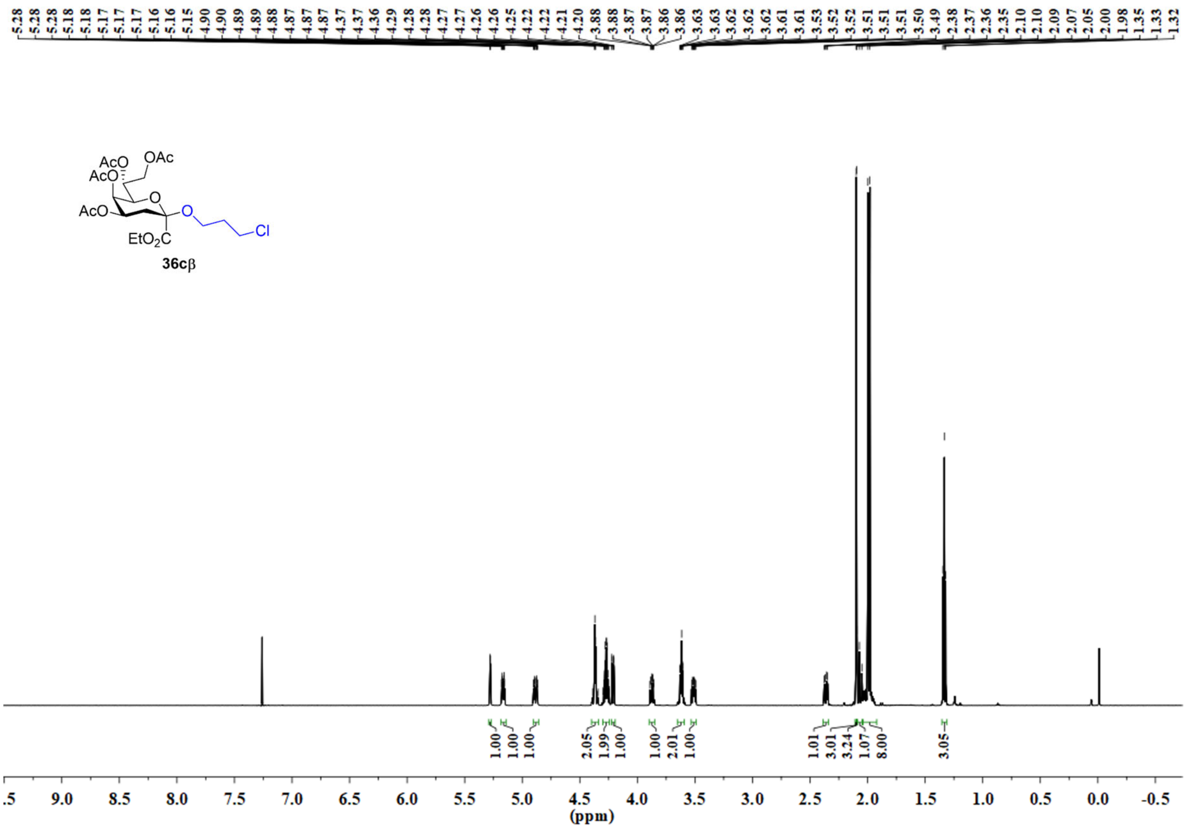

Fig. S31 ^{1}H NMR (600 MHz, $CDCl_3$) of **36cβ**

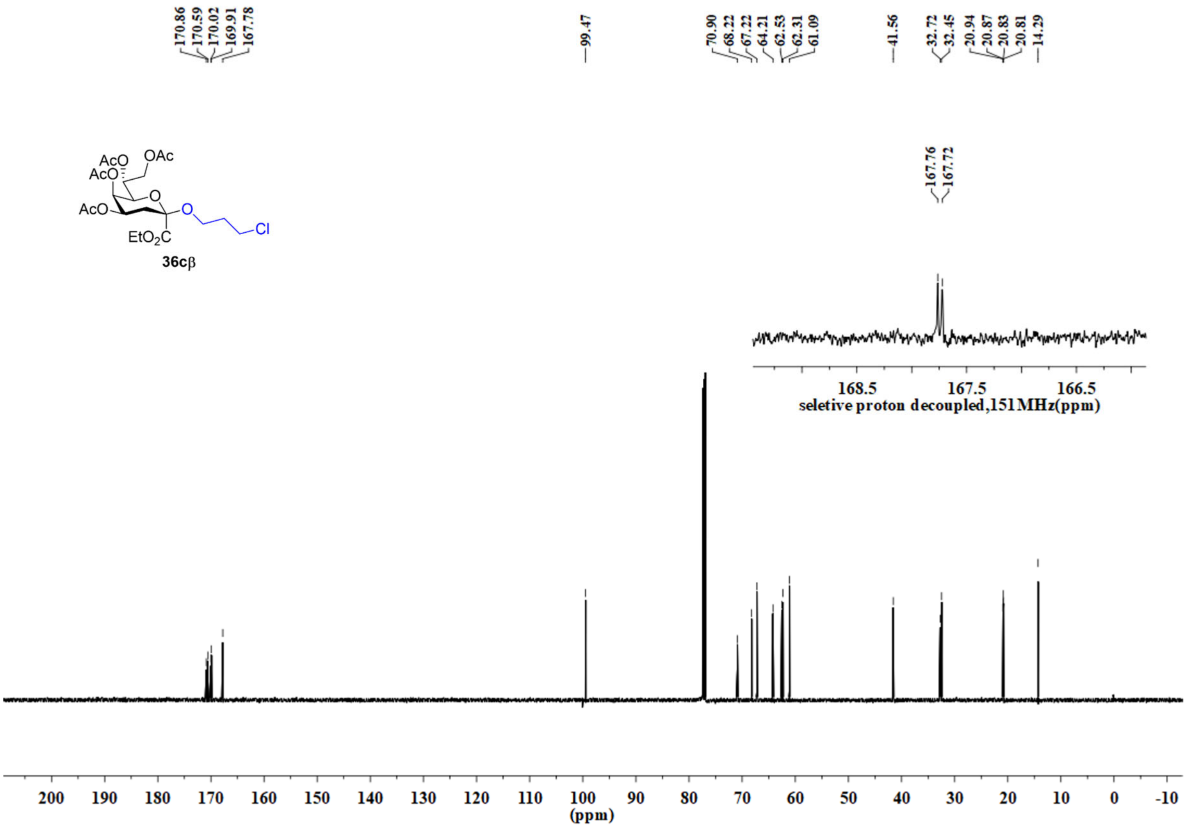

Fig. S32 ^{13}C NMR (151 MHz, $CDCl_3$) and

selective proton decoupled ^{13}C NMR (151 MHz, $CDCl_3$) of **36cβ**

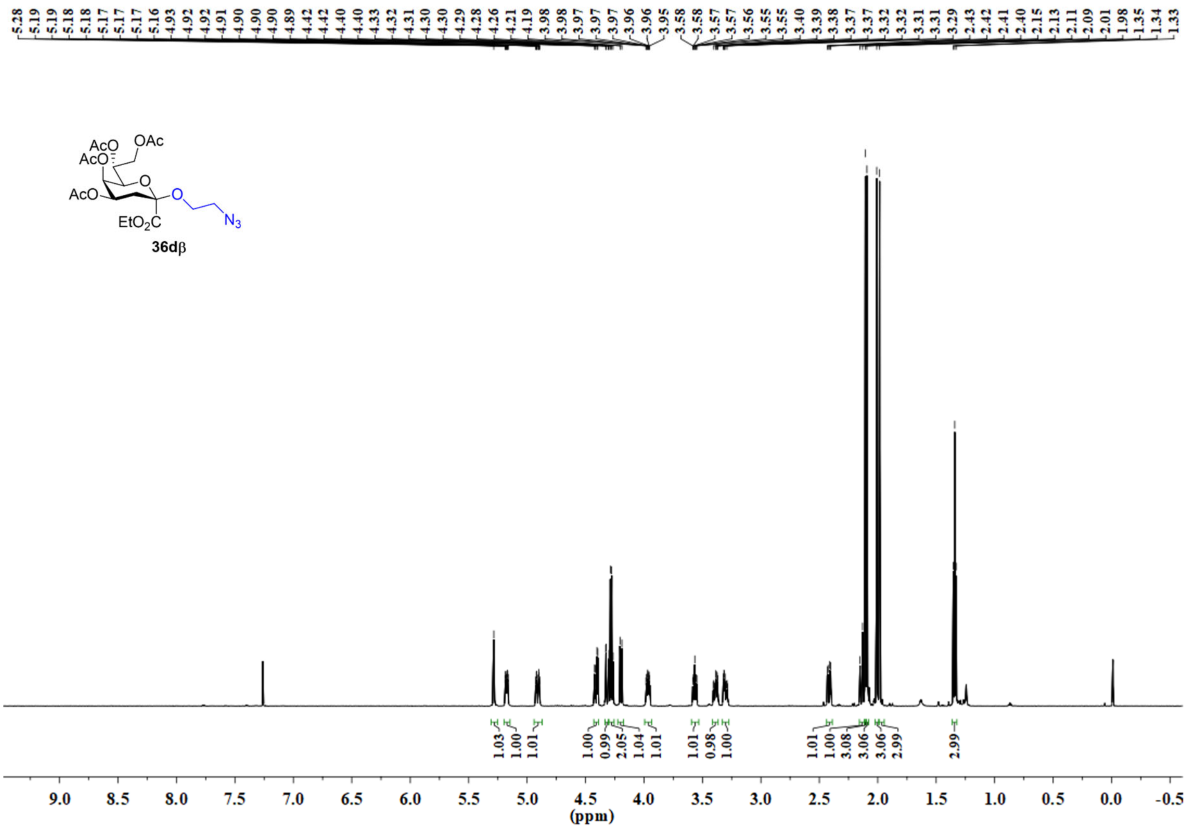

Fig. S33 ^{1}H NMR (600 MHz, $CDCl_3$) of **36dβ**

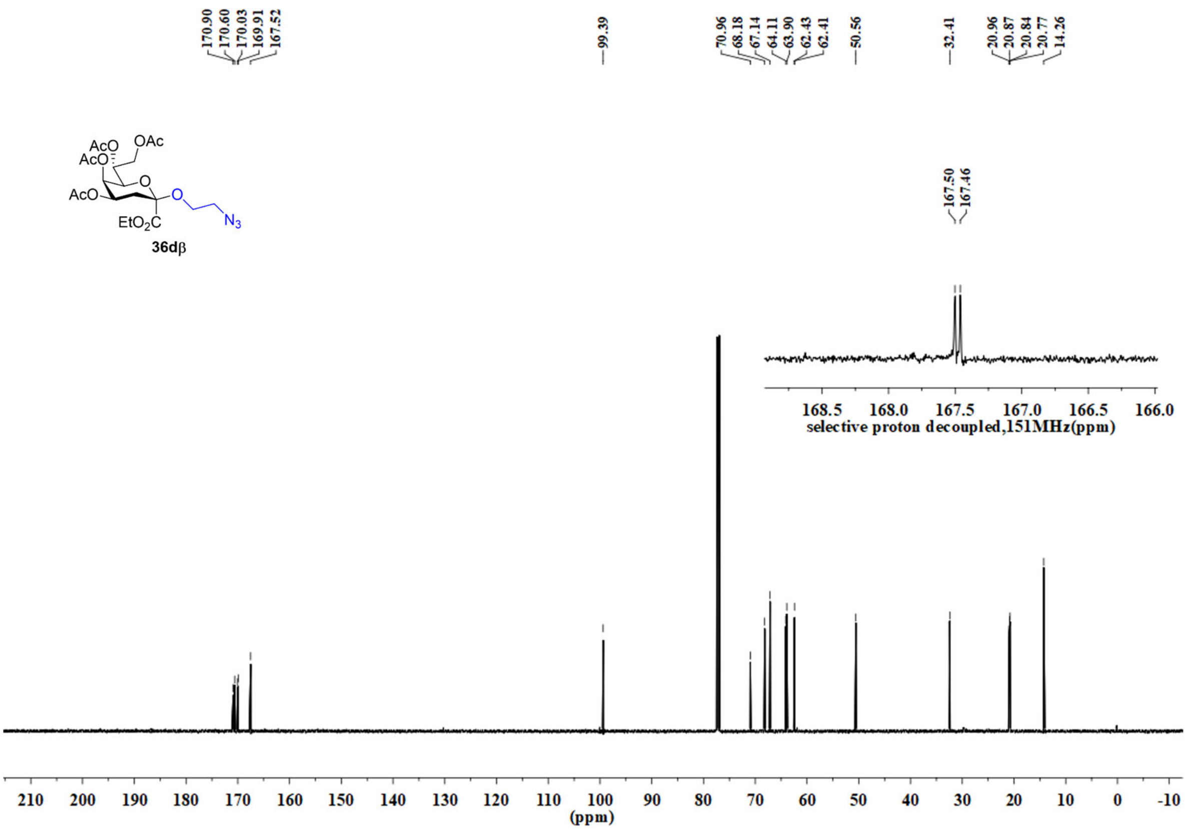

Fig. S34 ^{13}C NMR (151 MHz, $CDCl_3$) and

selective proton decoupled ^{13}C NMR (151 MHz, $CDCl_3$) of **36dβ**

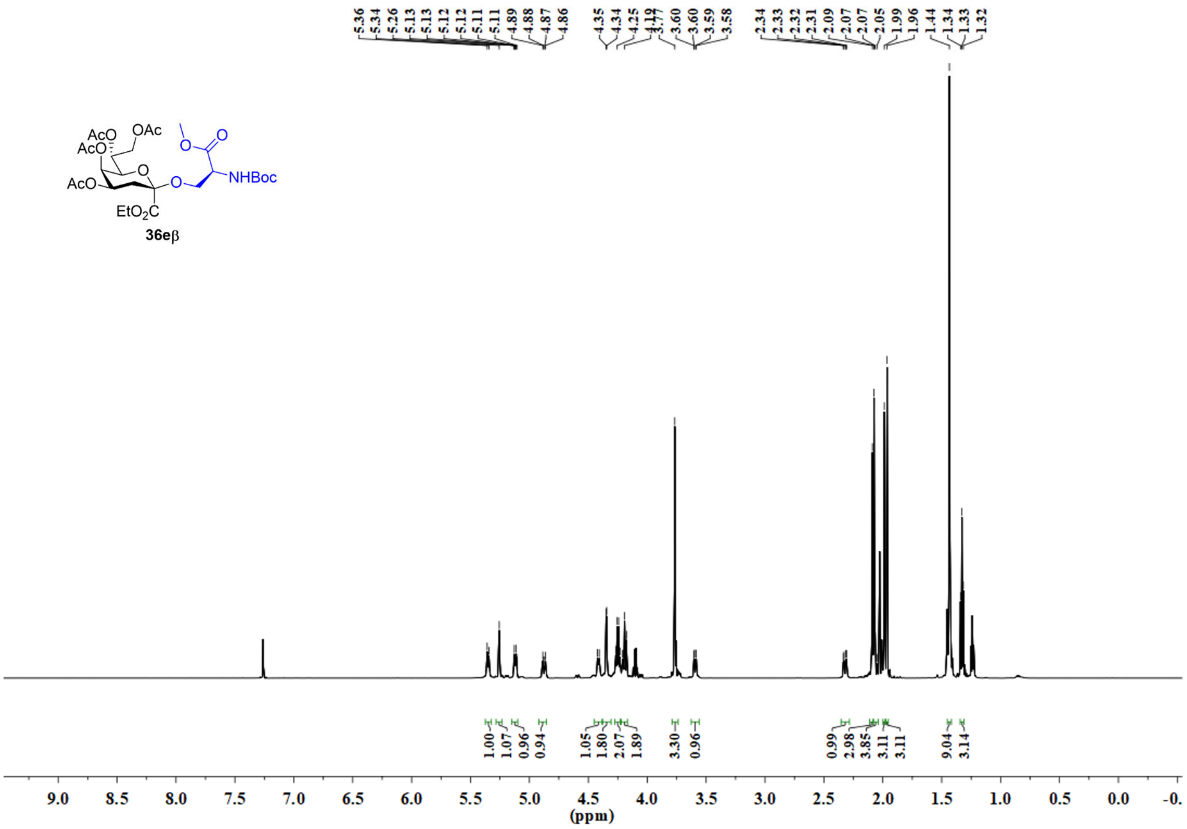

Fig. S35 ^{1}H NMR (600 MHz, $CDCl_3$) of **36eβ**

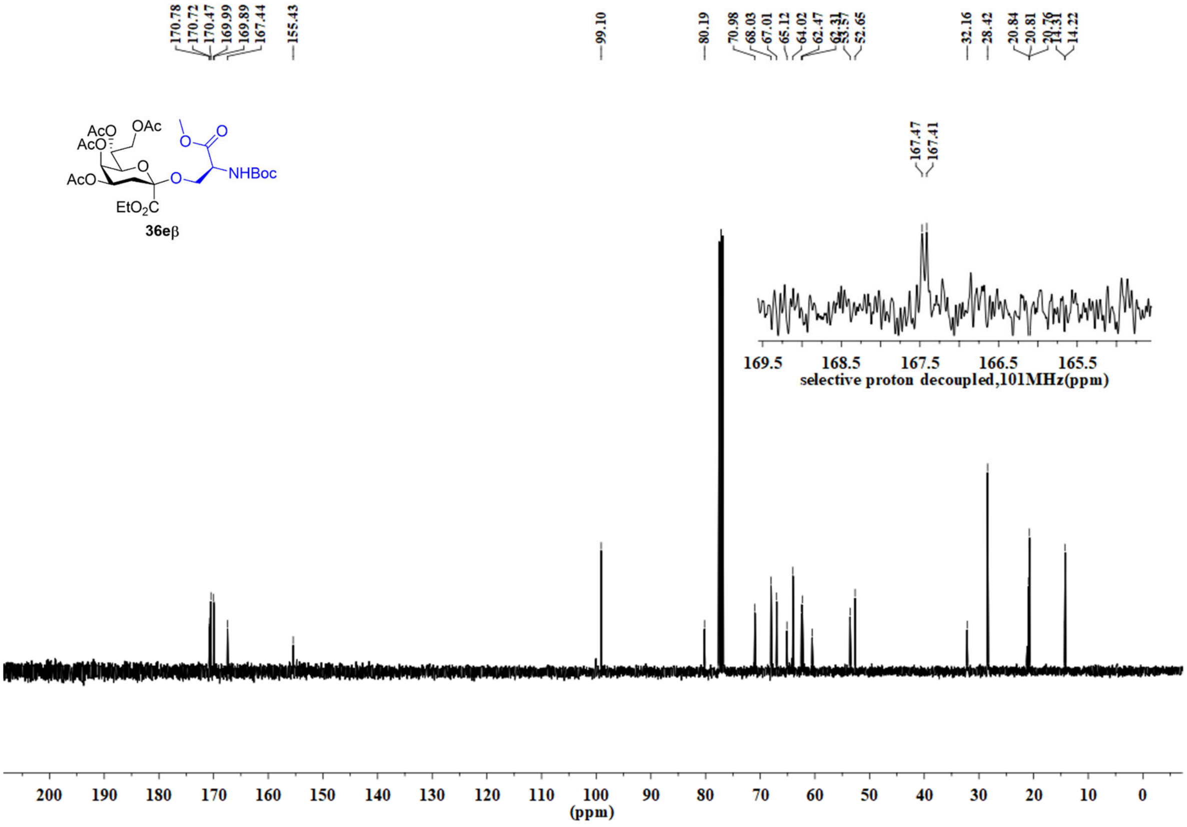

Fig. S36 ^{13}C NMR (101 MHz, $CDCl_3$) and

selective proton decoupled ^{13}C NMR (101 MHz, $CDCl_3$) of **36eβ**

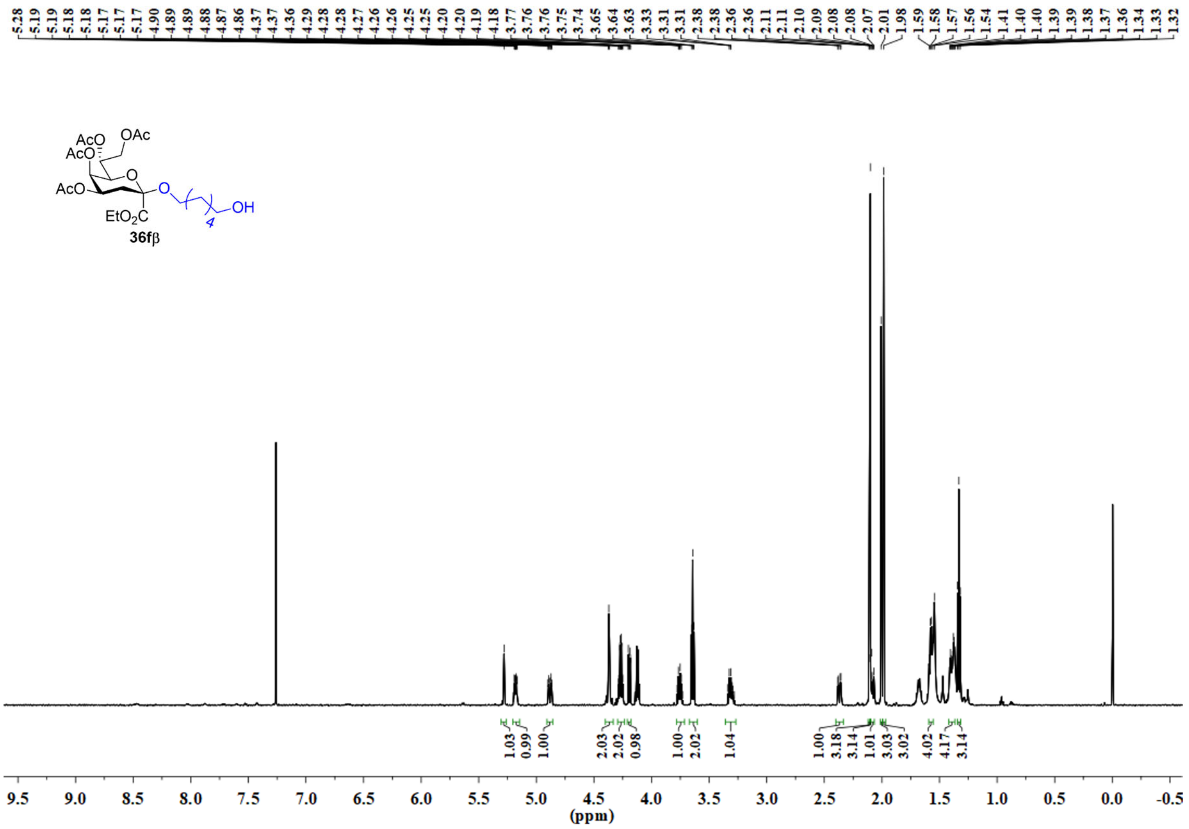

Fig. S37 ^{1}H NMR (600 MHz, $CDCl_3$) of **36fβ**

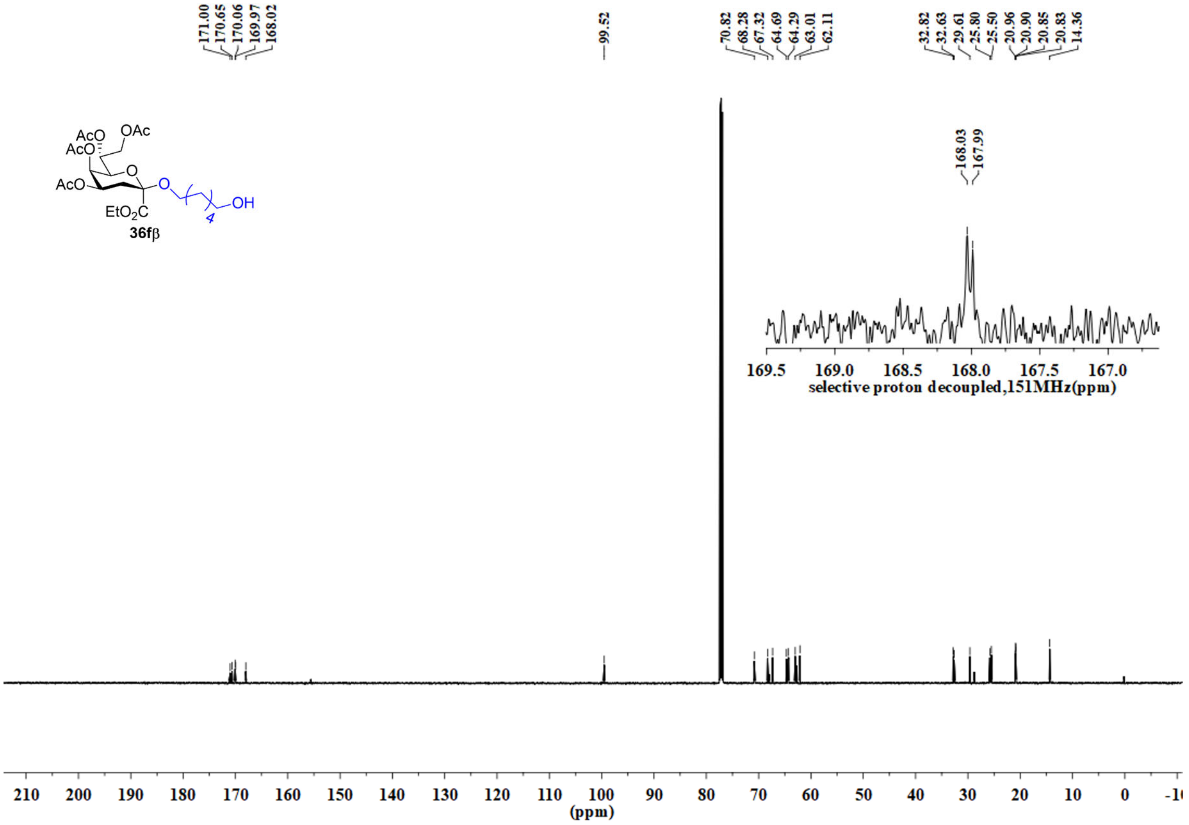

Fig. S38 ^{13}C NMR (151 MHz, $CDCl_3$) and

selective proton decoupled ^{13}C NMR (151 MHz, $CDCl_3$) of **36fβ**

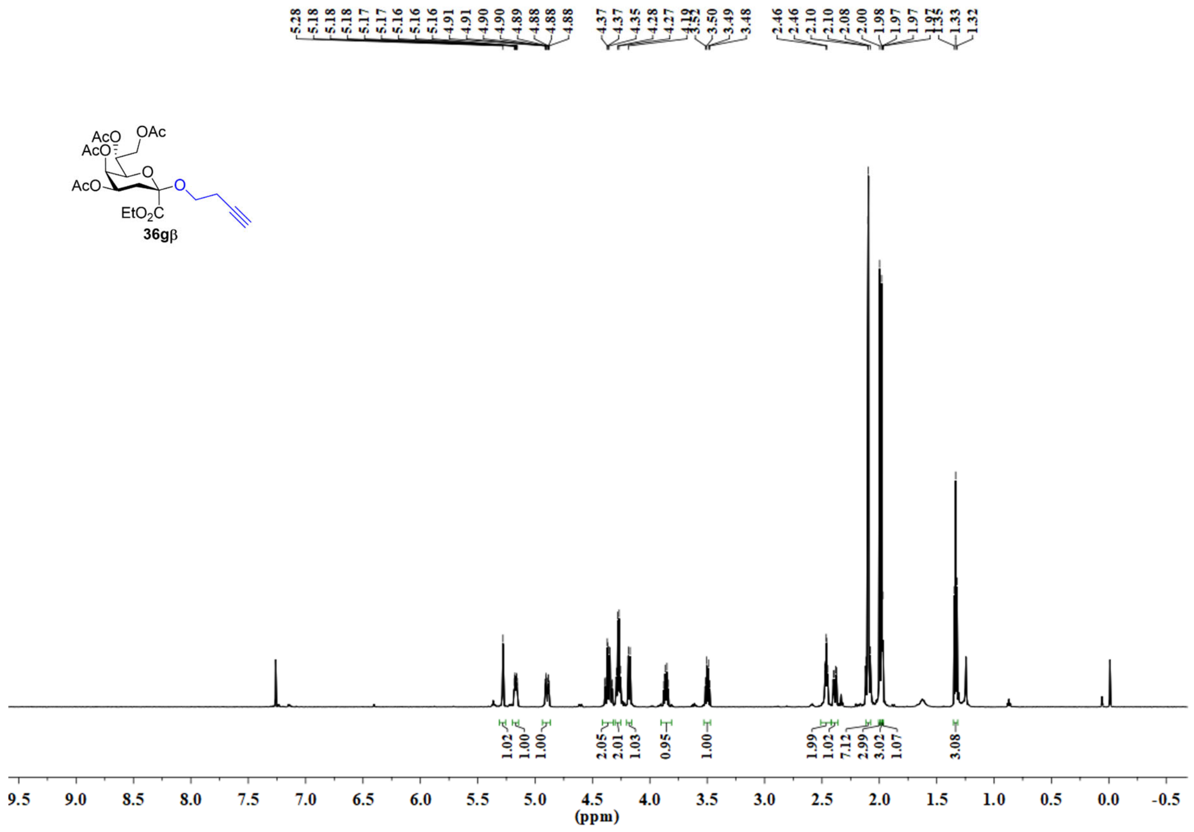

Fig. S39 ^{1}H NMR (600 MHz, $CDCl_3$) of **36gβ**

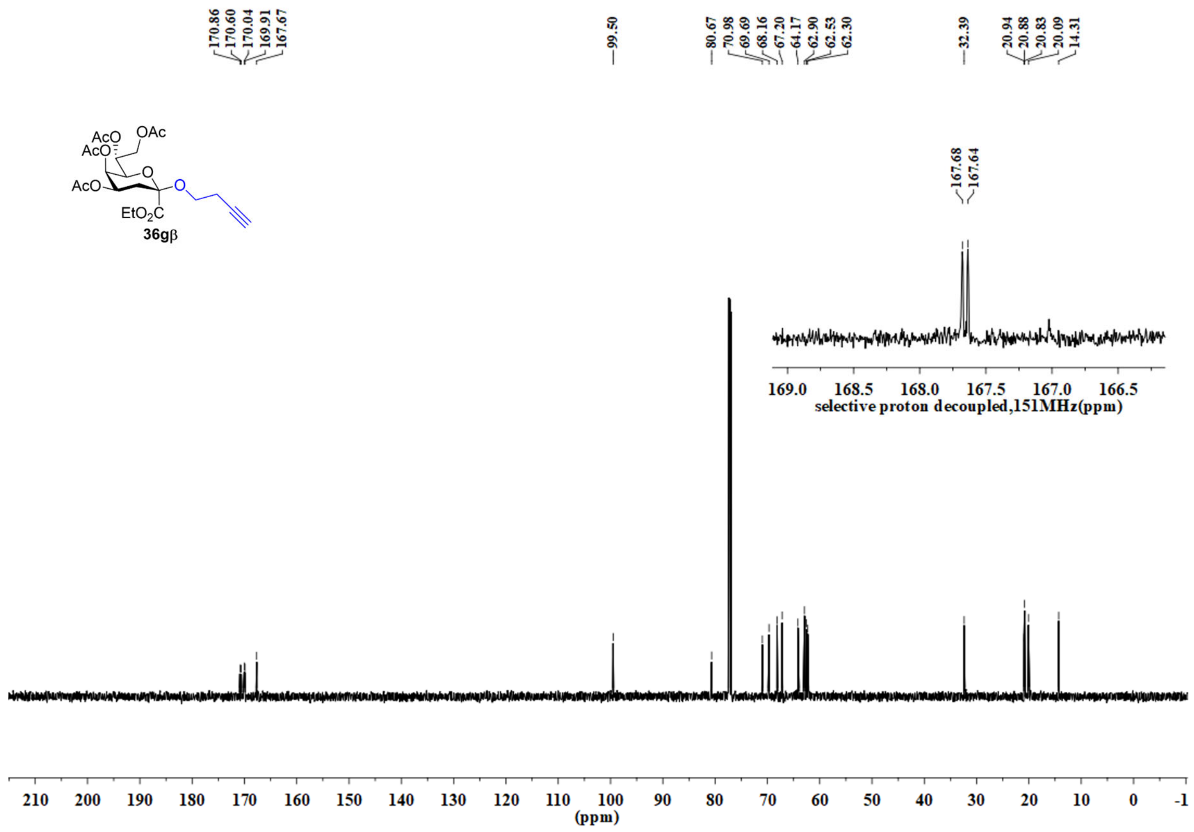

Fig. S40 ^{13}C NMR (151 MHz, $CDCl_3$) and

selective proton decoupled ^{13}C NMR (151 MHz, $CDCl_3$) of **36gβ**

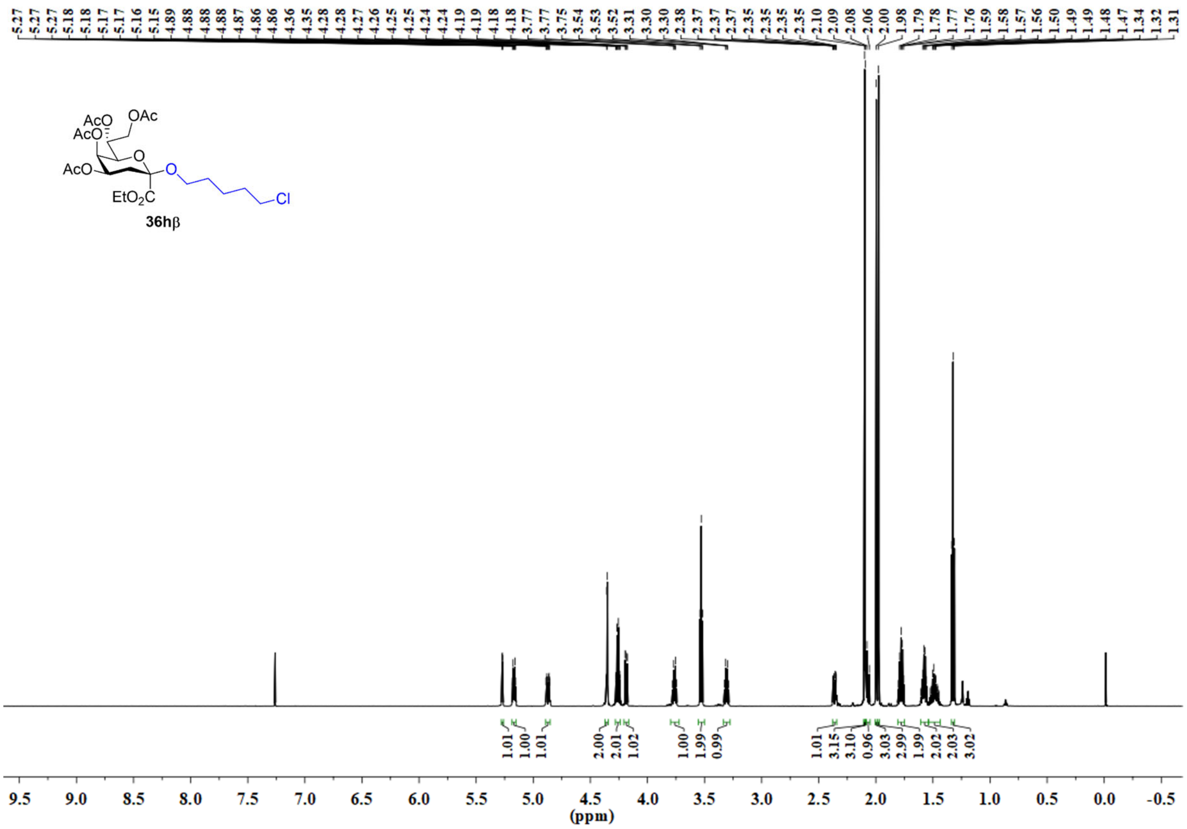

Fig. S41 ^{1}H NMR (600 MHz, $CDCl_3$) of **36hβ**

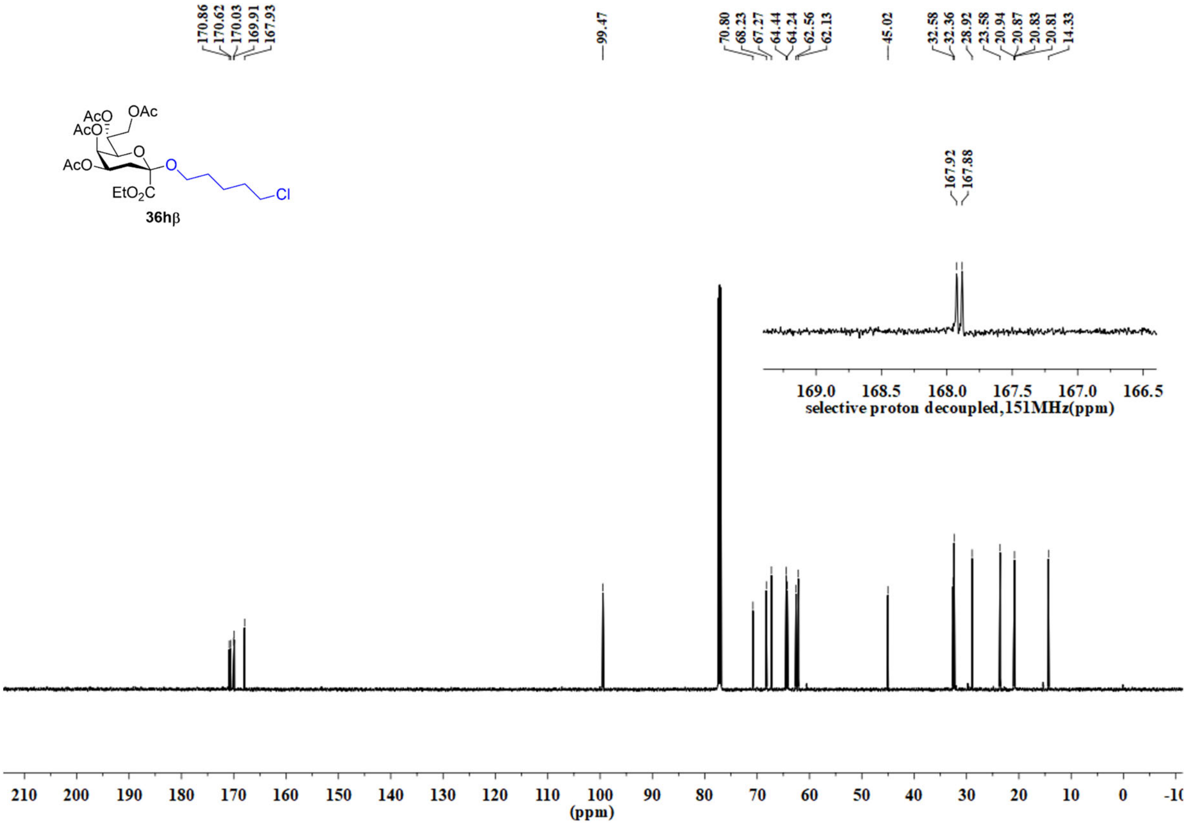

Fig. S42 ^{13}C NMR (151 MHz, $CDCl_3$) and

selective proton decoupled ^{13}C NMR (151 MHz, $CDCl_3$) of **36hβ**

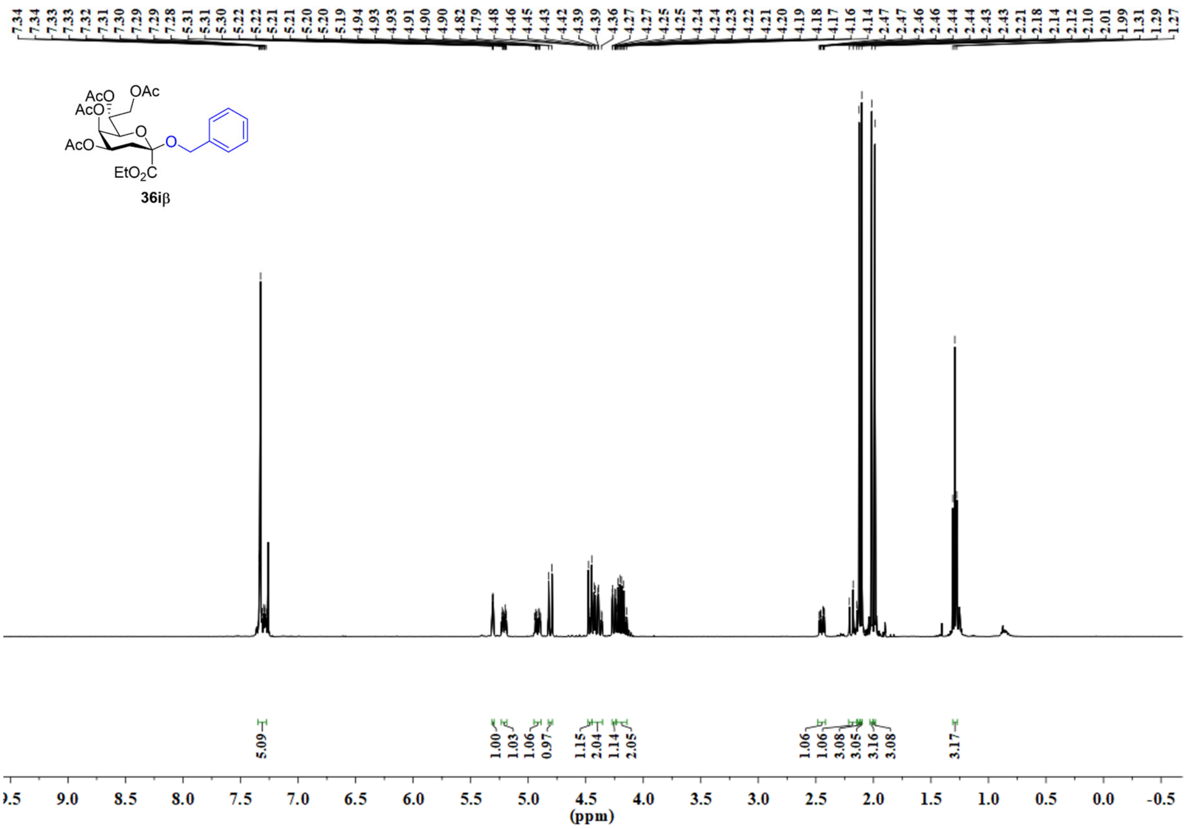

Fig. S43 ^{1}H NMR (400 MHz, $CDCl_3$) of **36iβ**

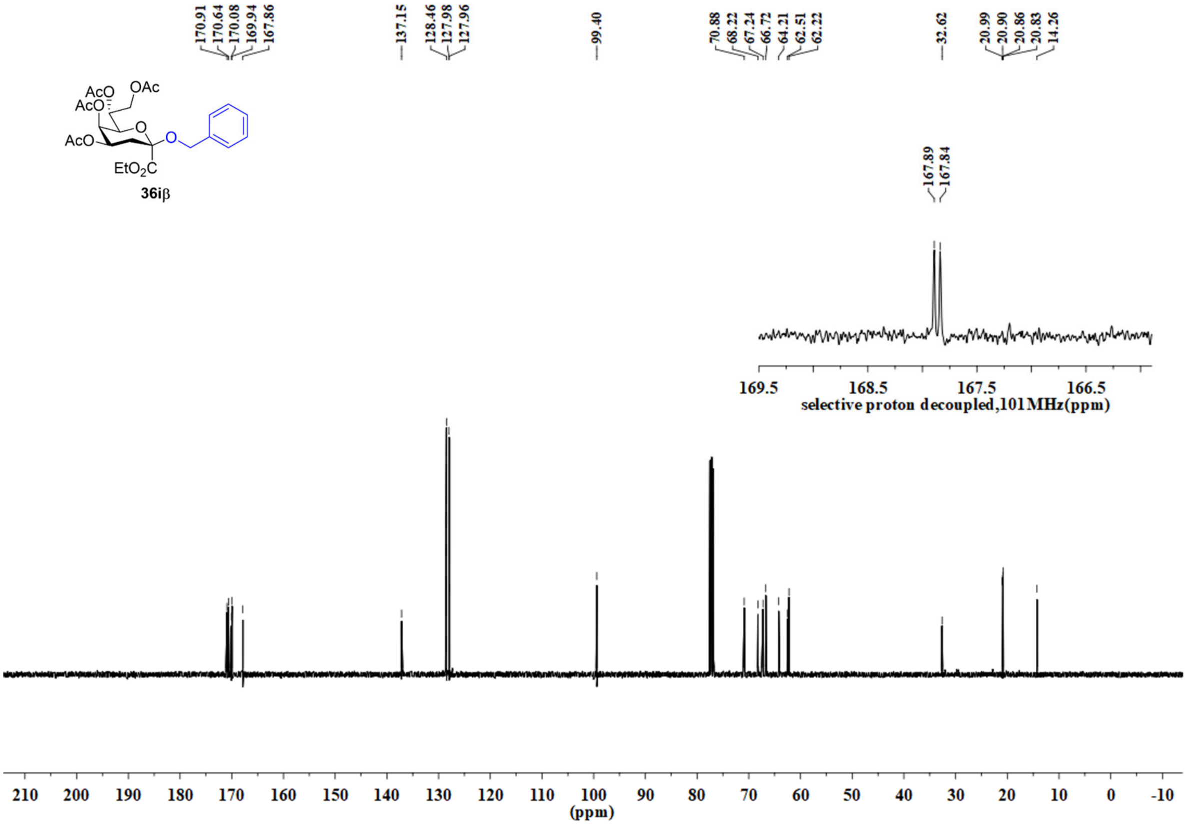

Fig. S44 ^{13}C NMR (101 MHz, $CDCl_3$) and

selective proton decoupled ^{13}C NMR (101 MHz, $CDCl_3$) of **36iβ**

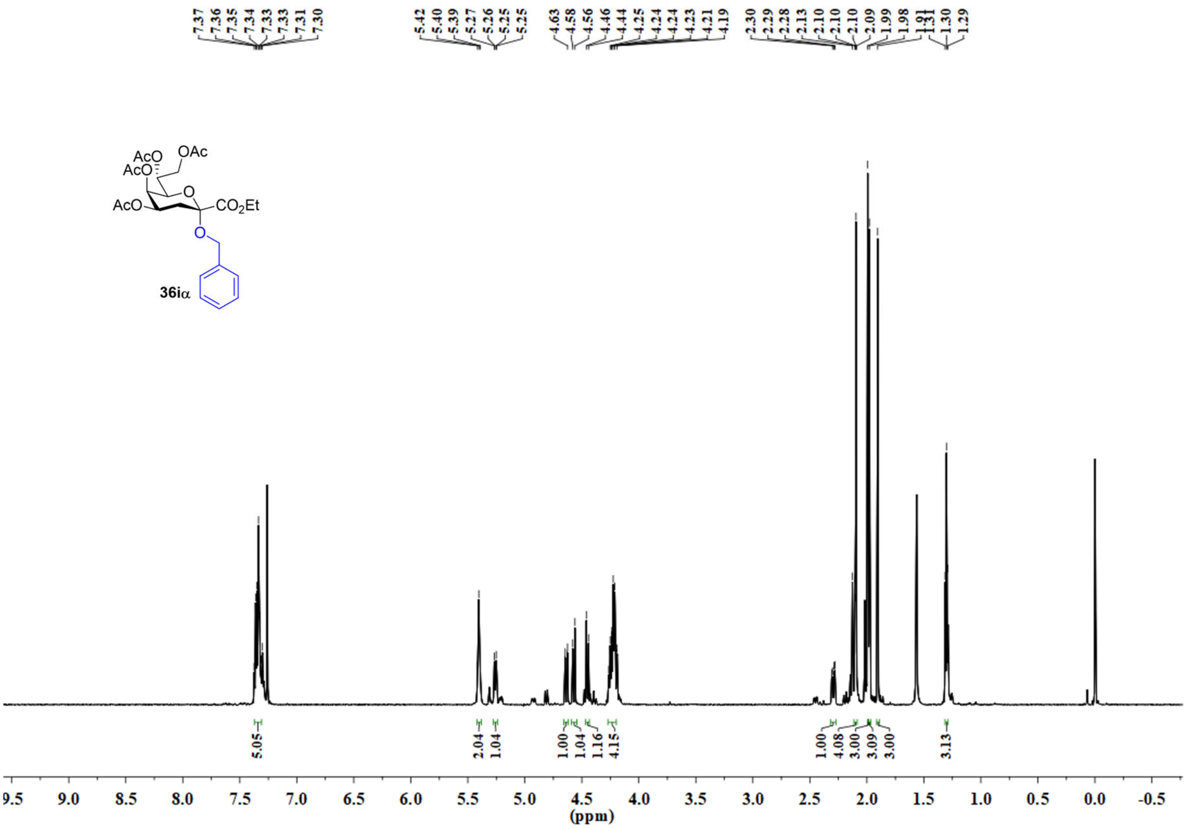

Fig. S45 ^{1}H NMR (600 MHz, CDCl$_3$) of **36iα**

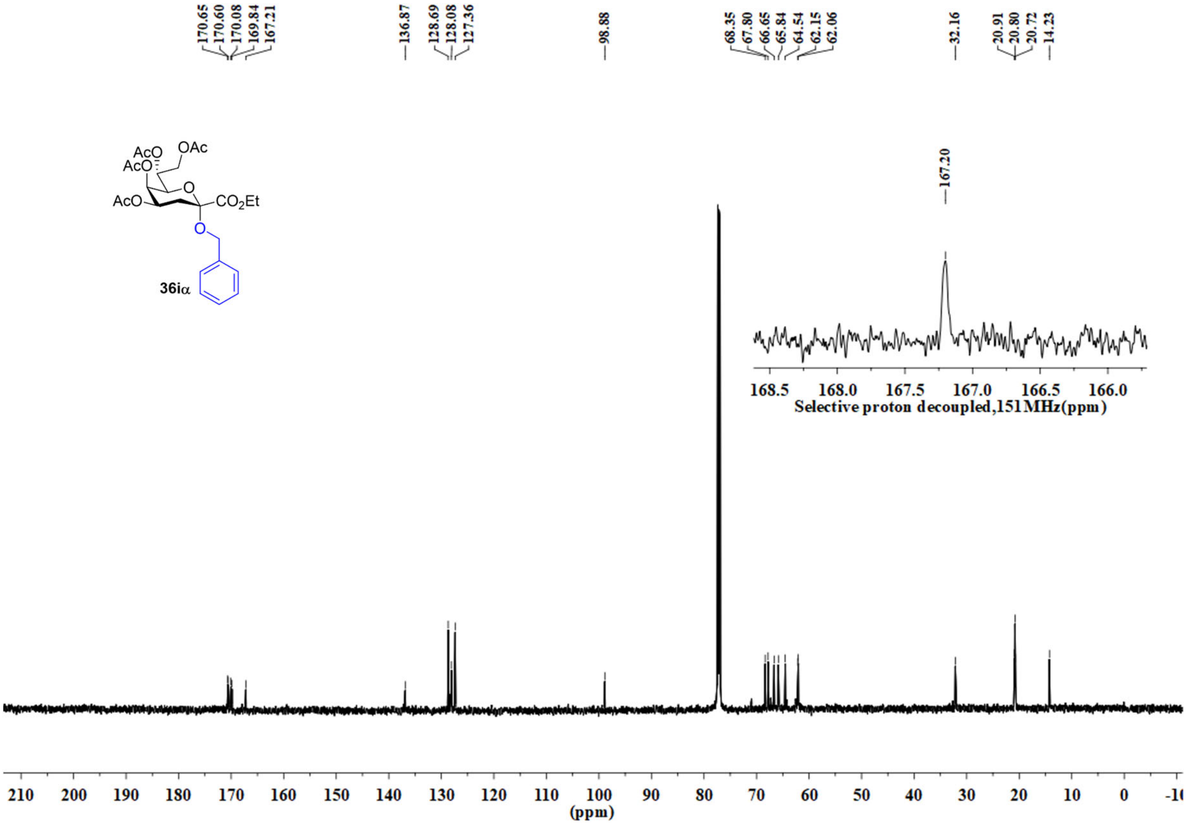

Fig. S46 ^{13}C NMR (151 MHz, CDCl$_3$) and

selective proton decoupled ^{13}C NMR (151 MHz, CDCl$_3$) of **36iα**

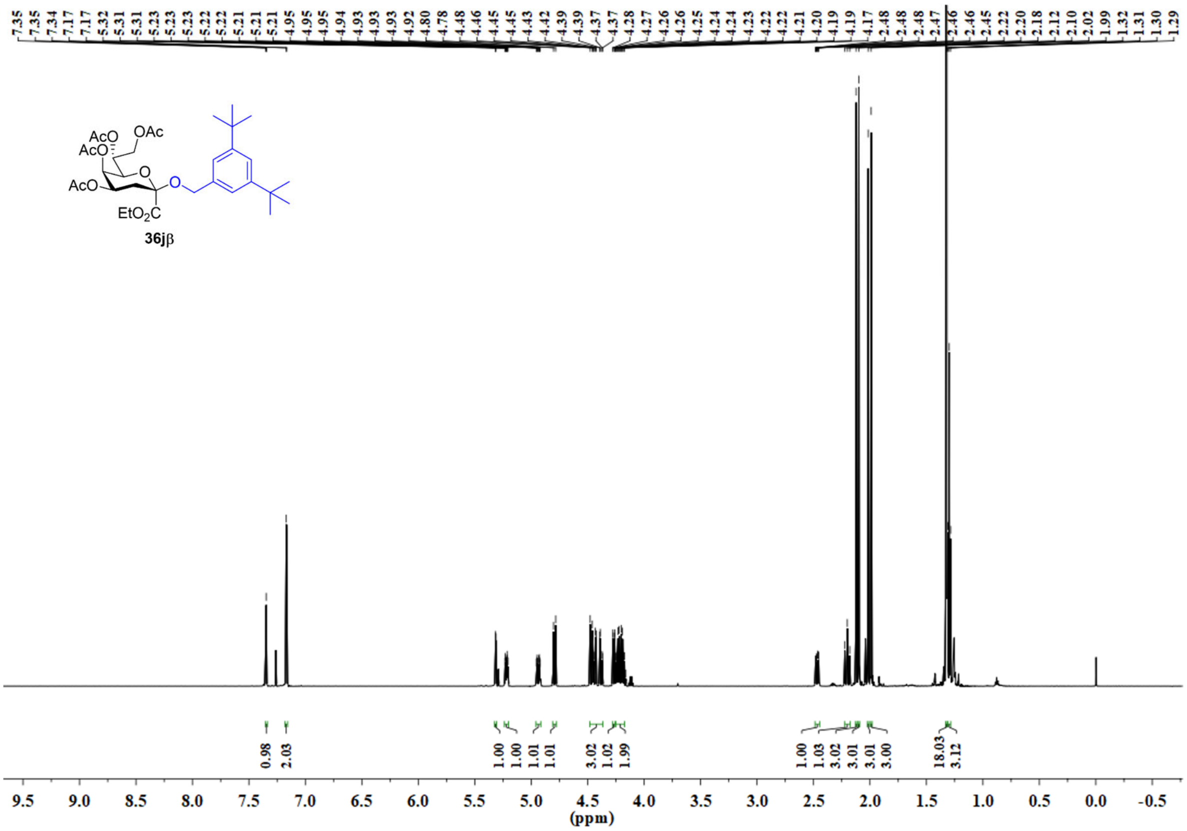

Fig. S47 ^{1}H NMR (600 MHz, $CDCl_3$) of **36jβ**

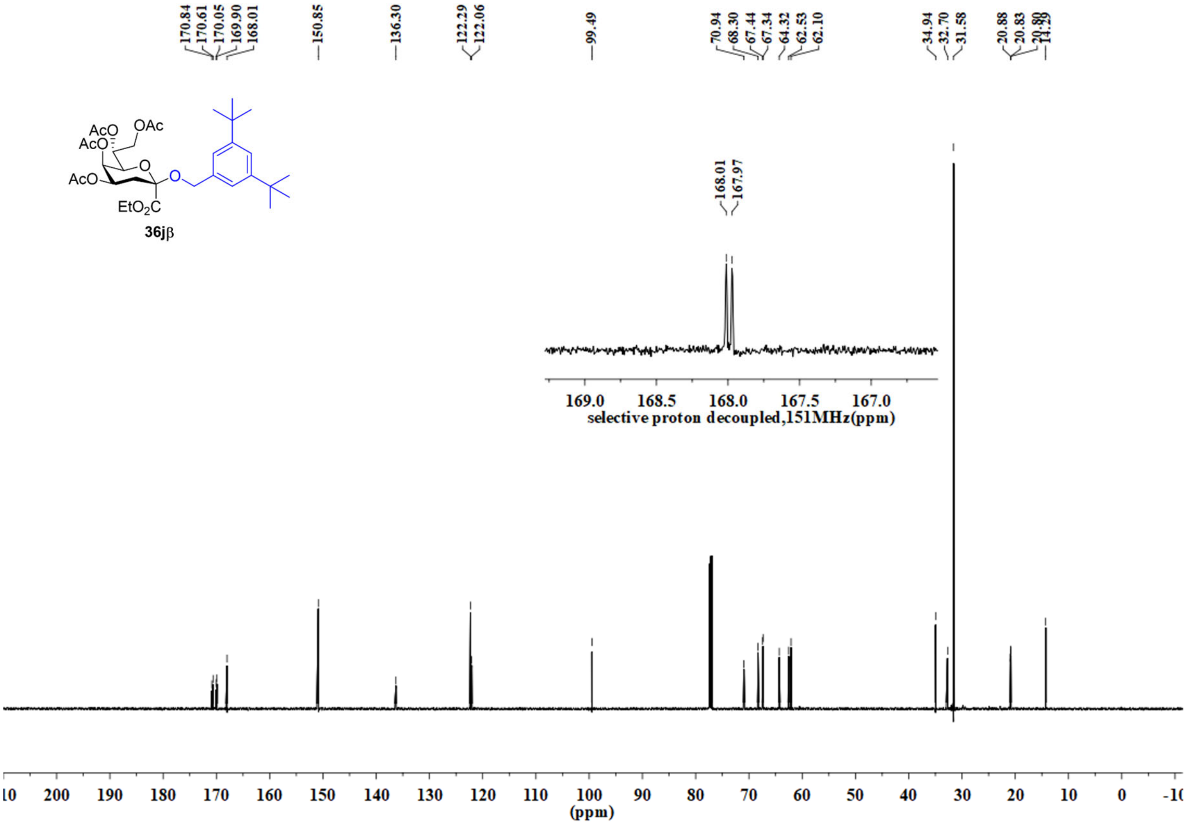

Fig. S48 ^{13}C NMR (151 MHz, $CDCl_3$) and

selective proton decoupled ^{13}C NMR (151 MHz, $CDCl_3$) of **36jβ**

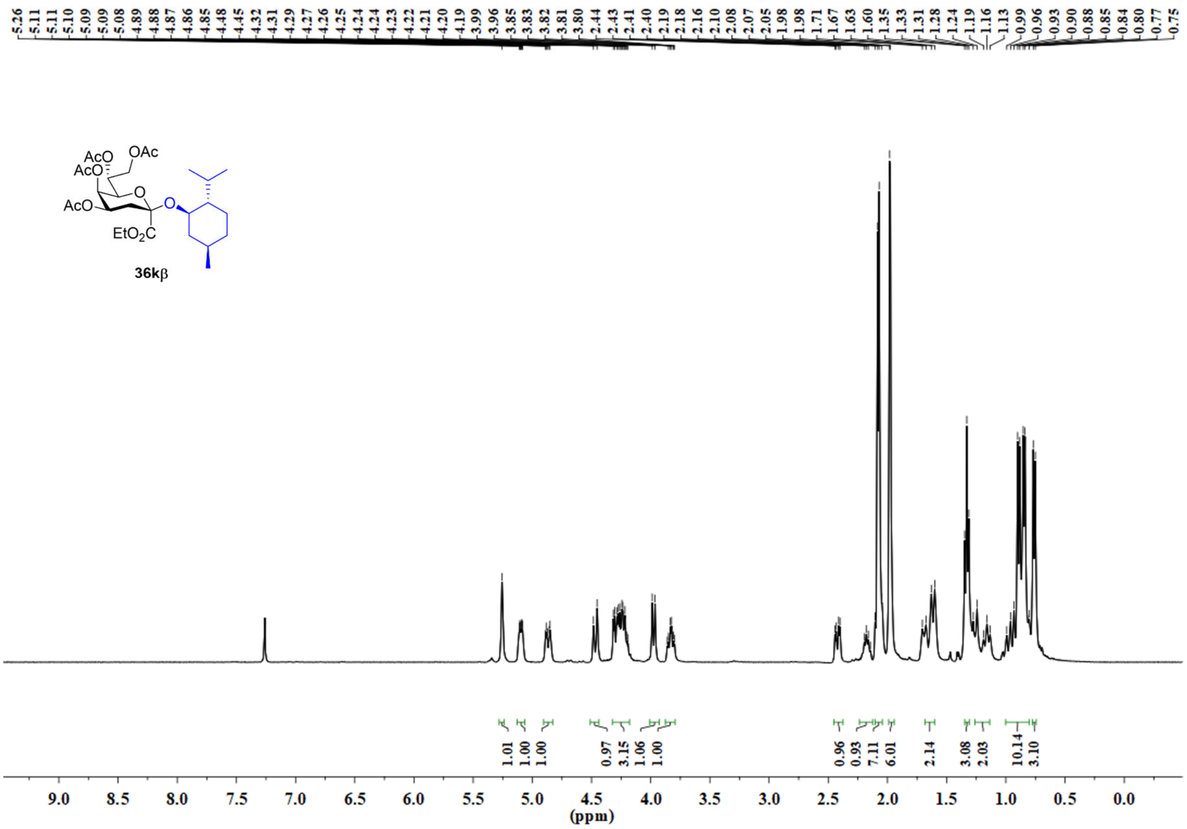

Fig. S49 ^{1}H NMR (400 MHz, CDCl3) of **36kβ**

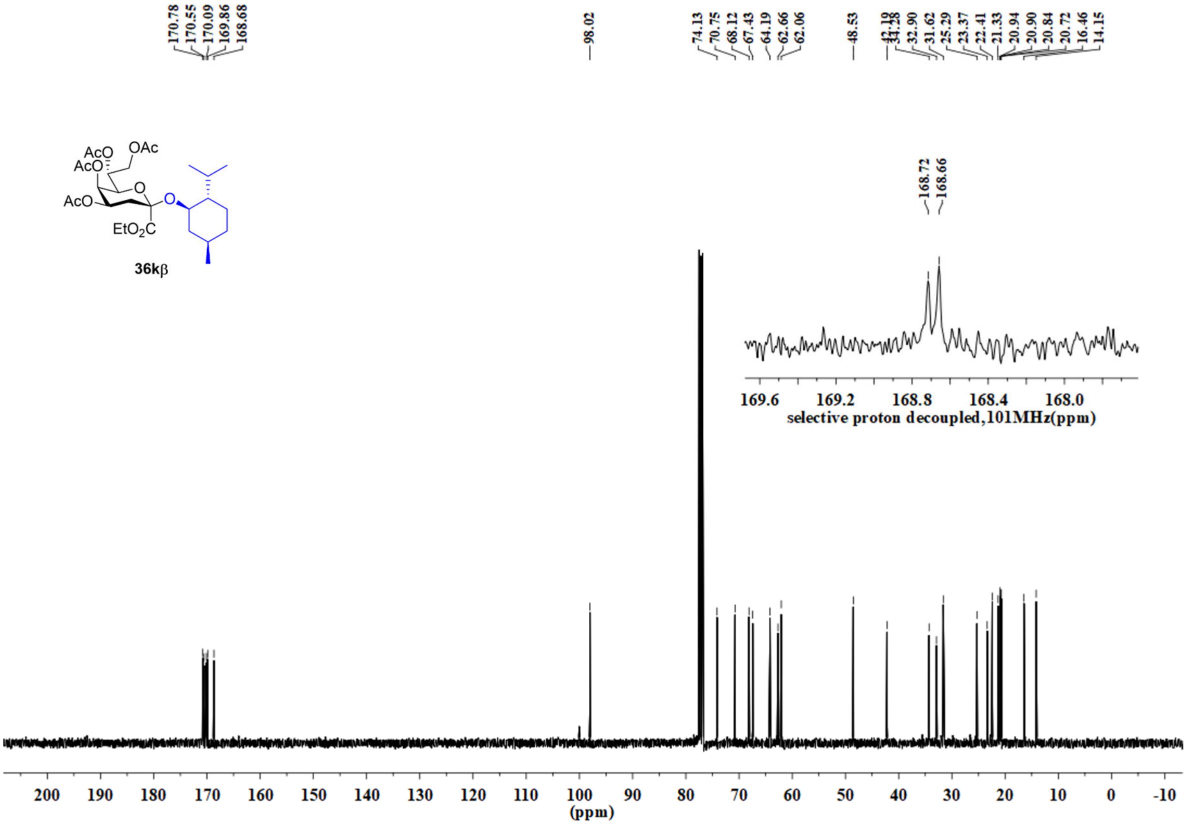

Fig. S50 ^{13}C NMR (101 MHz, CDCl3) and

selective proton decoupled ^{13}C NMR (101 MHz, CDCl3) of **36kβ**

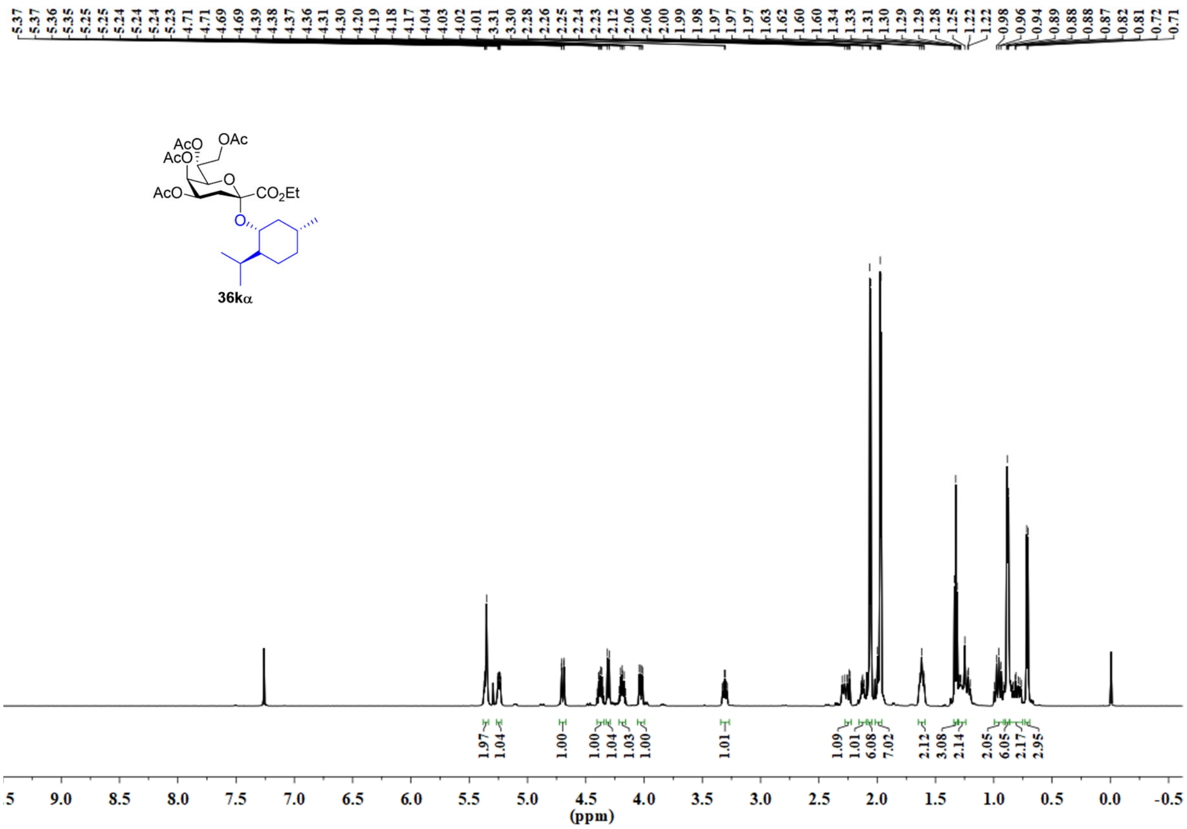

Fig. S51 ^{1}H NMR (600 MHz, $CDCl_3$) of **36kα**

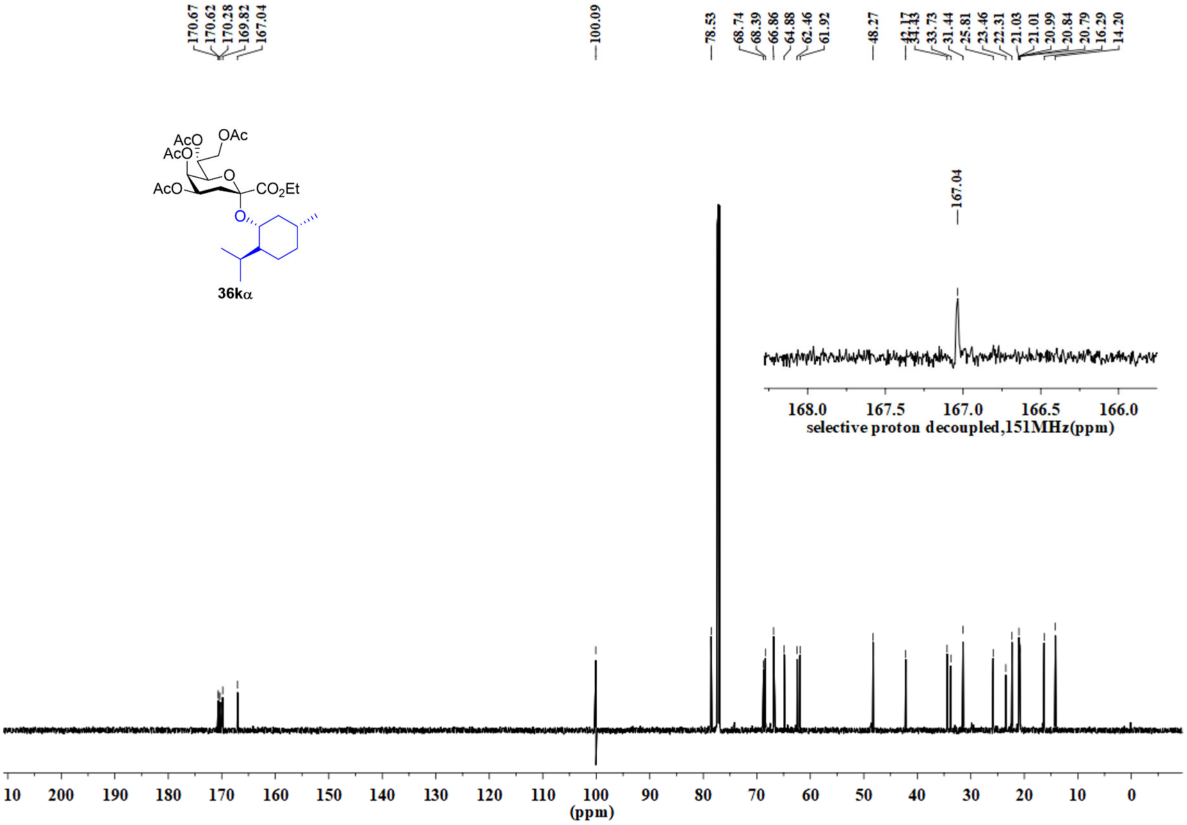

Fig. S52 ^{13}C NMR (151 MHz, $CDCl_3$) and

selective proton decoupled ^{13}C NMR (151 MHz, $CDCl_3$) of **36kα**

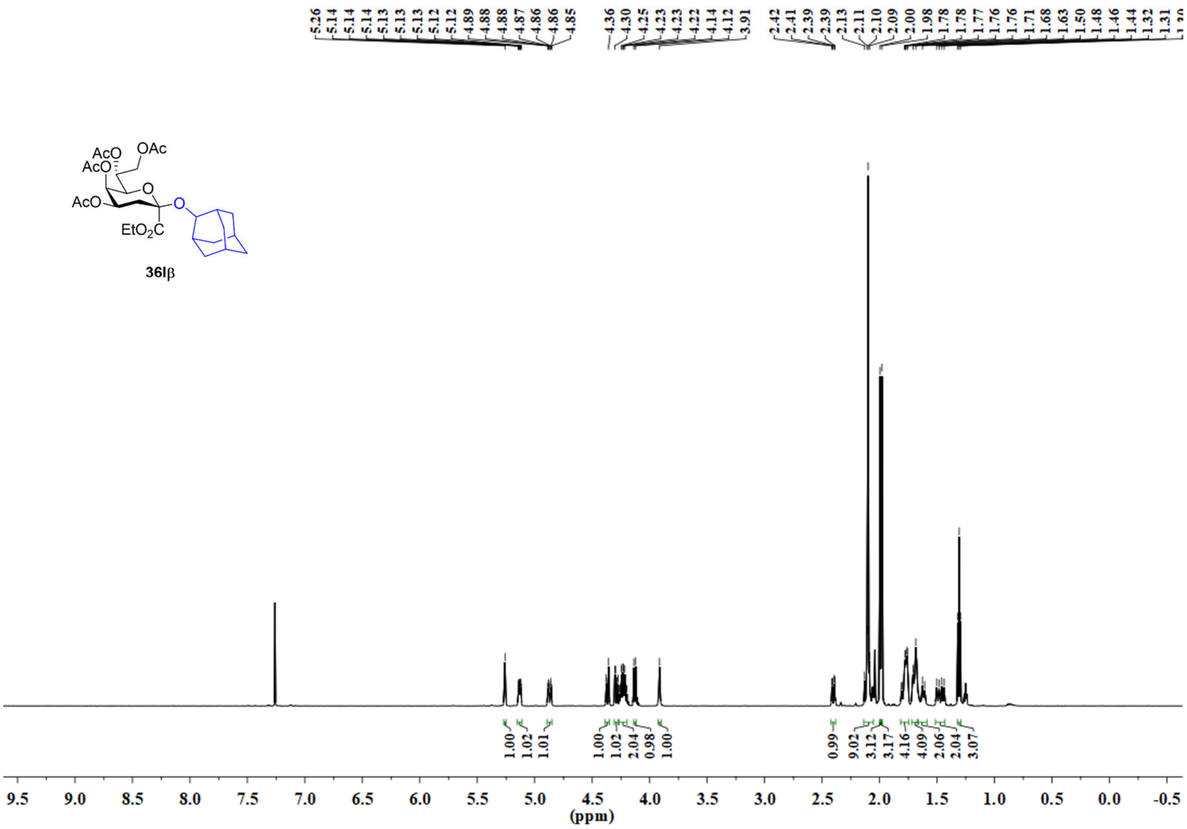

Fig. S53 ^{1}H NMR (600 MHz, CDCl3) of **36lβ**

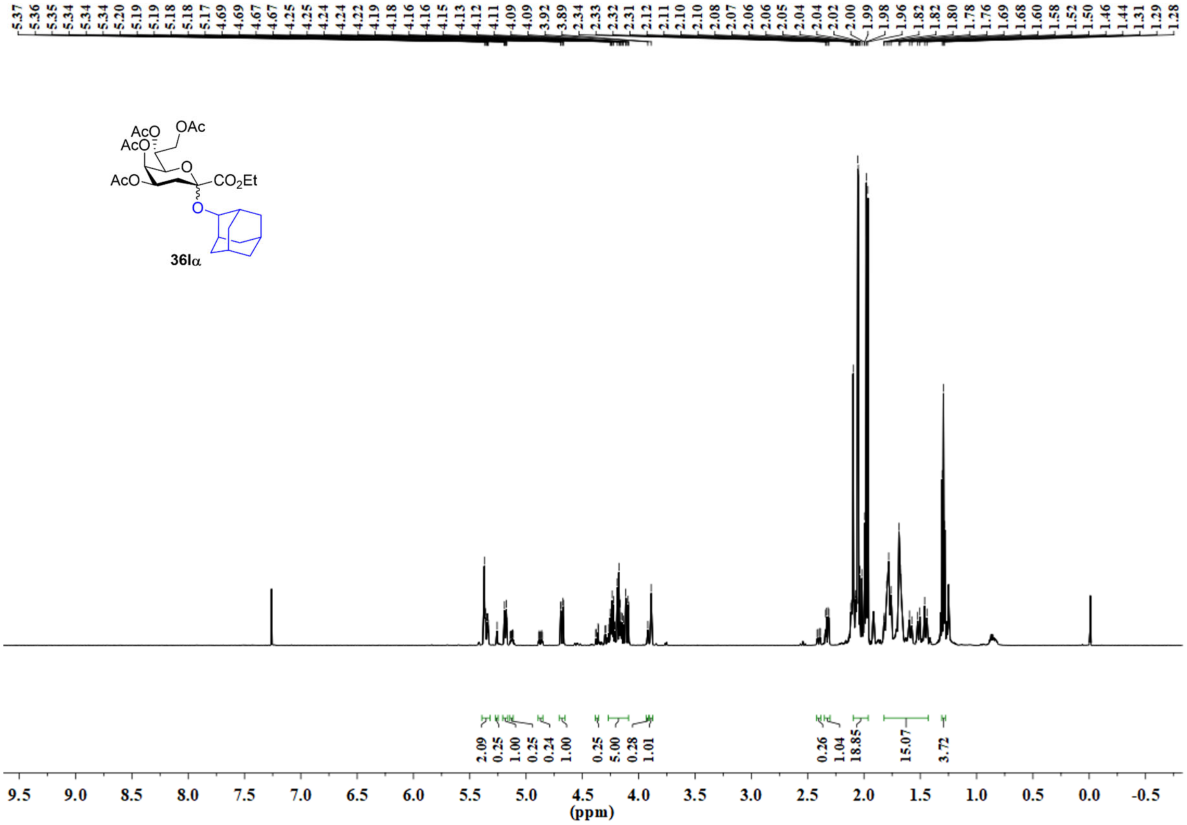

Fig. S54 ^{1}H NMR (600 MHz, CDCl3) of **36lα**

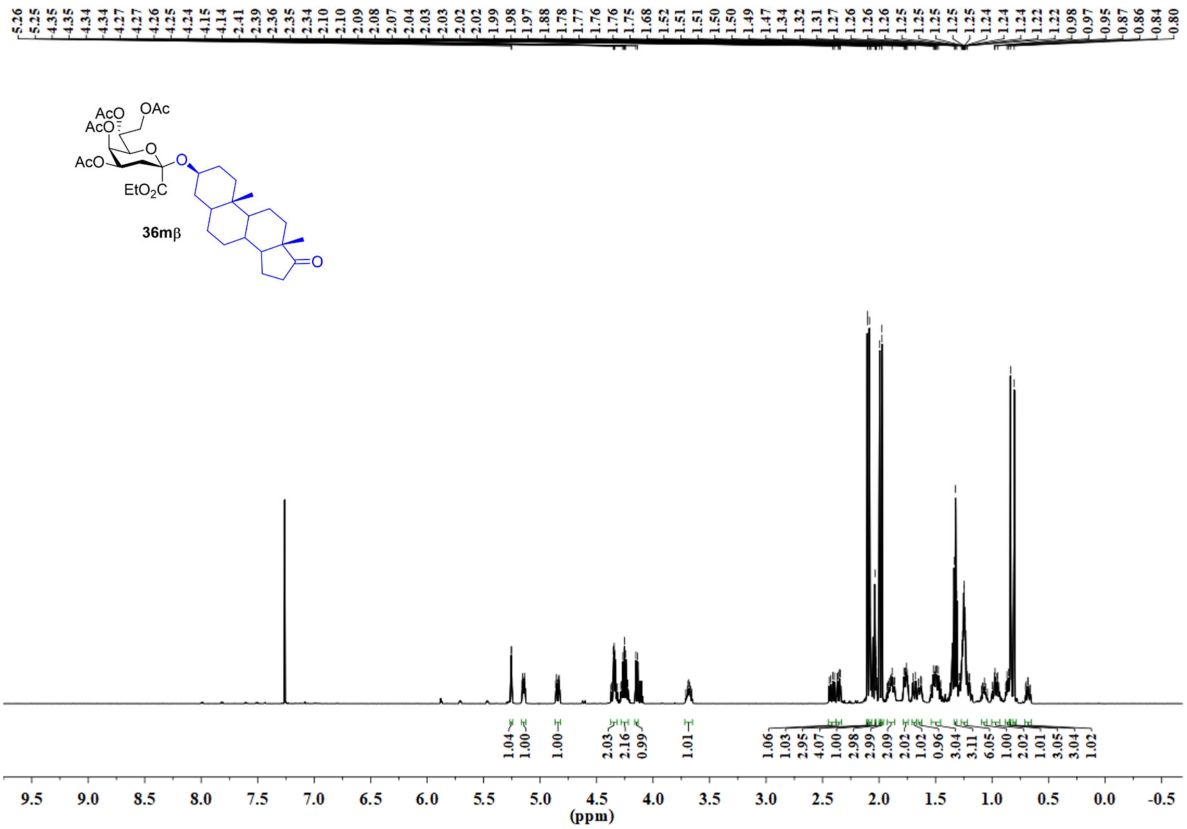

Fig. S55 ^{1}H NMR (600 MHz, $CDCl_3$) of **36mβ**

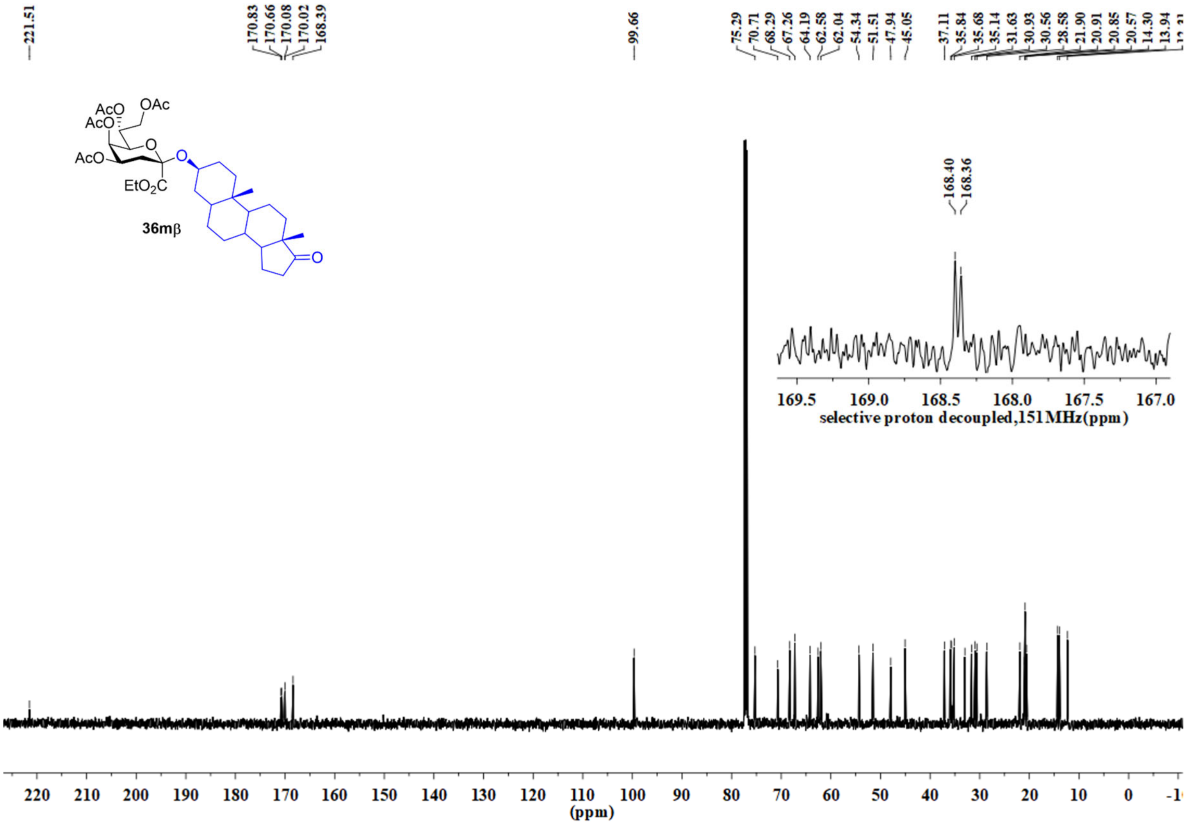

Fig. S56 ^{13}C NMR (151 MHz, $CDCl_3$) and

selective proton decoupled ^{13}C NMR (151 MHz, $CDCl_3$) of **36mβ**

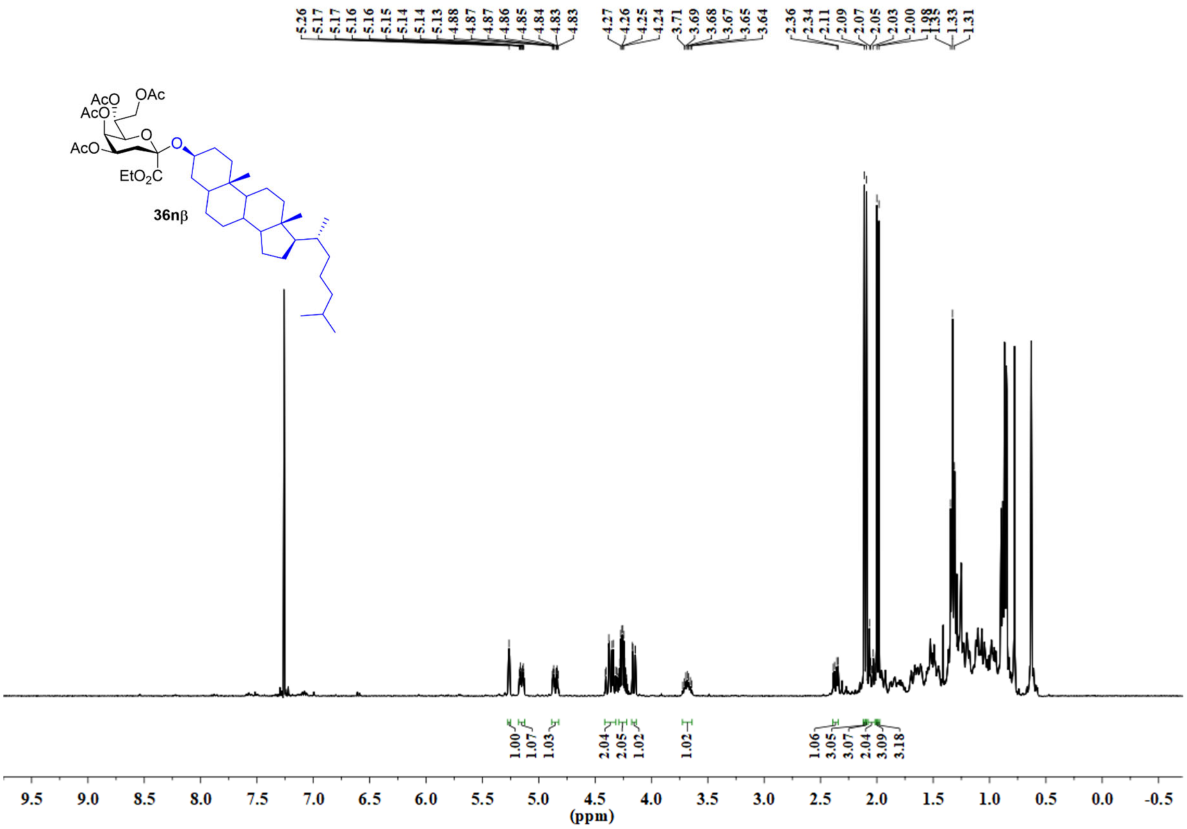

Fig. S57 ^{1}H NMR (400 MHz, $CDCl_3$) of **36nβ**

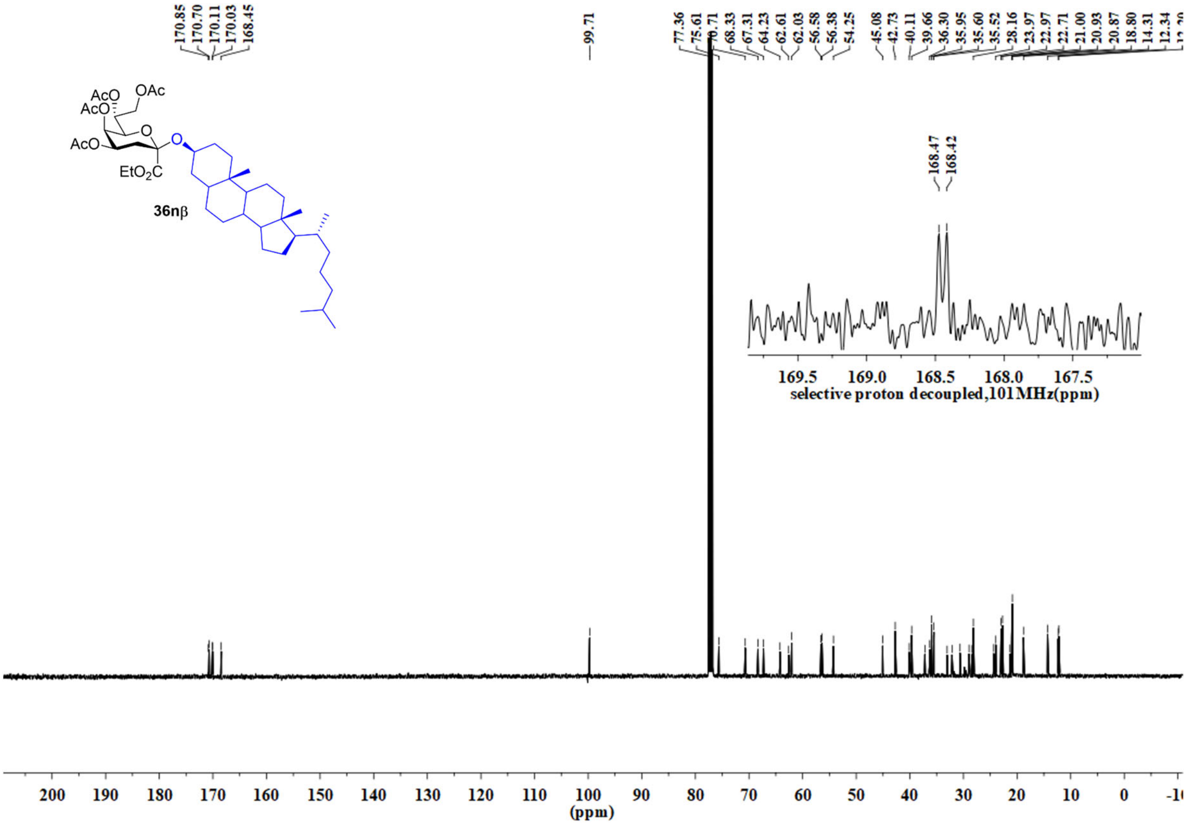

Fig. S58 ^{13}C NMR (101 MHz, $CDCl_3$) and

selective proton decoupled ^{13}C NMR (101 MHz, $CDCl_3$) of **36nβ**

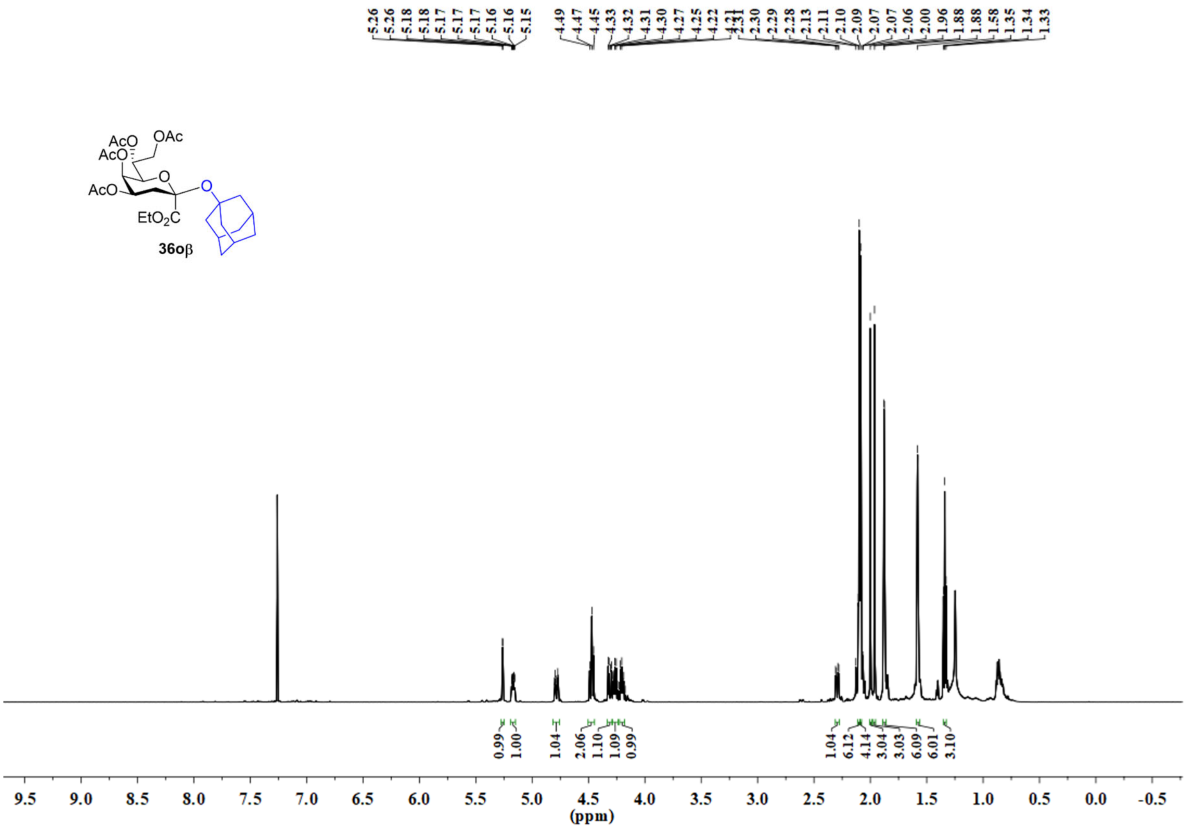

Fig. S59 ^{1}H NMR (600 MHz, $CDCl_3$) of **36oβ**

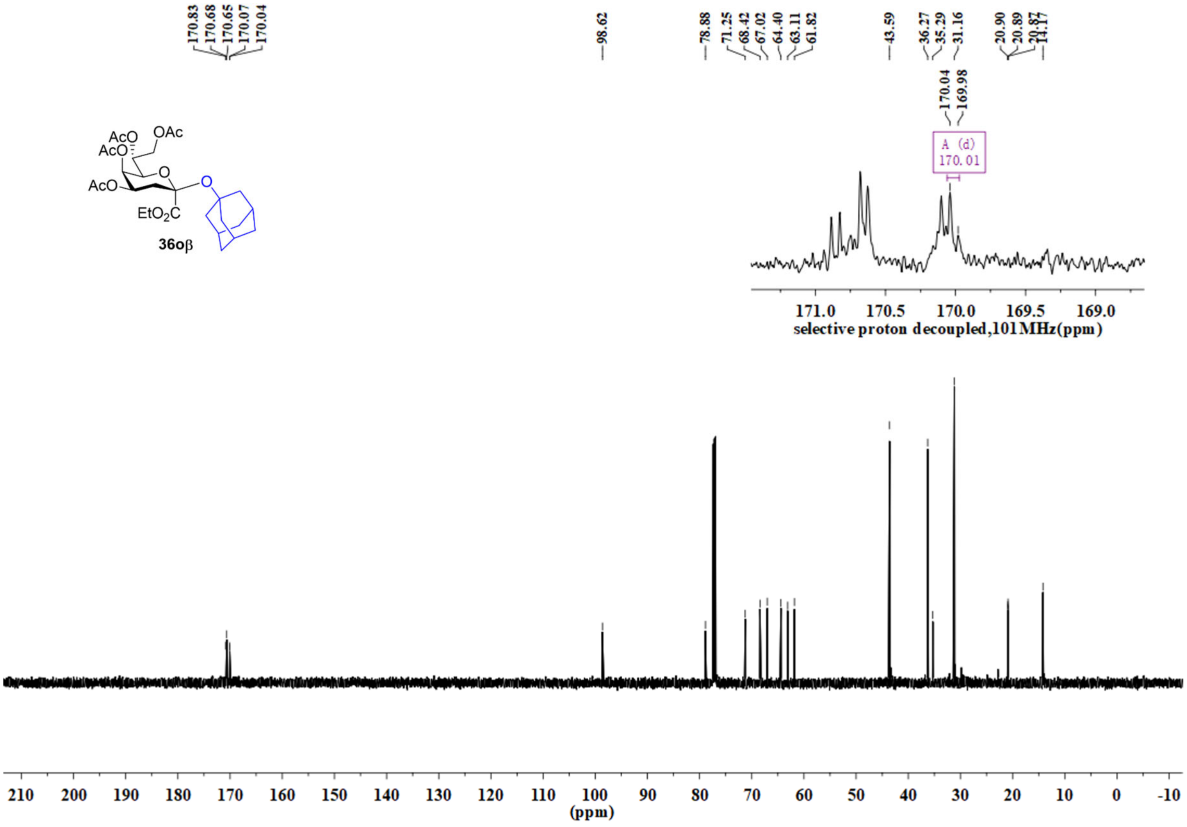

Fig. S60 ^{13}C NMR (151 MHz, $CDCl_3$) and

selective proton decoupled ^{13}C NMR (151 MHz, $CDCl_3$) of **36oβ**

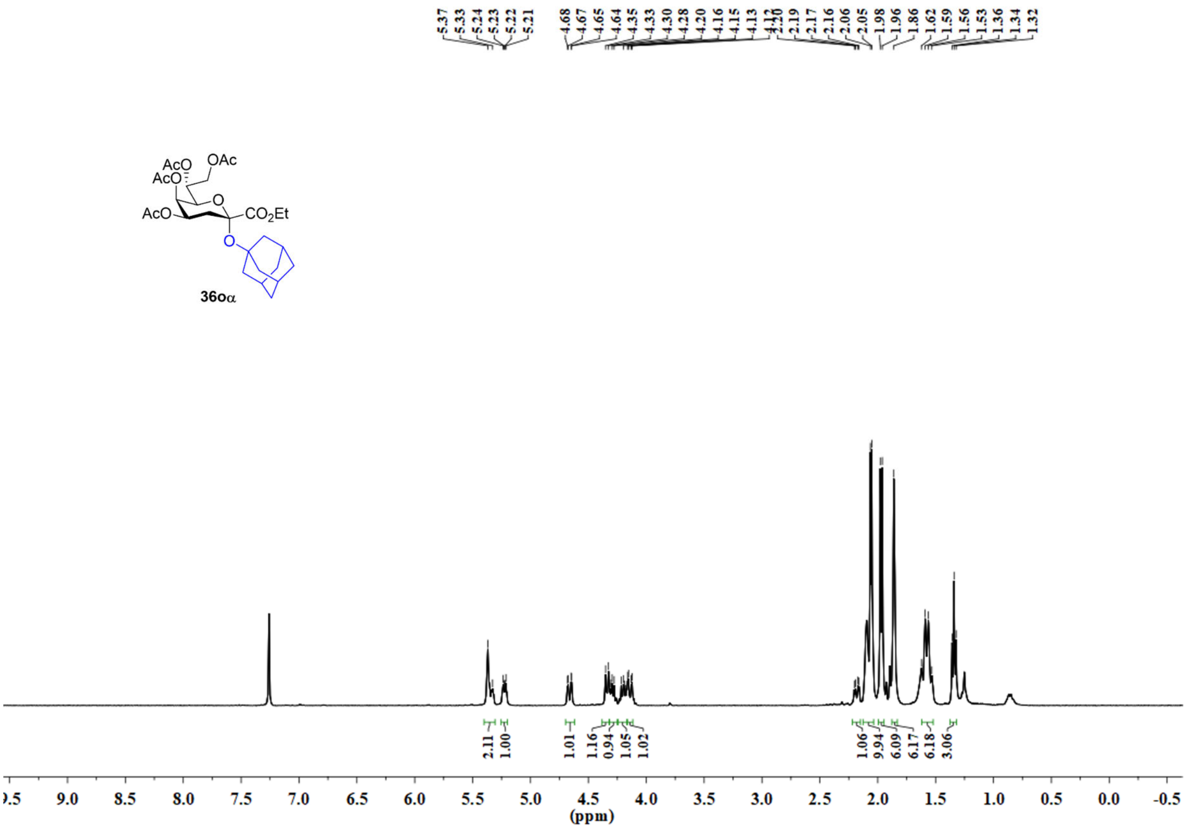

Fig. S61 ^{1}H NMR (400 MHz, $CDCl_3$) of **36oα**

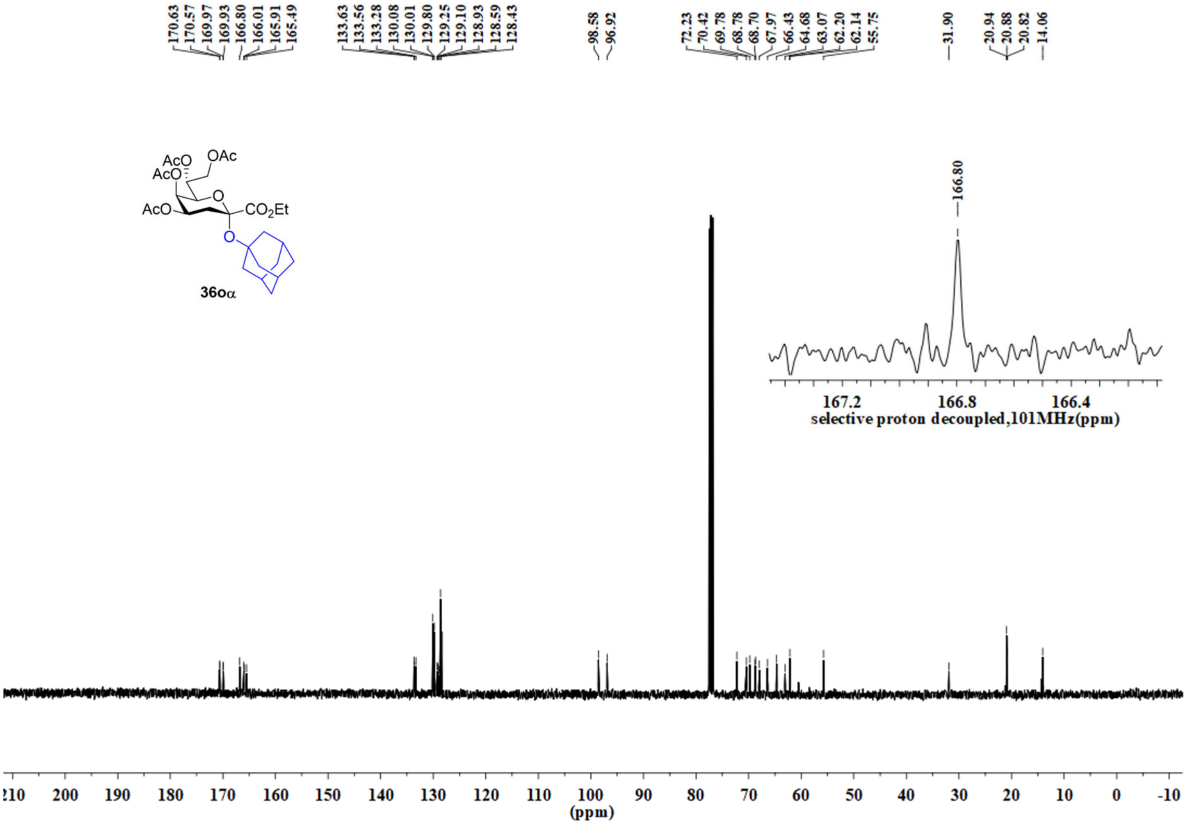

Fig. S62 ^{13}C NMR (101 MHz, $CDCl_3$) and

selective proton decoupled ^{13}C NMR (101 MHz, $CDCl_3$) of **36oα**

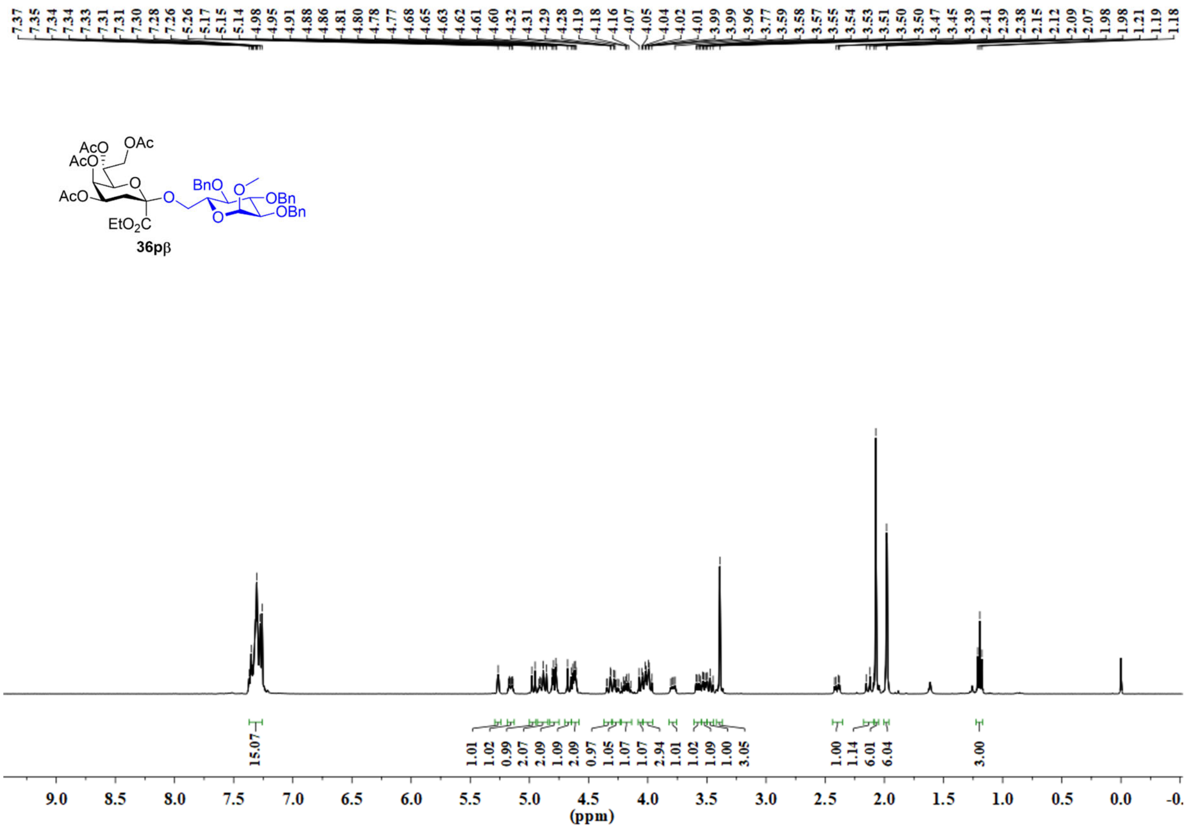

Fig. S63 ^{1}H NMR (400 MHz, CDCl3) of **36pβ**

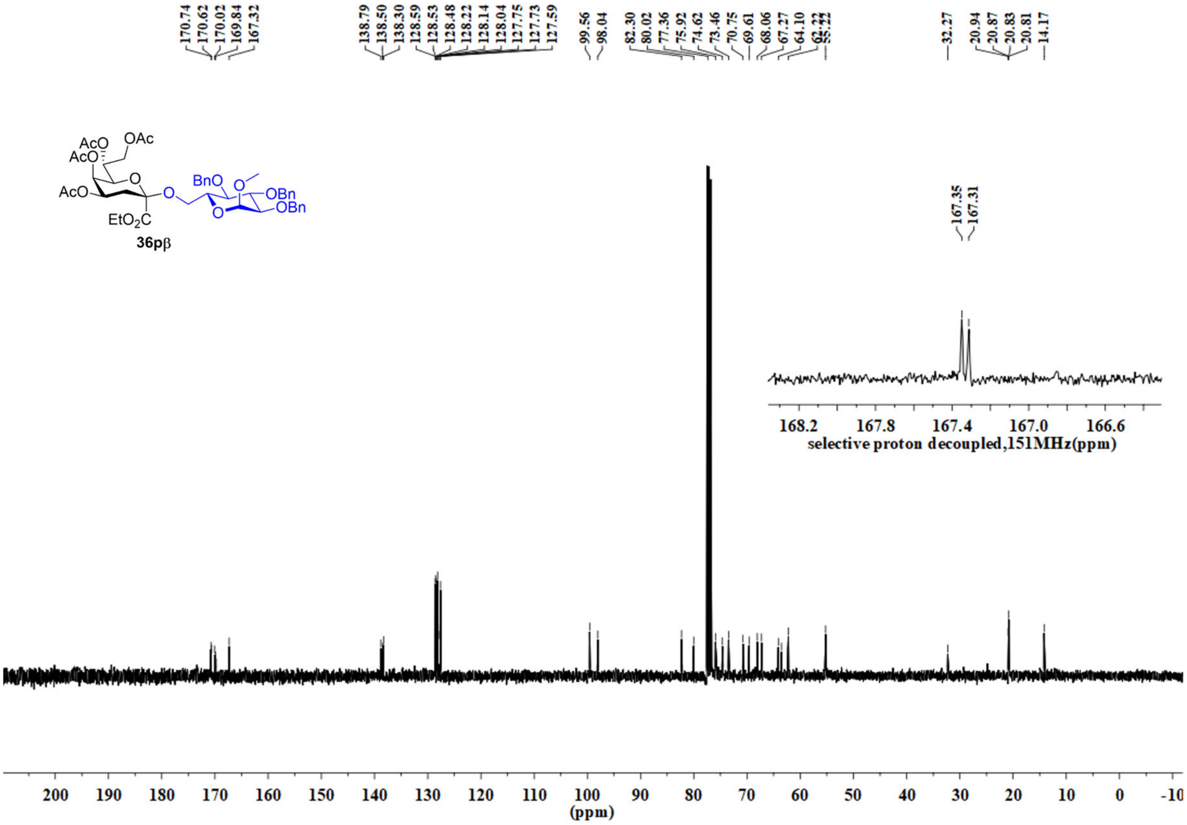

Fig. S64 ^{13}C NMR (101 MHz, CDCl3) and

selective proton decoupled ^{13}C NMR (151 MHz, CDCl3) of **36pβ**

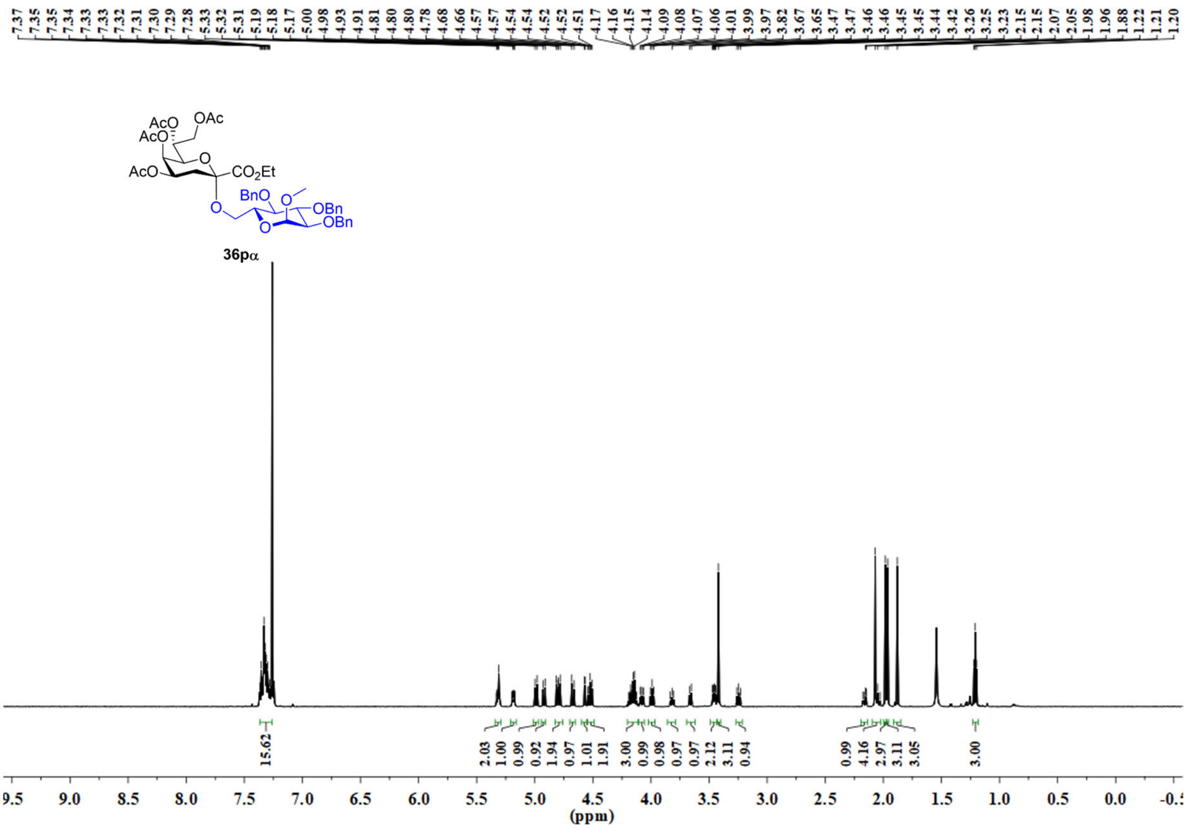

Fig. S65 ^{1}H NMR (600 MHz, $CDCl_3$) of **36pα**

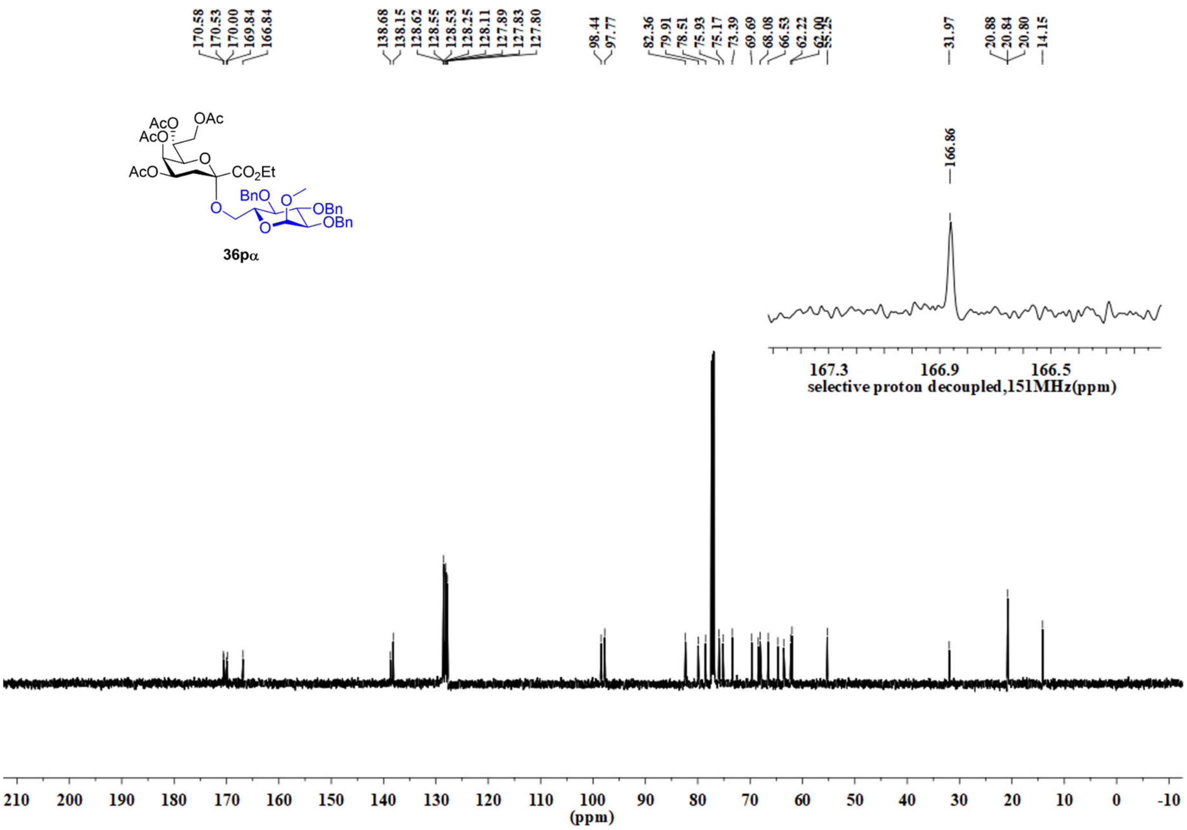

Fig. S66 ^{13}C NMR (151 MHz, $CDCl_3$) and

selective proton decoupled ^{13}C NMR (151 MHz, $CDCl_3$) of **36pα**

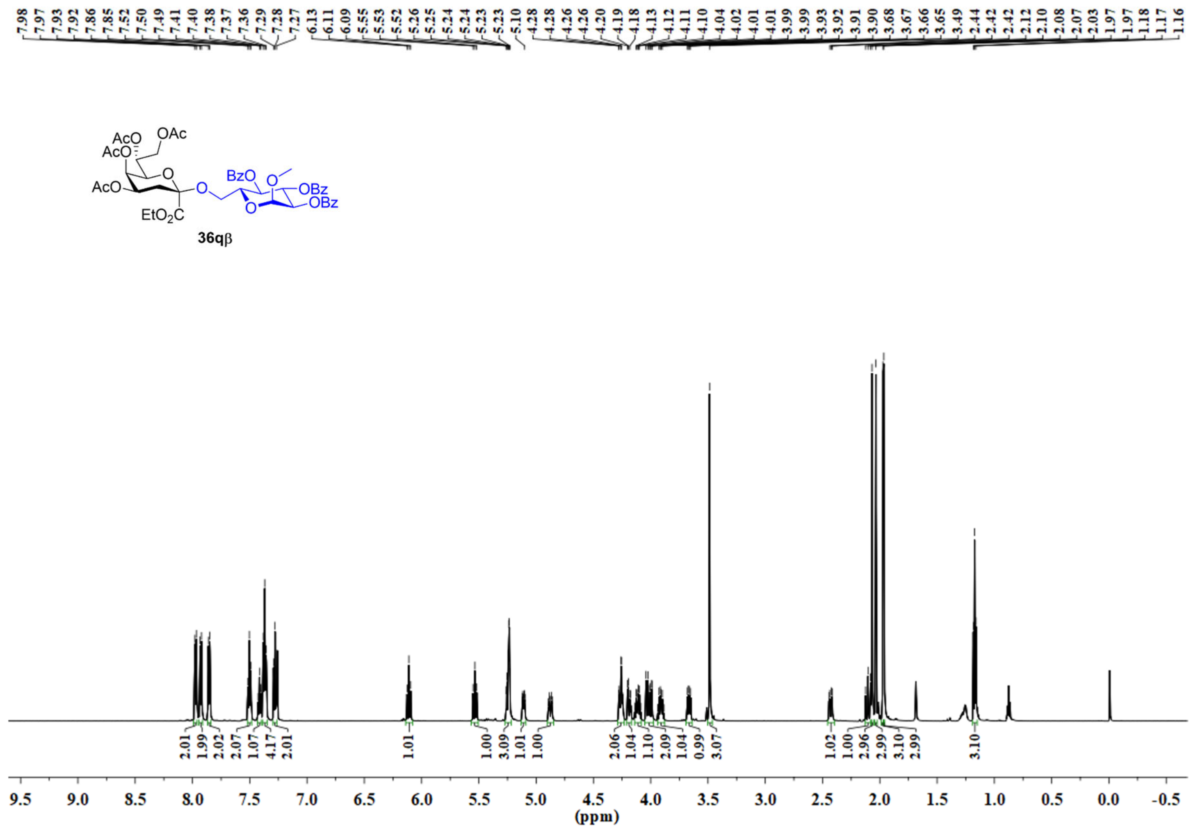

Fig. S67 ^{1}H NMR (600 MHz, $CDCl_3$) of **36qβ**

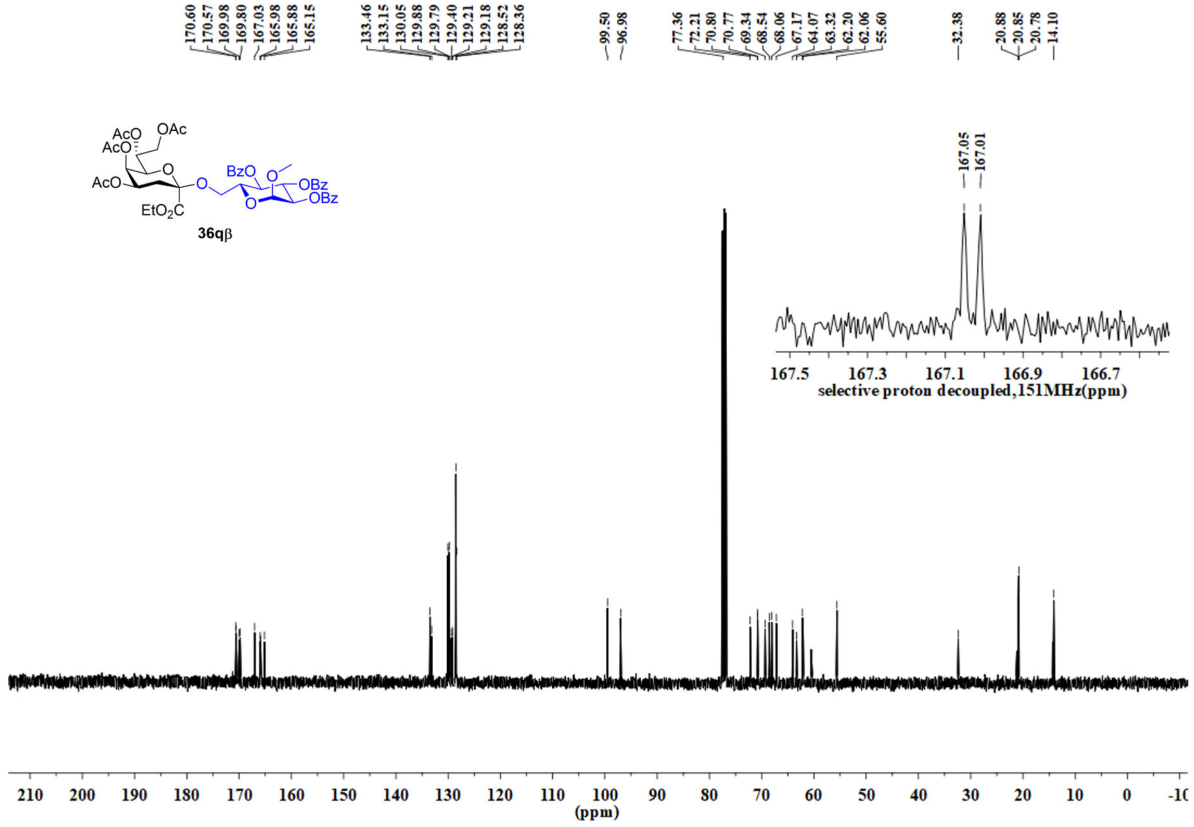

Fig. S68 ^{13}C NMR (101 MHz, $CDCl_3$) and

selective proton decoupled ^{13}C NMR (151 MHz, $CDCl_3$) of **36qβ**

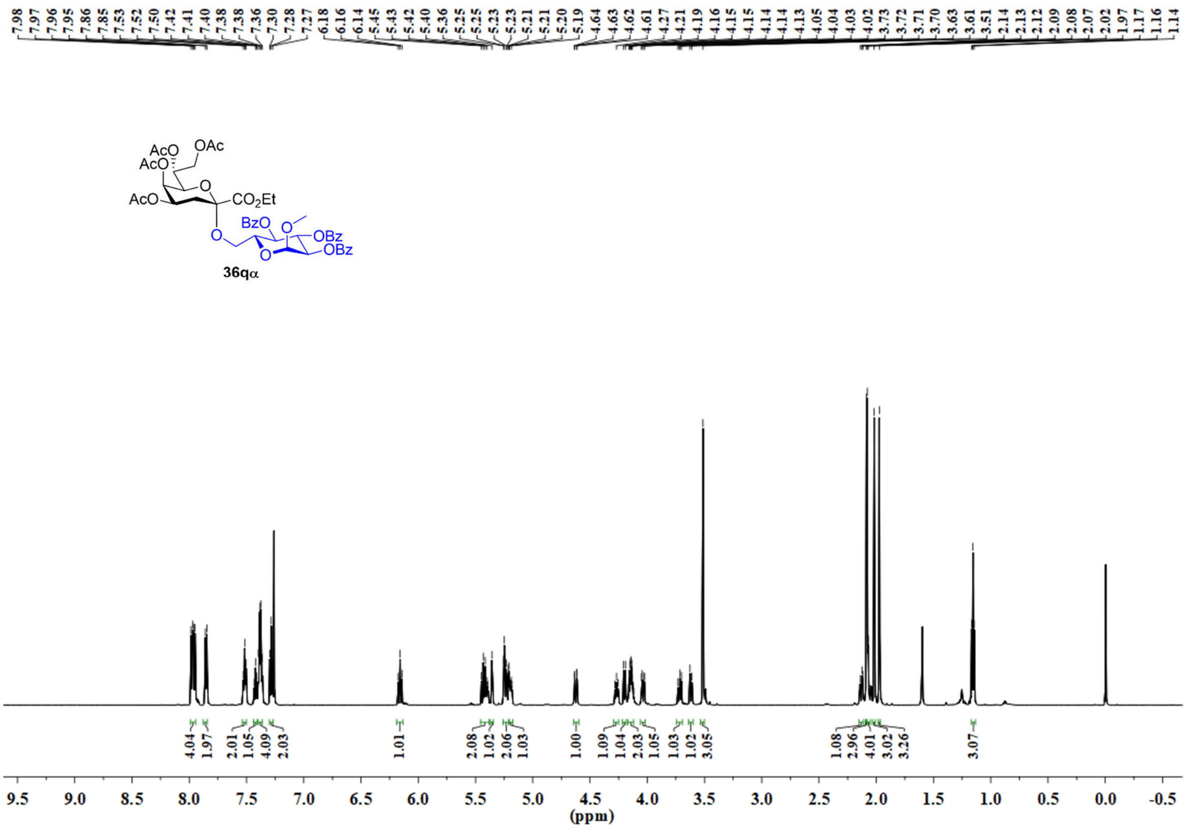

Fig. S69 ^{1}H NMR (600 MHz, CDCl3) of **36qα**

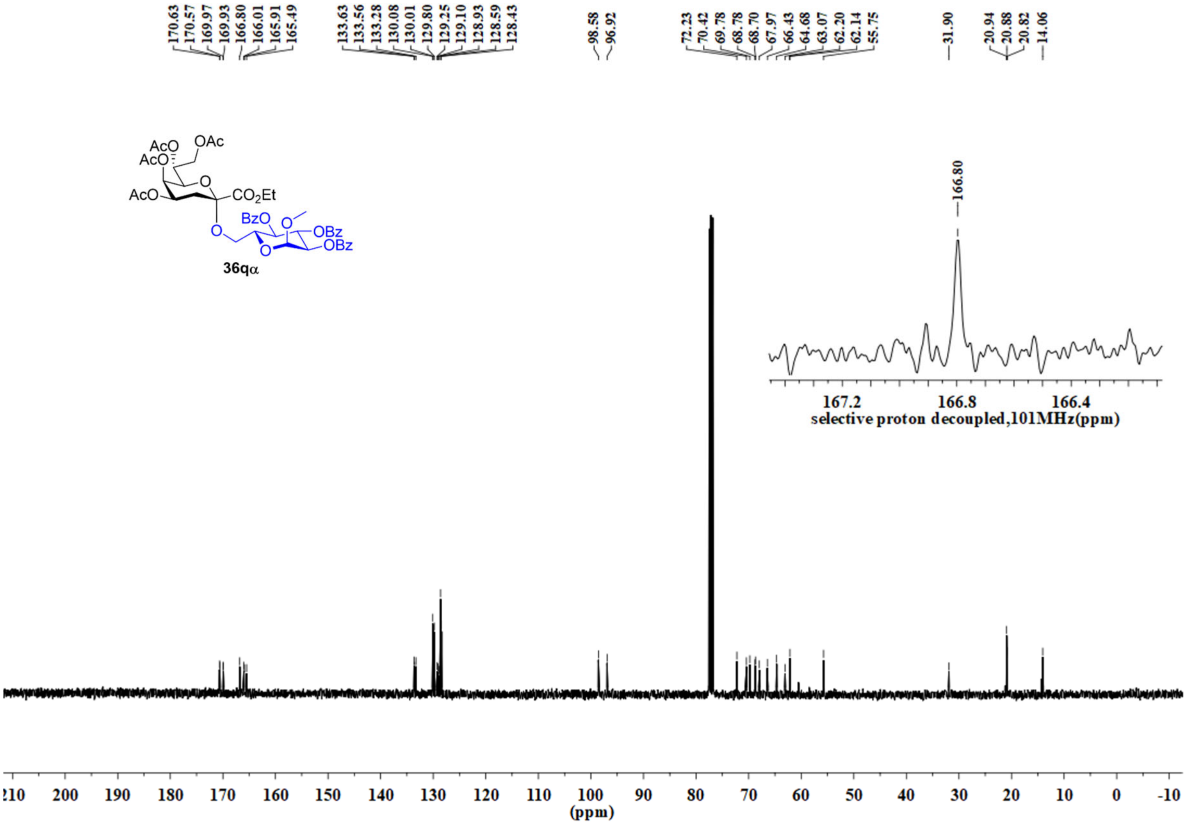

Fig. S70 ^{13}C NMR (101 MHz, CDCl3) and

selective proton decoupled ^{13}C NMR (101 MHz, CDCl3) of **36qα**

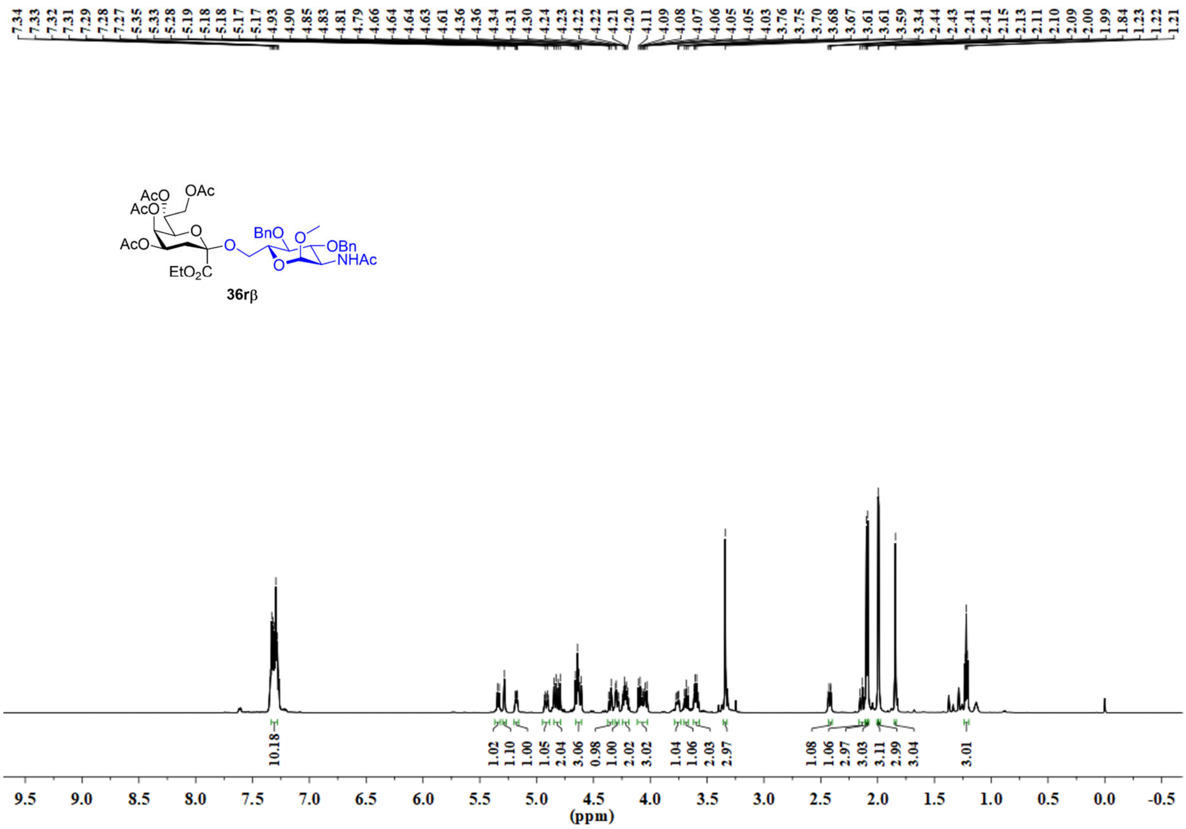

Fig. S71 ^{1}H NMR (600 MHz, $CDCl_3$) of **36rβ**

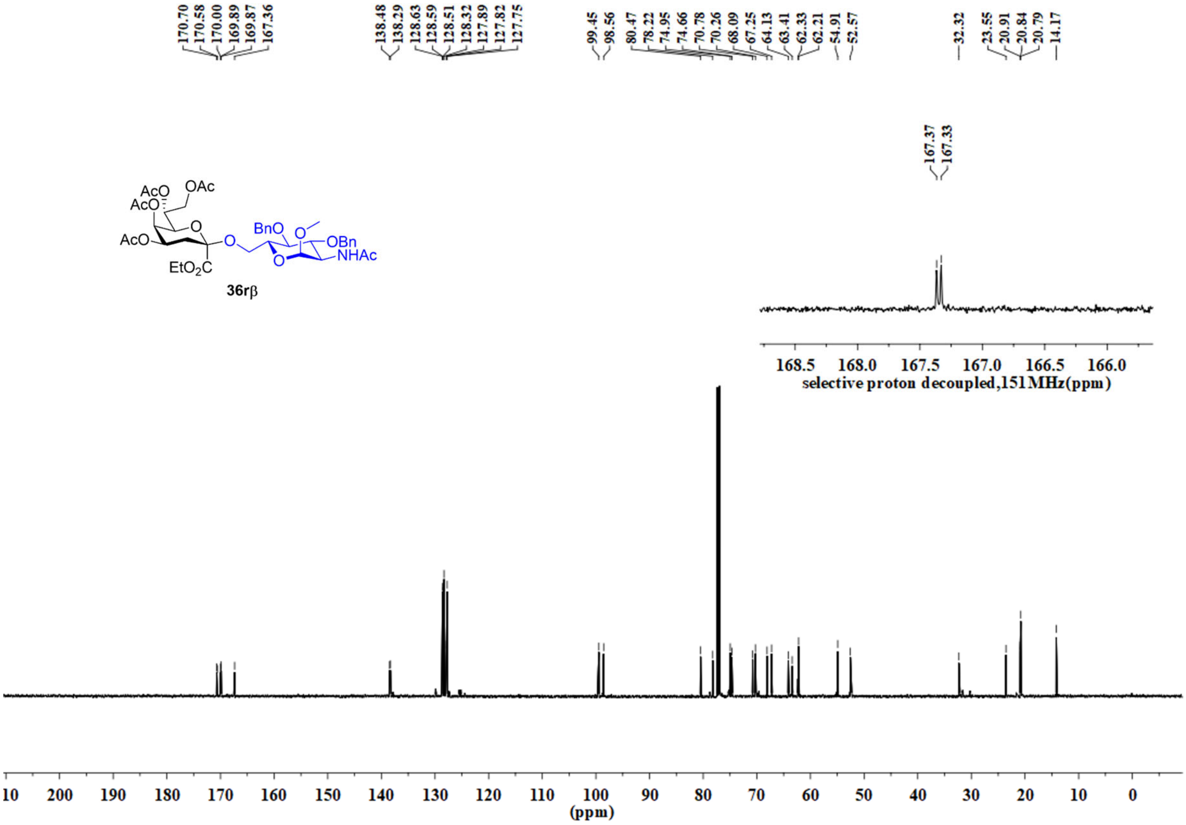

Fig. S72 ^{13}C NMR (151 MHz, $CDCl_3$) and

selective proton decoupled ^{13}C NMR (151 MHz, $CDCl_3$) of **36rβ**

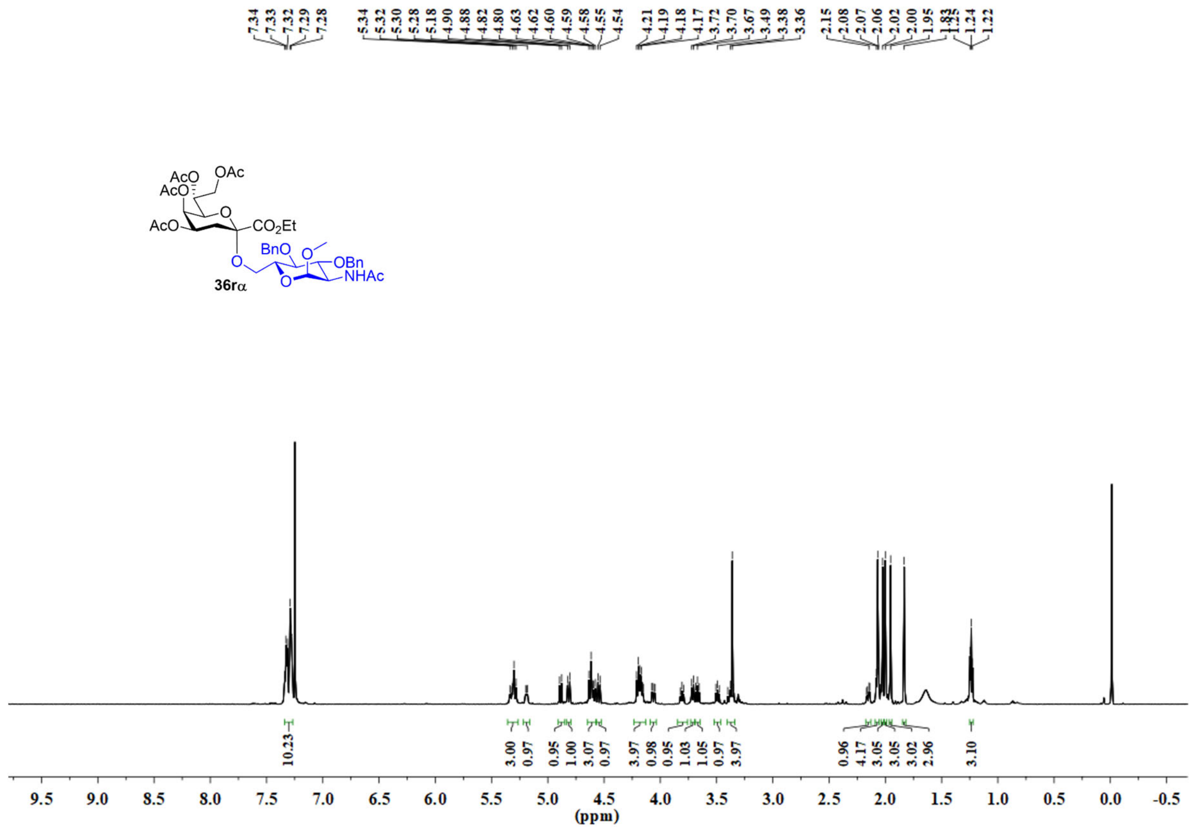

Fig. S73 ^{1}H NMR (600 MHz, $CDCl_3$) of **36rα**

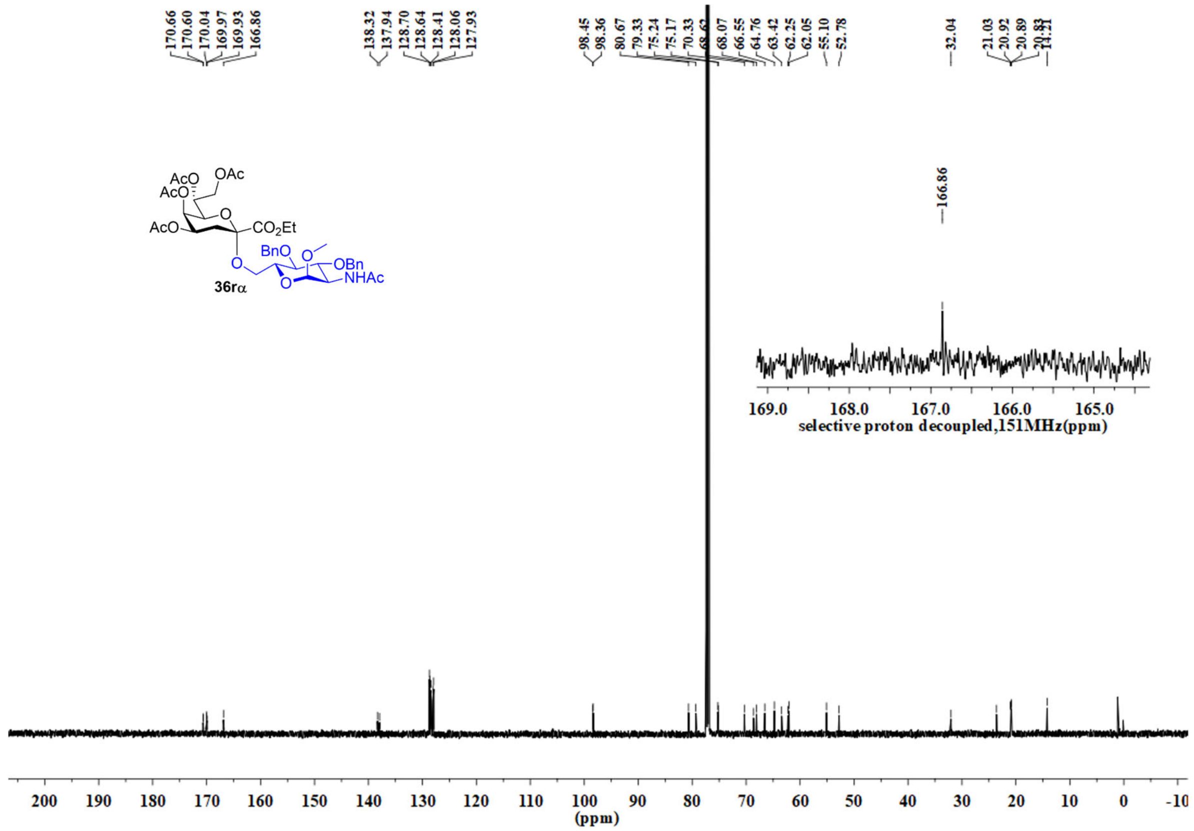

Fig. S74 ^{13}C NMR (151 MHz, $CDCl_3$) and

selective proton decoupled ^{13}C NMR (151 MHz, $CDCl_3$) of **36rα**

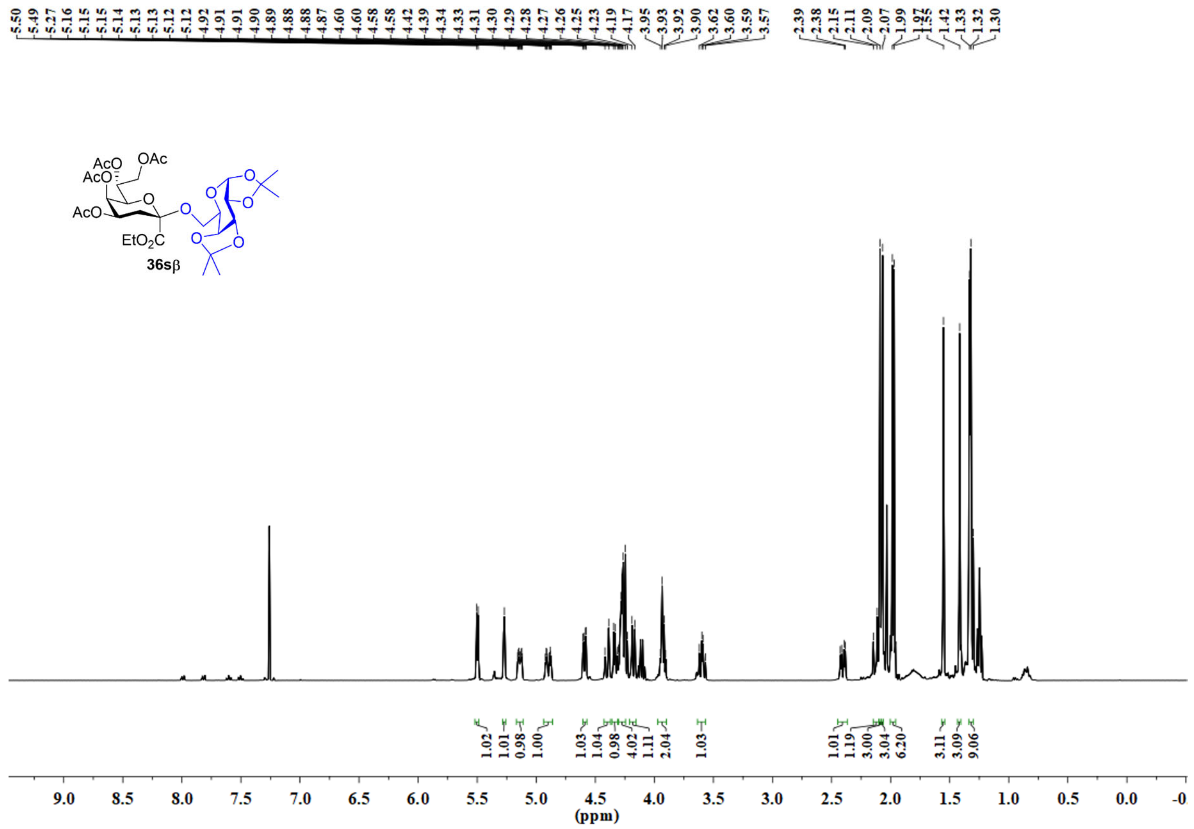

Fig. S75 ^{1}H NMR (400 MHz, CDCl3) of **36sβ**

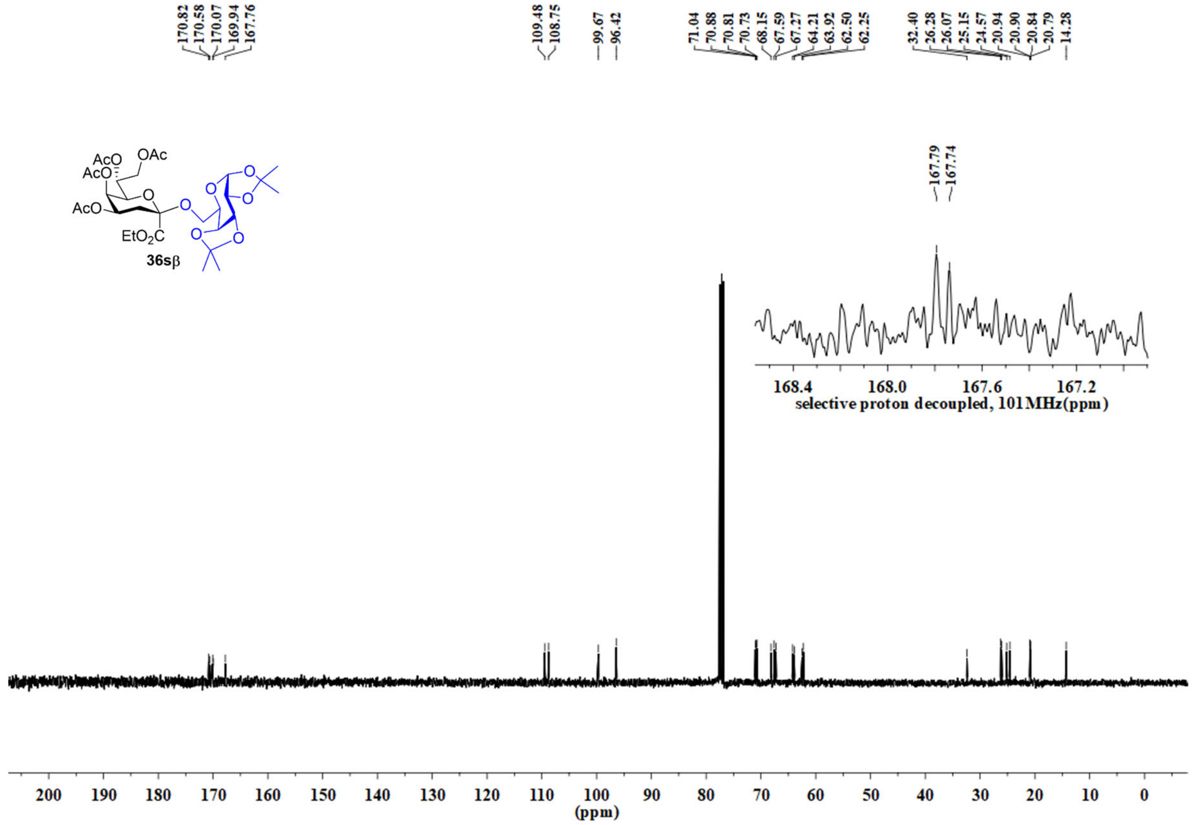

Fig. S76 ^{13}C NMR (101 MHz, CDCl3) and

selective proton decoupled ^{13}C NMR (101 MHz, CDCl3) of **36sβ**

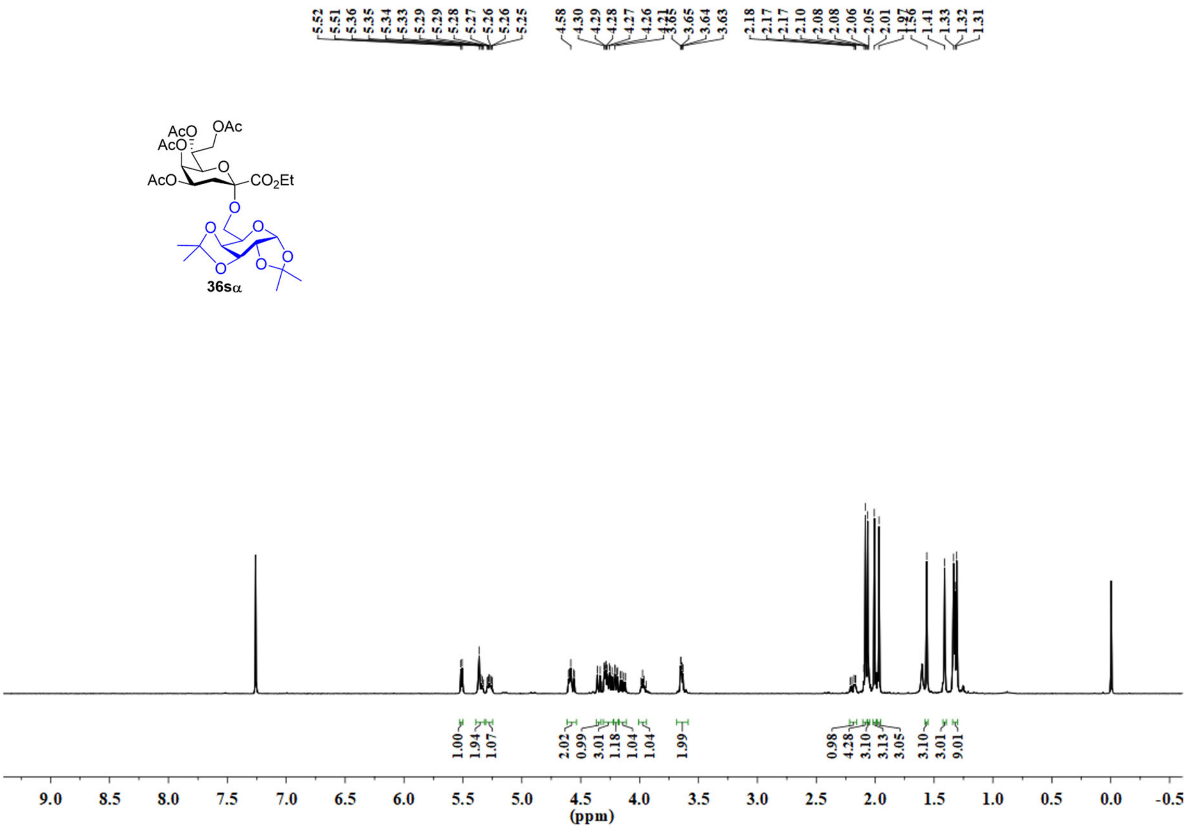

Fig. S77 ^{1}H NMR (400 MHz, $CDCl_3$) of **36sα**

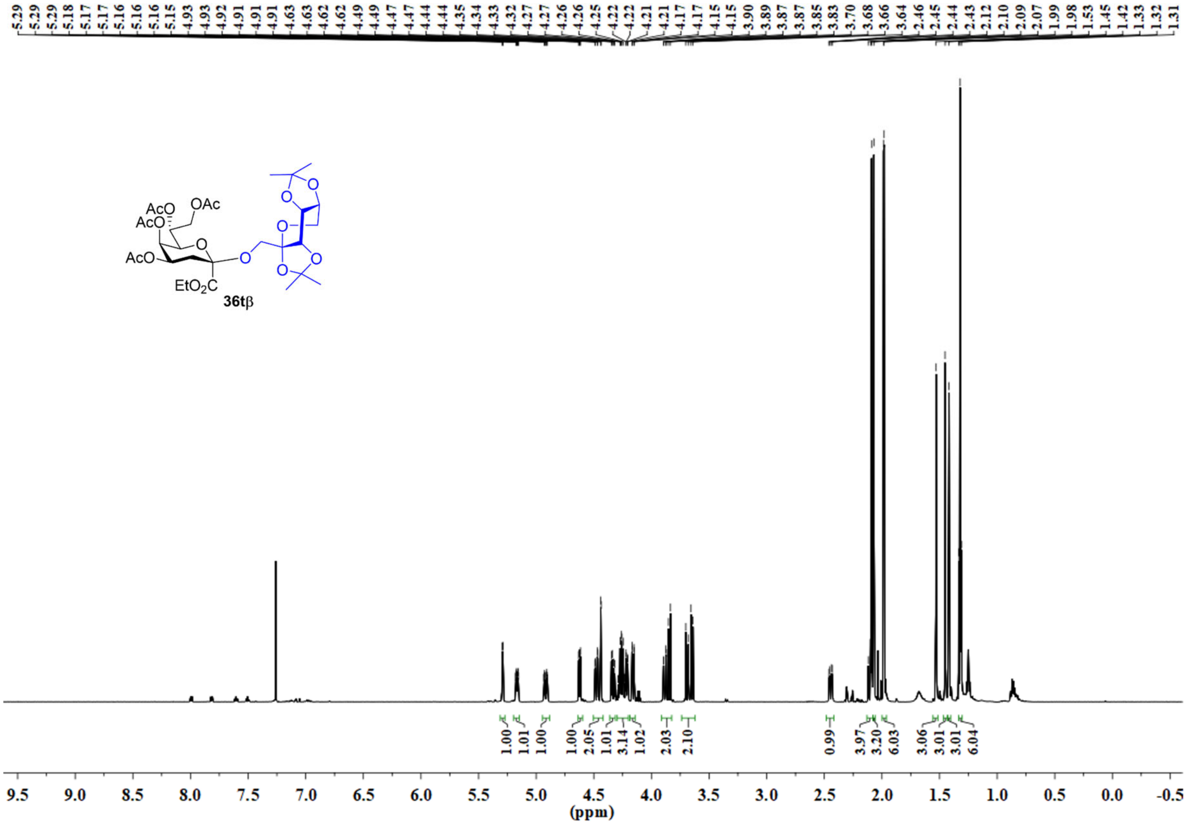

Fig. S78 ^{1}H NMR (600 MHz, $CDCl_3$) of **36tβ**

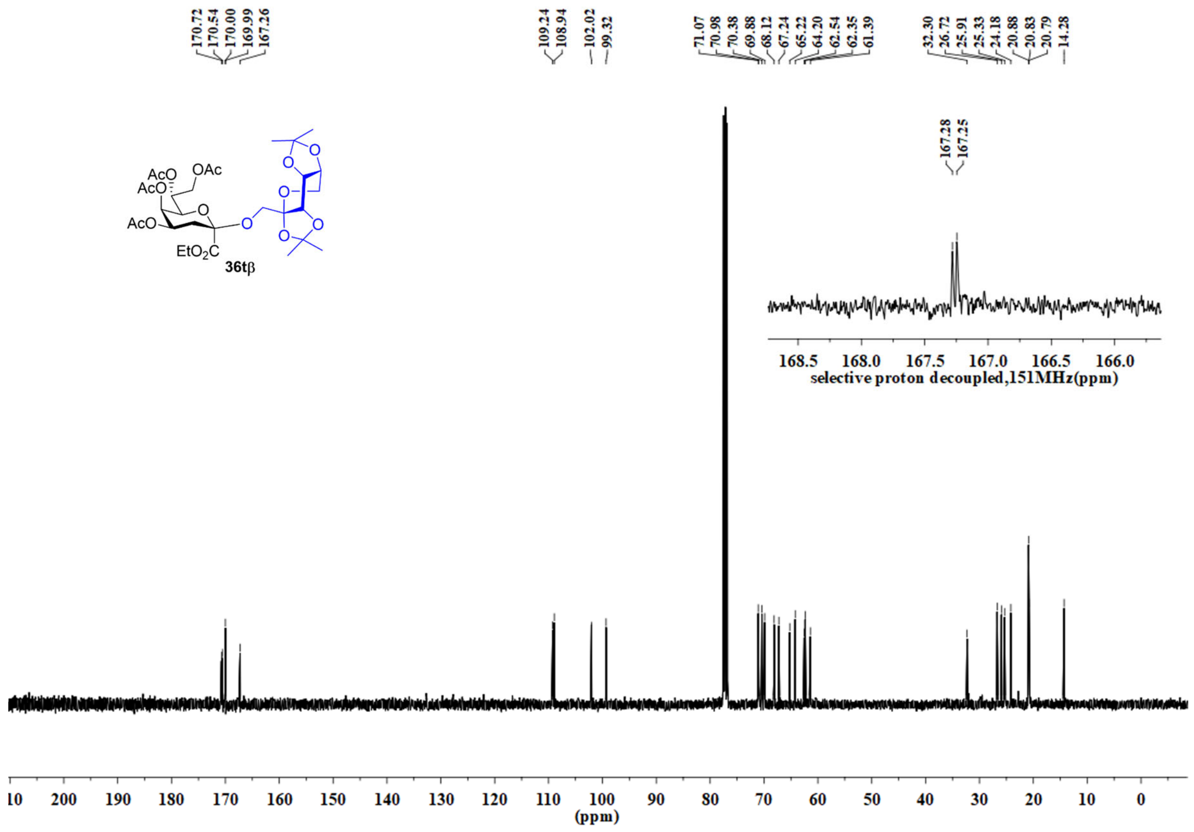

Fig. S79 ^{13}C NMR (101 MHz, $CDCl_3$) and

selective proton decoupled ^{13}C NMR (151 MHz, $CDCl_3$) of **36tβ**

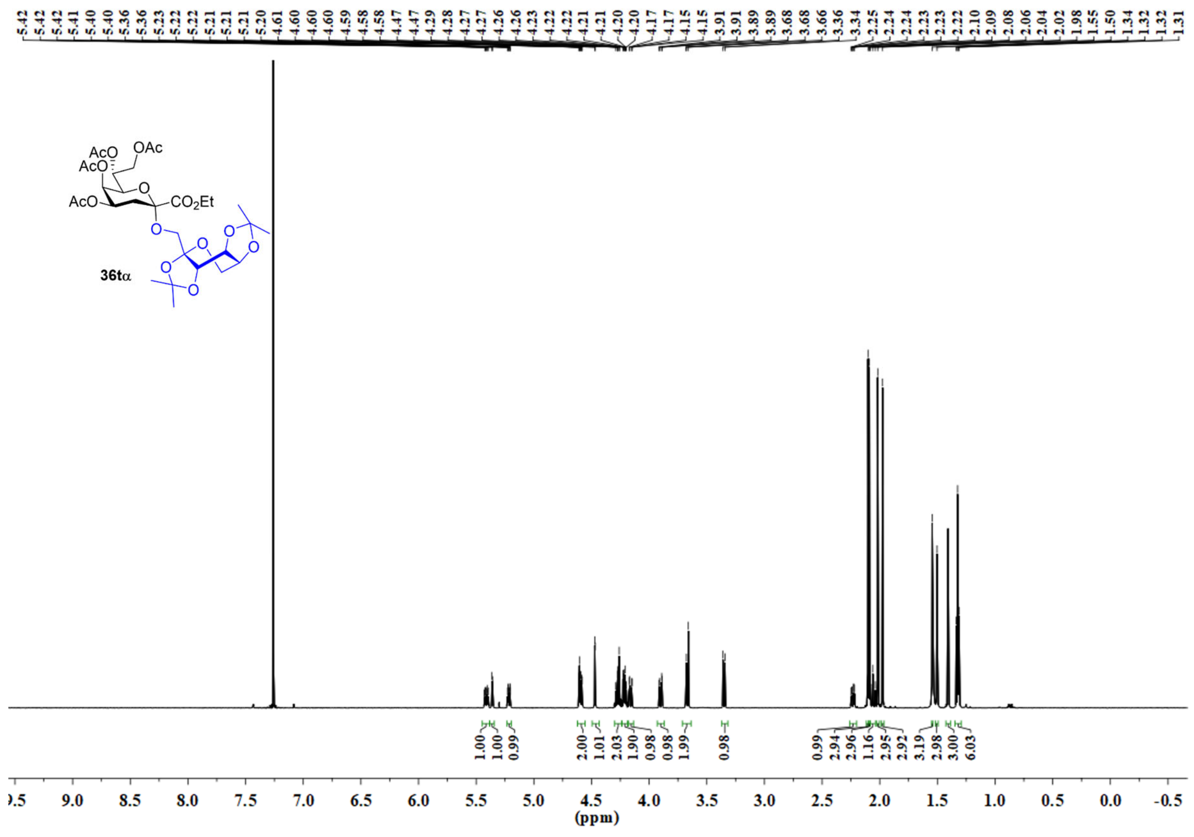

Fig. S80 ^{1}H NMR (600 MHz, $CDCl_3$) of **36tα**

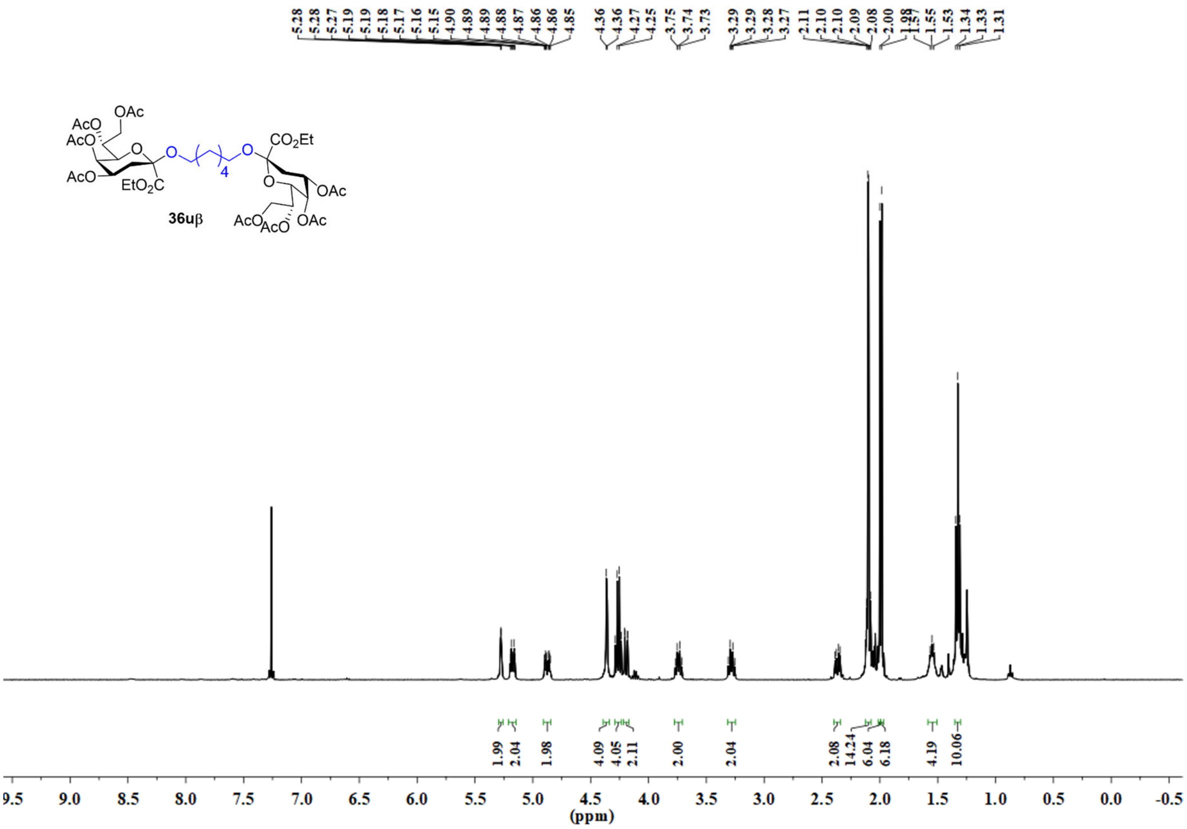

Fig. S81 ^{1}H NMR (400 MHz, $CDCl_3$) of **36uβ**

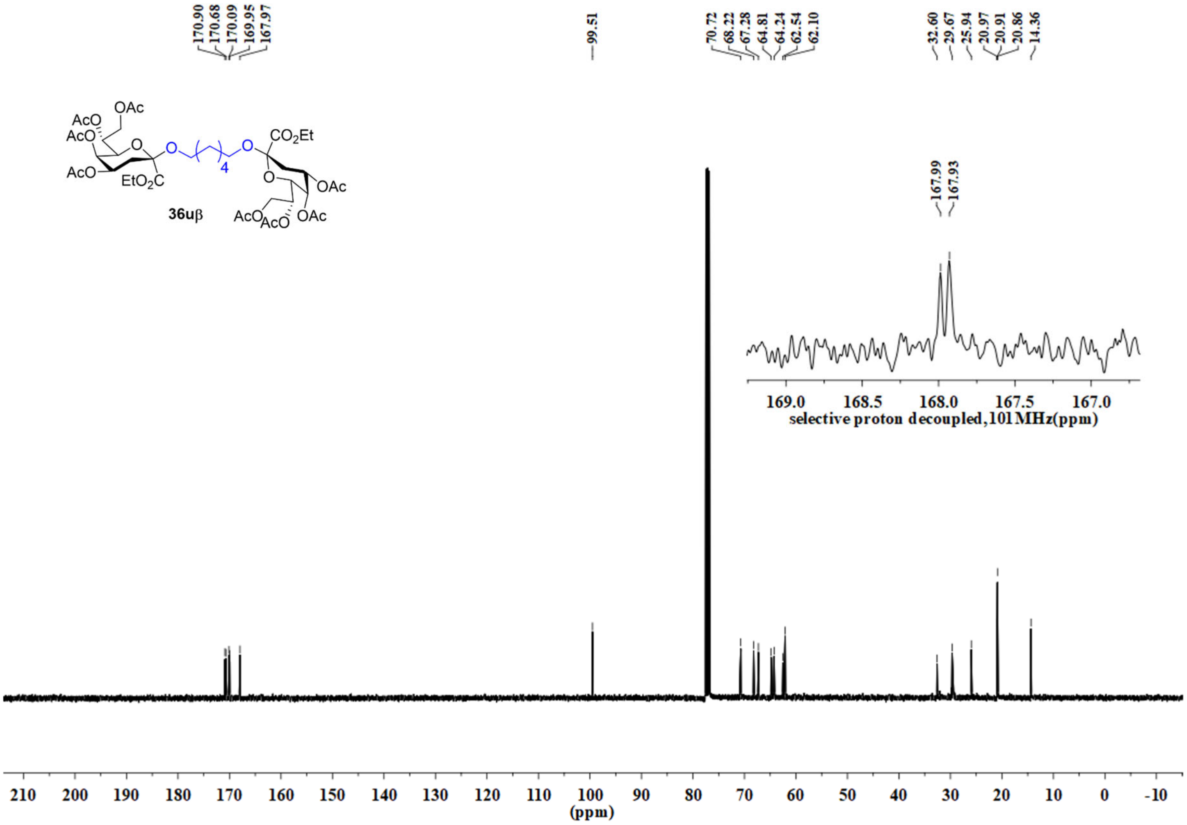

Fig. S82 ^{13}C NMR (101 MHz, $CDCl_3$) and

selective proton decoupled ^{13}C NMR (101 MHz, $CDCl_3$) of **36uβ**

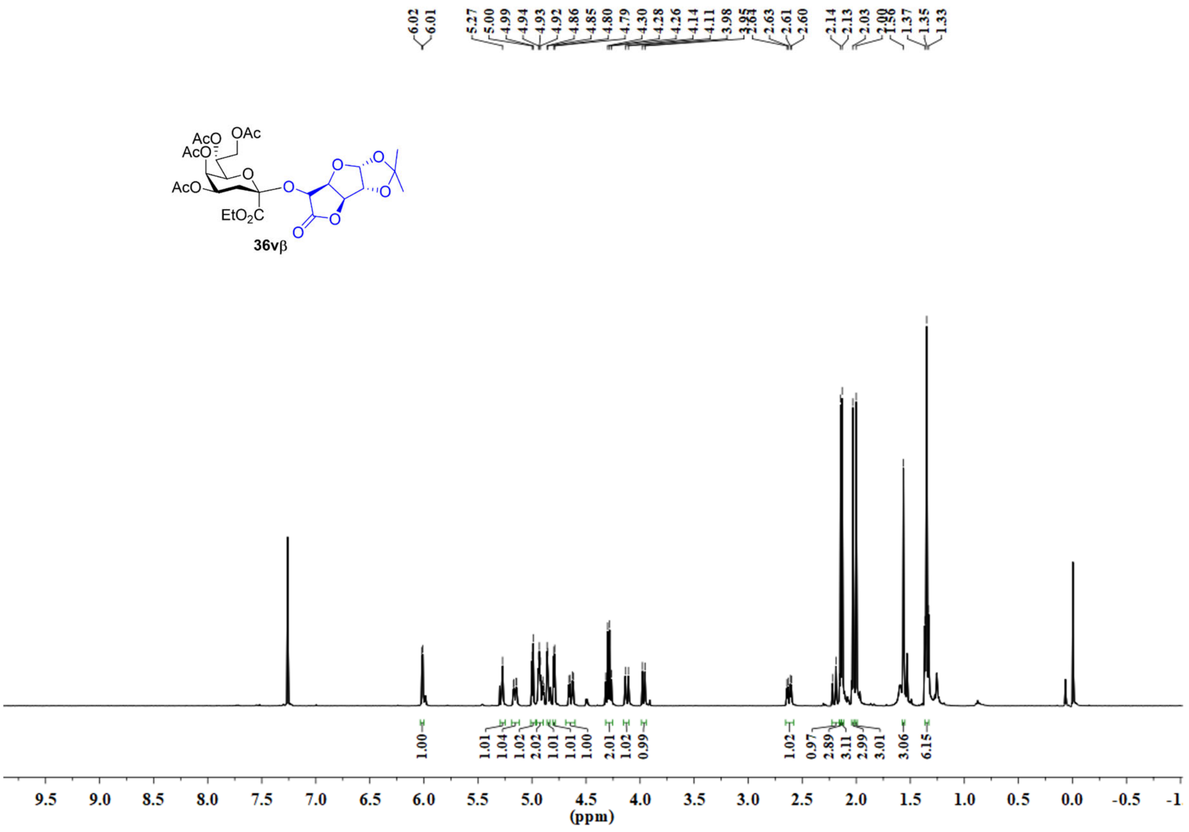

Fig. S83 ^{1}H NMR (600 MHz, $CDCl_3$) of **36vβ**

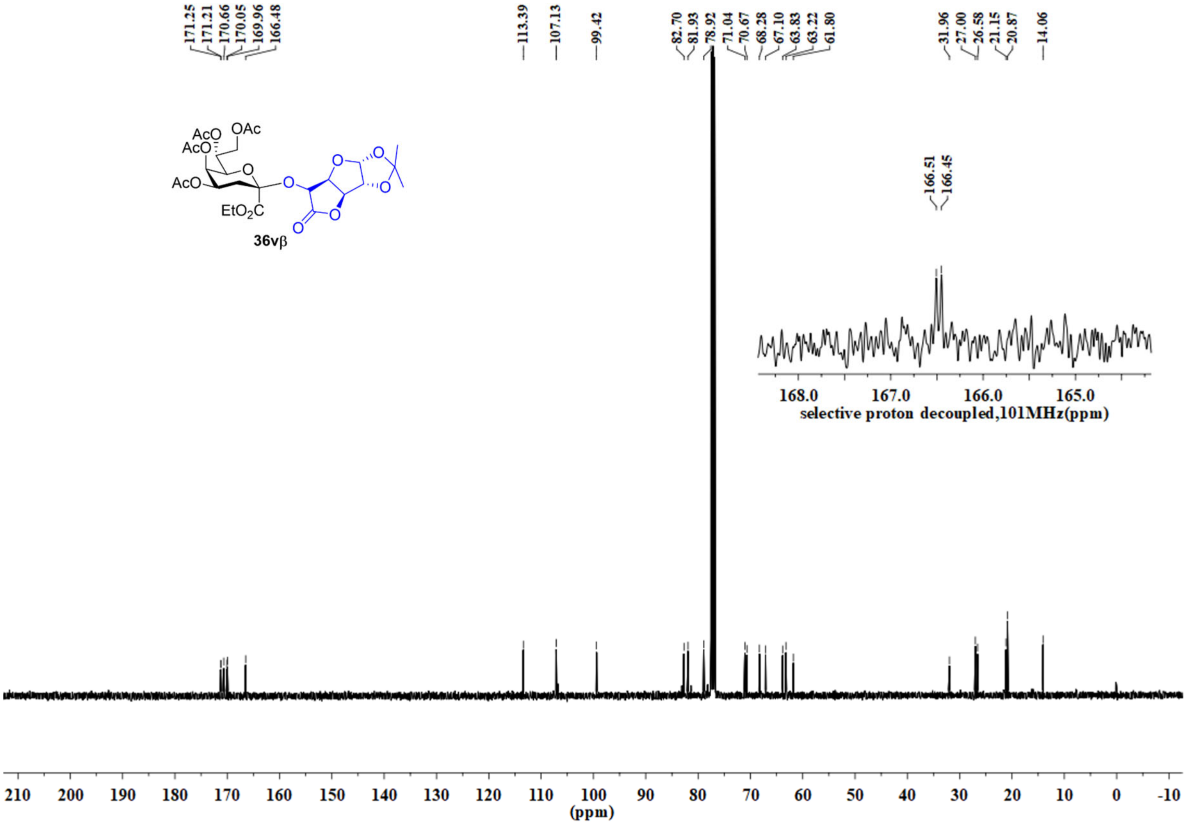

Fig. S84 ^{13}C NMR (101 MHz, $CDCl_3$) and

selective proton decoupled ^{13}C NMR (101 MHz, $CDCl_3$) of **36vβ**

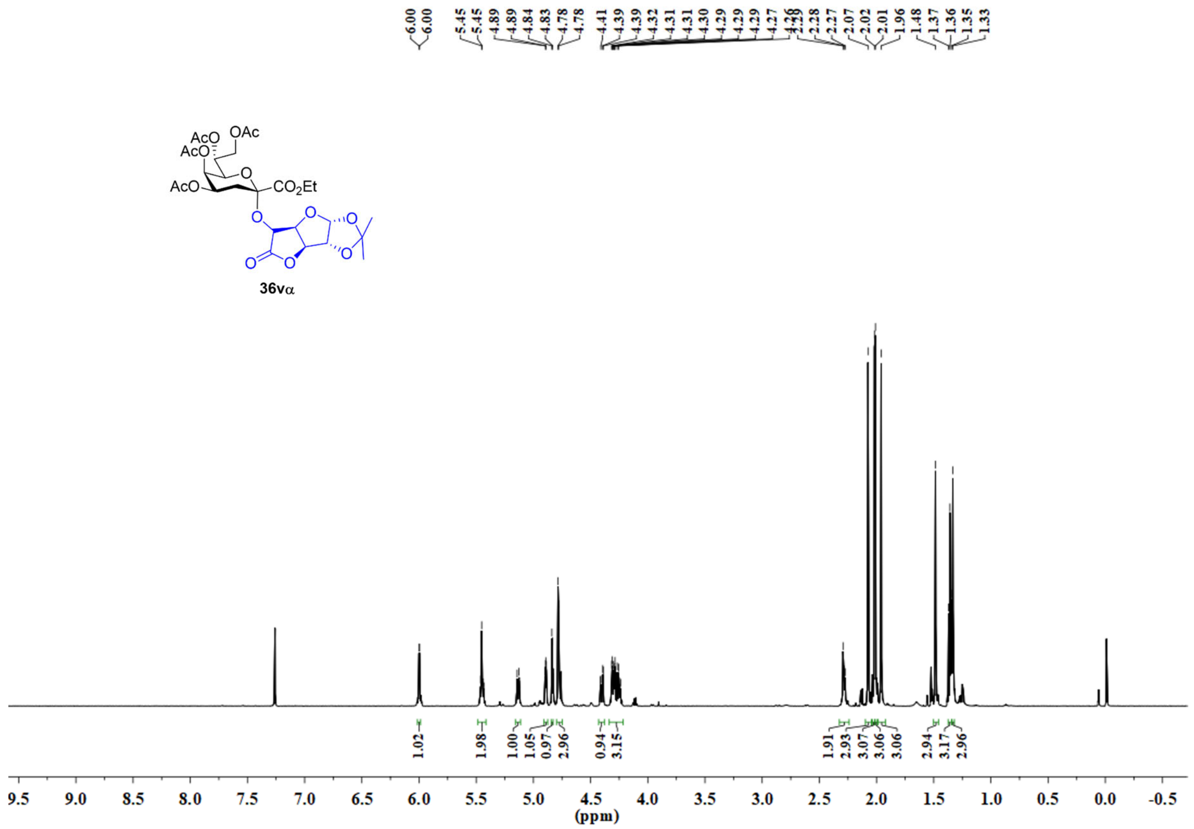

Fig. S85 ^{1}H NMR (600 MHz, $CDCl_3$) of **36vα**

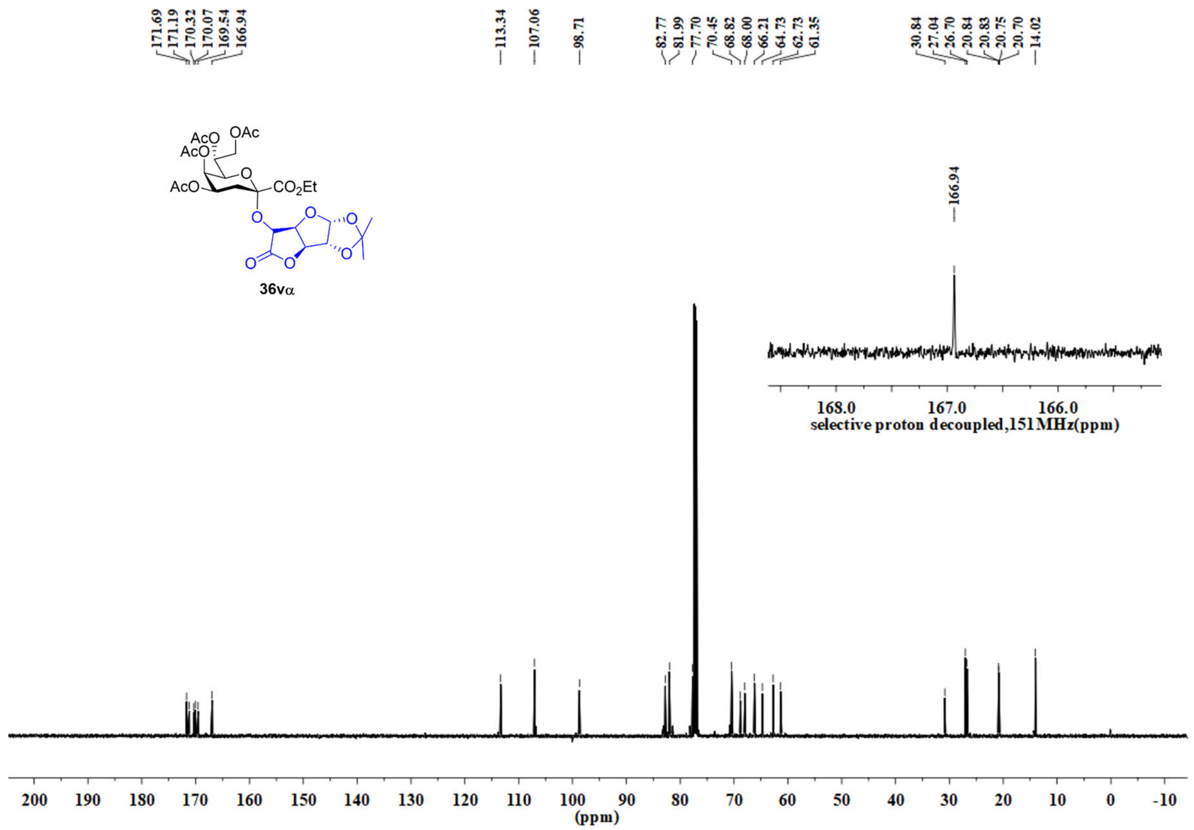

Fig. S86 ^{13}C NMR (151 MHz, $CDCl_3$) and

selective proton decoupled ^{13}C NMR (151 MHz, $CDCl_3$) of **36vα**

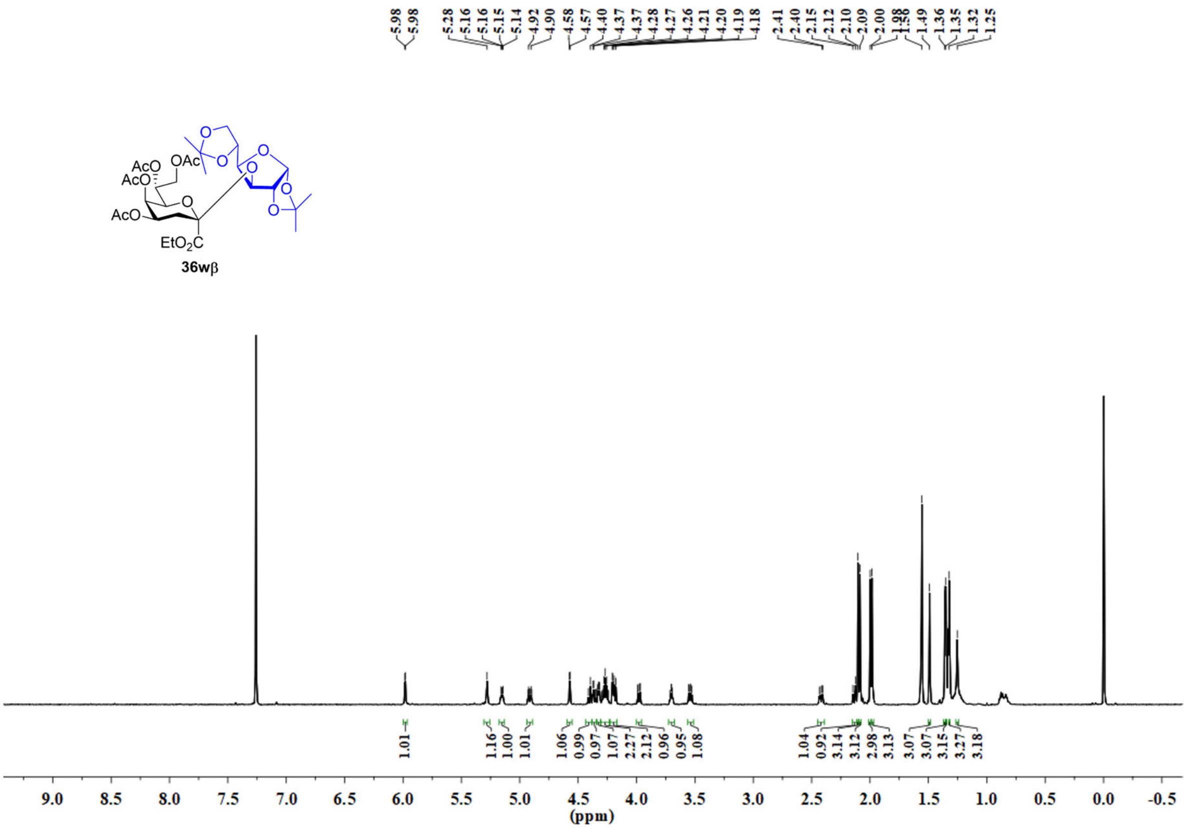

Fig. S87 ^{1}H NMR (600 MHz, CDCl3) of **36wβ**

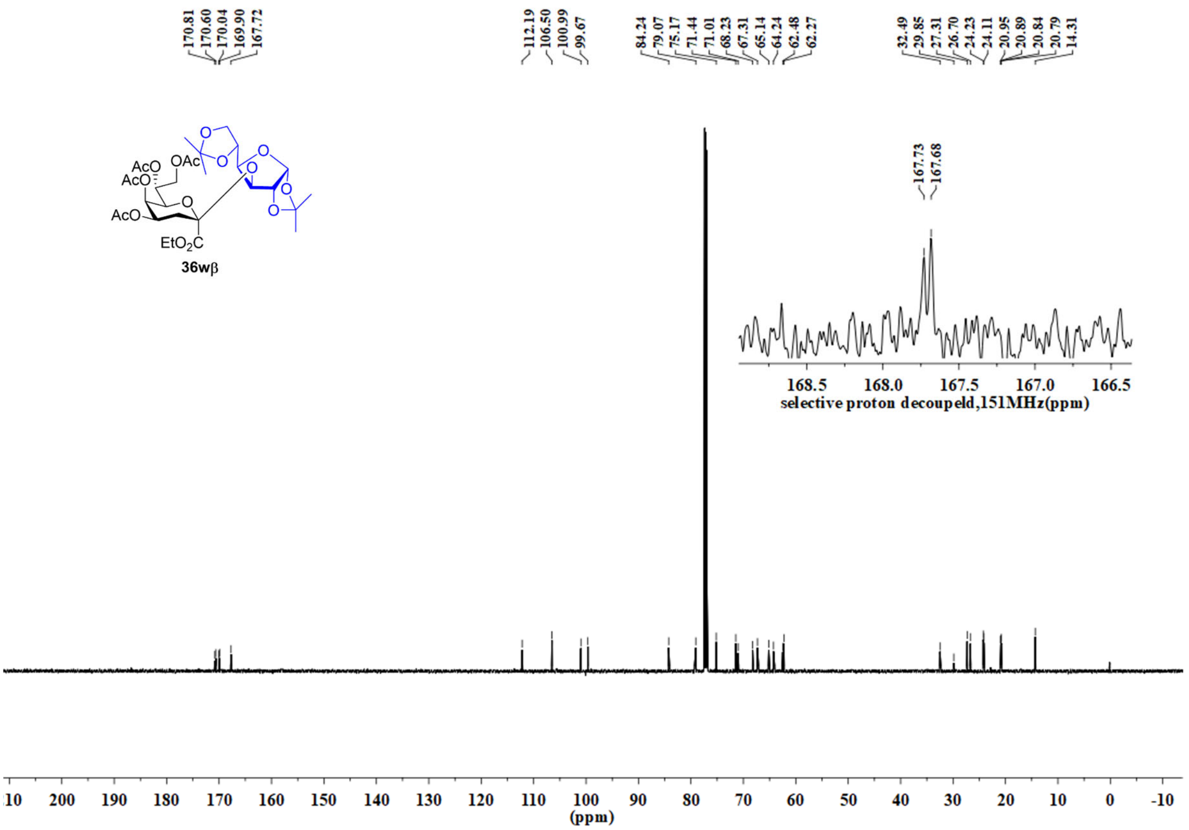

Fig. S88 ^{13}C NMR (151 MHz, CDCl3) and

selective proton decoupled ^{13}C NMR (151 MHz, CDCl3) of **36wβ**

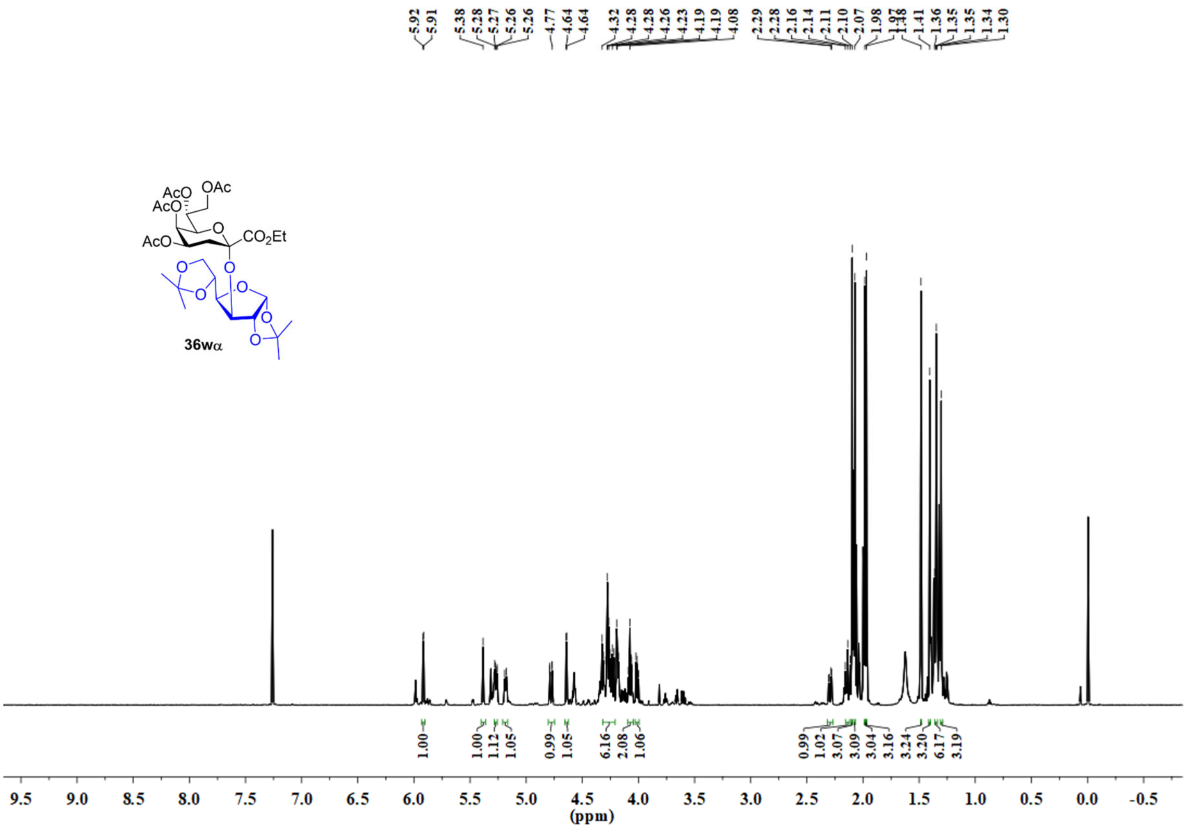

Fig. S89 ^{1}H NMR (600 MHz, $CDCl_3$) of **36wα**

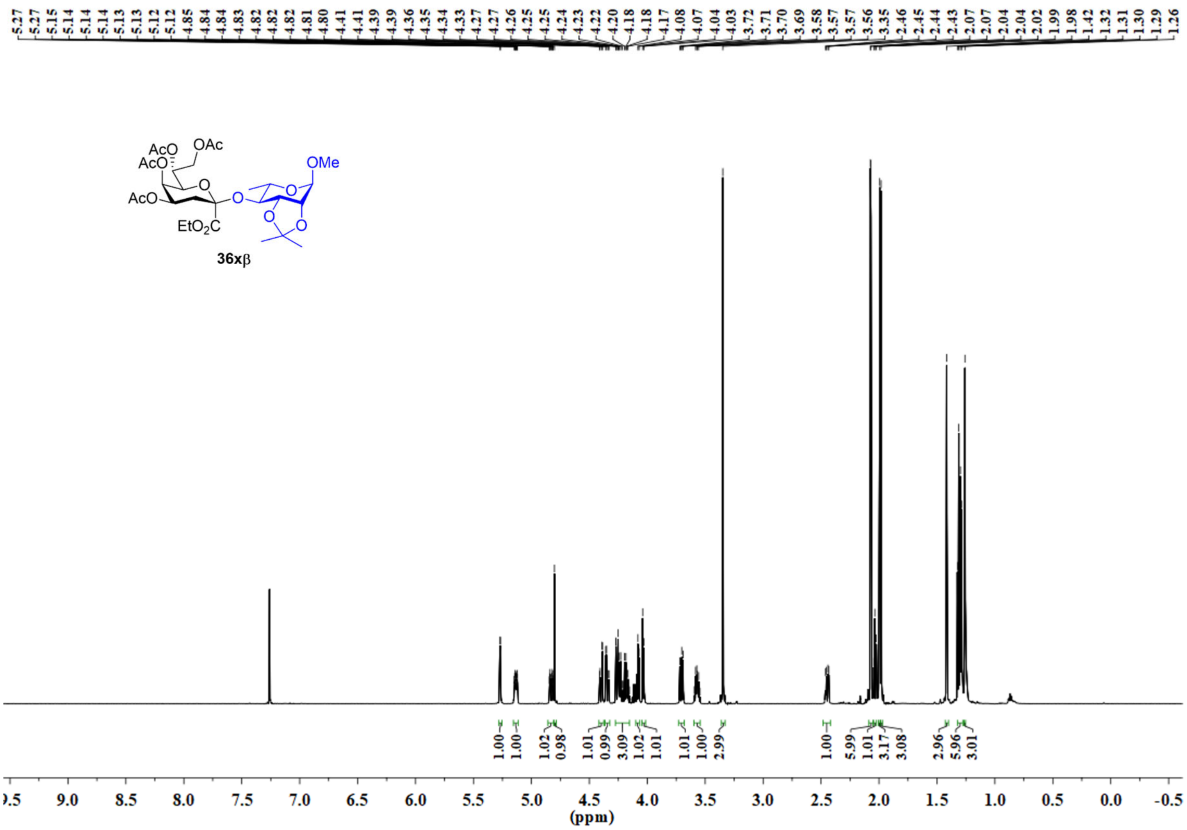

Fig. S90 ^{1}H NMR (600 MHz, $CDCl_3$) of **36xβ**

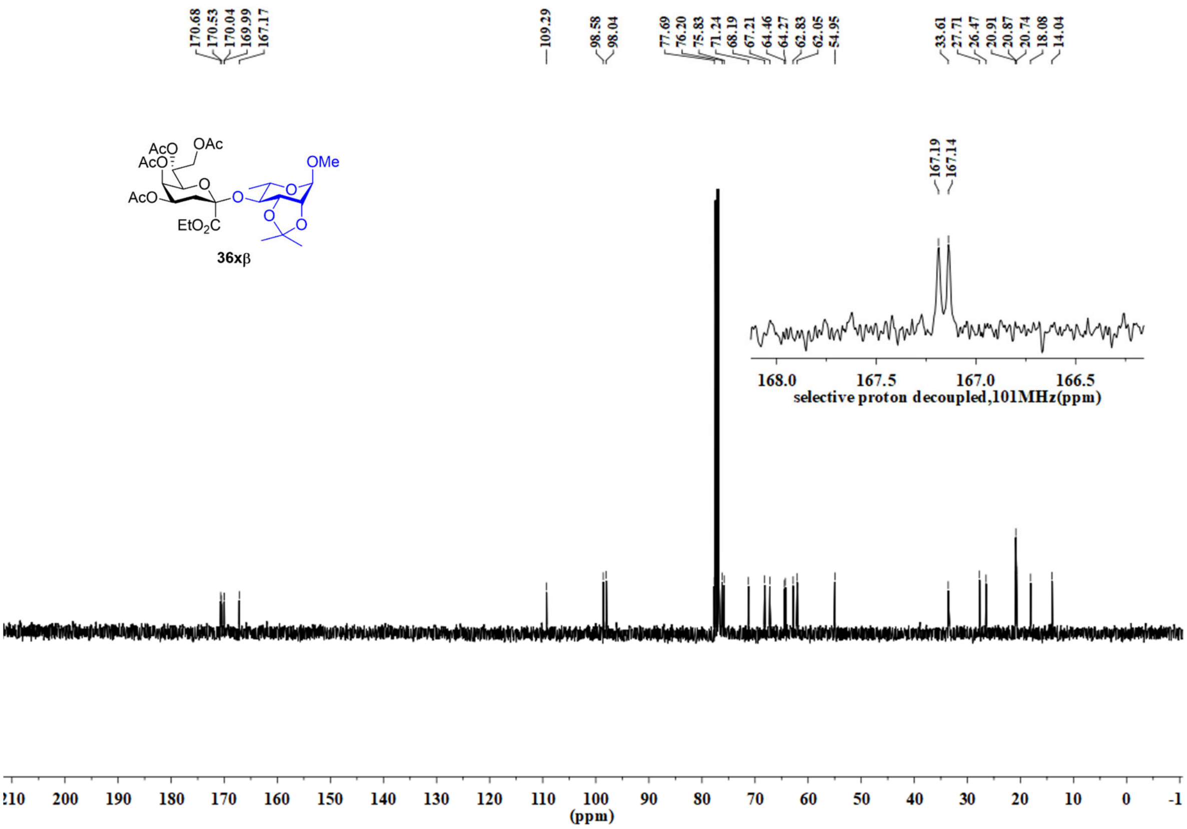

Fig. S91 ^{13}C NMR (101 MHz, $CDCl_3$) and

selective proton decoupled ^{13}C NMR (101 MHz, $CDCl_3$) of **36xβ**

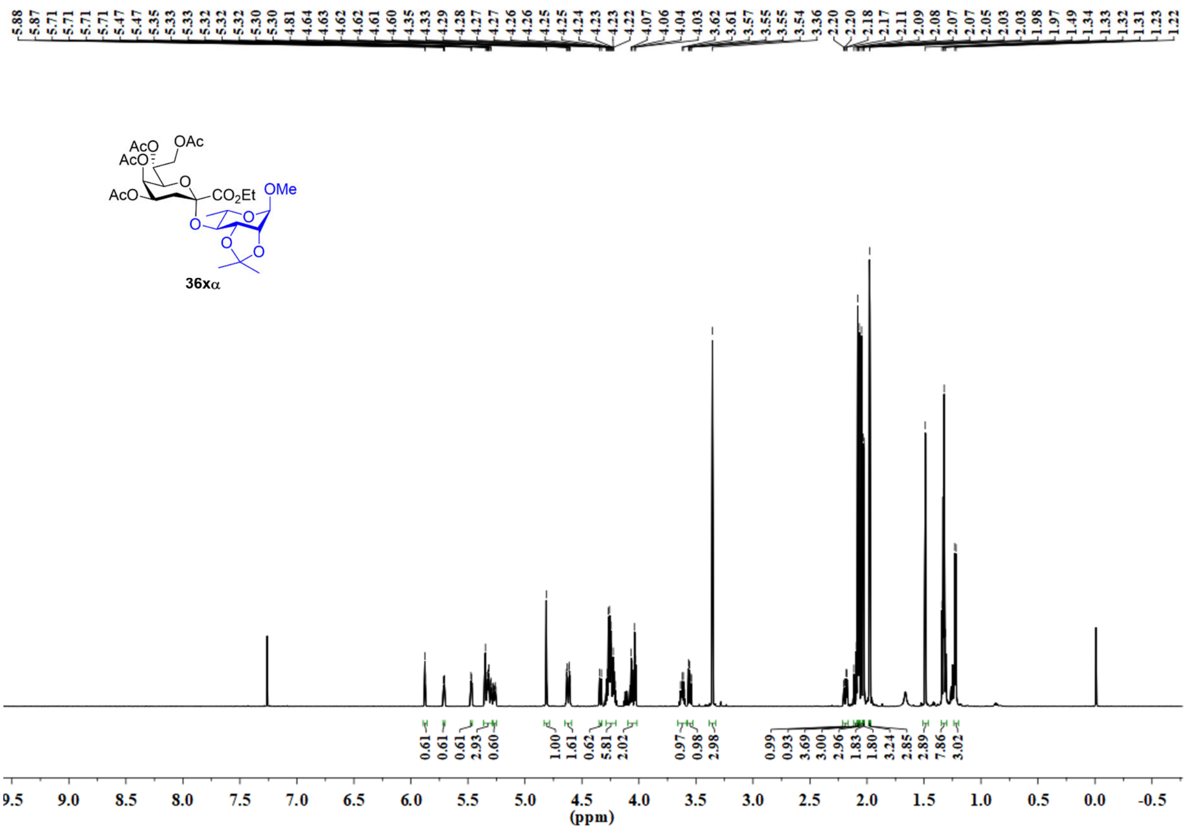

Fig. S92 ^{1}H NMR (600 MHz, $CDCl_3$) of **36xα**

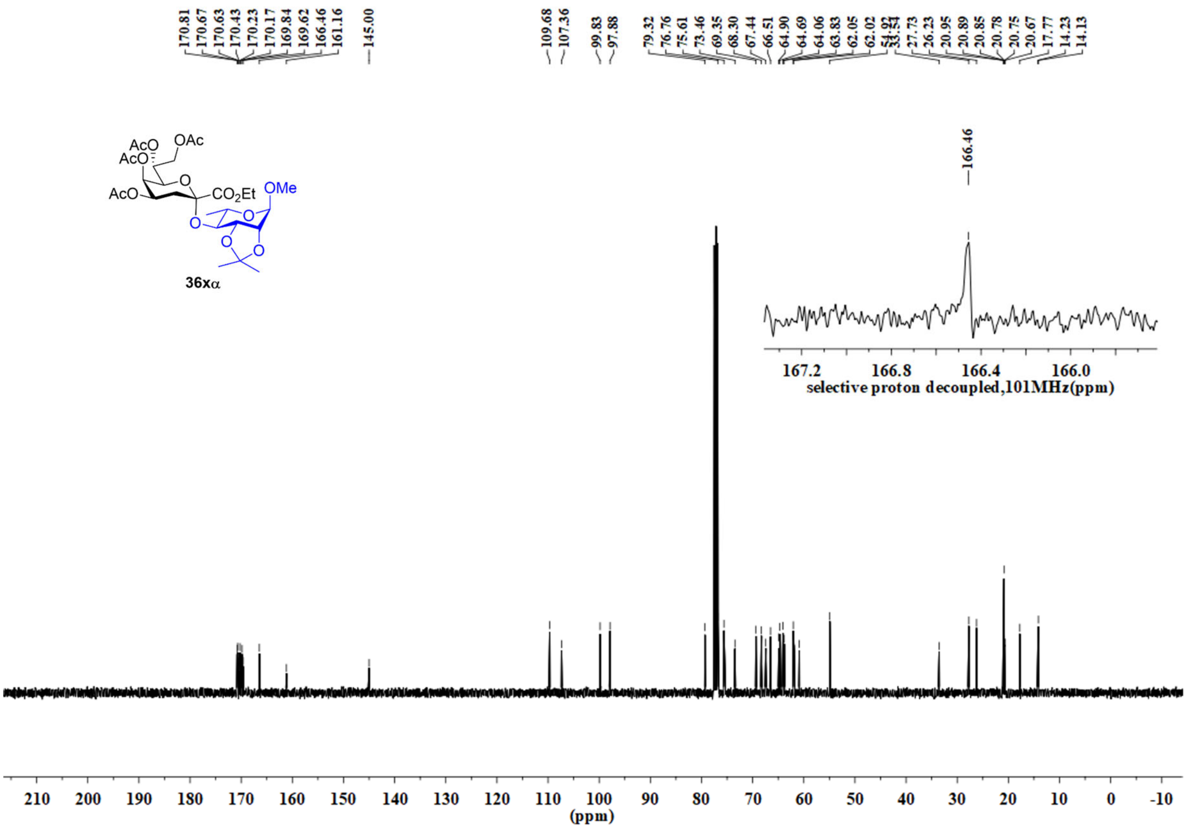

Fig. S93 ^{13}C NMR (101 MHz, $CDCl_3$) and

selective proton decoupled ^{13}C NMR (101 MHz, $CDCl_3$) of **36xα**

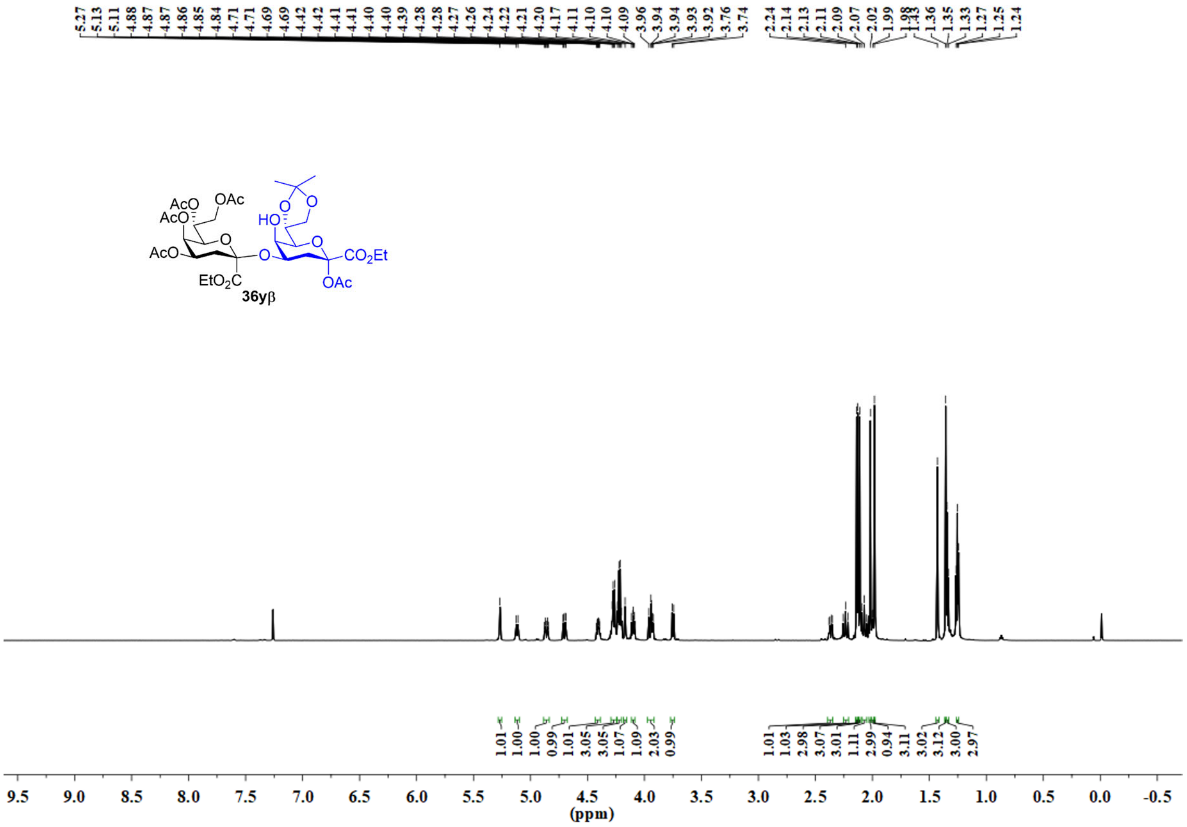

Fig. S94 ^{1}H NMR (600 MHz, $CDCl_3$) of **36yβ**

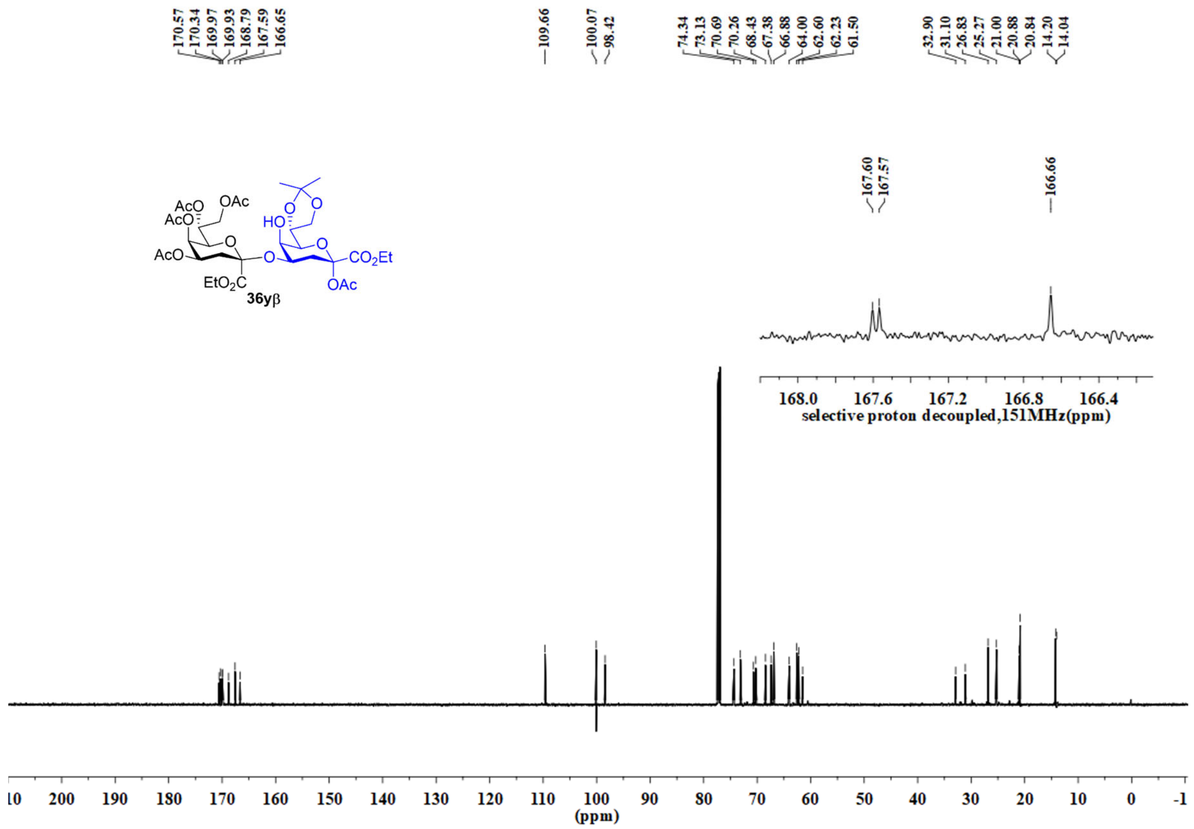

Fig. S95 ^{13}C NMR (151 MHz, CDCl3) and

selective proton decoupled ^{13}C NMR (151 MHz, CDCl3) of **36yβ**

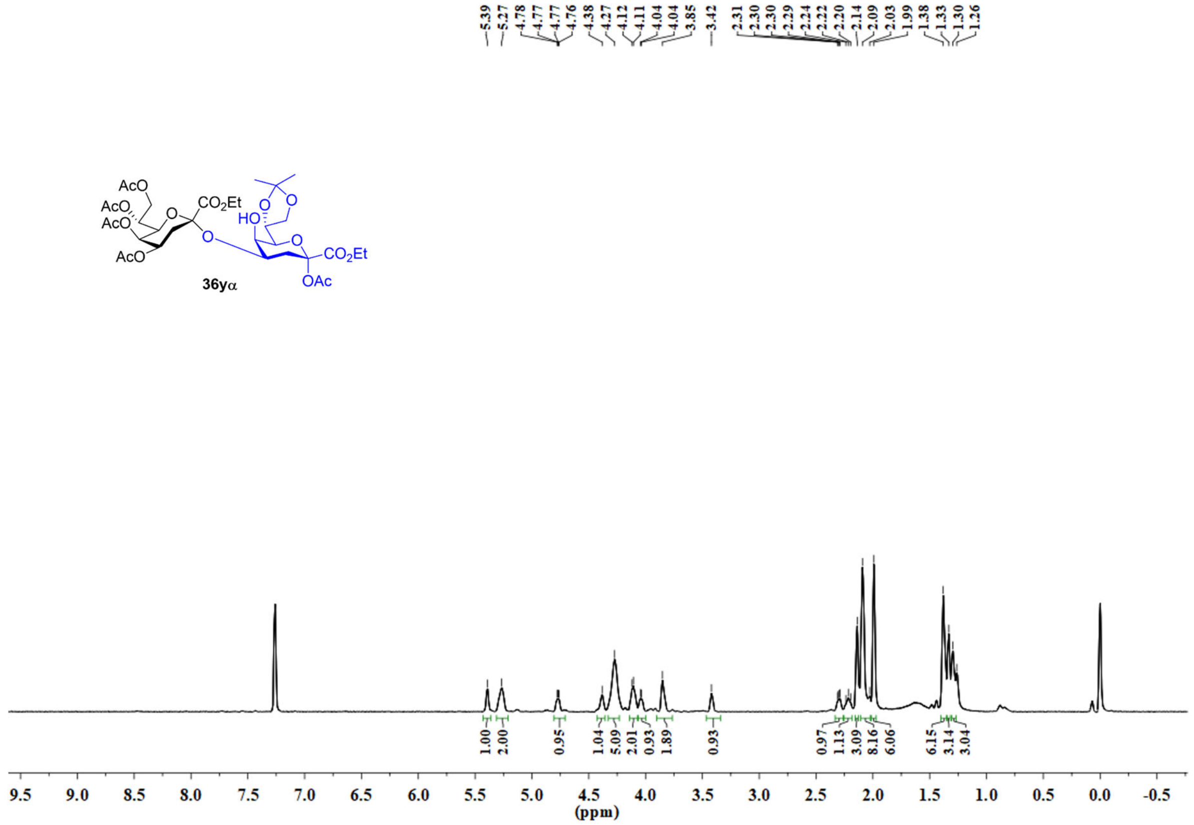

Fig. S96 ^{1}H NMR (600 MHz, CDCl3) of **36yα**

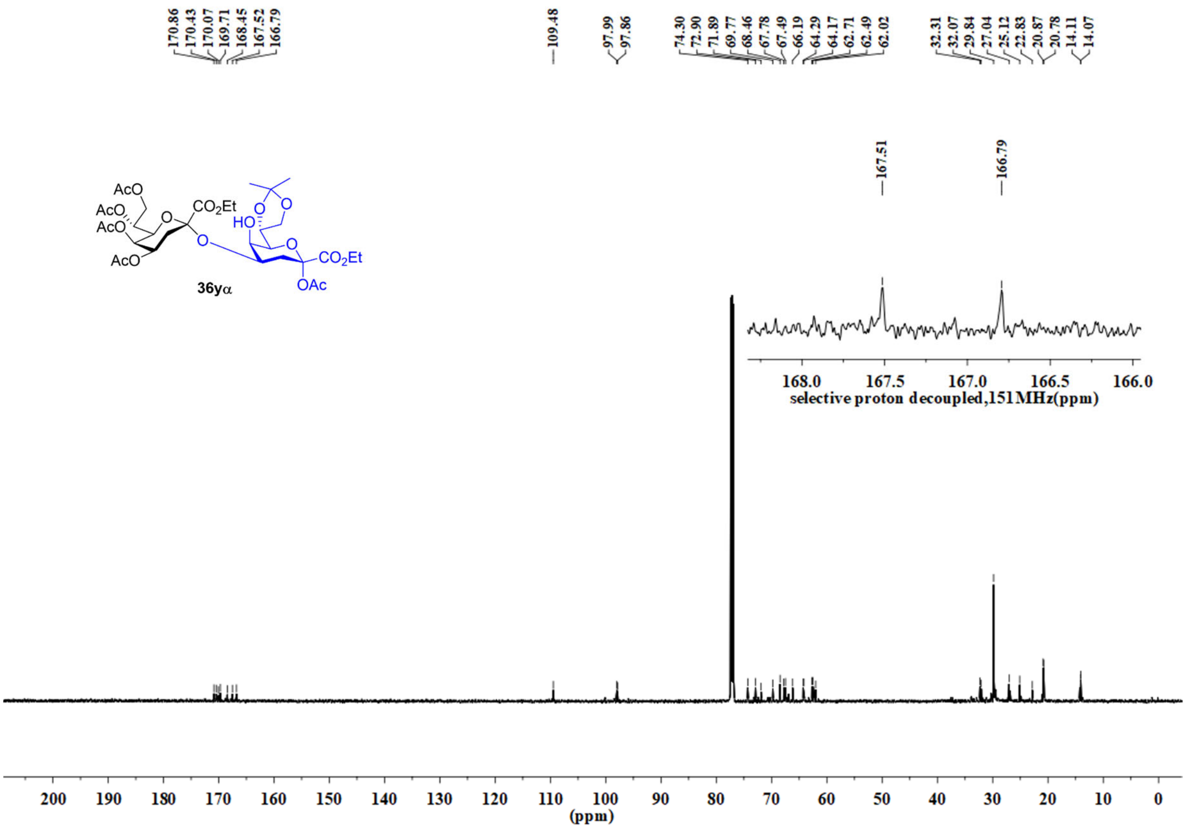

Fig. S97 ^{13}C NMR (151 MHz, CDCl$_3$) and

selective proton decoupled ^{13}C NMR (151 MHz, CDCl$_3$) of **36yα**

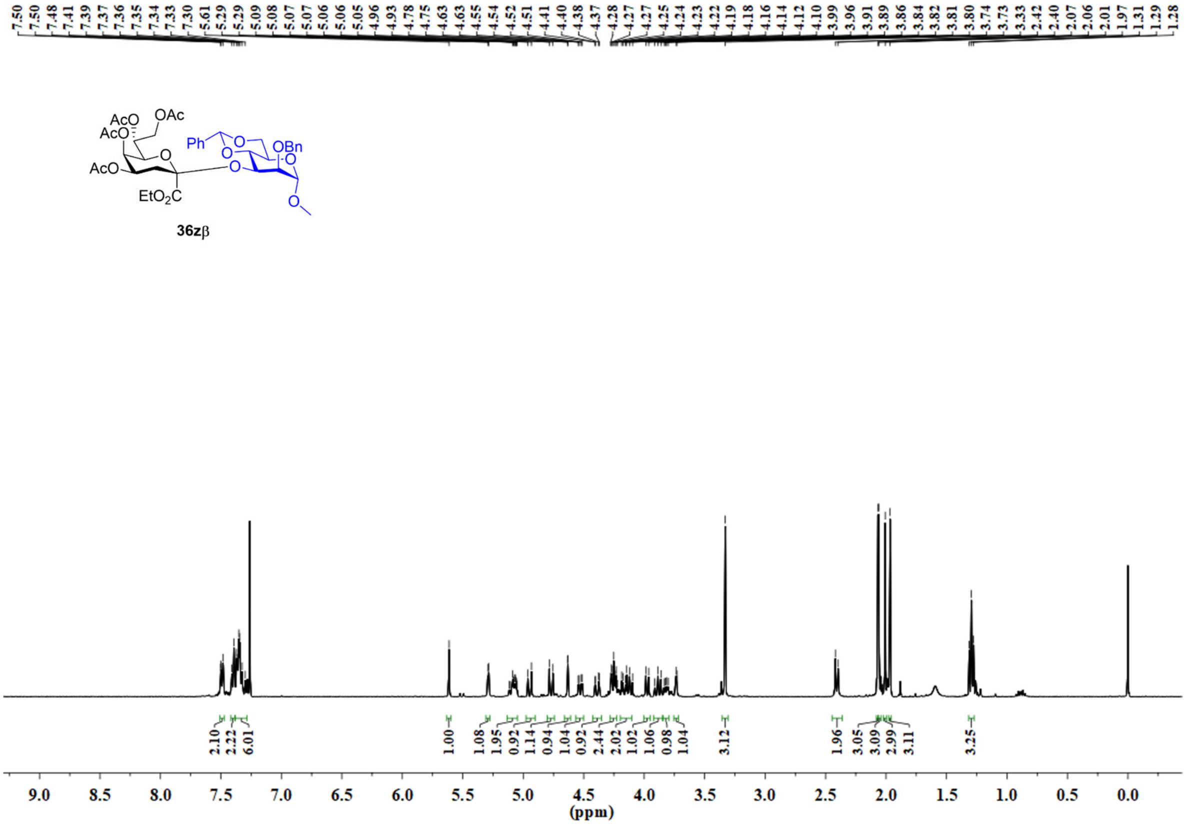

Fig. S98 ^{1}H NMR (400 MHz, CDCl$_3$) of **36zβ**

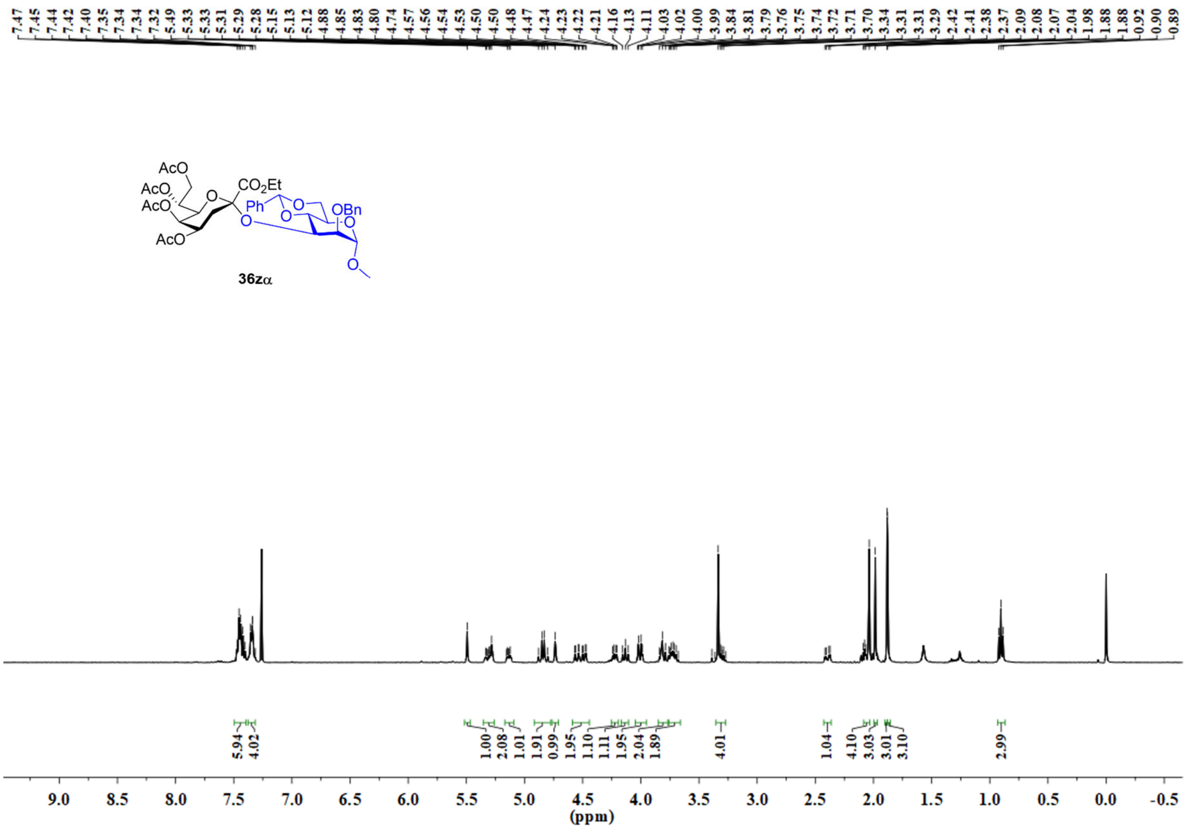

Fig. S99 ^{1}H NMR (400 MHz, CDCl3) of **36zα**

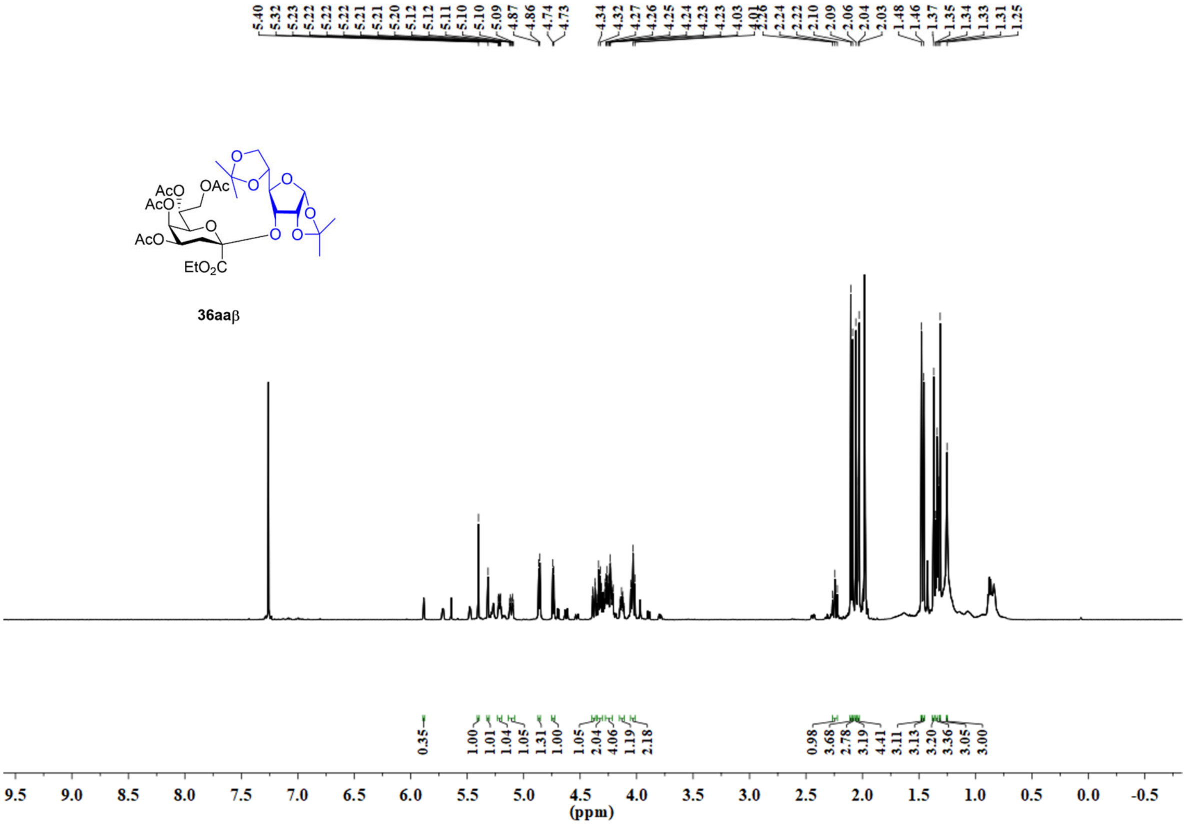

Fig. S100 ^{1}H NMR (600 MHz, CDCl3) of **36aaβ**

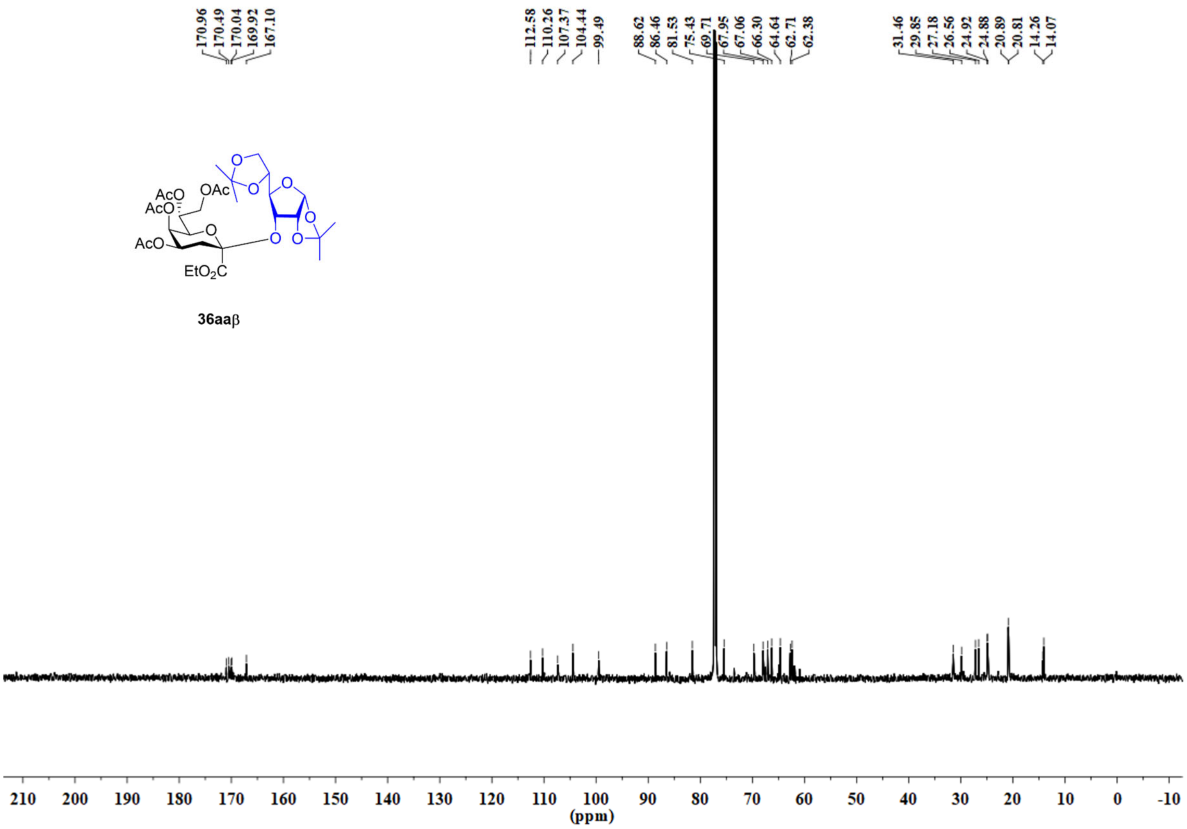

Fig. S101 ^{13}C NMR (151 MHz, $CDCl_3$) of **36aaβ**

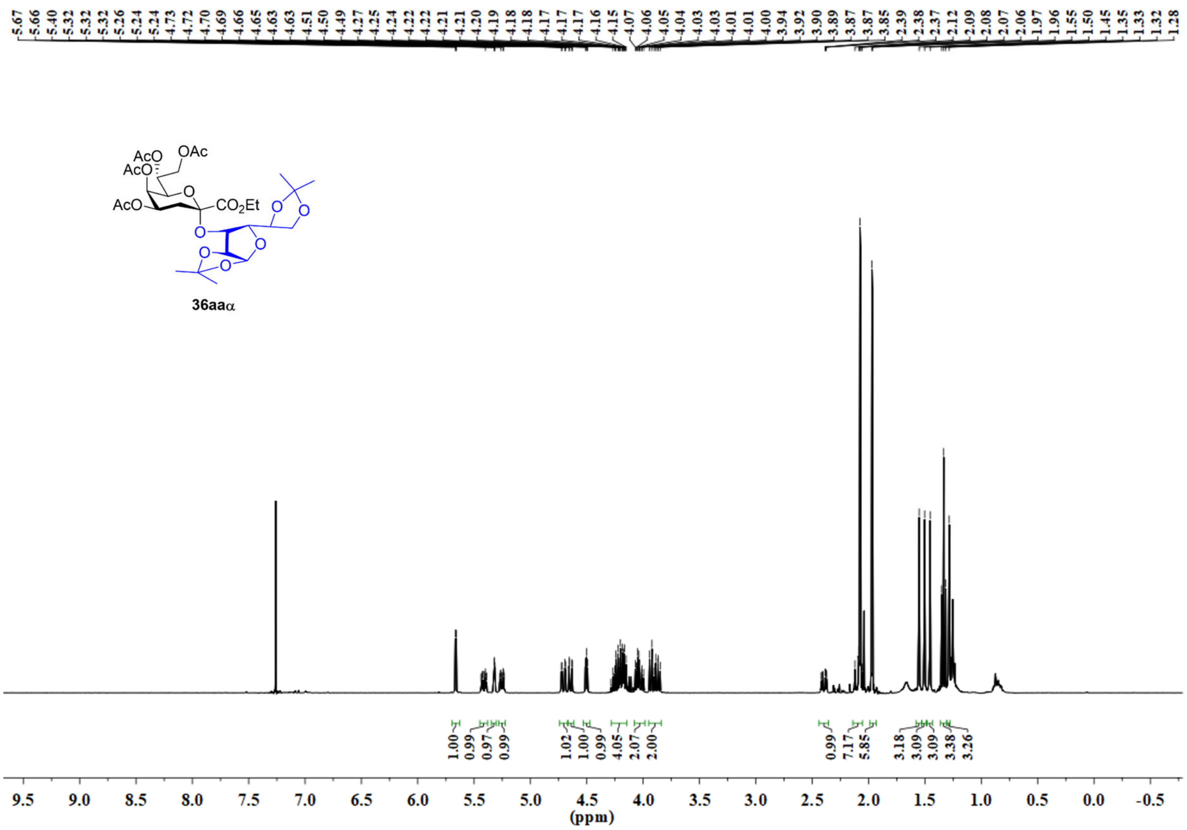

Fig. S102 ^{1}H NMR (400 MHz, $CDCl_3$) of **36aaα**

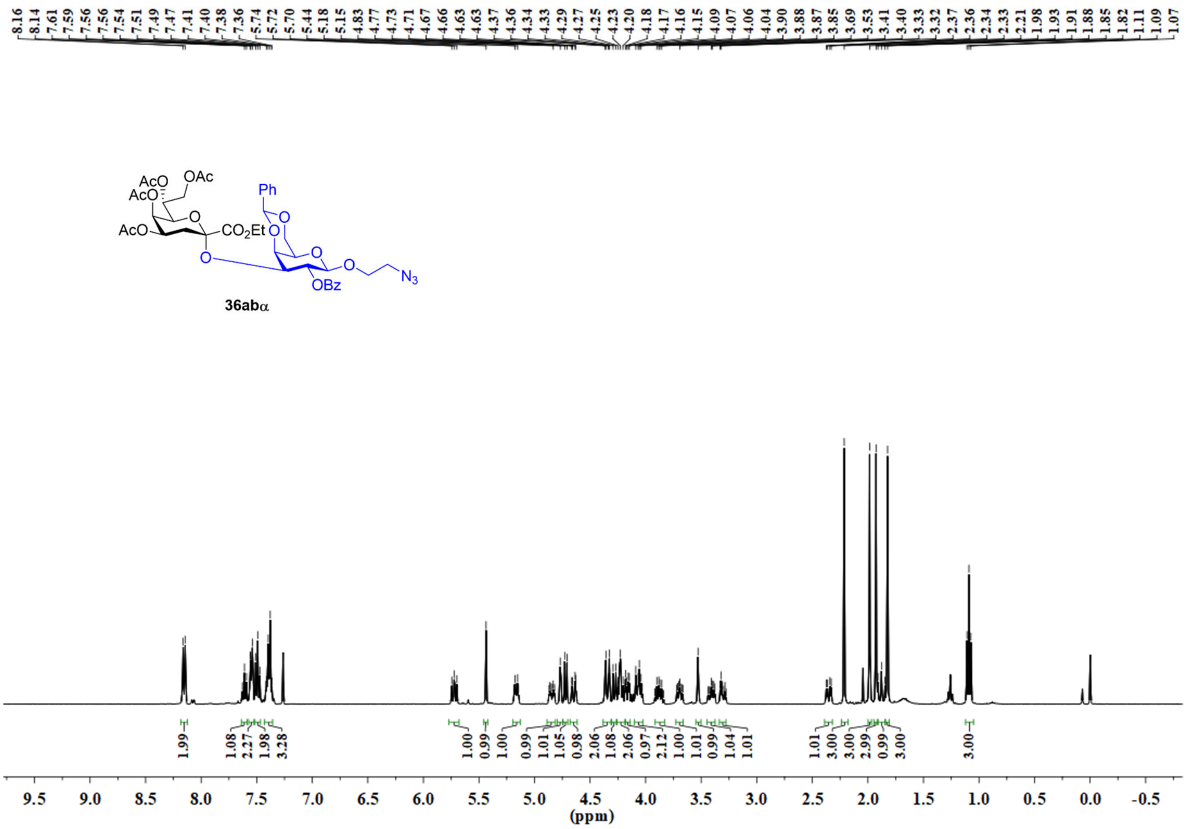

Fig. S103 ^{1}H NMR (400 MHz, $CDCl_3$) of **36abα**

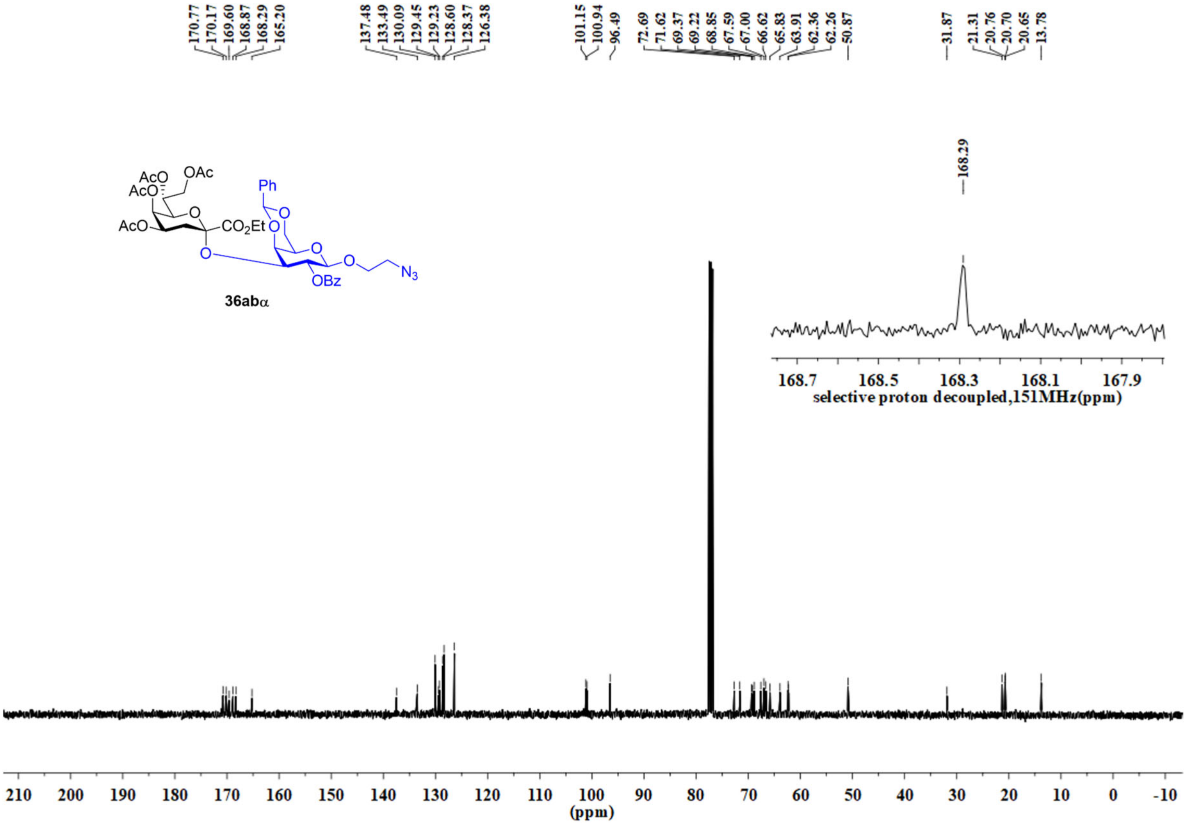

Fig. S104 ^{13}C NMR (101 MHz, $CDCl_3$) and

selective proton decoupled ^{13}C NMR (151 MHz, $CDCl_3$) of **36abα**

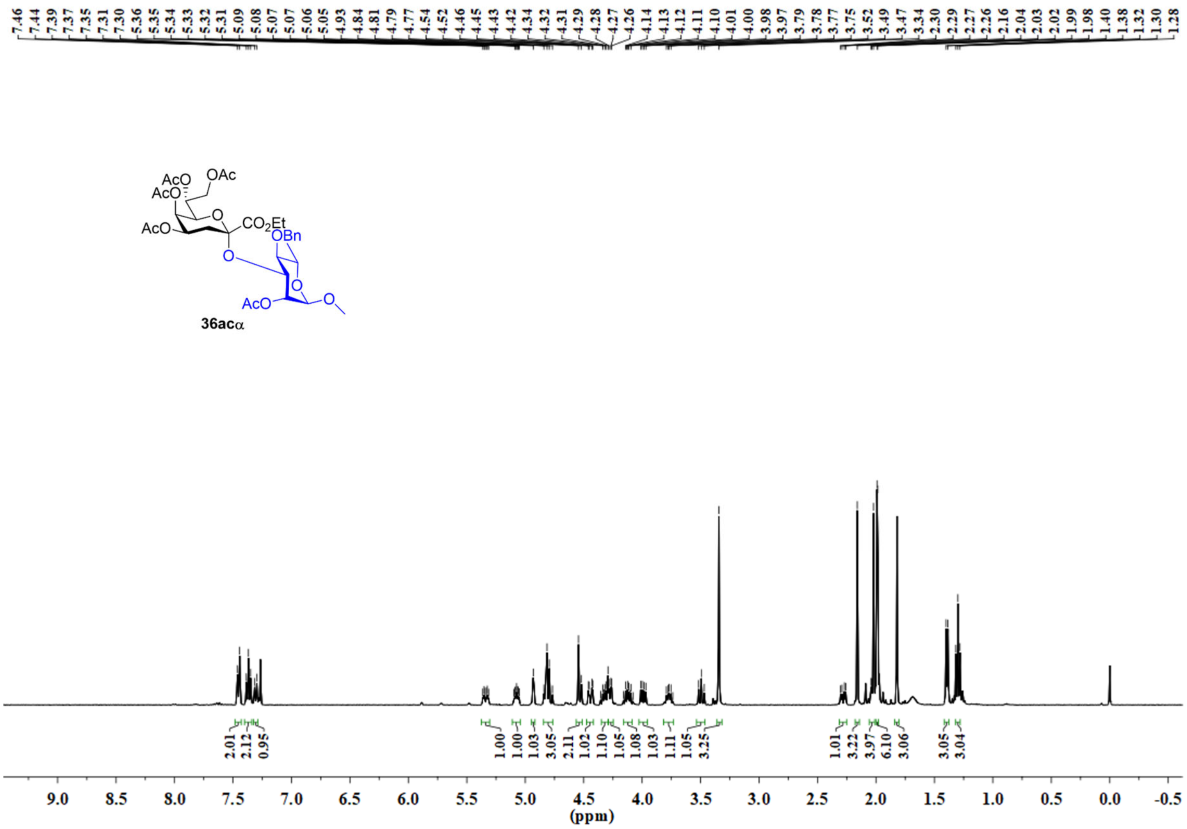

Fig. S105 ^{1}H NMR (400 MHz, $CDCl_3$) of **36acα**

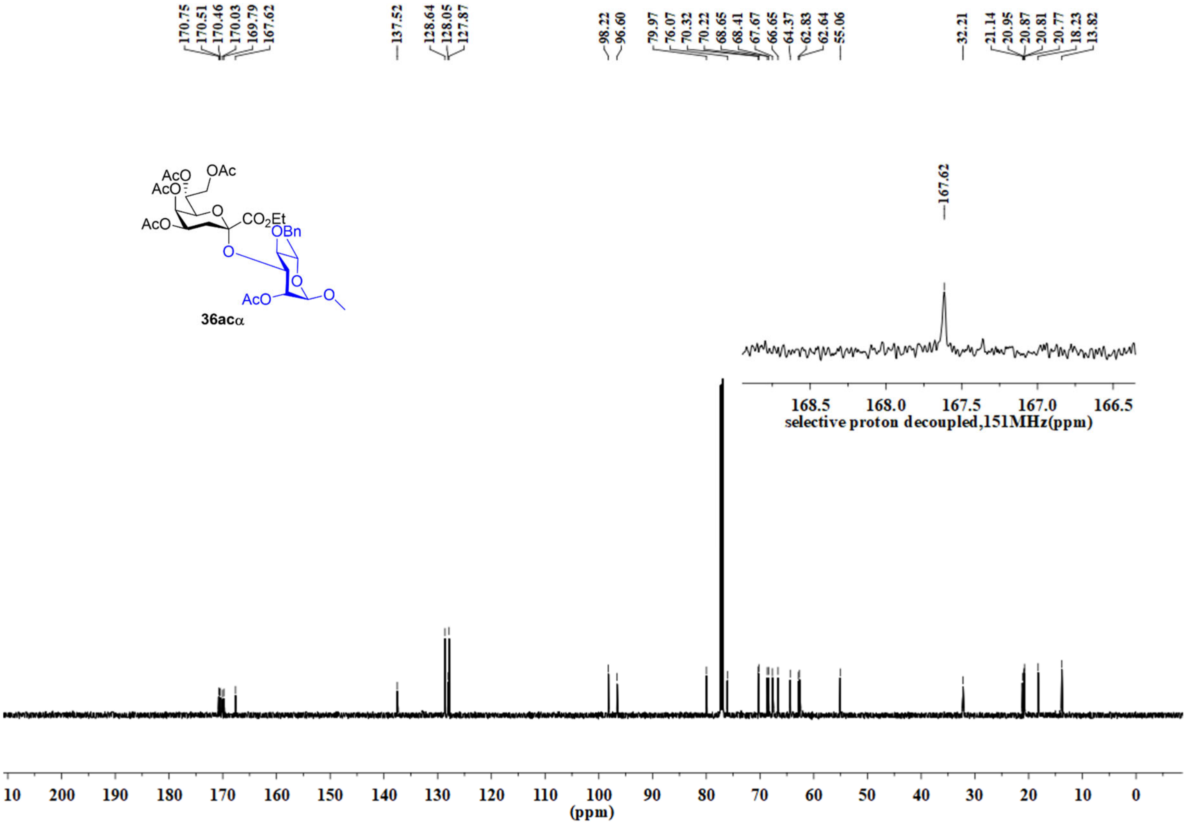

Fig. S106 ^{13}C NMR (151 MHz, $CDCl_3$) and

selective proton decoupled ^{13}C NMR (151 MHz, $CDCl_3$) of **36acα**

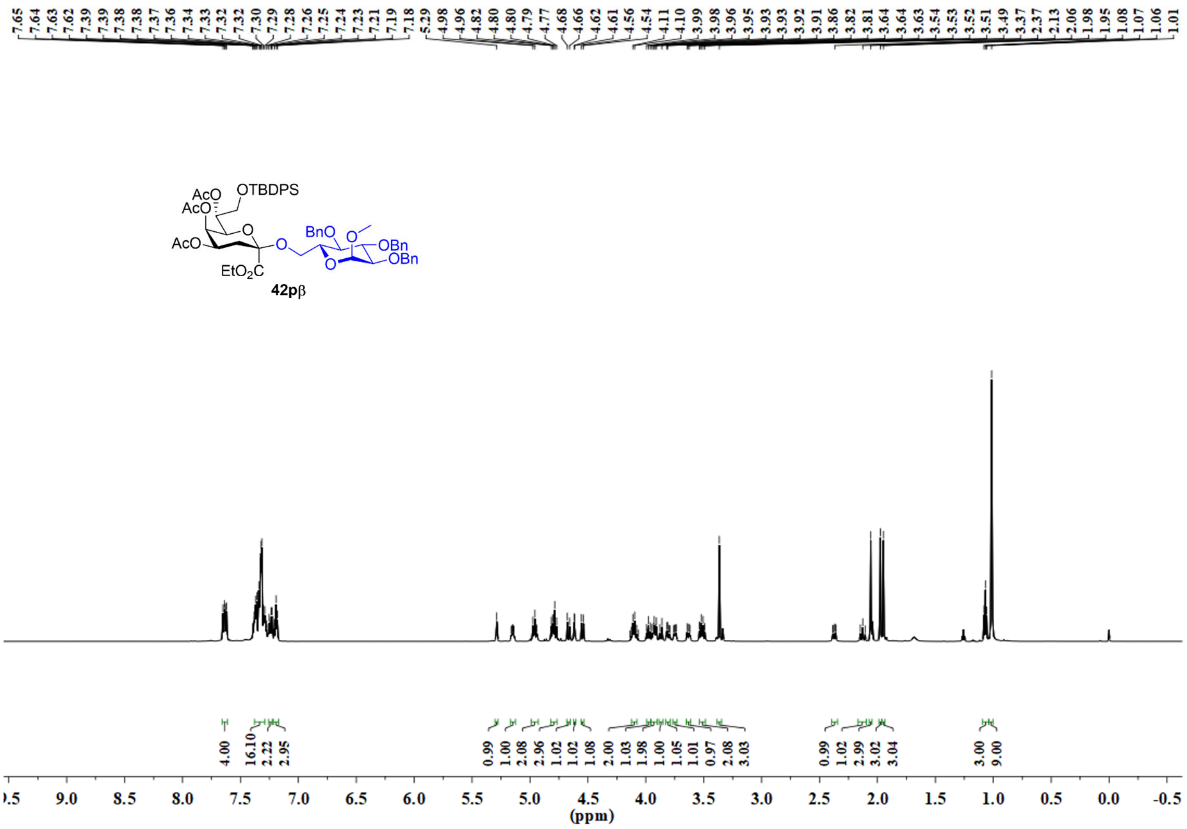

Fig. S107 ^{1}H NMR (600 MHz, $CDCl_3$) of **42pβ**

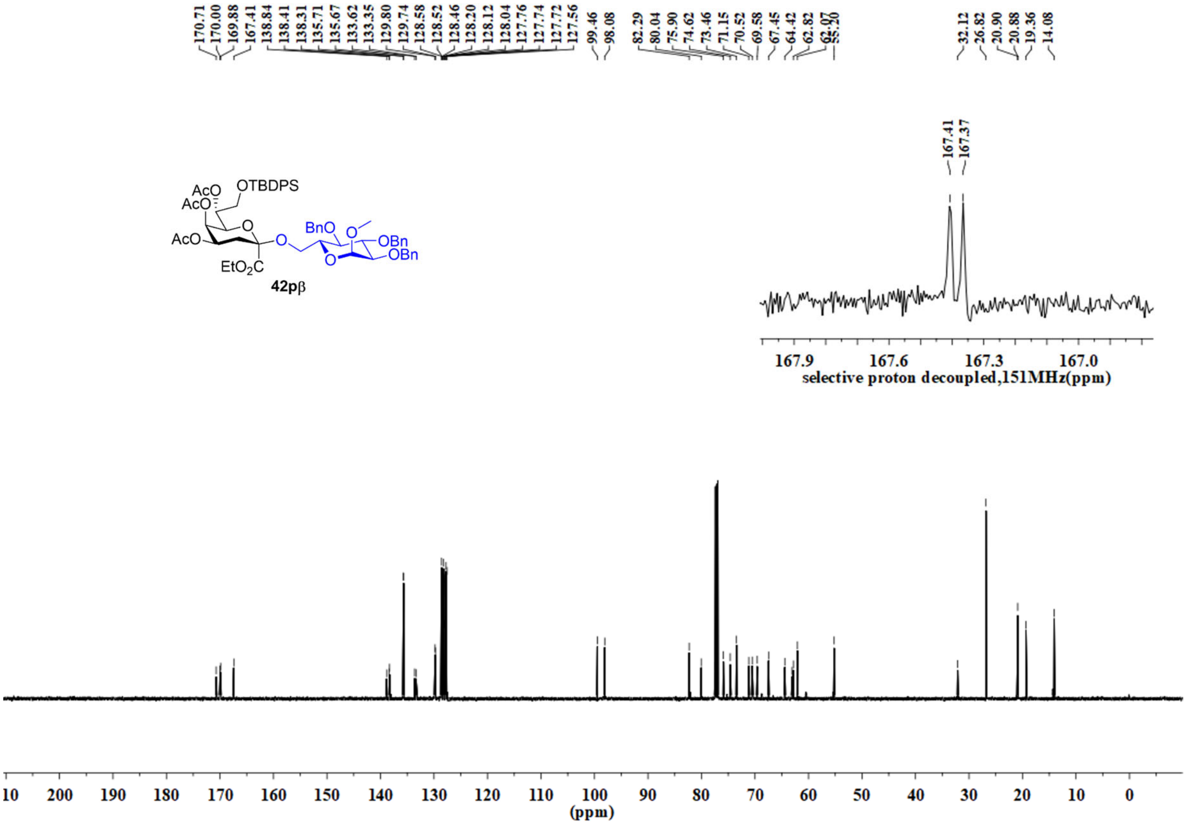

Fig. S108 ^{13}C NMR (151 MHz, $CDCl_3$) and
selective proton decoupled ^{13}C NMR (151 MHz, $CDCl_3$) of **42pβ**

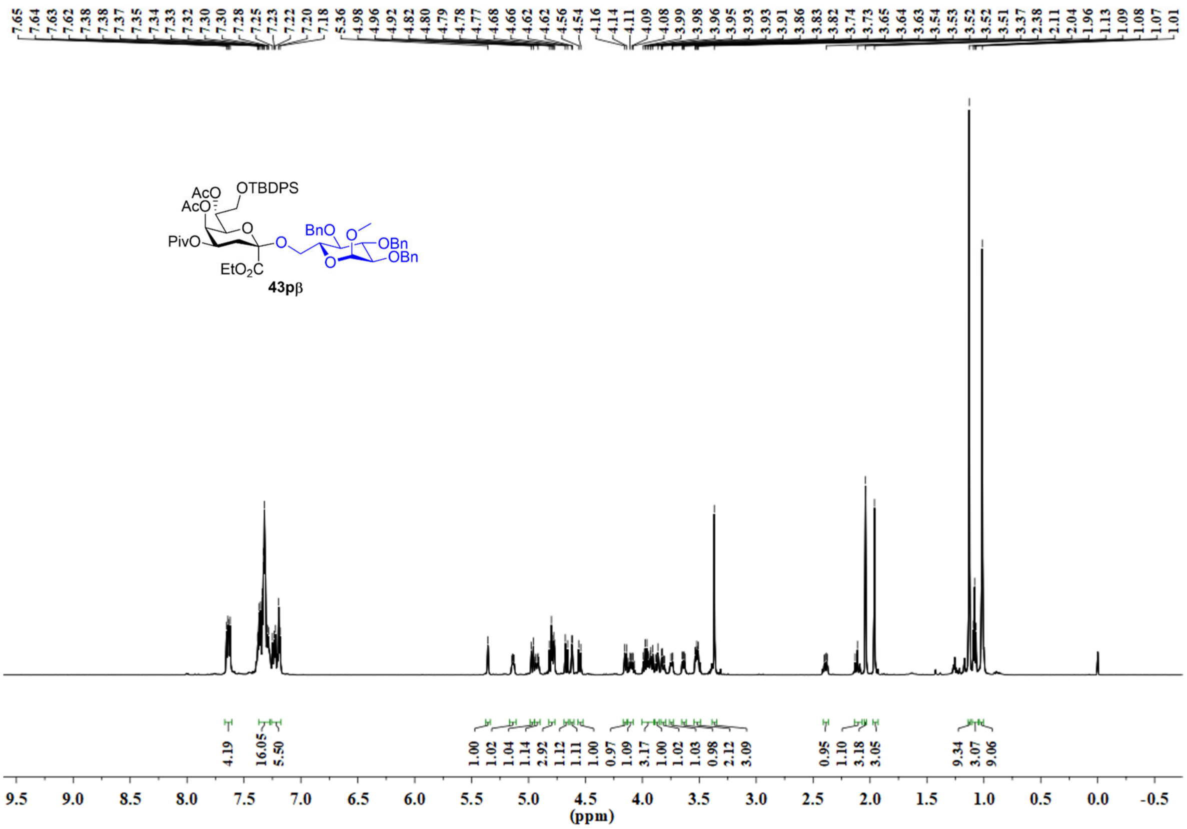

Fig. S109 ^{1}H NMR (600 MHz, CDCl$_3$) of **43pβ**

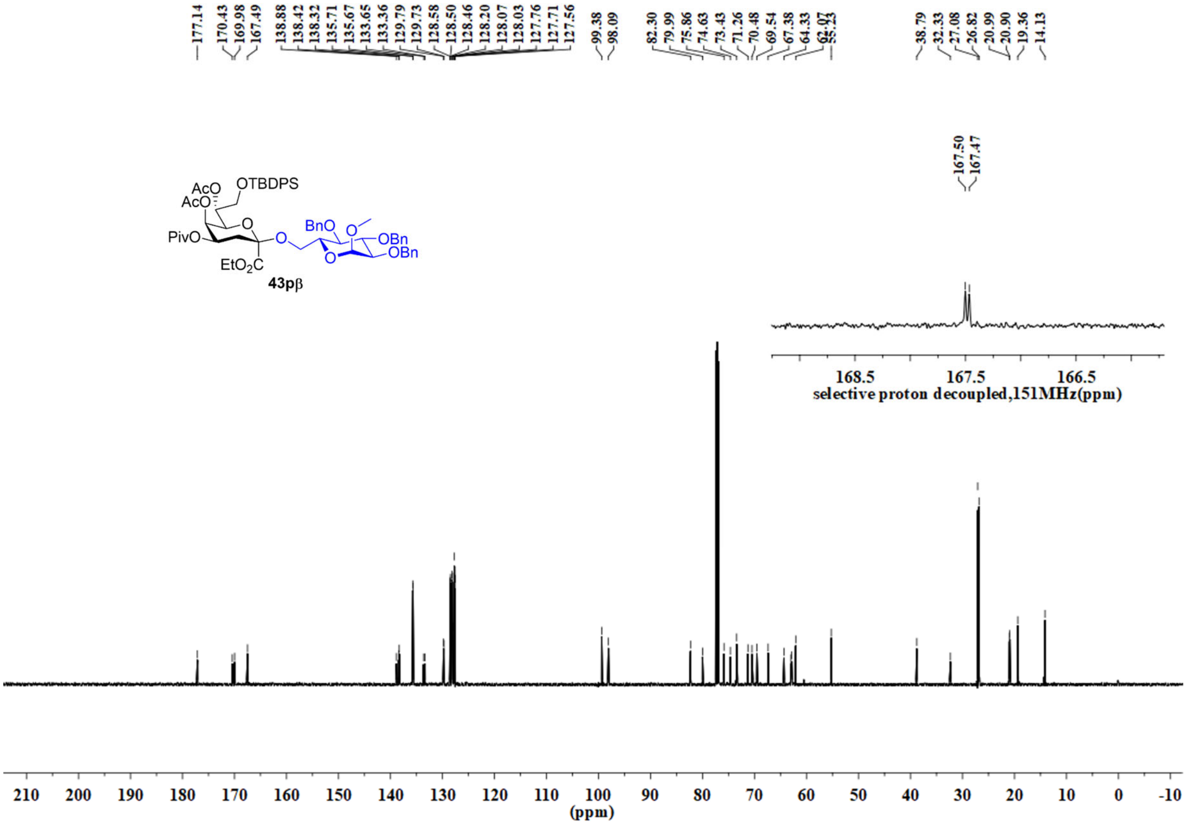

Fig. S110 ^{13}C NMR (151 MHz, CDCl$_3$) and

selective proton decoupled ^{13}C NMR (151 MHz, CDCl$_3$) of **43pβ**

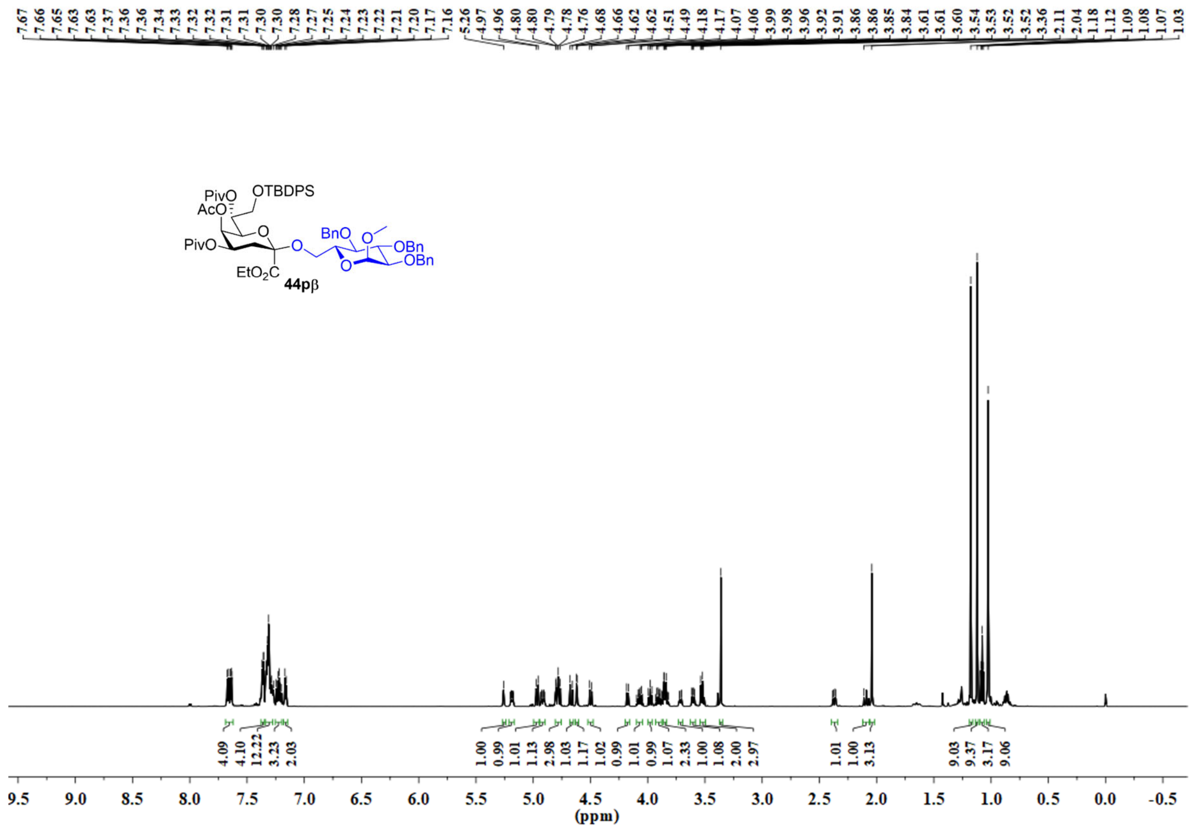

Fig. S111 ^{1}H NMR (600 MHz, $CDCl_3$) of **44pβ**

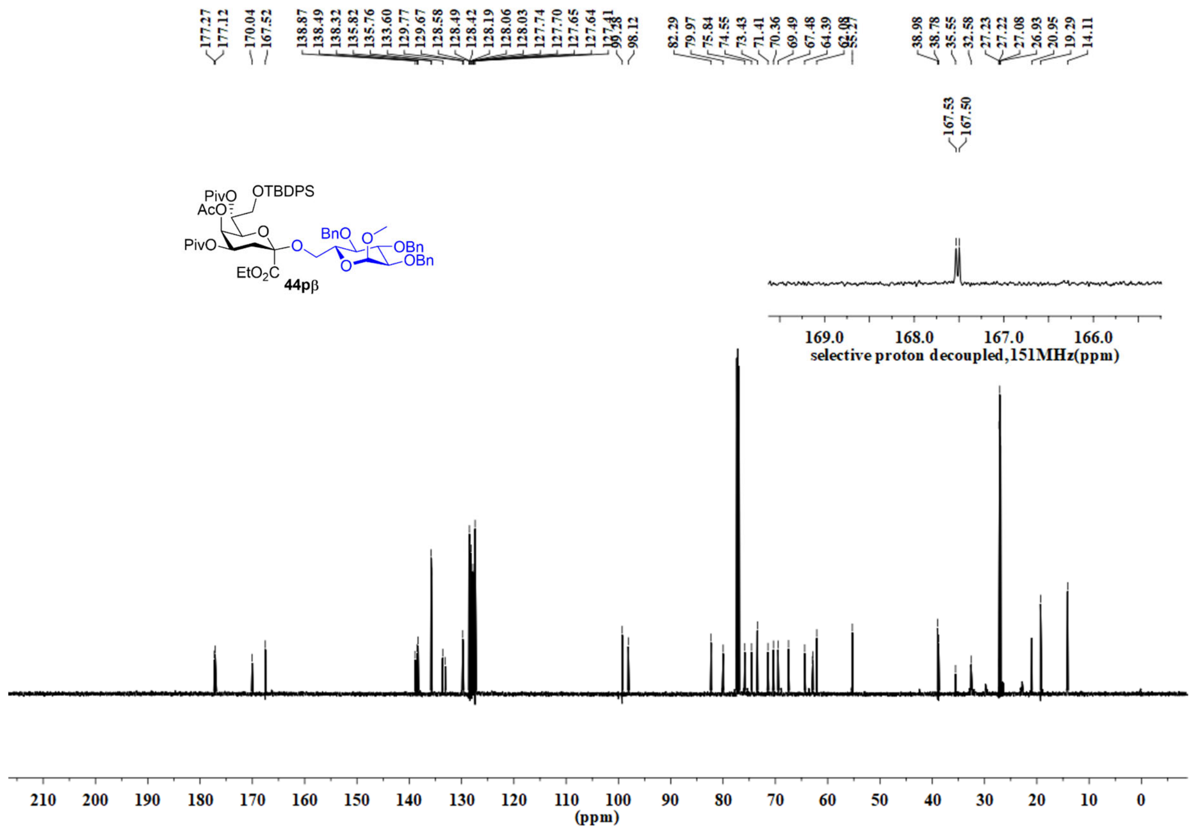

Fig. S112 ^{13}C NMR (151 MHz, $CDCl_3$) and

selective proton decoupled ^{13}C NMR (151 MHz, $CDCl_3$) of **44pβ**

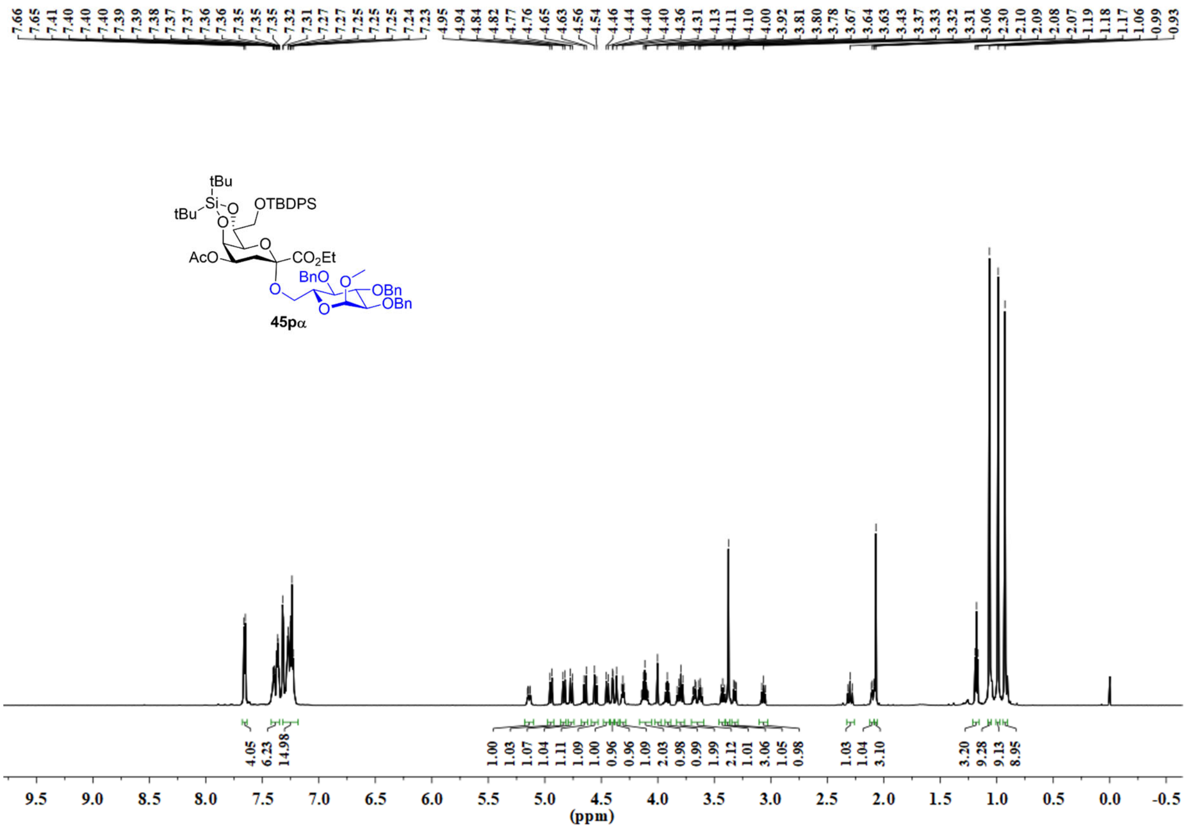

Fig. S113 ^{1}H NMR (600 MHz, $CDCl_3$) of **45pα**

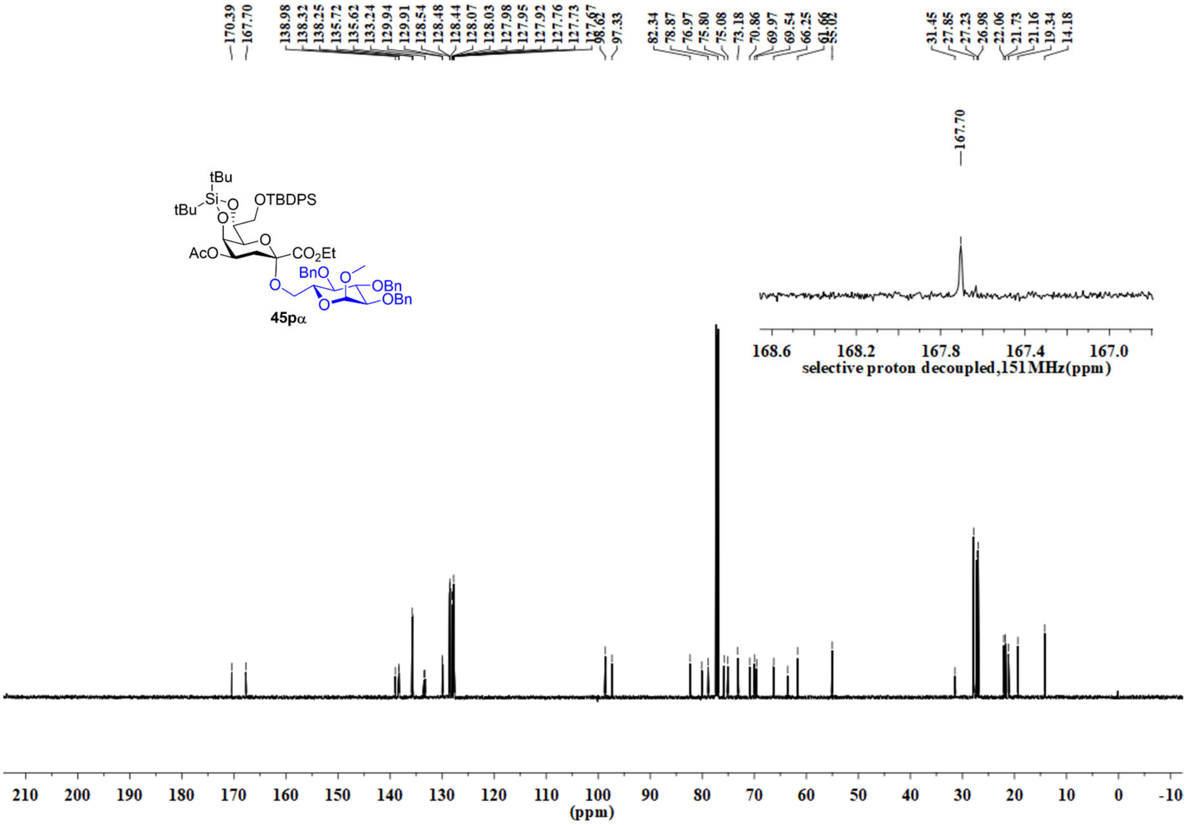

Fig. S114 ^{13}C NMR (101 MHz, $CDCl_3$) and

selective proton decoupled ^{13}C NMR (101 MHz, $CDCl_3$) of **45pα**

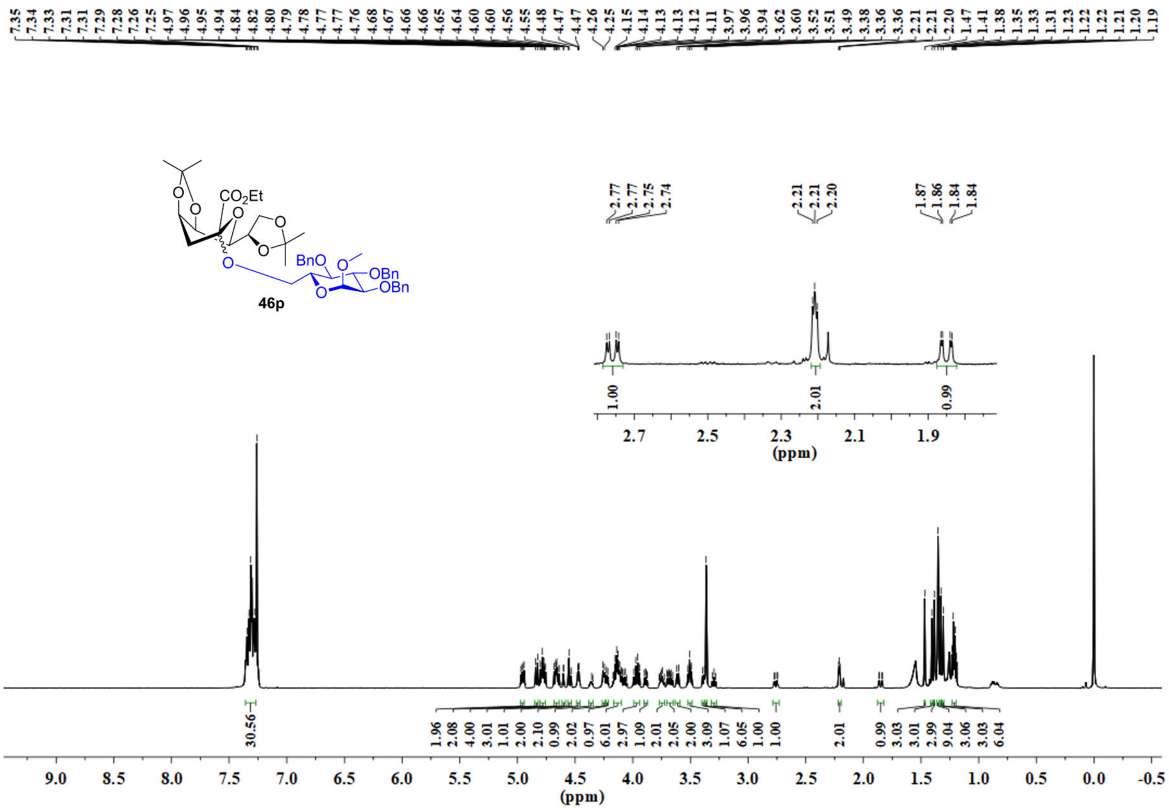

Fig. S115 ^{1}H NMR (600 MHz, $CDCl_3$) of **46p**

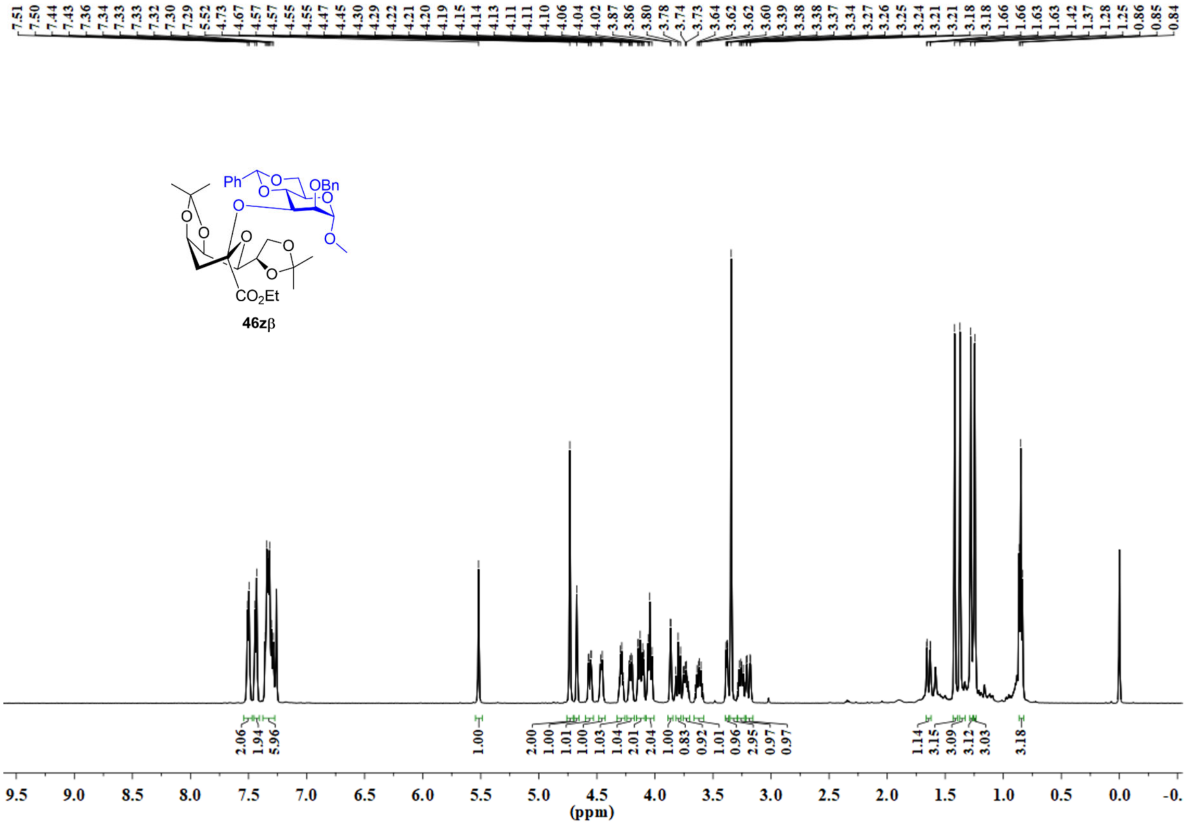

Fig. S116 ^{1}H NMR (500 MHz, $CDCl_3$) of **46zβ**

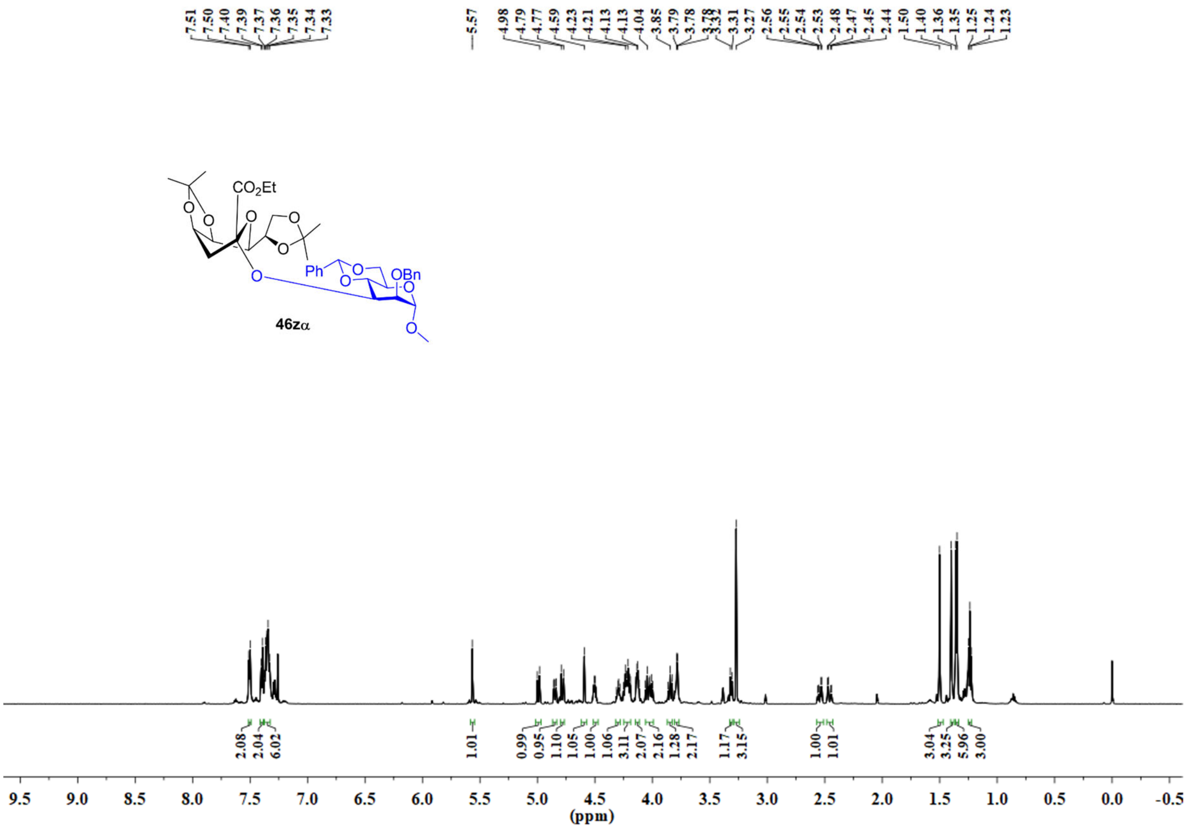

Fig. S117 ^{1}H NMR (600 MHz, $CDCl_3$) of **46zα**

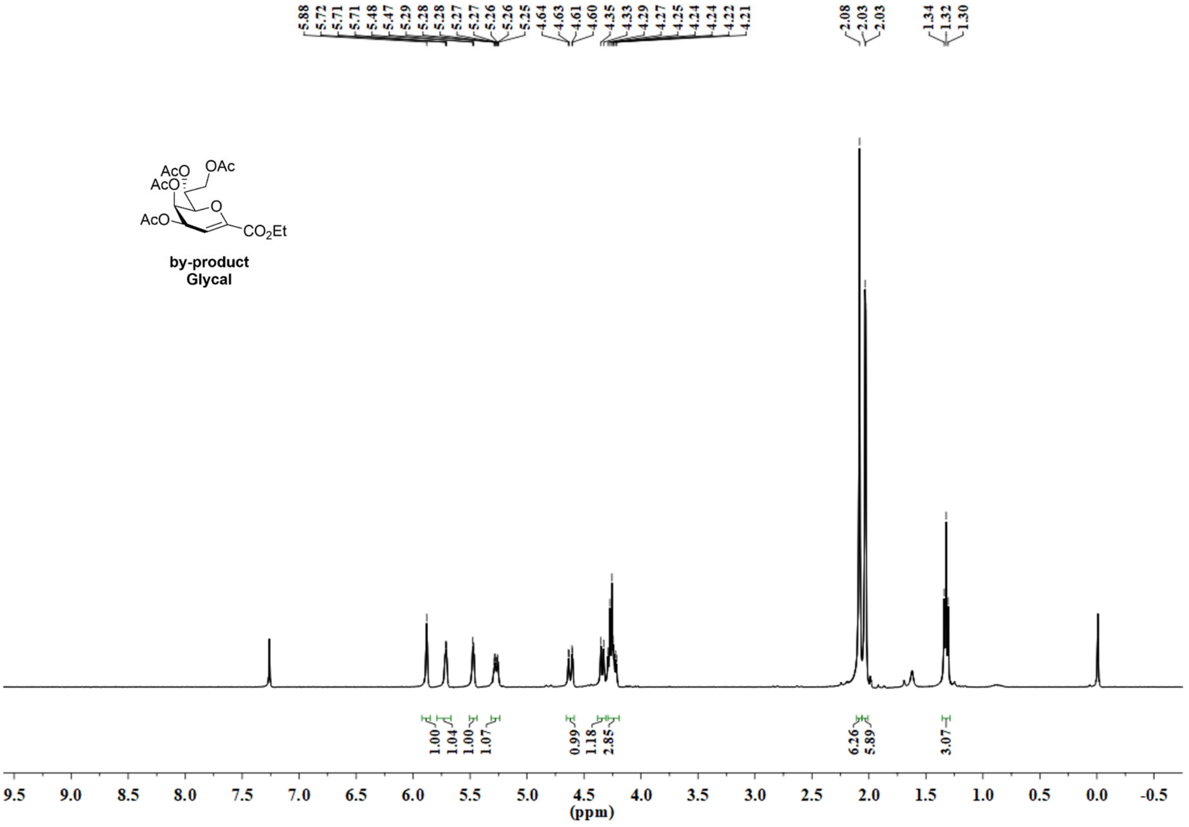

Fig. S118 ^{1}H NMR (400 MHz, $CDCl_3$) of **Glycal**

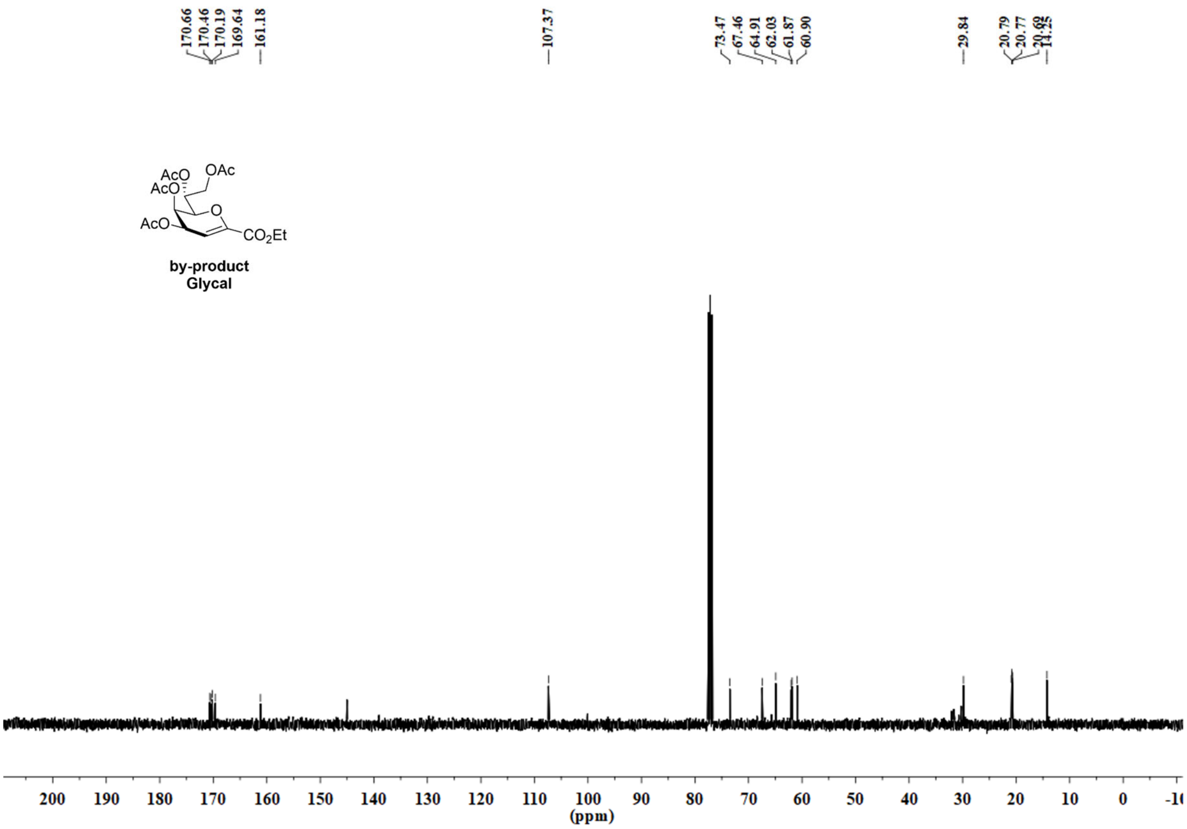

Fig. S119 ^{13}C NMR (101 MHz, $CDCl_3$) of **Glycal**

7 Reference

[1] (a) Imoto, M.; Kusunose, N.; Matsuura, Y.; Kusumoto, S.; Shiba, T. Preparation of novel pyranosyl fluorides of 3-deoxy-D-manno-2-octulosonic acid (KDO) feasible for synthesis of KDO α-glycosides. *Tetrahedron Lett.* **1987**, *28*, 6277–6280; (b) Boons, G. J. P. H.; van Delft, F. L.; van der Klein, P. A. M.; van der Marel, G. A.; van Boom, J. H. Synthesis of LD-Hep*p* and KDO containing di- and tetrasaccharide derivatives of Neisseria meningitidis inner-core region via iodonium ion promoted glycosidations. *Tetrahedron* **1992**, *48*, 885–904; (c) Mannerstedt, K.; Ekelöf, K.; Oscarson, S. Evaluation of thioglycosides of Kdo as glycosyl donors. *Carbohydr. Res.* **2007**, *342*, 631–637; (d) Yoshizaki, H.; Fukuda, N.; Sato, K.; Oikawa, M.; Fukase, K.; Suda, Y.; Kusumoto, S. First Total Synthesis of the Re-Type Lipopolysaccharide. *Angew. Chem. Int. Ed.* **2001**, *40*, 1475-1480; (e) Huang, J.; Huang, W.; Meng, X.; Wang, X.; Gao, P.; Yang, J. Stereoselective Synthesis of α-3-Deoxy-D-manno-oct-2-ulosonic Acid (α-Kdo) Glycosides Using 5,7-*O*-Di-tert-butylsilylene-Protected Kdo Ethyl Thioglycoside Donors. *Angew. Chem. Int. Ed.* **2015**, *54*, 10894–10898; (f) Hamajima, S.; Komura, N.; Tanaka, H.; Imamura, A.; Ishida, H.; Noguchi, H.; Ichiyanagi, T.; Ando, H. Full Stereocontrol in α-Glycosidation of 3-Deoxy-D-manno-2-octulosonic Acid (Kdo) Using Macrobicyclic Glycosyl Donors. *Org. Lett.* **2022**, *24*, 8672–8676; (g) Hamajima, S.; Komura, N.; Tanaka, H.; Imamura, A.; Ishida, H.; Ichiyanagi, T.; Ando, H. Investigation of the Protection of the C4 Hydroxyl Group in Macrobicyclic Kdo Donors. *Molecules* **2022**, *28*, 102.

[2] (a) Lou, Q.; Hua, Q.; Zhang, L.; Yang, Y. Dimethylformamide-Modulated Kdo Glycosylation for Stereoselective Synthesis of α-Kdo Glycosides. *Org. Lett.* **2020**, *22*, 981–985; (b) Miao, H.; Lu, S.; Chen, H.; Shang, J.; Zheng, J.; Yang, Y. Additive-assisted synthesis of α-Kdo glycosides with peracetylated glycosyl ynenoate as a donor. *Org. Biomol. Chem.* **2024**, *22*, 2365-2369.

[3] Zhang, J.; Gao, X.; Liu, S.; Geng, Z.; Chang, L.; Liu, Y.; Ma, Q.; Xing, G.; Liu, G.; Fang, D. α-Stereoselective 3-Deoxy-D-manno-oct-2-ulosonoic Acid (Kdo) O-Glycosylation with a *p*-Toluenethioglycoside Donor by the (p-Tol)$_2$SO/Tf$_2$O Preactivation Strategy. *Org. Lett.* **2023**, *25*, 4150-4155.

[4] (a) Schmidt, R. R.; Esswein, A. Simple Synthesis of KDO α-Glycosides by Anomerically Selective *O*-Alkylation. *Angew. Chem. Int. Ed.* **1988**, *27*, 1178-1180; (b) Esswein, A.; Rembold, H.; Schmidt, R. R. *O*-Alkylation at the anomeric centre for the stereoselective synthesis of Kdo-α-glycosides. *Carbohydr. Res.* **1990**, *200*, 287-305.

[5] (a) Pokorny, B.; Kosma, P. Scope and Limitations of 3-Iodo-Kdo Fluoride-Based Glycosylation Chemistry using N-Acetyl Glucosamine Acceptors. *Chemistryopen* **2015**, *4*, 722-728; (b) Ikeda, K.; Akamatsu, S.; Achiwa, K. A New Syhthesis of α-Glycosidically-linked Disaccharides Using 2-α-choloro-3-β-phenylthio KDO Derivatives. *Chem. Pharm. Bull.* **1990**, *38*, 279- 281; (c) Sun, A.; Li, Z.; Wang, Y.; Meng, S.; Zhang, X.; Meng, X.; Li, S.; Li, Z.; Li, Z. Stereocontrolled Synthesis of α-3-Deoxy-D-manno-oct-2-ulosonic Acid (α-Kdo) Glycosides Using C3-*p*-Tolylthio-Substituted Kdo Donors: Access to Highly Branched Kdo Oligosaccharides. *Angew. Chem. Int. Ed.* **2024**, *63*, e202313985; (d) Sun, A.; Li, Z.; Li, S.; Meng, X.; Li, Z.; Li, Z., Stereoselective synthesis of α-3-

deoxy-ᴅ-manno-oct-2-ulosonic acid (α-Kdo) derivatives using a C3-p-tolylthio-substituted Kdo fluoride donor. *Chin. Chem. Lett.* **2025**, *36*, 109972.

[6] Mong, K. T.; Pradhan, T. K.; Chiu, C.; Hung, W.; Chen, C.; Wang, Y. (2-Ketulosonyl)onate 2,3-*O*-thionocarbonate donors for the synthesis of KO and KDO α-glycosides and a one-pot glycosylation method for 2-keto acid donors. *Org. Chem. Front.* **2020**, *7*, 2179-2186.

[7] Ekelöf, K.; Oscarson, S. Synthesis of 2-(4-aminophenyl)ethyl 3-deoxy-5-*O*-(3,4,6-tri-*O*-β-D-glucopyranosyl-α-d-glucopyranosyl)-α-D-manno-oct-2-ulopyranosidonic acid, a highly branched pentasaccharide corresponding to structures found in lipopolysaccharides from Moraxella catarrhalis. *Carbohydr. Res.* **1995**, *278*, 289-300.

[8] Pradhan, T. K.; Lin, C. C.; Mong, K. T., Preparation of a Protected 3-Deoxy-D-manno-oct-2-ulosonate Glycal Donor for the Synthesis of β-KDO-Containing Oligosaccharides. *Org. Lett.* **2014**, *16*, 1474-1477.

[9] Mazur, M.; Barycza, B.; Andriamboavonjy, H.; Lavoie, S.; Tamigney Kenfack, M.; Laroussarie, A.; Blériot, Y.; Gauthier, C. 4′-Methoxyphenacyl-Assisted Synthesis of β-Kdo Glycosides. *J. Org. Chem.* **2016**, *81*, 10585-10599.

[10] Huang, W.; Zhou, Y.; Pan, X.; Zhou, X.; Lei, J.; Liu, D.; Chu, Y.; Yang, J. Stereodirecting Effect of C5-Carboxylate Substituents on the Glycosylation Stereochemistry of 3-Deoxy-D-manno-oct-2-ulosonic Acid (Kdo) Thioglycoside Donors: Stereoselective Synthesis of α- and β-Kdo Glycosides. *J. Am. Chem. Soc.* **2018**, *140*, 3574–3582.

[11] Ngoje, P.; Crich, D. Stereocontrolled Synthesis of the Equatorial Glycosides of 3-Deoxy-D-manno-oct-2-ulosonic Acid: Role of Side Chain Conformation. *J. Am. Chem. Soc.* **2020**, *142*, 7760–7764.

[12] Pramanik, S.; Mondal, S.; Chinarev, A.; Bovin, N. V.; Saha, J. Hydroxamate-directed access to β-Kdo glycosides. *Chem. Commun.* **2023**, *59*, 10028-10031.

[13] Teegardin, K.; Day, J. I.; Chan, J.; Weaver, J. Advances in Photocatalysis: A Microreview of Visible Light Mediated Ruthenium and Iridium Catalyzed Organic Transformations. *Org. Process Res. Dev.* **2016,** *20*, 1156-1163.

[14] Speckmeier, E.; Fischer, T. G.; Zeitler, K. A Toolbox Approach To Construct Broadly Applicable Metal-Free Catalysts for Photoredox Chemistry: Deliberate Tuning of Redox Potentials and Importance of Halogens in Donor–Acceptor Cyanoarenes. *J. Am. Chem. Soc.* **2018**, *140*, 15353–15365.

[15] Dutta, S.; Erchinger, J. E.; Strieth-Kalthoff, F.; Kleinmans, R.; Glorius, F. Energy transfer photocatalysis: exciting modes of reactivity. *Chem. Soc. Rev.* **2024**, *53*, 1068–1089.

[16] Liu, T.; Li, T.; Tea, Z. Y.; Wang, C.; Shen, T.; Lei, Z.; Chen, X.; Zhang, W.; Wu, J. Modular assembly of arenes, ethylene and heteroarenes for the synthesis of 1,2-arylheteroaryl ethanes. *Nat. Chem.* **2024**. DOI: https://doi.org/10.1038/s41557-024-01560-7.

[17] Bortolato, T.; Cuadros, S.; Simionato, G.; Dell Amico, L., The advent and development of organophotoredox catalysis. *Chem. Comm.* **2022**, *58*, 1263-1283.

[18] Stevenson, B. G.; Spielvogel, E. H.; Loiaconi, E. A.; Wambua, V. M.; Nakhamiyayev, R. V.; Swierk, J. R. Mechanistic Investigations of an α-Aminoarylation Photoredox Reaction. *J. Am. Chem. Soc.* **2021,** *143*, 8878-8885.

[19] Mao, R.; Xiong, D.; Guo, F.; Li, Q.; Duan, J.; Ye, X., Light-driven highly efficient glycosylation reactions. *Org. Chem. Front.* **2016***, 3,* 737-743.

[20] Zhang, C.; Wang, H.; Klein, A.; Biewer, C.; Stirnat, K.; Yamaguchi, Y.; Xu, L.; Gomez-Benitez, V.; Vicic, D. A. A Five-Coordinate Nickel(II) Fluoroalkyl Complex as a Precursor to a Spectroscopically Detectable Ni(III) Species. *J. Am. Chem. Soc.* **2013,** *135*, 8141-8144.

[21] Lu, T.; Chen, Q., Shermo: A general code for calculating molecular thermochemistry properties. *Comput. Theor. Chem.* **2021**, *1200*, 113249.

[22] Chambers, D. J.; Evans, G. R.; Fairbanks, A. J. An approach to the synthesis of α-(1-6)-C-disaccharides by tandem Tebbe methylenation and Claisen rearrangement. *Tetrahedron* **2005**, *61*, 7184-7192.

[23] Wang, Z.; Zhou, L.; El-Boubbou, K.; Ye, X.; Huang, X. Multi-Component One-Pot Synthesis of the Tumor-Associated Carbohydrate Antigen Globo-H Based on Preactivation of Thioglycosyl Donors. *J. Org. Chem.* **2007**, *72*, 6409-6420.

[24] Tanaka, N.; Ogawa, I.; Yoshigase, S.; Nokami, J. Regioselective ring opening of benzylidene acetal protecting group(s) of hexopyranoside derivatives by DIBAL-H. *Carbohydr. Res.* **2008**, *343*, 2675-2679.

[25] Mi, X.; Lou, Q.; Fan, W.; Zhuang, L.; Yang, Y. Gold(I)-catalyzed synthesis of β-Kdo glycosides using Kdo ortho-hexynylbenzoate as donor. *Carbohydr. Res.* **2017**, *448*, 161-165.

[26] Gaussian 16, Revision A.03, Frisch, M. J.; Trucks, G. W.; Schlegel, H. B.; Scuseria, G. E.; Robb, M. A.; Cheeseman, J. R.; Scalmani, G.; Barone, V.; Petersson, G. A.; Nakatsuji, H.; Li, X.; Caricato, M.; Marenich, A. V.; Bloino, J.; Janesko, B. G.; Gomperts, R.; Mennucci, B.; Hratchian, H. P.; Ortiz, J. V.; Izmaylov, A. F.; Sonnenberg, J. L.; Williams-Young, D.; Ding, F.; Lipparini, F.; Egidi, F.; Goings, J.; Peng, B.; Petrone, A.; Henderson, T.; Ranasinghe, D.; Zakrzewski, V. G.; Gao, J.; Rega, N.; Zheng, G.; Liang, W.; Hada, M.; Ehara, M.; Toyota, K.; Fukuda, R.; Hasegawa, J.; Ishida, M.; Nakajima, T.; Honda, Y.; Kitao, O.; Nakai, H.; Vreven, T.; Throssell, K.; Montgomery, J. A., Jr.; Peralta, J. E.; Ogliaro, F.; Bearpark, M. J.; Heyd, J. J.; Brothers, E. N.; Kudin, K. N.; Staroverov, V. N.; Keith, T. A.; Kobayashi, R.; Normand, J.; Raghavachari, K.; Rendell, A. P.; Burant, J. C.; Iyengar, S. S.; Tomasi, J.; Cossi, M.; Millam, J. M.; Klene, M.; Adamo, C.; Cammi, R.; Ochterski, J. W.; Martin, R. L.; Morokuma, K.; Farkas, O.; Foresman, J. B.; Fox, D. J. Gaussian, Inc., Wallingford CT, 2016.

[27] Lee, C.; Yang, W.; Parr, R. G. Development of the Colle-Salvetti correlation-energy formula into a functional of the electron density. *Phys. Rev. B* **1988**, *37*, 785–789.

[28] Becke, A. D. Density-functional thermochemistry. III. The role of exact exchange. *J. Chem. Phys.* **1993**, *98*, 5648–5652.

[29] Grimme, S.; Antony, J.; Ehrlich, S.; Krieg, H. A consistent and accurate ab initio parametrization of density functional dispersion correction (DFT-D) for the 94 elements H-Pu. *J. Chem. Phys.* **2010**, *132*, 154104.

[30] Clark, T.; Chandrasekhar, J.; Spitznagel, G. W.; Schleyer, P. V. R. Efficient diffuse function-augmented basis sets for anion calculations. III. The 3-21+G basis set for first-row elements, Li-F. *J. Comput. Chem.* **1983**, *4*, 294–301.

[31] Francl, M. M.; Pietro, W. J.; Hehre, W. J.; Binkley, J. S.; Gordon, M. S.; DeFrees, D. J.; Pople, J. A. Self-consistent molecular orbital methods. XXIII. A polarization-type basis set for second-row elements. *J. Chem. Phys.* **1982**, *77*, 3654–3665.

[32] Krishnan, R.; Binkley, J. S.; Seeger, R.; Pople, J. A. Self-consistent molecular orbital methods. XX. A basis set for correlated wave functions. *J. Chem. Phys.* **1980**, *72*, 650–654.

[33] McLean, A. D.; Chandler, G. S. Contracted Gaussian basis sets for molecular calculations. I. Second row atoms, Z=11-18. *J. Chem. Phys.* **1980**, *72*, 5639–5648.

[34] Spitznagel, G. W.; Clark, T.; von Ragué Schleyer, P.; Hehre, W. J. An evaluation of the performance of diffuse function-augmented basis sets for second row elements, Na-Cl. *J. Comput. Chem.* **1987**, *8*, 1109–1116.

[35] Marenich, A. V.; Cramer, C. J.; Truhlar, D. G. Universal solvation model based on solute electron density and on a continuum model of the solvent defined by the bulk dielectric constant and atomic surface tensions. *J. Phys. Chem. B* **2009**, *113*, 6378–6396.

[36] a) Fukui, K., Formulation of the reaction coordinate. *J. Phys. Chem.* **1970**, *74*, 4161-4164; b) Fukui, K., The path of chemical reactions - the IRC approach. *Acc. Chem. Res.* **1981**, *14*, 363 368.
